# Investigating the Landscape of C6-Azaindole Side Chain on the Epoxymorphinan Skeleton via the Nitrogen Walk Concept: A Strategy to Enhance Drug-Like Properties

**DOI:** 10.1021/acs.jmedchem.5c02175

**Published:** 2026-01-22

**Authors:** Logan Neel, Hongguang Ma, Ahmed Reda, Mengchu Li, Rachael Flammia, Samuel Woodard, James C. Gillespie, Dana E. Selley, William L. Dewey, Piyusha P. Pagare, Yan Zhang

**Affiliations:** a Department of Medicinal Chemistry, School of Pharmacy, 6889 Virginia Commonwealth University, 800 E Leigh Street, Richmond, Virginia 23298, United States; b Department of Pharmacology and Toxicology, 6889 Virginia Commonwealth University, 410 North 12th Street, Richmond, Virginia 23298, United States; c Center for Drug Discovery, 6889 Virginia Commonwealth University, 800 E Leigh Street, Richmond, Virginia 23298, United States; d Institute for Drug and Alcohol Studies, 6889 Virginia Commonwealth University, 203 East Cary Street, Richmond, Virginia 23298, United States

## Abstract

Opioid use disorder (OUD) affects 2.1 million people in the U.S., and current treatments have significant limitations. Therefore, there is a critical need for novel, selective, potent, and reversible mu opioid receptor (MOR) antagonists for OUD treatment. The “message-address” concept applied to the naltrexone skeleton keeps the epoxymorphinan core (message) consistent while modifying the C-6 substituent (address). This approach led to the development of 17-cyclopropylmethyl-3,14β-dihydroxy-4,5α-epoxy-6α-(indole-7-carboxamido)­morphinan (**NAN**). In this study, we have designed and evaluated **NAN** analogues to enhance their pharmacological properties by applying the “nitrogen-walk” concept, i.e., replacing each CH group with a nitrogen atom on the indole ring sequentially while exploring different attachment positions onto the azaindole ring. A total of 36 analogues were synthesized and characterized. Competitive binding assays and functional activity studies identified eight potential MOR antagonists, with compound **7** showing the highest potency in a mouse antinociception model and inducing fewer withdrawal symptoms than naloxone.

## Graphical abstract

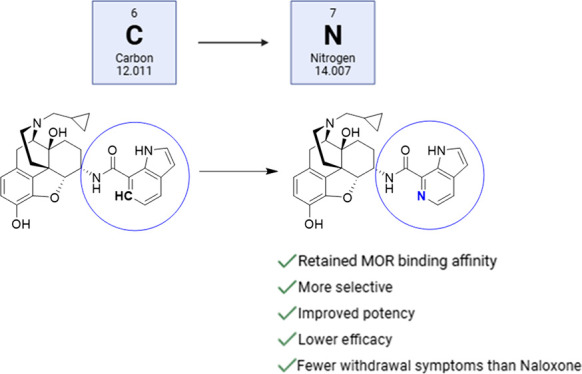

## Introduction

Opioid use disorder (OUD) is a prevalent substance use disorder affecting over 2.1 million individuals in the United States. Alarmingly, opioids have led to more deaths than any other drug, and mortality rates continue to rise.^1,2^ Between 1999 and 2018, opioid-related deaths increased by four times. Although the death rate stabilized from 2018 to 2019, the COVID-19 pandemic triggered a sharp rise in fatalities.^3,4^ From 2019 to 2020, opioid overdose deaths surged by 32%, driven by factors such as reduced access to interventions, heightened stress from job loss, worsening mental health due to isolation, and shifts in drug combinations or purity. This escalating opioid crisis poses a significant public health challenge. While current treatments for OUD typically combine medication with counseling, these options are limited and often come with undesirable side effects.^5–7^ Therefore, there is an urgent need for the development of innovative therapeutic compounds for individuals suffering from OUD.

The four main opioid receptors–mu (MOR), kappa (KOR), delta (DOR), and nociceptin/orphanin FQ (NOP)–play distinct roles in opioid signaling. Among these, extensive research has shown that MOR is primarily responsible for both the analgesic and addictive effects of opioids.^8–10^ Approved medications for OUD and opioid overdose include nalmefene, buprenorphine, naloxone, naltrexone, and methadone (Figure 1). However, these treatments are associated with several limitations, such as respiratory depression, nausea, vomiting, or a short duration of action. Despite these drawbacks, buprenorphine, naloxone, naltrexone, and nalmefene all feature a common epoxymorphinan scaffold, making it an ideal framework for designing new compounds for OUD.^11^ This scaffold is particularly promising due to its warranted high binding affinity to opioid receptors.

**Figure 1:**
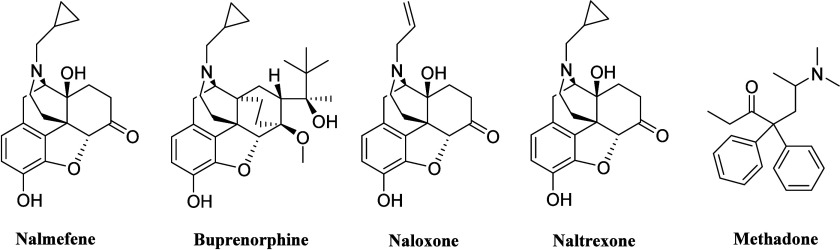
Chemical structures of the five FDA-approved drugs for OUD and opioid overdose.

Over the years, our lab has applied the ‘message-address’ concept where the epoxymorphinan scaffold represents the ‘message’, and the incorporation of various heteroaromatic ring systems at the C-6 position serves as the ‘address’. This led to several potential MOR antagonists and one of them being identified as 17-cyclopropylmethyl-3,14β-dihydroxy-4,5α-epoxy-6α-(indole-7-carboxamido)­morphinan, also known as **NAN**.^12^ Computational chemistry studies suggested that **NAN** may act as a bitopic ligand, i.e. its epoxymorphinan core (‘message’) binds to the MOR orthosteric site while the indole ring (‘address’) targets the MOR allosteric site. **NAN** demonstrated several favorable pharmacological characteristics, including high binding affinity to the MOR, reasonable selectivity to MOR over KOR and DOR, and absence of significant withdrawal symptoms in morphine-tolerant mice. Despite its favorable pharmacological properties, NAN’s suboptimal absorption, distribution, metabolism, excretion, and toxicity (ADMET) characteristics severely hindered its further development as a potential drug candidate.^13^

The nitrogen atom is a commonly employed element in drug design, largely due to its widespread presence in FDA-approved pharmaceuticals.^14,15^ As of 2020, over 75% of drugs approved by the US FDA contain heterocycles with nitrogen atoms.^16–18^ Substituting a CH group with a nitrogen atom has been found to improve various pharmacological properties, such as enhanced binding affinity, greater selectivity, better metabolic stability, and improved solubility. Its higher electron density, compared to that of a CH group, enables increased intermolecular and intramolecular interactions, which can strengthen the binding to biological targets. This not only results in possibly improved pharmacokinetic and pharmacodynamic properties but also leaves the molecular weight unchanged, ensuring that ligand efficiency remains consistent. Additionally, research indicates that the number and arrangement of nitrogen atoms within a molecule can affect these pharmacological characteristics. Figure 2 illustrates three FDA-approved drugs in which a CH group in an aromatic ring was replaced with a nitrogen atom.^19^ These examples include inhibitors or antagonists that became more effective after this modification. In each case, the introduction of nitrogen led to improvements in potency and/or selectivity. These findings demonstrate how a single atomic change can significantly enhance the therapeutic potential of a drug candidate, highlighting the importance of structure-based drug optimization in modern medicinal chemistry.

**Figure 2:**
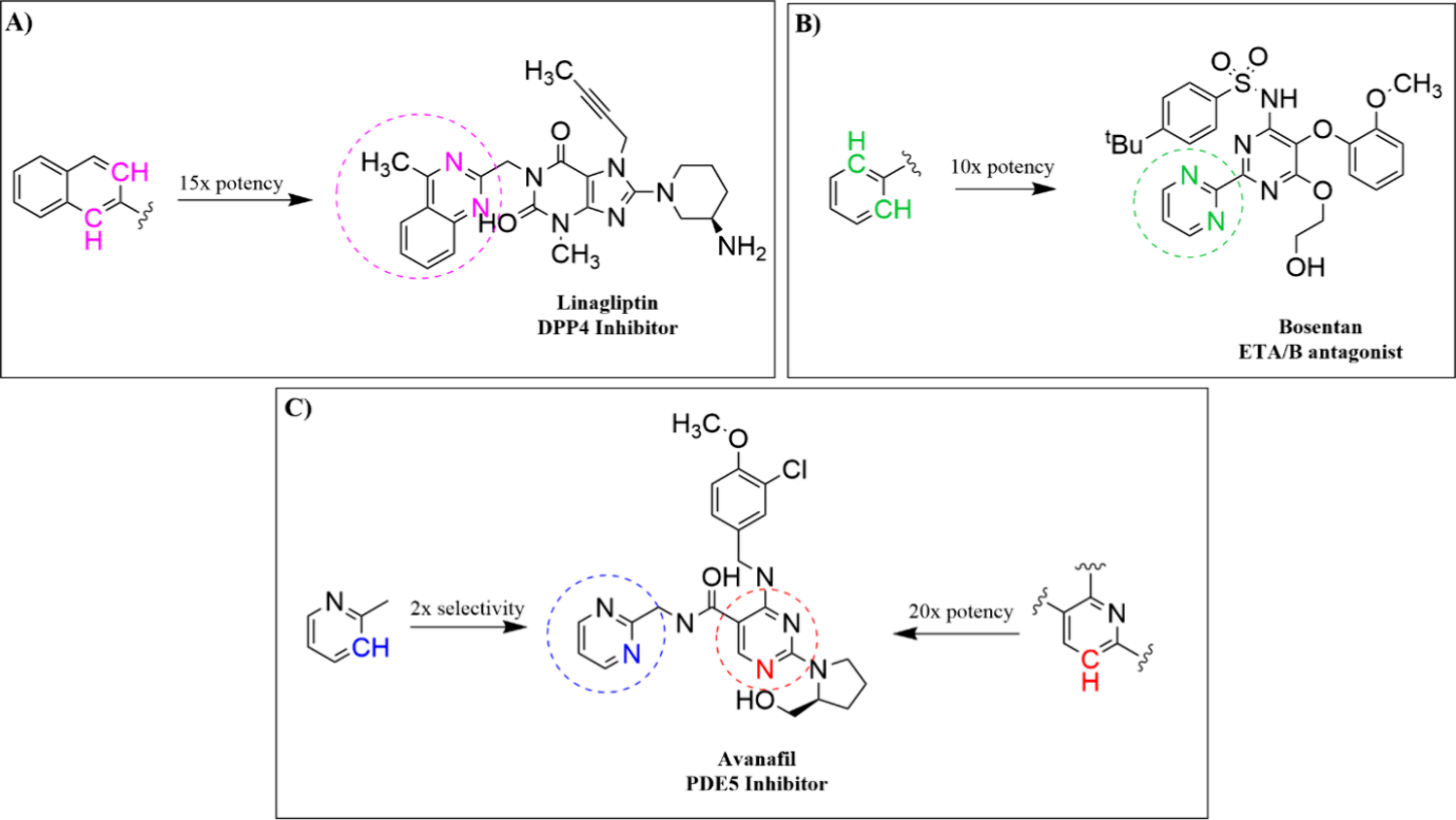
Nitrogen replacement of CH groups exemplified by three FDA-approved drugs: A) Linagliptin, B) Bosentan, and C) Avanafil.^19^

Building on the success of the aforementioned strategy, in the current work, we present our efforts in modifying the structure of NAN with the aim to enhance the MOR antagonism potency and function, as well as the drug-like properties. Such an effort will also expand our current small molecule library with more diversified structural features in order to strengthen the capacity of our drug discovery pipeline. In total, 36 novel compounds were designed, synthesized, and biologically characterized.

## Results and Discussion

### Molecular Design

We modified the structure of **NAN** by substituting a CH group in its indole side chain with an additional nitrogen atom. Moreover, this nitrogen atom was ‘walked’ around the indole ring while the effects of different substitution positions to the azaindole ring was also assessed. This resulted in a total of 36 novel derivatives as shown in Figure 3. The physicochemical properties of the 36 **NAN** derivatives were predicted using ACD/Percepta (v2020.2.0) (Table S2). All 36 compounds exhibited physiochemical properties similar to **NAN** and were within BBB permeability guidelines and Lipinski’s Rule of Five (e.g., MW < 500; HBD < 5; cLogP 2–5; p*K*_a_ 7.5–10.5).^20–22^

**Figure 3:**
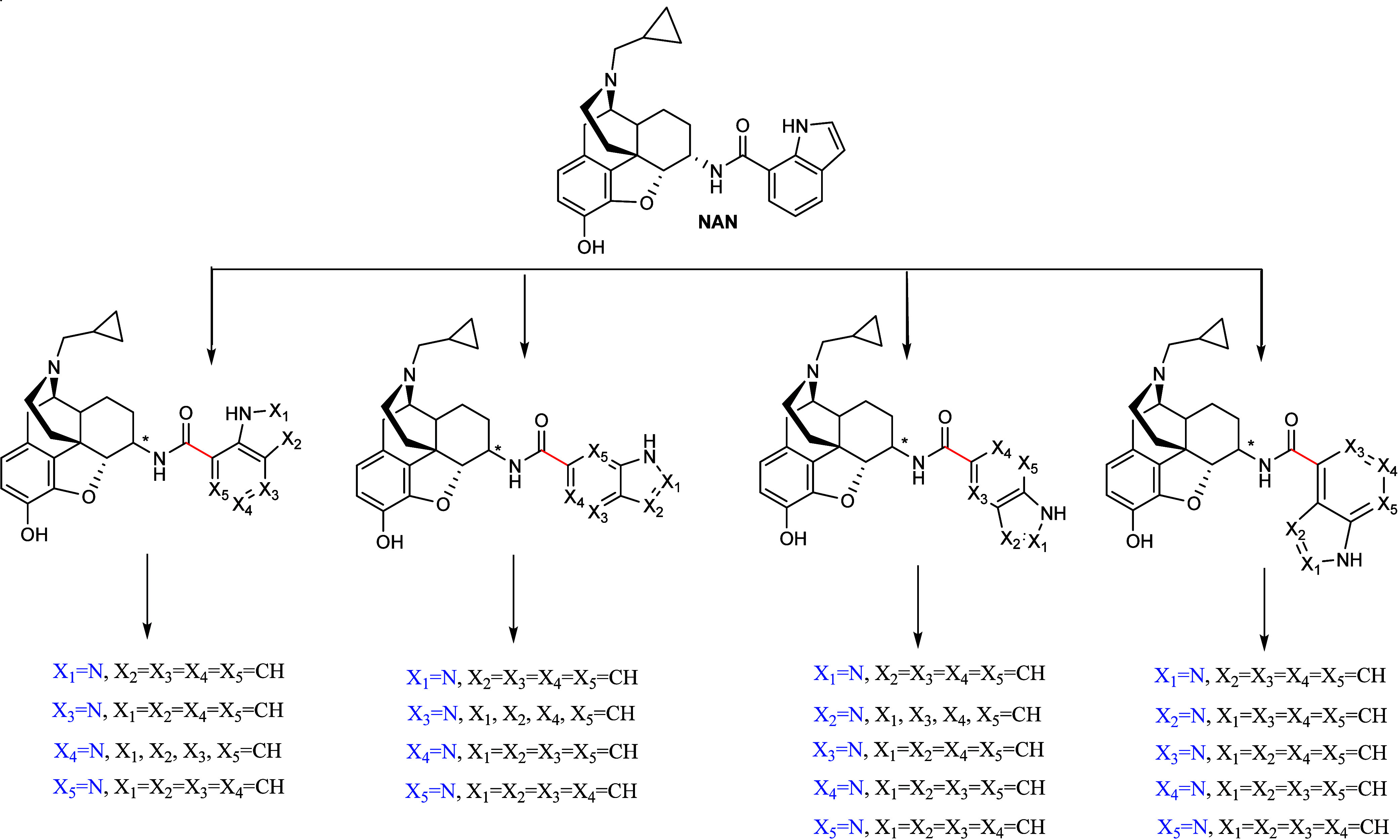
Structure of **NAN** and the 36 designed analogs which include introducing an additional nitrogen atom (blue) on the indole ring along with changing the location of the amide bond (red) to the indole ring.

### Chemical Synthesis

All 36 newly designed derivatives were synthesized based on established methods, using naltrexone (NTX) as the starting point.^11–24^ Reductive amination with benzylamine and sodium borohydride (NaBH_4_) produced the monobenzyl amine intermediate, while dibenzylamine (DBA) and sodium cyanoborohydride (NaCNBH_3_) generated the dibenzyl amine intermediate (Scheme 1). The intermediates were subjected to catalytic hydrogenation under acidic conditions, yielding the respective α- or β-naltrexamine salts. These intermediates were then coupled with the appropriate acid using EDCI, HOBt, and TEA under anhydrous conditions, followed by dealkylation with K_2_CO_3_. The resulting final compounds were converted to their hydrochloric acid salts, characterized by ^1^HNMR, ^13^CNMR and mass spectra, and were analyzed by HPLC to confirm >95% purity prior to subsequently employed in both in vitro and in vivo pharmacological studies.

**Scheme 1:**
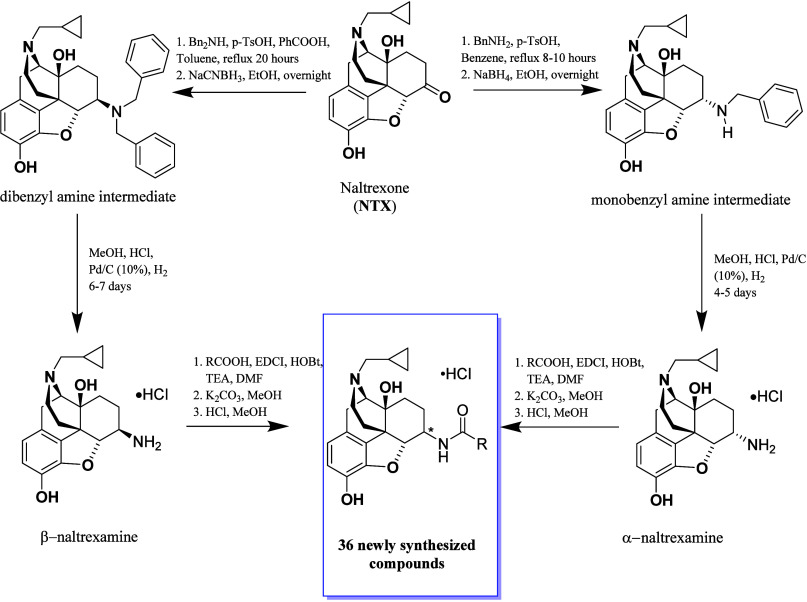
Synthetic Route for Nitrogen-Walk Derivatives

### In Vitro Pharmacological Studies

The affinity and selectivity profiles at MOR, KOR, and DOR were explored for all newly synthesized compounds using competitive radioligand binding assays. The [^35^S]-GTPγS binding assay was conducted at the MOR to assess each ligand’s agonist potency and efficacy, with efficacy measured relative to the full agonist DAMGO. In vitro data for the 6α-derivatives are presented in Table 1, the one for the 6β-derivatives are shown in Table 2.

**Table 1:**
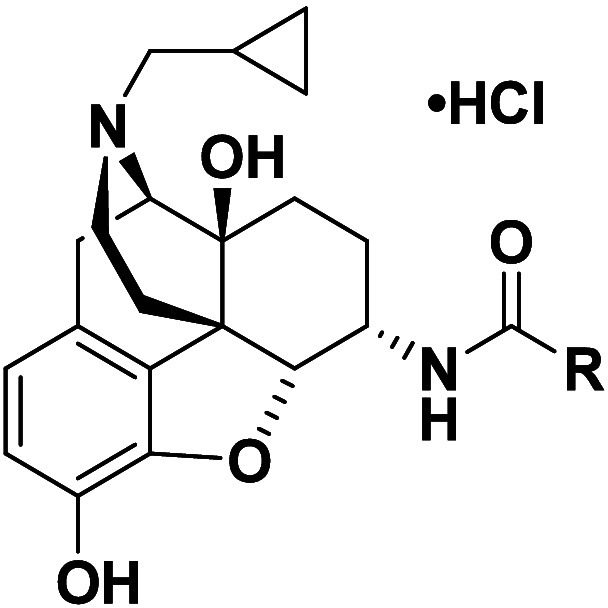

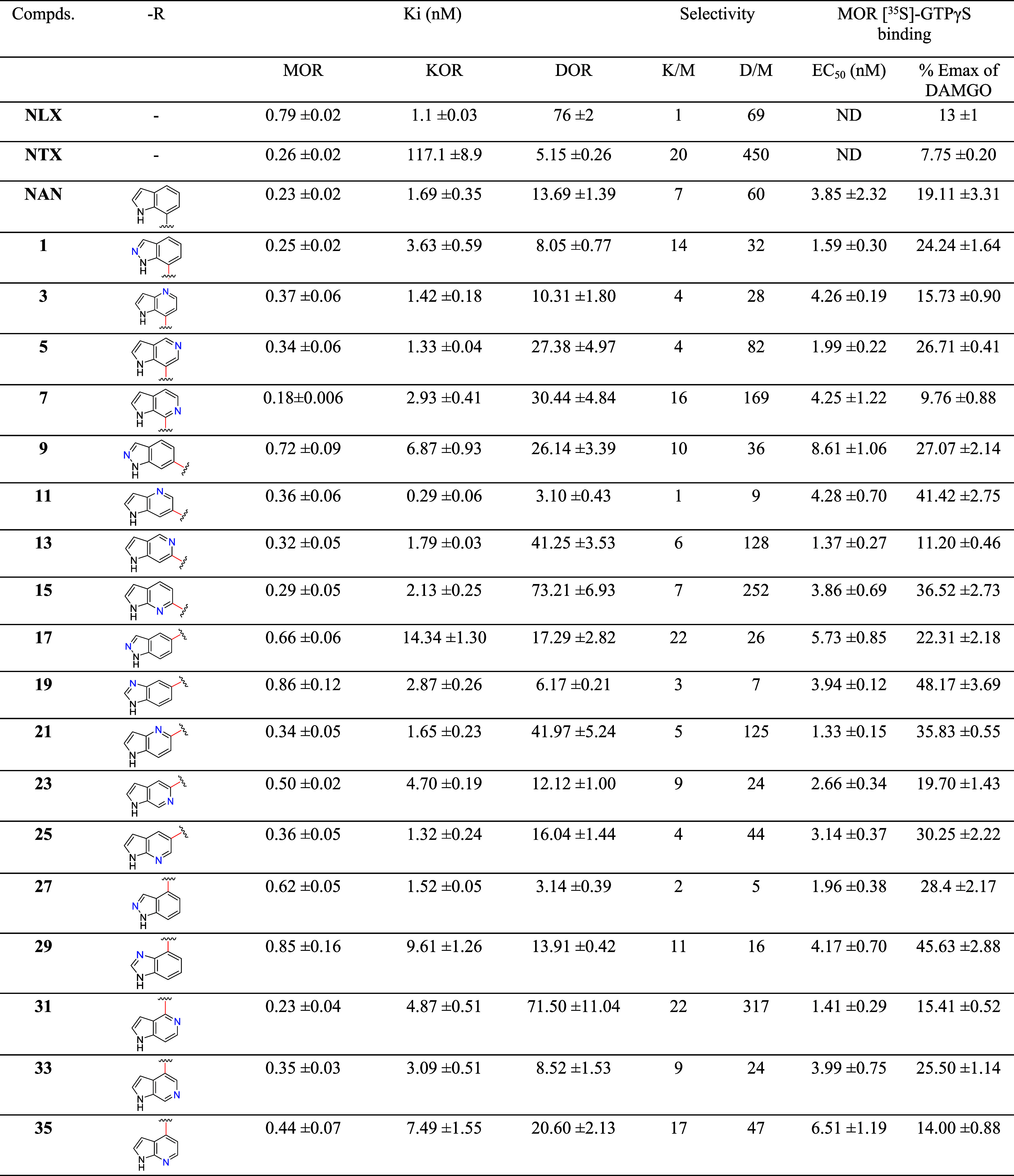
Binding Affinities at Opioid Receptors and MOR Functional Activity ([^35^S]-GTPγS) of 6α-Analogs^a^

a Values are presented as mean ± SEM (*n* ≥ 3).

**Table 2:**
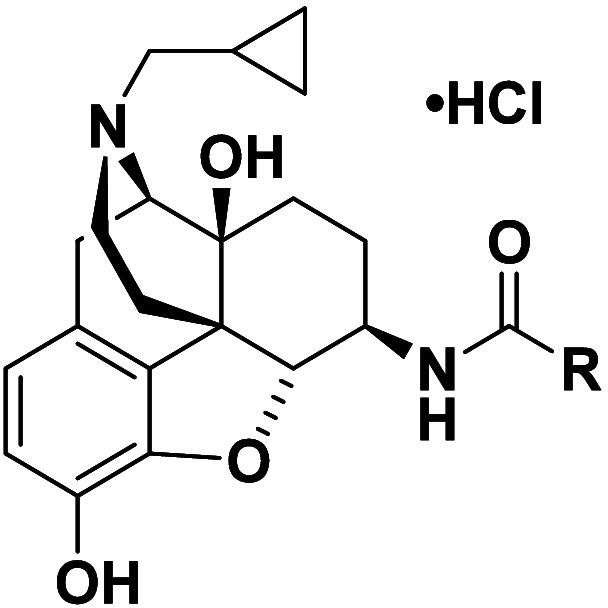

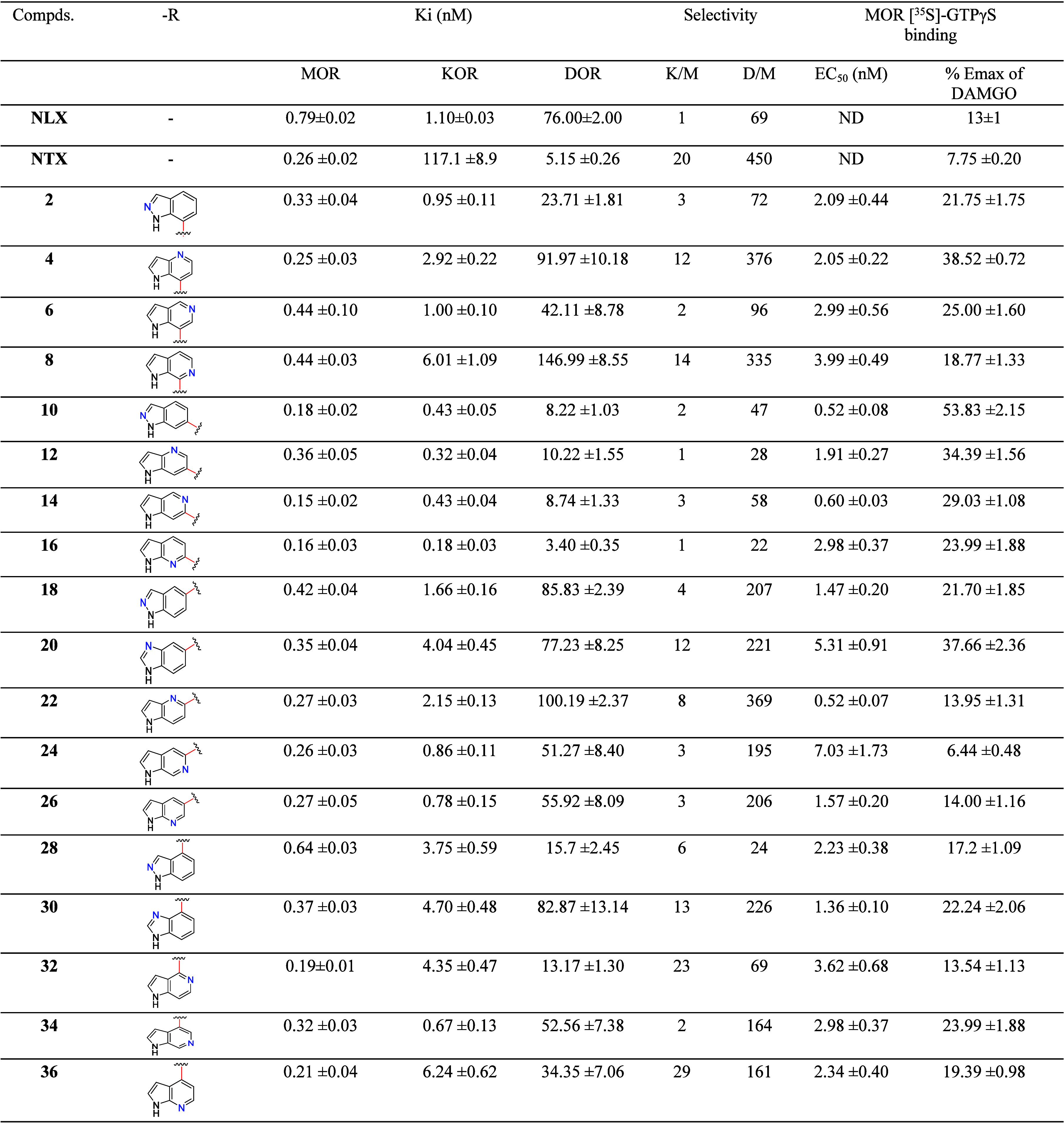
Binding Affinities at Opioid Receptors and MOR Functional Activity ([^35^S]-GTPγS) of 6β-Analogs^a^

a Values are presented as mean ± SEM (*n* ≥ 3).

All 36 epoxymorphinan derivatives (Tables 1 and 2) exhibited MOR binding affinities in the subnanomolar range, comparable to the parent compound **NAN**, indicating that they were equipotent in terms of receptor binding. This also indicates that introducing an additional nitrogen atom and/or altering the amide bond position did not significantly impact MOR binding. Similar to **NAN**, most compounds exhibited single-digit nanomolar binding affinities at the KOR, except for compounds **2**, **10**, **11**, **12**, **14**, **16**, **24**, **26**, and **34**. These nine compounds displayed subnanomolar binding at the KOR, with eight adopting the β configuration at C-6. Notably, compounds **11** and **12** share an additional nitrogen at position 4 of the indole ring and an amide bond at position 6, differing only in the C-6 stereochemistry. Additionally, DOR binding affinities varied without a discernible trend. Interestingly, 16 of the 36 compounds exhibited greater selectivity for the MOR over the KOR when compared with **NAN**, while 19 compounds showed greater selectivity for the MOR over the DOR. An improvement in MOR selectivity over KOR and DOR was observed for compounds **4**, **7**, **8**, **20**, **22**, **30**, **31**, **32**, and **36** when compared to NAN.

Subsequently, [^35^S]-GTPγS binding assays were employed to characterize the functional activity of all 36 compounds at the MOR. All synthesized compounds exhibited one-digit nanomolar potencies similar to **NAN** with only three compounds (**10**, **14,** and **22**) having subnanomolar potencies as shown in Tables 1 and 2. Notably, these three compounds showed a β configuration at the C6 position of the epoxymorphinan. All 36 derivatives showed efficacies ranging from 6.44 to 53.83% *E*_max_ of DAMGO. Based on their efficacy, most derivatives were identified as low-efficacy partial agonists. Interestingly, **7** and **24** exhibited *E*_max_ values of 9.76% and 6.44%, respectively, suggesting potential antagonists, comparable to the known MOR antagonist NTX (*E*_max_ 7.75% of DAMGO). The common feature between **7** and **24** is the presence of the additional nitrogen at position 6 on the indole ring. Both compounds (**7** and **24**) showed similar potency as the parent compounds **NAN** but showed lower efficacy than **NAN** (*E*_max_ 19.11% of DAMGO).

Overall, although there did not appear to be a clear trend with the introduction of the additional nitrogen or the position of the amide bond around the indole ring, the new analogs maintained binding affinity and selectivity at the MOR compared to **NAN**. Additionally, most of the compounds exhibited similar potency as the parent compound at the MOR, with a few showing a even lower efficacy than **NAN.**

### In Vivo Warm-Water Tail Immersion Assay

Using the warm-water tail immersion assay, the synthesized compounds were evaluated for antinociceptive activity and for antagonism of morphine’s antinociceptive effects, when applicable. In this test, a mouse’s tail is immersed in warm water, and the duration of tail immersion is recorded. A longer immersion time corresponds to a higher Maximum Possible Effect (MPE) and indicates stronger antinociceptive effects of the compound.

All newly synthesized compounds were first tested at a single dose to exclude any potential agonists from further studies. The test compound at a dose of 10 mg/kg was administered subcutaneously, and their tail withdrawal latency was measured 20 min later. As shown in Figure 4, all compounds showed significantly lower MPE compared to morphine. Compounds **8** (35.7% MPE) **16** (42.0% MPE), and **36** (31.7% MPE) exhibited the strongest antinociceptive effects at 10 mg/kg, suggesting they might behave as low efficacy partial opioid agonists. Interestingly, these three compounds are all in the beta confirmation at C6.

**Figure 4:**
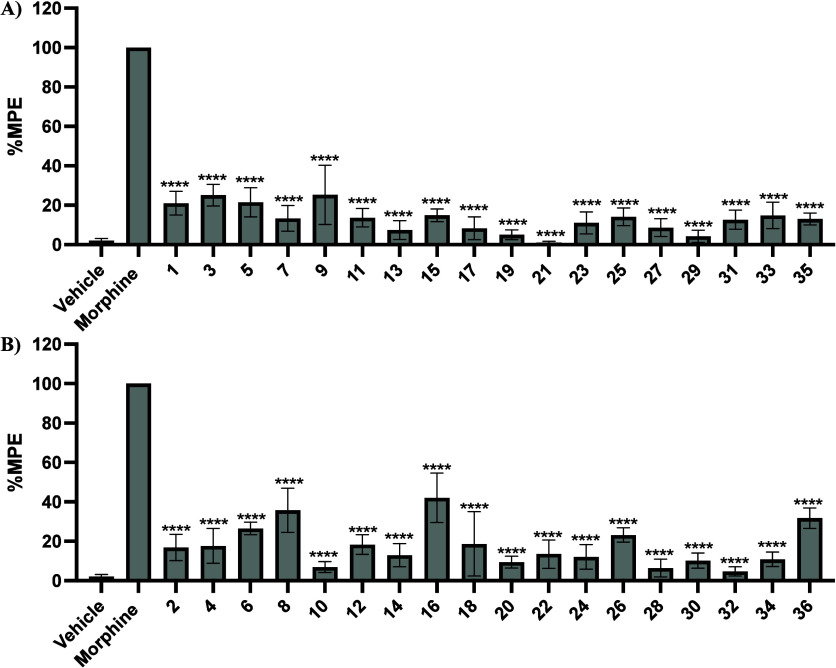
A) 6α-derivatives and B) 6β-derivatives in the warm-water tail immersion assay results of nitrogen-walk analogs (*n* = 6) as agonists at a single dose of 10 mg/kg s.c. Saline and morphine were used as the negative and positive controls, respectively. Data are presented as mean values ± SD *P < 0.05, **P < 0.01, and ***P < 0.0005, ****P < 0.0001, compared to 10 mg/kg morphine (s.c.).

Following this, the compounds were then tested at a fixed dose of 10 mg/kg to assess their ability to antagonize the antinociception of morphine (10 mg/kg). As illustrated in Figure 5, compounds **10, 12, 18, 19, 27, 28, 30,** and **35** did not antagonize morphine’s antinociception. Among the 36 derivatives tested, 12 compounds (**3, 10, 12, 17, 18, 19, 27, 28, 30, 34, 35,** and **36**) demonstrated an MPE greater than 50%, while the remaining 24 compounds showed an MPE below this threshold. Out of the 24 compounds, 8 compounds (**1, 4, 6, 7, 14, 15, 21,** and **31**) exhibited an MPE of less than 25%, indicating their potential to antagonize the antinociceptive effects of morphine most effectively. Structurally, except compound **1**, these compounds show the presence of the additional nitrogen on the six-membered benzene ring of the indole as opposed to the five-membered pyrrole ring. Although the nitrogen atoms were repositioned within the same heteroaromatic scaffold, these subtle changes can markedly affect physicochemical properties, membrane permeability, metabolic stability, and transporter interactions. This helps explain why compounds with similar in vitro binding affinity exhibit distinct pharmacokinetic and in vivo pharmacodynamic profiles.

**Figure 5:**
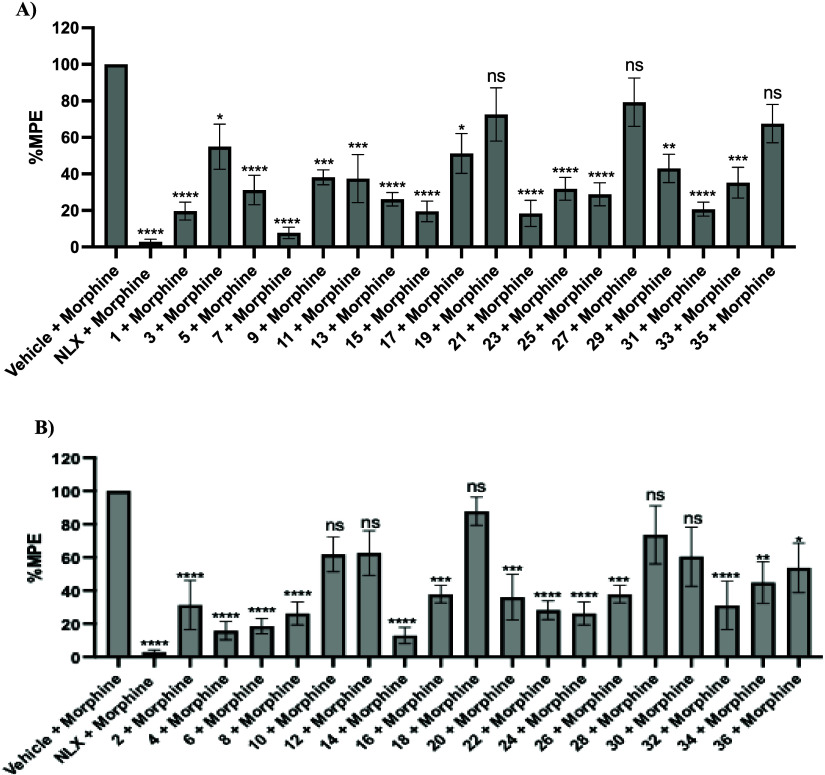
A) 6α-derivatives and B) 6β-derivatives in the warm-water tail immersion assay results of nitrogen-walk analogs (*n* = 6) as antagonists at a single dose of 10 mg/kg s.c. Saline and morphine were used as the negative and positive controls, respectively. Data are presented as mean values ± SD *P < 0.05, **P < 0.01, and ***P < 0.0005, ****P < 0.0001, compared to 10 mg/kg morphine (s.c.).

The eight compounds identified in the single-dose study were subsequently advanced to in vivo dose–response evaluation (Figure 6). The potencies of these compounds were assessed as shown by their AD_50_ values (the dose of a compound that antagonizes 50% of morphine’s antinociceptive effect), which ranged from 0.09 to 5.07 mg/kg. Seven of the eight compounds displayed AD_50_ values similar to that of **NAN**. Compound **7** demonstrated the highest potency, with an AD_50_ value of 0.09 mg/kg, significantly surpassing the parent compound **NAN** and comparable to **NLX** (Table 3). This data also aligns with its in vitro functional data shown in Table 1 where compound **7** showed lower efficacy (% *E*_max_ 9.76% of DAMGO) compared to **NAN** is (% *E*_max_ 19.11% of DAMGO).

**Figure 6:**
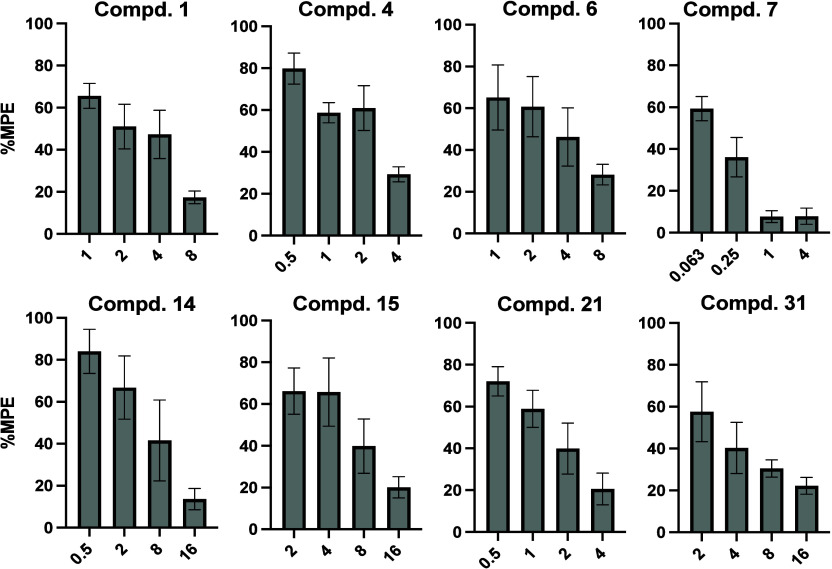
Dose response study of the most potent NAN analogs (*n* = 6) as antagonists in the presence of morphine (10 mg/kg) in the warm-water tail immersion assays along with the corresponding AD_50_ values. Doses are in mg/kg and data are presented as mean values ± SD.

**Table 3:** AD_50_ (mg/kg) (95% CL) Values of the Identified Potential Antagonists Compared to NLX and NAN

Compound	AD_50_ (mg/kg) (95% CL)
NLX	0.05 (0.03–0.09)
NAN	2.07 (0.40–10.74)
1	2.29 (1.39–3.75)
4	2.11 (1.15–3.85)
6	2.79 (1.96–3.97)
7	0.09 (0.06–0.14)
14	3.00 (1.76–5.15)
15	5.07 (3.67–7.00)
21	1.33 (1.07–1.64)
31	4.24 (2.39–7.52)

### In Vivo Opioid Withdrawal Studies

Naloxone is an opioid antagonist commonly used in emergencies to reverse opioid overdose. However, opioid neutral antagonists like **NLX** are often associated with undesirable withdrawal effects.^25^ Opioid withdrawal effects are often viewed negatively because they can trigger relapse, making it more difficult to break free from addiction. These effects can cause significant physical and mental distress, as well as lead to long-term health problems like dehydration and malnutrition. It is crucial to evaluate withdrawal symptoms early in the research process to help improve treatment design and develop safer, more tolerable medications. Hence, compound **7** was assessed for its ability to precipitate withdrawal effects in morphine-tolerant mice and evaluate its potential advantages over **NLX**. The withdrawal behaviors in morphine-pelleted mice, including wet dog shakes, jumps, and paw tremors, were scored for 20 min beginning 3 min after compound administration. Previous studies have reported that naltrexone induces withdrawal effects at a dose of 1 mg/kg, a finding that is consistent with our study,^13,26^ which is shown in Figure 7. When compared to **NLX** at a dose of 1 mg/kg, compound **7** results in significantly less wet dog shakes and paw tremors. More interestingly, even at a higher dose of 5 mg/kg, compound **7** induces significantly fewer wet dog shakes and paw tremors. One of the key advantages of compound **7** is that it exhibits the comparable potency as **NLX** (Table 3), yet has significantly fewer withdrawal effects, making it a potentially promising alternative for opioid use disorder.

**Figure 7:**
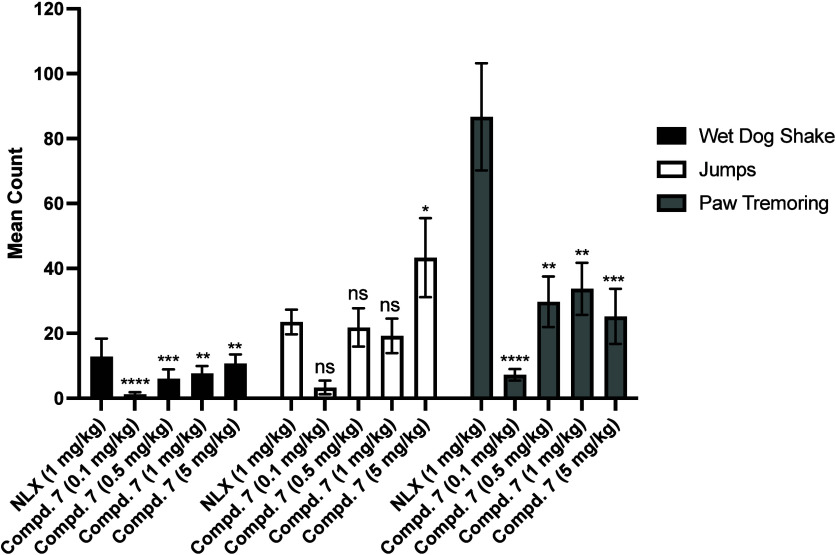
*In Vivo* withdrawal assays of compound **7** in morphine-pelleted mice (*n* = 6), including wet dog shakes, jumps and paw tremors. All doses of compound **7** were administered s.c. *P < 0.05, **P < 0.01, and ***P < 0.0005, ****P < 0.0001, compared to 1 mg/kg naloxone (NLX; s.c.).

### Calcium Flux Assay

To further validate the functional activity of compound **7** at the MOR, a calcium mobilization assay was performed with MOR-CHO cells. Activation of the G_q_ signaling pathway, a downstream component of G-protein coupled receptor signaling, leads to an increase in intracellular calcium levels, which can be quantitatively measured.^27,28^ In contrast, inhibition of the G_q_ pathway is assessed indirectly by monitoring the reduction in calcium release induced by a known agonist (e.g., DAMGO). This assay thus provides a sensitive measure of both agonist and antagonist activity at the receptor. The assay followed established protocols from previously reported literature.^12^ As shown in Figure 8, the results demonstrated that as expected, compound **7** exhibited no agonistic activity at the MOR. However, it showed dose-dependent antagonism of DAMGO-induced calcium flux. Notably, compound **7** demonstrated equipotency to the MOR antagonist naltrexone, with an IC_50_ value of 28.37 ± 12.57 nM, compared to the reported IC_50_ value of 15.59 ± 1.96 nM for naltrexone.^26^ Notably, compound **7** was more potent than **NAN**, which has a previously reported IC_50_ value of 50.29 ± 1.62 nM.^12^

**Figure 8:**
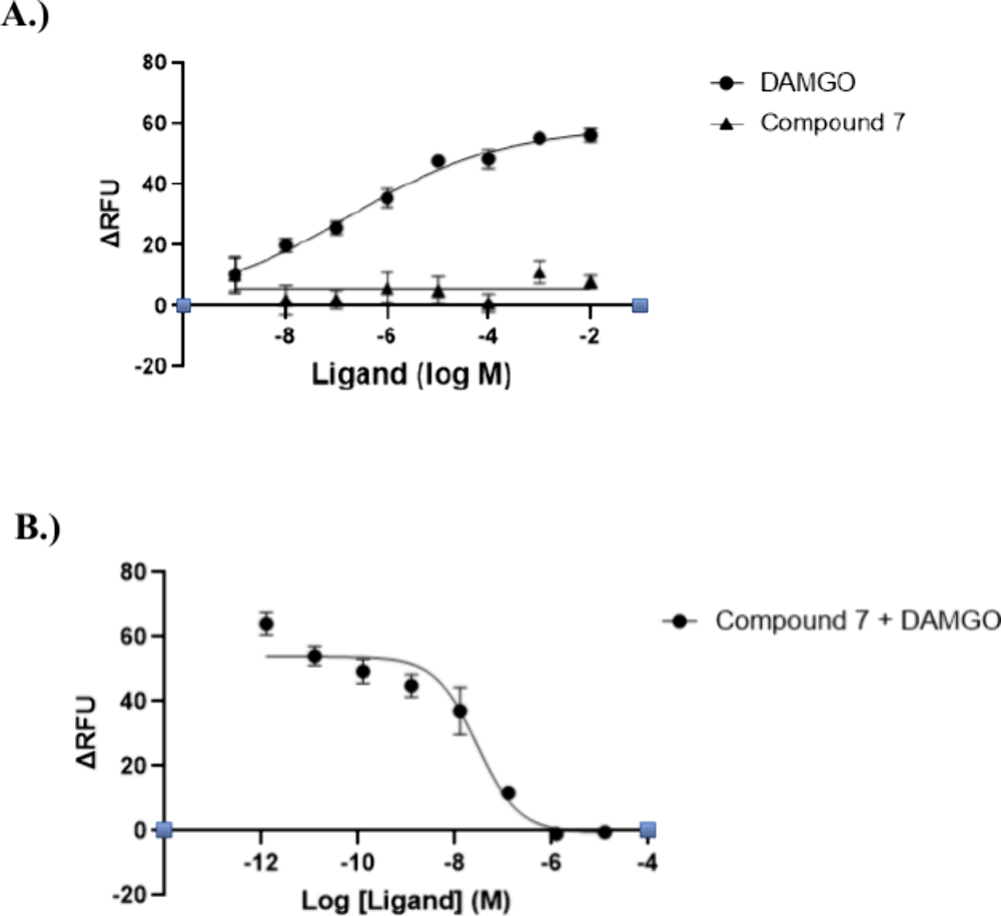
Calcium flux assay of compound 7 in Gα_qi4_-transfected MOR-CHO cells. (A) Compound **7**, exhibited no apparent agonism. DAMGO was used as a control. (B) Compound **7** significantly antagonized the DAMGO-induced intracellular calcium increase. The assay was repeated at least three times, and the data is shown as the mean ± SEM. The IC_50_ value for compound **7** was 28.37 ± 12.57 nM.

### Molecular Modeling Studies

While compound **7** exhibited binding affinity to the MOR comparable to **NAN**, it demonstrated lower efficacy in GTPγS assays compared to **NAN**. To understand the molecular basis for this functional activity, we conducted molecular modeling studies on how the structural modification in compound **7** may influence its interactions with the MOR. Compound **7** was first docked into the inactive MOR receptor (**PDB ID: 4DKL**).^29^ The top-scoring pose, as determined by the CHEM-PLP scoring function, was selected as the binding conformation for molecular dynamics (MD) simulations (Figure 9A).

**Figure 9:**
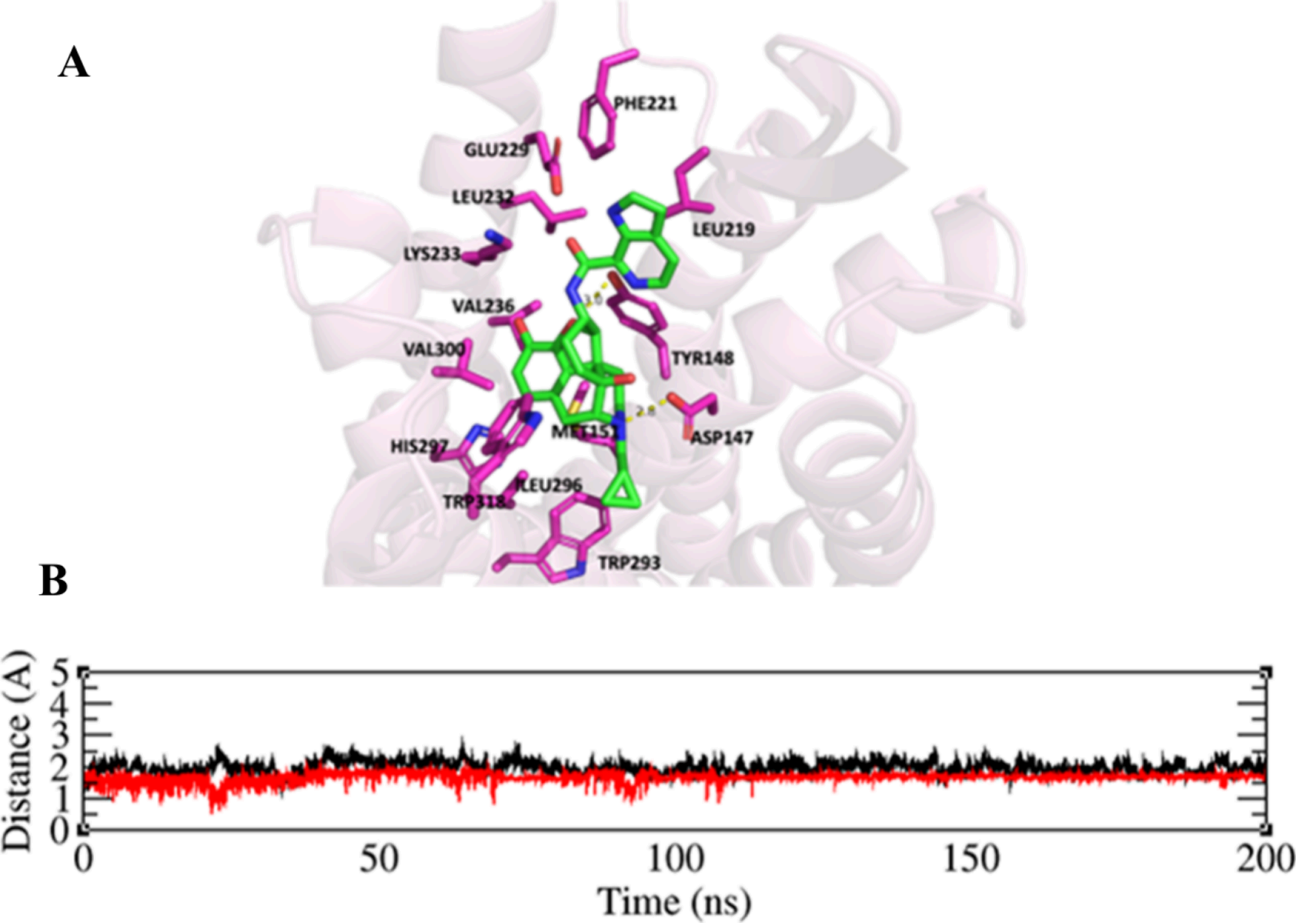
A. Binding mode of compound **7** in the inactive MOR with key residues in the binding pocket after 200 ns MD simulation; (PDB 4DKL).^27^ The MOR is shown as light pink cartoons. Compound **7** and key amino acid residues are shown on the sticks. Carbon atoms: Compound **7** (green); key amino acid residues (Magenta); oxygen atoms (red); nitrogen atoms (blue). B. Root Mean Square deviation (RMSD) for Compound **7** (red), and the backbones of the inactive MOR (black).

Prior to the MD simulations, the ligand–receptor complex was inserted into a membrane system using 1-palmitoyl-2-oleoylphosphatidylcholine (POPC) homogeneous lipid (approximately 80 POPC molecules at the upper and lower leaflets, respectively). In addition, sodium and chloride ions were added using the Monte Carlo ion replacement method to make the concentration of NaCl approximately 0.15 M. The system was then solved with the TIP3P water model in a rectangular box. This system, including the ligand–receptor complex, POPC lipid membrane, ions and TIP3P water molecules was generated as the starting structure to conduct the following MD simulations. The average system size was approximately 80,000 atoms. (Figure S1B)

Upon building the membrane-aqueous system, MD simulations were conducted for 200 ns using Amber 2020 software package. The root-mean-square deviation (RMSD) values for both the ligand and all protein backbone atoms were calculated relative to their initial structures. The analysis of the resulting data, as shown in Figure 9B, revealed that the system reached equilibrium after about 120 ns. During the 200 ns MD simulation, RMSD values for the ligand and protein backbone stayed consistently under 3 Å, reflecting a stable complex.^30,31^

Docking and MD simulations (Figure S2A) showed that the epoxymorphinan portion of compound **7** occupied the ″message″ domain of MOR, forming contacts with ASP147, TYR148, MET151, ILE296, HIS297, TRP293, and TRP318, consistent to other epoxymorphinan ligands. The quaternary ammonium nitrogen established an ionic interaction with ASP147, while the dihydrofuran oxygen engaged TYR148 through hydrophobic interactions.^32–35^ The cyclohexyl ring adopted a twisted-chair conformation, maintaining the α-configuration of the amide side chain, consistent with **NAN** binding.^12^ (Figure 9A)

The azaindole moiety of compound **7** underwent notable conformational changes after 200 ns of molecular dynamics (MD) simulation, resulting in a binding mode distinct from that observed in the initial molecular docking study. Specifically, the reorientation of the azaindole moiety strengthened contacts with critical residues in TM4 and TM5, thereby reinforcing the inactive state of MOR (Table S3). These residues include L219, E229, and K233. (Figure S2A). These binding characteristics were similar to those seen with the **NAN** ligand at the inactive MOR (Figure S2B),^12,35^ which may explain the high binding affinities and the low efficacy agonistic effects exhibited by both **NAN** and compound **7** at the MOR.

The azaindole ring of compound **7** slightly shifts away from the hydrophobic residues LEU232 and PHE221 to minimize unfavorable steric interactions with these residues (Figure 9A). However, the introduction of an additional nitrogen atom at the 2-position of the indole moiety in **NAN** could allow for the formation of a hydrogen bond with GLU229 for compound **7**.^12^ Previous studies have shown that GLU229 may play a role in stabilizing the inactive conformation of MOR.^36–38^ This difference may account for the lower efficacy of compound **7** relative to **NAN** in the GTPγS functional assay.

### In Vitro Metabolic Stability

Hepatic metabolic stability is a key consideration in early drug discovery, particularly for orally administered drugs. It is known that orally administered drugs undergo first-pass metabolism in the liver, primarily in hepatocytes, before reaching the target organs.^39^ To assess this, compound **7** and control drugs were tested using liver S9 fractions containing phase 1 and phase 2 metabolic enzymes from both human and rat sources. The half-life for compound **7** in human liver S9 fraction was >120 min, whereas in rat liver S9 fraction was 21.1 min (Table 4). This compound shows promise for oral dosing; however, a more comprehensive in vivo pharmacokinetic analysis is required for further evaluation.

**Table 4:** In Vitro Metabolism Study of Compound 7 and Control Compounds^a^

Compound	*T*_1/2_ human (min)	Cl_int_ human (μL/min/mg)	*T*_1/2_ rat (min)	Cl_int_ rat (μL/min/mg)
**7**	>120	<5.8	21.1	33.0
**Clozapine**	>120	<5.8	34.0	20.4
**Diclofenac**	18.5	37.4	101.8	6.8
**Imipramine**	102.7	22.8	20.3	113.7
**Propranolol**	>120	<19.3	14.1	164.2
**Terfenadine**	13.8	167.5	30.6	75.6

a Values represent the mean of two independent experiments using human and Sprague–Dawley rat liver S9 fractions.

### BBB-Penetration Studies

A recent article by Webborn et al., titled *″Free Drug Concepts: A Lingering Problem in Drug Discovery,″* emphasizes the importance of comparing concentrations of drug in plasma and brain as a critical parameter in CNS drug development.^40^ This concept underlies the rationale for conducting the CNS permeability study on compound **7**. Swiss Webster mice (n = 3 per time point) were administered compound **7** (10 mg/kg, s.c.) according to a previously reported protocol.^41,42^ At 5-, 10-, 30-, and 60 min postdose, brains and blood were harvested. Brains were rinsed with saline to remove residual blood and placed in 300 μL saline. Blood was centrifuged at 15,000 × g for 10 min at 4 °C to isolate plasma. Samples were stored at −80 °C until analysis.

As shown in Table 5, compound **7** reached a plasma concentration of 0.54 μg/mL at 5 min, peaking at 0.84 μg/mL at 10 min, followed by a decline to 0.67 μg/mL at 30 min and 0.38 μg/mL at 60 min. Compound **7** exhibited a gradual increase in brain concentration over time, reaching a peak of 0.18 μg/mL at the 60 min time point. The observed pharmacokinetic profile of compound **7**, characterized by a gradual increase in brain concentration over 60 min alongside a decline in plasma levels, suggests a time-dependent redistribution of the compound from systemic circulation into the brain.^43^ This trend is indicative of compound **7**’s ability to cross the blood-brain barrier (BBB), albeit at a moderate rate, and may reflect a delayed but sustained accumulation in brain tissue. Such a profile could be advantageous for CNS-targeted compounds, as it may support prolonged target engagement and therapeutic activity within the central nervous system despite declining systemic levels. These findings also suggest a potentially favorable brain-to-plasma ratio over time, which is often used as a surrogate measure of brain penetration and CNS exposure, warranting further investigation into compound **7**’s CNS pharmacokinetics and pharmacodynamic effects.

**Table 5:** Time Course of Blood–Brain Barrier Penetration for Compound **7** (10 mg/kg, s.c.) in Mice (n = 3, mean ± SD)

Time (min)	5	10	30	60
Brain (μg/g)	0.15 ± 0.08	0.17 ± 0.03	0.16 ± 0.02	0.18 ± 0.1
Plasma (μg/mL)	0.54 ± 0.08	0.84 ± 0.04	0.67 ± 0.06	0.38 ± 0.08
Brain-to-plasma ratio	0.29	0.20	0.24	0.46

### In Vitro Absorption Studies

Compound **7** was evaluated in Caco-2 cells to assess potential transporter-mediated efflux, which can impact oral absorption.^44^ Under the conditions with no inhibitor, **7** exhibited a minimal efflux ratio of 1.08, indicating that apical to basolateral and basolateral to apical transport were nearly equal and suggesting reasonable passive intestinal permeability. In the presence of the P-gp inhibitor verapamil, the efflux ratio decreased to 0.44, and with the BCRP inhibitor KO143 it decreased to 0.39, demonstrating that **7** may act as a substrate for both P-gp and BCRP. Overall, these results indicate that although **7** can interact with efflux transporters, its baseline efflux is minimal, and transporter-mediated effects may moderately limit its oral absorption (Table 6).

**Table 6:** In Vitro Absorption of Compound **7** and Reference Compounds in Caco-2 Cells

Compd.	Substrate	Efflux ratio	Efflux ratio + inhibitor
**7**	P-gp	1.08	0.44
**7**	BCRP	1.08	0.39
**Colchicine**	P-gp	25.0	5.0
**Estrone sulfate**	BCRP	33.5	1.7

### Plasma Protein Binding

After a drug reaches the systemic circulation, it can reversibly bind to plasma proteins, most commonly albumin and α_1_-acid glycoprotein.^45^ However, only the unbound, or “free,” drug is pharmacologically active, making it important to assess plasma protein binding. The extent of binding determines the fraction of free drug, which in turn influences distribution, efficacy, and elimination. Compound **7** demonstrated high plasma protein binding in both human (87%) and mouse (77%) plasma (Table 7), indicating that a substantial portion of the compound is retained in circulation. This property can be advantageous for maintaining stable plasma concentrations and a prolonged duration of action. For comparison, warfarin, a well-characterized highly protein-bound drug, acts as a systemic reservoir, which helps prolong its half-life (20–60 h) and allows for convenient once-daily dosing.^46^

**Table 7:** Plasma Protein Binding of Compound 7, Acebutolol, and Warfarin

compound	% Protein Bound human	% Protein Bound mouse, CD-1
**7**	86.98	77.31
**Acebutolol**	14.43	4.55
**Warfarin**	98.01	87.51

## Conclusion

Extensive literature supports the incorporation of an additional nitrogen into an aromatic ring system as a strategy to enhance drug-like properties. This approach is widely used in medicinal chemistry, as evidenced by the fact that approximately 75% of FDA-approved drugs contain nitrogen atoms. In our study, we applied this ‘nitrogen walk’ strategy by introducing an extra nitrogen into the ‘address’ portion of the C6 epoxymorphinan skeleton to improve upon the parent compound, **NAN**. Total 36 novel opioid ligands were synthesized and studied in various in vitro assays, including radioligand binding, GTPγS, and calcium flux. Additionally, in vivo assays, such as warm water tail immersion for agonism and antagonism, led to the identification of 8 potential opioid receptor antagonists. These antagonists were further tested in dose–response studies in mice, with compound **7** emerging as the most potent. Notably, compound **7** demonstrated similar potency to the FDA-approved naloxone, but exhibited significantly fewer withdrawal symptoms, offering a key advantage. Overall, the introduction of an additional nitrogen atom by application of the “Nitrogen-Walk” concept in the C6-side chain of epoxymorphinan skeleton showed benefits in enhancing the pharmacological profiles of the newly prepared opioid ligands and helped identify novel hits for future development of potential therapeutics for OUD.

## Experimental Section

### Chemistry

All nonaqueous reactions were performed under a predried nitrogen atmosphere. Solvents and reagents were obtained from Sigma-Aldrich, Alfa Aesar, and Fisher Scientific and used as received without further purification. Analytical thin-layer chromatography (TLC) was performed on Analtech Uniplate F254 plates, and flash column chromatography (FCC) was carried out on silica gel (230–400 mesh, Merck). ^1^H (400 MHz) and ^13^C (100 MHz) NMR spectra were recorded on a Bruker Ultrashield 400 Plus spectrometer; chemical shifts are reported in ppm. High-resolution mass spectra (HRMS) were acquired on an Applied BioSystems 3200 Q Trap with a Turbo V source (TurbolonSpray). Analytical reversed-phase HPLC was conducted on a Waters Arc system using an XBridge C_18_ column (3.5 μm, 4.6 × 50 mm) at ambient temperature and a flow rate of 0.8 mL/min. The mobile phase employed a linear gradient (10–15 min) from 90% 0.1% trifluoroacetic acid (TFA) in water/10% acetonitrile to 10% 0.1% TFA in water/90% acetonitrile. UV detection was set at 254 nm. Compound purities were calculated from peak area, and retention times (Rt) are reported in minutes. ALL COMPOUNDS ARE > 95% PURE BY HPLC ANALYSIS.

### General Procedure for the Amide Coupling/Hydrolysis Reaction

A solution of the carboxylic acid (2.5 equiv) in dry DMF (1.5 mL) was combined with hydroxybenzotriazole (HOBt, 3 equiv), N-(3-(dimethylamino)­propyl)-N′-ethylcarbodiimide (EDCI, 3 equiv), 4 Å molecular sieves, and triethylamine (5 equiv) in an ice–water bath. After stirring for 1 h, a solution of 6α- or 6β-naltrexamine (1 equiv) in predried DMF (1.5 mL) was added dropwise, and the mixture was stirred at room temperature. Upon completion as indicated by TLC, the reaction mixture was filtered through Celite, concentrated under reduced pressure, and the residue dissolved in anhydrous methanol (3 mL). Potassium carbonate (2.5 equiv) was then added, and the mixture was stirred overnight at room temperature, followed by filtration over Celite. The filtrate was concentrated, and the residue was purified by flash column chromatography using CH_2_Cl_2_/MeOH (1% NH_3_·H_2_O) to afford the free base. After confirmation of the structure by ^1^H NMR, the free base was converted to the hydrochloride salt and fully characterized by ^1^H NMR, ^13^C NMR, HRMS, and HPLC.

#### 17-Cyclopropylmethyl-3,14β-dihydro-4,5α-epoxy-6α-[1H-indazole-7-carboxamide]­morphinan Hydrochloride (1)

Compound **1** was synthesized as shown in the general procedure with 62% yield. ^1^H NMR (400 MHz, DMSO-*d*_6_) δ: 13.04 (s, 1H), 8.90 (s, 1H), 8.36 (s, 1H), 8.19 (s, 1H), 7.99 (t, *J* = 7.0 Hz, 2H), 7.24 (t, *J* = 7.4 Hz, 1H), 6.73 (d, *J* = 8.1 Hz, 1H), 6.58 (d, *J* = 8.1 Hz, 1H), 6.38 (s, 1H), 4.86 (d, *J* = 3.8 Hz, 1H), 4.71 (m, 1H), 3.96 (d, *J* = 6.8 Hz, 1H), 3.35 (m, 1H), 3.29 (m, 1H), 3.10 (m, 1H), 3.05 (m, 1H), 2.97 (m, 1H), 2.74 (m, 1H), 2.54 (m, 1H), 1.97 (m, 1H), 1.67 (m, 1H), 1.58 (m, 1H), 1.49 (m, 1H), 1.22 (m, 1H), 1.08 (m, 1H), 0.70 (m, 1H), 0.62 (m, 1H), 0.50 (m, 1H), 0.41 (m, 1H). ^13^C NMR (100 MHz, DMSO-*d*_6_) δ: 165.45, 146.34, 146.08, 138.84, 128.72, 125.50, 125.46, 124.53, 124.52, 124.14, 122.11, 119.66, 119.18, 118.28, 87.25, 69.44, 61.07, 57.06, 45.90, 45.28, 30.30, 29.27, 23.53, 19.38, 5.71, 5.20, 2.59. HRMS *m*/*z*: calc. 487.2345 for C_28_H_31_N_4_O_4_ [M + H]^+^; obs.: 487.2344 [M + H]^+^. The purity of the compound was checked by HPLC (Rt= 2.518 min) and was found to be 99.33% pure.

#### 17-Cyclopropylmethyl-3,14β-dihydro-4,5α-epoxy-6β-[1H-indazole-7-carboxamide]­morphinan Hydrochloride (2)

Compound **2** was synthesized as shown in the general procedure with 58% yield.^1^H NMR (400 MHz, DMSO-*d*_6_) δ: 12.98 (s, 1H), 9.32 (s, 1H), 8.87 (s, 1H), 8.85 (s, 1H), 8.14 (d, *J* = 1.4 Hz, 1H), 7.99 (t, *J* = 7.1 Hz, 2H), 7.23 (t, *J* = 7.4 Hz, 1H), 6.73 (d, *J* = 8.1 Hz, 1H), 6.67 (d, *J* = 8.1 Hz, 1H), 6.18 (s, 1H), 4.89 (d, *J* = 7.9 Hz, 1H), 3.87 (d, *J* = 5.1 Hz, 1H), 3.87 (m, 1H), 3.81–3.76 (m, 1H), 3.43–3.39 (m, 2H), 3.14–3.03 (m, 2H), 2.86 (m, 1H), 2.46–2.41 (m, 2H), 1.95 (m, 1H), 1.79 (m, 1H), 1.66 (m, 1H), 1.50 (m, 1H), 1.39 (m, 1H), 1.08 (m, 1H), 0.68 (m, 1H), 0.60 (m, 1H), 0.51 (m, 1H), 0.42 (m, 1H). ^13^C NMR (100 MHz, DMSO-*d*_6_) δ: 165.41, 142.14, 141.32, 137.78, 133.55, 129.68, 124.52, 124.51, 124.39, 120.57, 119.58, 119.31, 117.91, 116.82, 89.81, 69.77, 61.74, 56.71, 51.04, 46.52, 45.66, 29.46, 27.35, 23.84, 23.04, 5.72, 5.12, 2.63. HRMS *m*/*z* calc. 487.2345 for C_28_H_31_N_4_O_4_ [M + H]^+^; obs.: 487.2356 [M + H]^+^. The purity of the compound was checked by HPLC (Rt= 2.482 min) and was found to be 100.00% pure.

#### 17-Cyclopropylmethyl-3,14β-dihydro-4,5α-epoxy-6α-[1H-pyrrolo­[3,2-*b*]­pyridine-7-carboxamide]­morphinan Hydrochloride (3)

Compound **3** was synthesized as shown in the general procedure with 75% yield. ^1^H NMR (400 MHz, DMSO-*d*_6_) δ: 12.48 (s, 1H), 9.23 (s, 1H), 9.02 (d, *J* = 7.5 Hz, 1H), 8.92 (s, 1H), 8.78 (d, *J* = 5.8 Hz, 1H), 8.11 (t, *J* = 3.0 Hz, 1H), 8.00 (d, *J* = 5.8 Hz, 1H), 6.90 (dd, *J* = 3.0, 1.8 Hz, 1H), 6.74 (d, *J* = 8.1 Hz, 1H), 6.59 (d, *J* = 8.1 Hz, 1H), 6.46 (s, 1H), 4.87 (d, *J* = 3.9 Hz, 1H), 4.78–4.70 (m, 1H), 3.98 (d, *J* = 6.7 Hz, 1H), 3.30–3.28 (m, 2H), 3.12–3.04 (m, 2H), 3.00–2.95 (m, 1H), 2.78–2.70 (m, 1H), 2.55–2.53 (m, 1H), 2.02–1.95 (m, 1H), 1.71–1.60 (m, 2H), 1.53–1.47 (m, 1H), 1.30–1.21 (m, 1H), 1.12–1.05 (m, 1H), 0.71–0.67 (m, 1H), 0.65–0.60 (m, 1H), 0.52–0.48 (m, 1H), 0.43–0.39 (m, 1H). ^13^C NMR (100 MHz, DMSO-*d*_6_) δ: 163.68, 146.51, 139.37, 129.33, 129.10, 128.16, 122.59, 122.34, 119.71, 118.82, 116.50, 115.92, 114.71, 100.00, 87.22, 69.93, 61.49, 57.54, 50.45, 46.97, 45.77, 30.73, 29.54, 24.00, 19.49, 6.18, 5.66, 3.08. HRMS *m*/*z*: calc. 487.2345 for C_28_H_31_N_4_O_4_ [M + H]^+^; obs. 487.2335 [M + H]^+^. The purity of the compound was checked by HPLC (Rt= 2.295 min) and was found to be 98.30% pure.

#### 17-Cyclopropylmethyl-3,14β-dihydro-4,5α-epoxy-6β-[1H-pyrrolo­[3,2-*b*]­pyridine-7-carboxamide]­morphinan Hydrochloride (4)

Compound **4** was synthesized as shown in the general procedure with 76% yield. ^1^H NMR (400 MHz, DMSO-*d*_6_) δ: 12.43 (s, 1H), 9.59 (d, *J* = 8.0 Hz, 1H), 9.37 (s, 1H), 8.91 (s, 1H), 8.81 (d, *J* = 5.8 Hz, 1H), 8.08 (dd, *J* = 6.2, 4.5 Hz, 2H), 6.88 (dd, *J* = 3.0, 1.8 Hz, 1H), 6.76 (d, *J* = 8.1 Hz, 1H), 6.67 (d, *J* = 8.1 Hz, 1H), 6.30 (s, 1H), 4.93 (d, *J* = 7.7 Hz, 1H), 3.92 (d, *J* = 5.1 Hz, 1H), 3.83–3.77 (m, 1H), 3.28–3.24 (m, 2H), 3.14–3.03 (m, 3H), 2.92–2.86 (m, 1H), 2.46–2.43 (m, 1H), 2.09–2.00 (m, 1H), 1.85 (d, *J* = 13.7 Hz, 1H), 1.70–1.64 (m, 1H), 1.50–1.40 (m, 2H), 1.11–1.07 (m, 1H), 0.71–0.67 (m, 1H), 0.63–0.57 (m, 1H), 0.54–0.51 (m, 1H), 0.46–0.39 (m, 1H). ^13^C NMR (100 MHz, DMSO-*d*_6_) δ: 163.38, 142.47, 141.88, 136.33, 130.05, 129.57, 125.15, 121.08, 120.59, 119.94, 118.41, 116.25, 114.00, 99.99, 89.95, 70.18, 62.14, 57.21, 52.12, 46.96, 46.17, 29.88, 27.80, 23.99, 23.54, 6.21, 5.61, 3.13. HRMS *m*/*z*: calc. 487.2345 for C_28_H_31_N_4_O_4_ [M + H]^+^; obs.: 487.2316 [M + H]^+^. The purity of the compound was checked by HPLC (Rt= 2.297 min) and was found to be 99.06% pure.

#### 17-Cyclopropylmethyl-3,14β-dihydro-4,5α-epoxy-6α-[1H-pyrrolo­[3,2-*c*]­pyridine-7-carboxamide]­morphinan Hydrochloride (5)

Compound **5** was synthesized as shown in the general procedure with 89% yield. ^1^H NMR (400 MHz, DMSO-*d*_6_) δ: 12.76 (s, 1H), 9.40 (s, 1H), 9.26 (s, 1H), 9.08 (s, 1H), 9.02 (d, *J* = 7.4 Hz, 1H), 8.92 (s, 1H), 7.88 (dd, *J* = 5.5, 2.7 Hz, 1H), 7.11 (dd, *J* = 3.1, 1.5 Hz, 1H), 6.74 (d, *J* = 8.1 Hz, 1H), 6.60 (d, *J* = 8.1 Hz, 1H), 6.46 (s, 1H), 4.84 (d, *J* = 3.8 Hz, 1H), 4.79–4.70 (m, 1H), 3.98 (d, *J* = 6.8 Hz, 1H), 3.29–3.26 (m, 2H), 3.13–3.05 (m, 2H), 3.00–2.95 (m, 1H), 2.78–2.71 (m, 1H), 2.56–2.53 (m, 1H), 2.03–1.94 (m, 1H), 1.69–1.59 (m, 2H), 1.50 (dd, *J* = 14.7, 9.9 Hz, 1H), 1.31–1.22 (m, 1H), 1.13–1.06 (m, 1H), 0.74–0.67 (m, 1H), 0.65–0.60 (m, 1H), 0.53–0.47 (m, 1H), 0.45–0.38 (m, 1H). ^13^C NMR (100 MHz, DMSO-*d*_6_) δ: 162.94, 146.58, 139.97, 139.33, 137.85, 133.80, 130.74, 129.10, 126.07, 122.63, 119.71, 118.88, 115.66, 104.90, 87.30, 69.93, 61.45, 57.50, 52.73, 46.82, 45.74, 30.72, 29.54, 23.99, 19.49, 6.18, 5.67, 3.07. HRMS *m*/*z*: calc. 487.2345 for C_28_H_31_N_4_O_4_ [M + H]^+^; obs.: 487.2360 [M + H]^+^. The purity of the compound was checked by HPLC (Rt= 2.302 min) and was found to be 99.46% pure.

#### 17-Cyclopropylmethyl-3,14β-dihydro-4,5α-epoxy-6β-[1H-pyrrolo­[3,2-*c*]­pyridine-7-carboxamide]­morphinan Hydrochloride (6)

Compound **6** was synthesized as shown in the general procedure with 85% yield.^1^H NMR (400 MHz, DMSO-*d*_6_) δ: 12.69 (s, 1H), 9.59 (d, *J* = 7.9 Hz, 1H), 9.40 (s, 2H), 9.16 (s, 1H), 8.92 (s, 1H), 7.85 (d, *J* = 2.9 Hz, 1H), 7.09 (dd, *J* = 2.9, 1.3 Hz, 1H), 6.76 (d, *J* = 8.1 Hz, 1H), 6.68 (d, *J* = 8.1 Hz, 1H), 6.32 (s, 1H), 4.92 (d, *J* = 7.8 Hz, 1H), 3.93 (d, *J* = 5.3 Hz, 1H), 3.84–3.79 (m, 1H), 3.38–3.32 (m, 3H), 3.13–3.06 (m, 2H), 2.92–2.87 (m, 1H), 2.45–2.43 (m, 1H), 2.06–1.96 (m, 1H), 1.86 (d, *J* = 13.7 Hz, 1H), 1.71–1.64 (m, 1H), 1.52–1.39 (m, 2H), 1.13–1.05 (m, 1H), 0.72–0.66 (m, 1H), 0.64–0.58 (m, 1H), 0.56–0.50 (m, 1H), 0.45–0.40 (m, 1H). ^13^C NMR (100 MHz, DMSO-*d*_6_) δ: 162.86, 142.46, 141.89, 139.93, 137.95, 133.85, 130.34, 130.06, 126.25, 121.08, 119.92, 118.37, 115.41, 104.82, 90.01, 70.16, 62.09, 57.16, 51.90, 46.97, 46.15, 29.86, 27.78, 24.08, 23.51, 6.22, 5.62, 3.11. HRMS *m*/*z*: calc. 487.2345 for C_28_H_31_N_4_O_4_ [M + H]^+^; obs.: 487.2351 [M + H]^+^. The purity of the compound was checked by HPLC (Rt= 2.300 min) and was found to be 99.01% pure.

#### 17-Cyclopropylmethyl-3,14β-dihydro-4,5α-epoxy-6α-[1H-pyrrolo­[2,3-*c*]­pyridine-7-carboxamide]­morphinan Hydrochloride (7)

Compound **7** was synthesized as shown in the general procedure with 80% yield. ^1^H NMR (400 MHz, DMSO-*d*_6_) δ: 11.91 (s, 1H), 8.93 (s, 1H), 8.69 (d, *J* = 7.7 Hz, 1H), 8.24 (d, *J* = 5.5 Hz, 1H), 7.93 (d, *J* = 5.5 Hz, 1H), 7.82 (s, 1H), 6.76 (d, *J* = 8.1 Hz, 1H), 6.73 (s, 1H), 6.60 (d, *J* = 8.1 Hz, 1H), 4.82 (d, *J* = 3.8 Hz, 1H), 4.79–4.71 (m, 1H), 3.98 (d, *J* = 6.6 Hz, 1H), 3.40–3.27 (m, 2H), 3.12–2.96 (m, 3H), 2.78–2.69 (m, 1H), 2.57–2.53 (m, 1H), 2.04–1.95 (m, 1H), 1.70–1.67 (m, 2H), 1.49 (dd, *J* = 15.1, 9.7 Hz, 1H), 1.15–1.05 (m, 2H), 0.75–0.67 (m, 1H), 0.67–0.61 (m, 1H), 0.52–0.48 (m, 1H), 0.43–0.39 (m, 1H). ^13^C NMR (100 MHz, DMSO-*d*_6_) δ: 163.75, 146.24, 139.44, 136.60, 134.74, 134.48, 131.08, 129.20, 122.59, 119.90, 118.99, 118.76, 101.98, 87.99, 69.88, 61.38, 57.50, 45.83, 45.67, 30.66, 29.69, 23.99, 20.37, 6.19, 5.68, 3.06. HRMS *m*/*z*: calc. 487.2345 for C_28_H_31_N_4_O_4_ [M + H]^+^; obs.: 487.2363 [M + H]^+^. The purity of the compound was checked by HPLC (Rt= 2.482 min) and was found to be 99.69% pure.

#### 17-Cyclopropylmethyl-3,14β-dihydro-4,5α-epoxy-6β-[1H-pyrrolo­[2,3-*c*]­pyridine-7-carboxamide]­morphinan Hydrochloride (8)

Compound **8** was synthesized as shown in the general procedure with 62% yield. ^1^H NMR (400 MHz, DMSO-*d*_6_) δ: 11.98 (s, 1H), 9.45 (s, 1H), 8.90 (s, 1H), 8.24 (d, *J* = 5.6 Hz, 1H), 7.96 (d, *J* = 5.6 Hz, 1H), 7.82 (s, 1H), 6.76 (d, *J* = 8.2 Hz, 1H), 6.74 (s, 1H), 6.67 (d, *J* = 8.2 Hz, 1H), 5.04 (d, *J* = 7.7 Hz, 1H), 3.90 (d, *J* = 5.2 Hz, 1H), 3.84–3.76 (m, 1H), 3.40–3.30 (m, 2H), 3.14–3.02 (m, 2H), 2.92–2.85 (m, 1H), 2.48–2.45 (m, 2H), 2.13–2.03 (m, 1H), 1.81 (d, *J* = 13.8 Hz, 1H), 1.67–1.60 (m, 1H), 1.50–1.40 (m, 2H), 1.11–1.07 (m, 1H), 0.72–0.66 (m, 1H), 0.63–0.60 (m, 1H), 0.54–0.51 (m, 1H), 0.46–0.43 (m, 1H). ^13^C NMR (100 MHz, DMSO-*d*_6_) δ: 161.80, 142.59, 141.86, 132.92, 131.04, 130.18, 121.07, 119.82, 118.98, 118.36, 117.45, 102.08, 90.26, 70.21, 66.93, 62.07, 57.15, 51.55, 46.97, 46.17, 30.06, 27.81, 24.10, 23.49, 6.23, 5.61, 3.11. HRMS *m*/*z*: calc. 487.2345 for C_28_H_31_N_4_O_4_ [M + H]^+^; obs.: 487.2346 [M + H]^+^. The purity of the compound was checked by HPLC (Rt= 2.452 min) and was found to be 99.88% pure.

#### 17-Cyclopropylmethyl-3,14β-dihydro-4,5α-epoxy-6α-[1H-indazole-6-carboxamide]­morphinan Hydrochloride (9)

Compound **9** was synthesized as shown in the general procedure with 78% yield. ^1^H NMR (400 MHz, DMSO-*d*_6_) δ: 8.88 (s, 1H), 8.20 (d, *J* = 7.6 Hz, 1H), 8.15 (d, *J* = 0.9 Hz, 1H), 8.09 (s, 1H), 7.85 (d, *J* = 8.5 Hz, 1H), 7.61 (d, *J* = 8.5 Hz, 1H), 6.73 (d, *J* = 8.1 Hz, 1H), 6.58 (d, *J* = 8.1 Hz, 1H), 4.81 (d, *J* = 3.9 Hz, 1H), 4.63 (m, 1H), 3.94 (d, *J* = 6.7 Hz, 1H), 3.36 (m, 1H), 3.29 (m, 1H), 3.10 (m, 1H), 3.05 (m, 1H), 2.97 (m, 1H), 2.73 (m, 1H), 2.54 (m, 1H), 1.94 (m, 1H), 1.65 (m, 1H), 1.54 (m, 1H), 1.46 (m, 1H), 1.19 (m, 1H), 1.08 (m, 1H), 0.69 (m, 1H), 0.62 (m, 1H), 0.49 (m, 1H), 0.41 (m, 1H). ^13^C NMR (100 MHz, DMSO-*d*_6_) δ: 166.46, 146.10, 139.37, 138.82, 133.40, 132.20, 128.74, 124.23, 122.10, 120.24, 119.53, 119.10, 118.31, 109.96, 87.18, 69.39, 61.05, 57.02, 52.45, 46.12, 45.23, 30.25, 29.24, 23.50, 19.37, 5.70, 5.18, 2.56. HRMS *m*/*z*: calc. 487.2345 for C_28_H_31_N_4_O_4_ [M + H]^+^; obs.: 487.2400 [M + H]^+^. The purity of the compound was checked by HPLC (Rt= 2.453 min) and was found to be 99.95% pure.

#### 17-Cyclopropylmethyl-3,14β-dihydro-4,5α-epoxy-6β-[1H-indazole-6-carboxamide]­morphinan Hydrochloride (10)

Compound **10** was synthesized as shown in the general procedure with 70% yield. ^1^H NMR (400 MHz, DMSO-*d*_6_) δ: 13.41 (s, 1H), 9.33 (s, 1H), 8.85 (s, 1H), 8.78 (d, *J* = 8.0 Hz, 1H), 8.15 (s, 1H), 8.10 (s, 1H), 7.83 (d, *J* = 8.5 Hz, 1H), 7.64 (d, *J* = 8.5 Hz, 1H), 6.73 (d, *J* = 8.1 Hz, 1H), 6.66 (d, *J* = 8.1 Hz, 1H), 6.15 (s, 1H), 4.87 (d, *J* = 7.8 Hz, 1H), 3.86 (d, *J* = 5.1 Hz, 1H), 3.73 (m, 1H), 3.44–3.38 (m, 2H), 3.11 (m, 1H), 3.04 (m, 1H), 2.86 (m, 1H), 2.46–2.41 (m, 2H), 1.90 (m, 1H), 1.77 (m, 1H), 1.63 (m, 1H), 1.47 (m, 1H), 1.42 (m, 1H), 1.05 (m, 1H), 0.68 (m, 1H), 0.59 (m, 1H), 0.51 (m, 1H), 0.42 (m, 1H). ^13^C NMR (100 MHz, DMSO-*d*_6_) δ: 165.99, 142.17, 141.30, 139.40, 133.46, 131.98, 129.68, 124.25, 120.58, 120.23, 119.20, 117.92, 109.62, 89.84, 69.75, 61.75, 56.71, 51.32, 48.55, 46.50, 45.62, 29.41, 27.35, 23.76, 23.03, 5.70, 5.09, 2.61. HRMS *m*/*z*: calc. 487.2345 for C_28_H_31_N_4_O_4_ [M + H]^+^; obs.: 487.2323 [M + H]^+^. The purity of the compound was checked by HPLC (Rt = 2.452 min) and was found to be 99.92% pure.

#### 17-Cyclopropylmethyl-3,14β-dihydro-4,5α-epoxy-6α-[1H-pyrrolo­[3,2-*b*]­pyridine-6-carboxamide]­morphinan Hydrochloride(11)

Compound **11** was synthesized as shown in the general procedure with 82% yield.^1^H NMR (400 MHz, DMSO-*d*_6_) δ: 12.82 (s, 1H), 9.21 (s, 1H), 9.11 (s, 1H), 8.84 (s, 1H), 8.84 (s, 1H), 8.66 (d, *J* = 7.2 Hz, 1H), 8.26 (s, 1H), 6.88 (s, 1H), 6.73 (d, *J* = 8.1 Hz, 1H), 6.59 (d, *J* = 8.1 Hz, 1H), 6.34 (s, 1H), 4.80 (d, *J* = 3.8 Hz, 1H), 4.71–4.64 (m, 1H), 3.94 (d, *J* = 6.8 Hz, 1H), 3.27–3.23 (m, 2H), 3.11–3.05 (m, 2H), 2.98–2.93 (m, 1H), 2.77–2.68 (m, 1H), 2.56–2.54 (m, 1H), 1.98–1.89 (m, 1H), 1.67–1.47 (m, 3H), 1.29–1.19 (m, 1H), 1.11–1.09 (m, 1H), 0.74–0.67 (m, 1H), 0.65–0.61 (m, 1H), 0.52–0.47 (m, 1H), 0.44–0.37 (m, 1H). ^13^C NMR (100 MHz, DMSO-*d*_6_) δ: 163.58, 146.64, 139.33, 138.91, 131.29, 129.17, 125.82, 123.45, 122.62, 119.64, 118.95, 118.62, 87.45, 69.89, 61.53, 57.52, 50.27, 46.94, 45.74, 30.68, 29.62, 24.00, 19.73, 6.18, 5.66, 3.05. HRMS *m*/*z*: calc. 487.2345 for C_28_H_31_N_4_O_4_ [M + H]^+^; obs.: 487.2347 [M + H]^+^. The purity of the compound was checked by HPLC (Rt= 2.290 min) and was found to be 99.81% pure.

#### 17-Cyclopropylmethyl-3,14β-dihydro-4,5α-epoxy-6β-[1H-pyrrolo­[3,2-*b*]­pyridine-6-carboxamide]­morphinan Hydrochloride (12)

Compound **12** was synthesized as shown in the general procedure with 79% yield. ^1^H NMR (400 MHz, DMSO-*d*_6_) δ: 13.12 (s, 1H), 9.33 (d, *J* = 7.8 Hz, 2H), 9.18 (s, 1H), 8.96 (s, 1H), 8.89 (s, 1H), 8.33 (t, *J* = 2.9 Hz, 1H), 6.91 (s, 1H), 6.75 (d, *J* = 8.1 Hz, 1H), 6.67 (d, *J* = 8.1 Hz, 1H), 6.27 (s, 1H), 4.90 (d, *J* = 7.8 Hz, 1H), 3.91 (d, *J* = 5.1 Hz, 1H), 3.79–3.73 (m, 1H), 3.30–3.28 (m, 2H), 3.13–3.05 (m, 2H), 2.92–2.86 (m, 1H), 2.47–2.44 (m, 1H), 2.01–1.92 (m, 1H), 1.82 (d, *J* = 13.7 Hz, 1H), 1.68–1.62 (m, 1H), 1.49–1.41 (m, 2H), 1.12–1.06 (m, 1H), 0.68 (dd, *J* = 8.5, 4.9 Hz, 1H), 0.71–0.66 (m, 1H), 0.56–0.49 (m, 1H), 0.44–0.41 (m, 1H). ^13^C NMR (100 MHz, DMSO-*d*_6_) δ: 163.18, 142.57, 141.86, 139.02, 131.50, 130.10, 127.63, 125.49, 123.31, 121.12, 119.89, 118.45, 90.13, 70.19, 62.18, 57.20, 52.13, 46.99, 46.14, 29.85, 27.82, 24.18, 23.53, 6.22, 5.60, 3.13. HRMS *m*/*z*: calc. 487.2345 for C_28_H_31_N_4_O_4_ [M + H]^+^; obs.: 487.2343 [M + H]^+^. The purity of the compound was checked by HPLC (Rt= 2.295 min) and was found to be 99.16% pure.

#### 17-Cyclopropylmethyl-3,14β-dihydro-4,5α-epoxy-6α-[1H-pyrrolo­[3,2-*c*]­pyridine-6-carboxamide]­morphinan Hydrochloride (13)

Compound **13** was synthesized as shown in the general procedure with 55% yield. ^1^H NMR (400 MHz, DMSO-*d*_6_) δ: 13.24 (s, 1H), 9.18 (s, 1H), 9.10 (s, 1H), 8.93 (s, 1H), 8.69 (s, 1H), 7.99 (s, 1H), 7.04 (s, 1H), 6.76 (d, *J* = 8.1 Hz, 1H), 6.60 (d, *J* = 8.1 Hz, 1H), 6.48 (s, 1H), 4.79 (d, *J* = 3.8 Hz, 1H), 4.72–4.69 (m, 1H), 3.99 (d, *J* = 6.7 Hz, 1H), 3.31–3.27 (m, 2H), 3.11–2.98 (m, 3H), 2.76–2.70 (m, 1H), 2.57–2.51 (m, 1H), 2.02–1.93 (m, 1H), 1.67–1.60 (m, 2H), 1.49 (dd, *J* = 14.9, 9.7 Hz, 1H), 1.25–1.19 (m, 1H), 1.12–1.08 (m, 1H), 0.72–0.68 (m, 1H), 0.65–0.59 (m, 1H), 0.52–0.48 (m, 1H), 0.42–0.39 (m, 1H). ^13^C NMR (100 MHz, DMSO-*d*_6_) δ: 146.03, 140.55, 138.90, 134.43, 128.67, 125.46, 122.15, 119.28, 118.50, 107.93, 87.05, 69.40, 60.94, 57.02, 46.37, 45.31, 45.22, 30.19, 29.10, 23.53, 19.41, 5.71, 5.19, 2.59.] HRMS *m*/*z*: calc. 487.2345 for C_28_H_31_N_4_O_4_ [M + H]^+^; obs.: 487.2319 [M + H]^+^. The purity of the compound was checked by HPLC (Rt= 2.305 min) and was found to be 99.33% pure.

#### 17-Cyclopropylmethyl-3,14β-dihydro-4,5α-epoxy-6β-[1H-pyrrolo­[3,2-*c*]­pyridine-6-carboxamide]­morphinan Hydrochloride (14)

Compound **14** was synthesized as shown in the general procedure with 67% yield. ^1^H NMR (400 MHz, DMSO-*d*_6_) δ: 13.50 (s, 1H), 9.80 (s, 1H), 9.39 (s, 1H), 9.20 (s, 1H), 8.91 (s, 1H), 8.76 (s, 1H), 8.04 (s, 1H), 7.08 (s, 1H), 6.76 (d, *J* = 8.1 Hz, 1H), 6.68 (d, *J* = 8.1 Hz, 1H), 6.32 (s, 1H), 4.96 (d, *J* = 7.7 Hz, 1H), 3.93 (d, *J* = 4.7 Hz, 1H), 3.83–3.76 (m, 1H), 3.28–3.26 (m, 2H), 3.15–3.03 (m, 3H), 2.93–2.87 (m, 1H), 2.44–2.41 (m, 1H), 2.06–1.97 (m, 1H), 1.84 (d, *J* = 13.6 Hz, 1H), 1.69–1.61 (m, 1H), 1.50–1.42 (m, 2H), 1.10–1.08 (m, 1H), 0.72–0.69 (m, 1H), 0.64–0.58 (m, 1H), 0.55–0.52 (m, 1H), 0.46–0.39 (m, 1H). ^13^C NMR (100 MHz, DMSO-*d*_6_) δ: 142.49, 141.92, 141.34, 137.15, 130.07, 127.93, 125.86, 121.13, 119.93, 118.45, 108.12, 90.03, 70.16, 62.10, 57.18, 55.37, 52.25, 46.97, 46.16, 29.88, 27.78, 24.04, 23.51, 6.22, 5.62, 3.11. HRMS *m*/*z*: calc. 487.2345 for C_28_H_31_N_4_O_4_ [M + H]^+^; obs.: 487.2318 [M + H]^+^. The purity of the compound was checked by HPLC (Rt= 2.308 min) and was found to be 99.29% pure.

#### 17-Cyclopropylmethyl-3,14β-dihydro-4,5α-epoxy-6α-[1H-pyrrolo­[2,3-*b*]­pyridine-6-carboxamide]­morphinan Hydrochloride (15)

Compound **15** was synthesized as shown in the general procedure with 77% yield. ^1^H NMR (400 MHz, DMSO-*d*_6_) δ: 12.10 (s, 1H), 8.94 (s, 1H), 8.14 (d, *J* = 8.0 Hz, 1H), 8.12 (d, *J* = 7.3 Hz, 1H), 7.85 (d, *J* = 8.1 Hz, 1H), 7.71–7.68 (m, 1H), 6.81 (d, *J* = 8.1 Hz, 1H), 6.61 (d, *J* = 8.1 Hz, 1H), 6.58 (dd, *J* = 3.4, 1.8 Hz, 1H), 4.76 (d, *J* = 3.9 Hz, 1H), 4.75–4.69 (m, 1H), 3.99 (d, *J* = 6.8 Hz, 1H), 3.41–3.24 (m, 2H), 3.11–2.99 (m, 3H), 2.78–2.68 (m, 1H), 2.58–2.52 (m, 1H), 2.02–1.99 (m, 1H), 1.67–1.60 (m, 2H), 1.46 (dd, *J* = 15.1, 9.8 Hz, 1H), 1.13–0.98 (m, 2H), 0.74–0.67 (m, 1H), 0.65–0.60 (m, 1H), 0.54–0.48 (m, 1H), 0.44–0.41 (m, 1H). ^13^C NMR (100 MHz, DMSO-*d*_6_) δ: 164.54, 147.19, 146.12, 142.93, 139.46, 130.01, 129.42, 129.27, 123.01, 122.53, 119.87, 118.60, 114.25, 100.83, 88.45, 69.88, 61.35, 57.50, 45.85, 45.63, 45.52, 30.72, 29.74, 23.98, 20.62, 6.20, 5.68, 3.06. HRMS *m*/*z* calc. 487.2345 for C_28_H_31_N_4_O_4_ [M + H]^+^; obs.: 487.2337 [M + H]^+^. The purity of the compound was checked by HPLC (Rt= 2.560 min) and was found to be 99.39% pure.

#### 17-Cyclopropylmethyl-3,14β-dihydro-4,5α-epoxy-6β-[1H-pyrrolo­[2,3-*b*]­pyridine-6-carboxamide]­morphinan Hydrochloride (16)

Compound **16** was synthesized as shown in the general procedure with 69% yield. ^1^H NMR (400 MHz, DMSO-*d*_6_) δ: 11.80 (s, 1H), 8.88 (s, 1H), 8.64 (d, *J* = 8.6 Hz, 1H), 8.11 (d, *J* = 8.1 Hz, 1H), 7.77 (d, *J* = 8.1 Hz, 1H), 7.71–7.67 (m, 1H), 6.74 (d, *J* = 8.1 Hz, 1H), 6.66 (d, *J* = 8.1 Hz, 1H), 6.57 (dd, *J* = 3.4, 1.8 Hz, 1H), 5.03 (d, *J* = 7.8 Hz, 1H), 3.89 (d, *J* = 5.2 Hz, 1H), 3.70–3.67 (m, 2H), 3.37–3.30 (m, 2H), 3.09–3.04 (m, 2H), 2.90–2.85 (m, 1H), 2.48–2.40 (m, 1H), 2.08–1.99 (m, 1H), 1.75 (d, *J* = 13.7 Hz, 1H), 1.66–1.58 (m, 1H), 1.49–1.39 (m, 2H), 1.12–1.06 (m, 1H), 0.72–0.65 (m, 1H), 0.62–0.57 (m, 1H), 0.54–0.50 (m, 1H), 0.43–0.40 (m, 1H). ^13^C NMR (100 MHz, DMSO-*d*_6_) δ: 165.19, 147.07, 143.64, 142.66, 141.81, 130.29, 129.83, 129.23, 122.71, 121.09, 119.73, 118.37, 114.32, 100.80, 90.61, 70.26, 62.01, 57.13, 51.46, 46.98, 46.20, 30.19, 27.79, 24.27, 23.46, 6.22, 5.61, 3.10. HRMS *m*/*z*: calc. 487.2345 for C_28_H_31_N_4_O_4_ [M + H]^+^: 487.2340; obs.: 487.2327 [M + H]^+^. The purity of the compound was checked by HPLC (Rt= 2.543 min) and was found to be 99.37% pure.

#### 17-Cyclopropylmethyl-3,14β-dihydro-4,5α-epoxy-6α-[1H-indazole-5-carboxamide]­morphinan Hydrochloride (17)

Compound **17** was synthesized as shown in the general procedure with 64% yield.^1^H NMR (400 MHz, DMSO-*d*_6_) δ: 8.89 (s, 1H), 8.39 (s, 1H), 8.22 (d, *J* = 0.8 Hz, 1H), 8.05 (d, *J* = 7.7 Hz, 1H), 7.89 (d, *J* = 8.8 Hz, 1H), 7.60 (d, *J* = 8.8 Hz, 1H), 6.73 (d, *J* = 8.1 Hz, 1H), 6.58 (d, *J* = 8.1 Hz, 1H), 4.80 (d, *J* = 3.8 Hz, 1H), 4.64 (m, 1H), 3.95 (d, *J* = 6.7 Hz, 1H), 3.36 (m, 1H), 3.25 (m, 1H), 3.09 (m, 1H), 3.04 (m, 1H), 2.98 (m, 1H), 2.73 (m, 1H), 2.54 (m, 1H), 1.94 (m, 1H), 1.64 (m, 1H), 1.53 (m, 1H), 1.45 (m, 1H), 1.20 (m, 1H), 1.08 (m, 1H), 0.69 (m, 1H), 0.61 (m, 1H), 0.50 (m, 1H), 0.40 (m, 1H). ^13^C NMR (100 MHz, DMSO-*d*_6_) δ: 166.32, 146.10, 140.95, 138.81, 134.68, 128.76, 126.84, 125.50, 122.25, 122.10, 120.85, 119.10, 118.31, 109.77, 87.32, 69.40, 61.04, 57.01, 45.98, 45.22, 30.24, 29.25, 23.51, 19.45, 5.70, 5.18, 2.56. HRMS *m*/*z*: calc. 487.2345 for C_28_H_31_N_4_O_4_ [M + H]^+^; obs.: 487.2340 [M + H]^+^. The purity of the compound was checked by HPLC (Rt= 2.437 min) and was found to be 99.89% pure.

#### 17-Cyclopropylmethyl-3,14β-dihydro-4,5α-epoxy-6β-[1H-indazole-5-carboxamide]­morphinan Hydrochloride (18)

Compound **18** was synthesized as shown in the general procedure with 59% yield. ^1^H NMR (400 MHz, DMSO-*d*_6_) δ: 8.87 (s, 1H), 8.68 (d, *J* = 8.0 Hz, 1H), 8.39 (s, 1H), 8.22 (d, *J* = 0.9 Hz, 1H), 7.90 (dd, *J* = 8.8 Hz, 1.5 Hz, 1H), 7.59 (d, *J* = 8.8 Hz, 1H), 6.73 (d, *J* = 8.1 Hz, 1H), 6.66 (d, *J* = 8.1 Hz, 1H), 6.20 (s, 1H), 4.87 (d, *J* = 7.8 Hz, 1H), 3.88 (d, *J* = 5.2 Hz, 1H), 3.72 (m, 1H), 3.33 (m, 1H), 3.30 (m, 1H), 3.10 (m, 1H), 3.04 (m, 1H), 2.87 (m, 1H), 2.47 (m, 1H), 2.45 (m, 1H), 1.91 (m, 1H), 1.78 (m, 1H), 1.62 (m, 1H), 1.48 (m, 1H), 1.40 (m, 1H), 1.07 (m, 1H), 0.68 (m, 1H), 0.59 (m, 1H), 0.52 (m, 1H), 0.42 (m, 1H). ^13^C NMR (100 MHz, DMSO-*d*_6_) δ: 165.96, 142.20, 141.29, 140.93, 134.69, 129.70, 126.70, 125.20, 123.83, 122.33, 120.58, 119.23, 117.90, 109.75, 89.95, 69.76, 61.76, 56.70, 51.21, 46.50, 45.60, 29.40, 27.36, 23.85, 23.04, 5.71, 5.10, 2.62. HRMS *m*/*z*: calc. 487.2345 for C_28_H_31_N_4_O_4_ [M + H]^+^; obs.: 487.2338 [M + H]^+^. The purity of the compound was checked by HPLC (Rt= 2.435 min) and was found to be 99.75% pure.

#### 17-Cyclopropylmethyl-3,14β-dihydro-4,5α-epoxy-6α-[1H-benzo­[d]­imidazole-5-carboxamide]­morphinan Hydrochloride (19)

Compound **19** was synthesized as shown in the general procedure with 40% yield. ^1^H NMR (400 MHz, DMSO-*d*_6_) δ: 9.42 (s, 1H), 9.23 (s, 1H), 8.86 (s, 1H), 8.38 (d, *J* = 7.6 Hz, 1H), 8.35 (s, 1H), 8.04 (d, *J* = 8.6 Hz, 1H), 7.88 (d, *J* = 8.6 Hz, 1H), 6.73 (d, *J* = 8.1 Hz, 1H), 6.58 (d, *J* = 8.1 Hz, 1H), 6.34 (s, 1H), 4.80 (d, *J* = 3.8 Hz, 1H), 4.68–4.60 (m, 1H), 3.94 (d, *J* = 6.6 Hz, 1H), 3.20–3.17 (m, 2H), 3.11–3.05 (m, 2H), 2.98–2.94 (m, 1H), 2.77–2.67 (m, 1H), 2.55–2.52 (m, 1H), 1.97–1.90 (m, 1H), 1.67–1.62 (m, 1H), 1.57–1.51 (m, 1H), 1.49–1.44 (m, 1H), 1.26–1.18 (m, 1H), 1.10–1.06 (m, 1H), 0.74–0.67 (m, 1H), 0.66–0.59 (m, 1H), 0.52–0.47 (m, 1H), 0.43–0.37 (m, 1H).^13^C NMR (100 MHz, DMSO-*d*_6_) δ: 165.39, 146.15, 142.35, 138.83, 133.31, 131.70, 131.37, 128.74, 125.01, 122.14, 119.12, 118.39, 114.38, 114.19, 87.09, 69.41, 64.89, 61.02, 57.02, 46.31, 45.24, 30.26, 29.21, 23.53, 19.29, 5.72, 5.20, 2.58. HRMS *m*/*z*: calc. 487.2345 for C_28_H_31_N_4_O_4_ [M + H]^+^; obs.: 487.2360 [M + H]^+^. The purity of the compound was checked by HPLC (Rt= 2.287 min) and was found to be 99.77% pure.

#### 17-Cyclopropylmethyl-3,14β-dihydro-4,5α-epoxy-6β-[1H-benzo­[d]­imidazole-5-carboxamide]­morphinan Hydrochloride (20)

Compound **20** was synthesized as shown in the general procedure with 25% yield. ^1^H NMR (400 MHz, DMSO-*d*_6_) δ: 9.34 (s, 1H), 9.27 (s, 1H), 8.90 (d, *J* = 8.1 Hz, 1H), 8.85 (s, 1H), 8.33 (s, 1H), 8.02 (dd, *J* = 8.6, 1.3 Hz, 1H), 7.85 (d, *J* = 8.6 Hz, 1H), 6.73 (d, *J* = 8.1 Hz, 1H), 6.67 (d, *J* = 8.1 Hz, 1H), 6.19 (s, 1H), 4.88 (d, *J* = 7.8 Hz, 1H), 3.88 (d, *J* = 5.0 Hz, 1H), 3.77–3.70 (m, 1H), 3.34–3.29 (m, 2H), 3.13–3.04 (m, 2H), 2.89–2.84 (m, 1H), 2.47–2.42 (m, 2H), 1.95–1.87 (m, 1H), 1.80–1.76 (m, 1H), 1.65–1.60 (m, 1H), 1.49–1.40 (m, 2H), 1.10–1.04 (s, 1H), 0.71–0.66 (m, 1H), 0.63–0.58 (m, 1H), 0.55–0.49 (m, 1H), 0.44–0.38 (m, 1H). ^13^C NMR (100 MHz, DMSO-*d*_6_) δ: 165.13, 142.62, 142.17, 141.37, 134.67, 134.07, 131.18, 129.72, 124.39, 120.68, 119.37, 117.97, 114.36, 114.19, 89.83, 69.79, 61.75, 56.74, 51.48, 46.55, 45.69, 29.46, 27.38, 23.81, 23.08, 5.77, 5.18, 2.66. HRMS *m*/*z*: calc. 487.2345 for C_28_H_31_N_4_O_4_ [M + H]^+^; obs.: 487.2317 [M + H]^+^. The purity of the compound was checked by HPLC (Rt= 2.287 min) and was found to be 99.05% pure.

#### 17-Cyclopropylmethyl-3,14β-dihydro-4,5α-epoxy-6α-[1H-pyrrolo­[3,2-*b*]­pyridine-5-carboxamide]­morphinan Hydrochloride (21)

Compound **21** was synthesized as shown in the general procedure with 83% yield. ^1^H NMR (400 MHz, DMSO-*d*_6_) δ: 11.80 (s, 1H), 8.88 (s, 1H), 8.39 (d, *J* = 8.7 Hz, 1H), 8.00 (d, *J* = 8.5 Hz, 1H), 7.93 (d, *J* = 8.4 Hz, 1H), 7.86 (t, *J* = 2.9 Hz, 1H), 6.76 (d, *J* = 8.1 Hz, 1H), 6.73 (s, 1H), 6.61 (d, *J* = 8.1 Hz, 1H), 6.37 (s, 1H), 4.77 (d, *J* = 3.7 Hz, 1H), 4.74–4.67 (m, 1H), 3.94 (d, *J* = 6.6 Hz, 1H), 3.39–3.32 (m, 2H), 3.31–3.26 (m, 1H), 3.12–3.04 (m, 2H), 3.00–2.94 (m, 1H), 2.78–2.69 (m, 1H), 2.5–2.51 (m, 1H), 1.99–1.90 (m, 1H), 1.69–1.59 (m, 2H), 1.48 (dd, *J* = 15.2, 9.8 Hz, 1H), 1.10–1.04 (m, 2H), 0.74–0.67 (m, 1H), 0.65–0.59 (m, 1H), 0.51–0.46 (m, 1H), 0.43–0.38 (m, 1H). ^13^C NMR (100 MHz, DMSO-*d*_6_) δ: 164.36, 146.18, 142.65, 139.43, 132.59, 130.87, 129.24, 122.51, 120.16, 119.87, 118.71, 115.27, 102.08, 88.31, 69.86, 61.50, 57.52, 49.05, 45.82, 45.66, 30.69, 29.72, 23.95, 20.61, 6.17, 5.65, 3.04. HRMS *m*/*z*: calc. 487.2345 for C_28_H_31_N_4_O_4_ [M + H]^+^; obs.: 487.2363 [M + H]^+^. The purity of the compound was checked by HPLC (Rt= 2.392 min) and was found to be 99.94% pure.

#### 17-Cyclopropylmethyl-3,14β-dihydro-4,5α-epoxy-6β-[1H-pyrrolo­[3,2-*b*]­pyridine-5-carboxamide]­morphinan Hydrochloride (22)

Compound **22** was synthesized as shown in the general procedure with 69% yield. ^1^H NMR (400 MHz, DMSO-*d*_6_) δ: 12.01 (s, 1H), 9.08 (d, *J* = 6.9 Hz, 1H), 8.85 (s, 1H), 8.11 (d, *J* = 8.0 Hz, 1H), 7.98 (d, *J* = 8.5 Hz, 1H), 7.94 (s, 1H), 6.73 (d, *J* = 8.2 Hz, 1H), 6.72–6.70 (m, 1H), 6.66 (d, *J* = 8.2 Hz, 1H), 6.21 (s, 1H), 5.02 (d, *J* = 7.7 Hz, 1H), 3.87 (d, *J* = 4.9 Hz, 1H), 3.77–3.70 (m, 2H), 3.37–3.32 (m, 2H), 3.13–3.01 (m, 3H), 2.89–2.84 (m, 1H), 2.47–2.44 (m, 1H), 2.05–1.96 (m, 1H), 1.76 (d, *J* = 13.6 Hz, 1H), 1.64–1.58 (m, 1H), 1.48–1.40 (m, 2H), 1.12–1.07 (m, 1H), 0.71–0.68 (m, 1H), 0.63–0.58 (m, 1H), 0.53–0.49 (m, 1H), 0.46–0.44 (m, 1H). ^13^C NMR (100 MHz, DMSO-*d*_6_) δ: 161.39, 142.65, 141.82, 130.21, 121.08, 119.77, 118.38, 115.29, 90.46, 70.24, 62.13, 57.16, 51.60, 46.97, 46.13, 30.03, 27.83, 24.21, 23.46, 6.20, 5.59, 3.09. HRMS *m*/*z*: calc. 487.2345 for C_28_H_31_N_4_O_4_ [M + H]^+^; obs.: 487.2338 [M + H]^+^. The purity of the compound was checked by HPLC (Rt= 2.372 min) and was found to be 99.88% pure.

#### 17-Cyclopropylmethyl-3,14β-dihydro-4,5α-epoxy-6α-[1H-pyrrolo­[2,3-*c*]­pyridine-5-carboxamide]­morphinan Hydrochloride (23)

Compound **23** was synthesized as shown in the general procedure with 58% yield. ^1^H NMR (400 MHz, DMSO-*d*_6_) δ: 12.91 (s, 1H), 9.32 (s, 1H), 9.00 (s, 1H), 8.93 (s, 1H), 8.84 (s, 1H), 8.77 (s, 1H), 8.15 (s, 1H), 6.96 (s, 1H), 6.76 (d, *J* = 8.0 Hz, 1H), 6.60 (d, *J* = 8.0 Hz, 1H), 6.47 (s, 1H), 4.78 (d, *J* = 3.6 Hz, 1H), 4.70–4.68 (m, 1H), 3.99 (d, *J* = 4.8 Hz, 1H), 3.37–3.21 (m, 2H), 3.12–3.01 (m, 3H), 2.77–2.69 (m, 1H), 2.56–2.54 (m, 1H), 1.99–1.93 (m, 1H), 1.67–1.58 (m, 2H), 1.54–1.44 (m, 1H), 1.18–1.10 (m, 2H), 0.72–0.68 (m, 1H), 0.65–0.61 (m, 1H), 0.53–0.48 (m, 1H), 0.44–0.41 (m, 1H). ^13^C NMR (100 MHz, DMSO-*d*_6_) δ: 159.75, 146.42, 139.40, 129.18, 125.33, 122.60, 119.78, 118.89, 116.10, 109.20, 87.71, 69.88, 61.40, 57.49, 45.79, 30.67, 29.61, 24.00, 6.20, 5.68, 3.07. HRMS *m*/*z*: calc. 487.2345 for C_28_H_31_N_4_O_4_ [M + H]^+^; obs.: 487.2362 [M + H]^+^. The purity of the compound was checked by HPLC (Rt= 2.310 min) and was found to be 99.86% pure.

#### 17-Cyclopropylmethyl-3,14β-dihydro-4,5α-epoxy-6β-[1H-pyrrolo­[2,3-*c*]­pyridine-5-carboxamide]­morphinan Hydrochloride (24)

Compound **24** was synthesized as shown in the general procedure with 80% yield. ^1^H NMR (400 MHz, DMSO-*d*_6_) δ: 13.11 (s, 1H), 9.60 (s, 1H), 9.38 (s, 1H), 9.01 (s, 1H), 8.89 (s, 1H), 8.86 (s, 1H), 8.25 (s, 1H), 7.04 (s, 1H), 6.76 (d, *J* = 8.1 Hz, 1H), 6.67 (d, *J* = 8.1 Hz, 1H), 6.34 (s, 1H), 4.96 (d, *J* = 7.8 Hz, 1H), 3.93 (d, *J* = 5.0 Hz, 1H), 3.81–3.76 (m, 1H), 3.32–3.30 (m, 2H), 3.12–3.03 (m, 3H), 2.93–2.86 (m, 1H), 2.47–2.38 (m, 1H), 2.01 (dd, *J* = 25.7, 13.0 Hz, 1H), 1.87–1.79 (m, 1H), 1.67–1.61 (m, 1H), 1.48–1.42 (m, 2H), 1.12–1.06 (m, 1H), 0.72–0.64 (m, 1H), 0.62–0.59 (m, 1H), 0.55–0.53 (m, 1H), 0.44–0.38 (m, 1H). ^13^C NMR (100 MHz, DMSO-*d*_6_) δ: 161.69, 142.53, 141.91, 140.01, 135.28, 132.65, 130.11, 121.13, 119.88, 118.95, 118.43, 115.87, 108.24, 90.17, 70.17, 62.06, 57.16, 52.13, 46.97, 46.15, 29.90, 27.79, 24.11, 23.51, 6.23, 5.63, 3.12. HRMS *m*/*z*: calc. 487.2345 for C_28_H_31_N_4_O_4_ [M + H]^+^; obs.: 487.2319 [M + H]^+^. The purity of the compound was checked by HPLC (Rt= 2.300 min) and was found to be 99.19% pure.

#### 17-Cyclopropylmethyl-3,14β-dihydro-4,5α-epoxy-6α-[1H-pyrrolo­[2,3-*b*]­pyridine-5-carboxamide]­morphinan Hydrochloride (25)

Compound **25** was synthesized as shown in the general procedure with 78% yield. ^1^H NMR (400 MHz, DMSO-*d*_6_) δ: 12.12 (s, 1H), 8.91 (s, 1H), 8.80 (d, *J* = 2.0 Hz, 1H), 8.58 (d, *J* = 2.0 Hz, 1H), 8.21 (d, *J* = 7.6 Hz, 1H), 7.62–7.59 (m, 1H), 6.73 (d, *J* = 8.1 Hz, 1H), 6.63 (dd, *J* = 3.4, 1.7 Hz, 1H), 6.58 (d, *J* = 8.1 Hz, 1H), 4.80 (d, *J* = 3.9 Hz, 1H), 4.69–4.61 (m, 1H), 3.97 (d, *J* = 6.7 Hz, 1H), 3.41–3.26 (m, 2H), 3.13–2.97 (m, 3H), 2.77–2.69 (m, 1H), 2.59–2.51 (m, 1H), 1.99–1.91 (m, 1H), 1.65 (d, *J* = 10.8 Hz, 1H), 1.58–1.43 (m, 2H), 1.28–1.17 (m, 1H), 1.13–1.06 (m, 1H), 0.72–0.67 (m, 1H), 0.65–0.62 (m, 1H), 0.52–0.49 (m, 1H), 0.42–0.40 (m, 1H). ^13^C NMR (100 MHz, DMSO-*d*_6_) δ: 165.95, 148.77, 146.59, 142.16, 139.30, 129.23, 128.48, 122.69, 122.59, 119.80, 119.57, 118.79, 101.66, 87.73, 69.89, 61.49, 57.48, 57.12, 46.46, 45.70, 30.72, 29.70, 24.00, 19.88, 6.20, 5.68, 3.05. HRMS *m*/*z*: calc. 487.2345 for C_28_H_31_N_4_O_4_ [M + H]^+^; obs.: 487.2326 [M + H]^+^. The purity of the compound was checked by HPLC (Rt= 2.417 min) and was found to be 99.84% pure.

#### 17-Cyclopropylmethyl-3,14β-dihydro-4,5α-epoxy-6β-[1H-pyrrolo­[2,3-*b*]­pyridine-5-carboxamide]­morphinan Hydrochloride (26)

Compound **26** was synthesized as shown in the general procedure with 72% yield. ^1^H NMR (400 MHz, DMSO-*d*_6_) δ: 12.15 (s, 1H), 8.91 (s, 1H), 8.82 (d, *J* = 5.4 Hz, 1H), 8.81 (s, 1H), 8.60 (d, *J* = 1.8 Hz, 1H), 7.61 (dd, *J* = 3.4, 5.4 Hz, 1H), 6.75 (d, *J* = 8.1 Hz, 1H), 6.66 (d, *J* = 8.1 Hz, 1H), 6.63 (dd, *J* = 3.4, 1.7 Hz, 1H), 4.88 (d, *J* = 7.8 Hz, 1H), 3.89 (d, *J* = 5.0 Hz, 1H), 3.75–3.71 (m, 1H), 3.38–3.29 (m, 2H), 3.12–3.03 (m, 2H), 2.90–2.86 (m, 1H), 2.47–2.43 (m, 2H), 1.97–1.88 (m, 1H), 1.81 (d, *J* = 13.8 Hz, 1H), 1.6–1.60 (m, 1H), 1.50–1.39 (m, 2H), 1.11–1.08 (m, 1H), 0.72–0.65 (m, 1H), 0.64–0.58 (m, 1H), 0.54–0.51 (m, 1H), 0.44–0.41 (m, 1H). ^13^C NMR (100 MHz, DMSO-*d*_6_) δ: 165.59, 148.57, 142.65, 141.80, 141.78, 130.19, 129.11, 128.57, 122.49, 121.11, 120.01, 119.75, 118.99, 118.39, 101.74, 90.40, 70.23, 62.13, 57.15, 51.68, 46.99, 46.10, 29.87, 27.84, 24.36, 23.52, 6.23, 5.62, 3.12. HRMS *m*/*z*: calc. 487.2345 for C_28_H_31_N_4_O_4_ [M + H]^+^; obs.: 487.2317 [M + H]^+^. The purity of the compound was checked by HPLC (Rt= 2.415 min) and was found to be 99.67% pure.

#### 17-Cyclopropylmethyl-3,14β-dihydro-4,5α-epoxy-6α-[1H-indazole-4-carboxamide]­morphinan Hydrochloride (27)

Compound **27** was synthesized as shown in the general procedure with 64% yield. ^1^H NMR (400 MHz, DMSO-*d*_6_) δ: 8.90 (s, 1H), 8.39 (d, *J* = 0.8 Hz, 1H), 8.10 (d, *J* = 7.7 Hz, 1H), 7.73 (d, *J* = 8.3 Hz, 1H), 7.62 (d, *J* = 6.9 Hz, 1H), 7.43 (dd, *J* = 8.3, 6.9 Hz, 1H), 6.72 (d, *J* = 8.1 Hz, 1H), 6.58 (d, *J* = 8.1 Hz, 1H), 4.85 (d, *J* = 3.8 Hz, 1H), 4.67 (m, 1H), 3.96 (d, *J* = 6.7 Hz, 1H), 3.35 (m, 1H), 3.26 (m, 1H), 3.10 (m, 1H), 3.05 (m, 1H), 2.98 (m, 1H), 2.73 (m, 1H), 2.54 (m, 1H), 1.96 (m, 1H), 1.67 (m, 1H), 1.56 (m, 1H), 1.47 (m, 1H), 1.17 (m, 1H), 1.07 (m, 1H), 0.69 (m, 1H), 0.62 (m, 1H), 0.50 (m, 1H), 0.41 (m, 1H). ^13^C NMR (100 MHz, DMSO-*d*_6_) δ: 166.05, 146.09, 140.35, 138.87, 133.55, 128.77, 127.38, 125.25, 122.12, 120.68, 120.06, 119.14, 118.30, 113.30, 87.24, 69.37, 61.03, 57.03, 48.56, 45.88, 45.25, 30.22, 29.29, 23.52, 19.46, 5.69, 5.17, 2.56. HRMS *m*/*z*: calc. 487.2345 for C_28_H_31_N_4_O_4_ [M + H]^+^; obs.: 487.2345. The purity of the compound was checked by HPLC (Rt= 2.462 min) and was found to be 99.63% pure.

#### 17-Cyclopropylmethyl-3,14β-dihydro-4,5α-epoxy-6β-[1H-indazole-4-carboxamide]­morphinan Hydrochloride (28)

Compound **28** was synthesized as shown in the general procedure with 64% yield. ^1^H NMR (400 MHz, DMSO-*d*_6_) δ: 8.89 (s, 1H), 8.73 (d, *J* = 8.1 Hz, 1H), 8.38 (d, *J* = 0.9 Hz, 1H), 7.72 (d, *J* = 8.2 Hz, 1H), 7.67 (d, *J* = 7.1 Hz, 1H), 7.44 (dd, *J* = 8.2 Hz, 7.1 Hz, 1H), 6.74 (d, *J* = 8.1 Hz, 1H), 6.67 (d, *J* = 8.1 Hz, 1H), 4.90 (d, *J* = 7.8 Hz, 1H), 3.90 (d, *J* = 5.1 Hz, 1H), 3.76 (m, 1H), 3.37 (m, 1H), 3.31 (m, 1H), 3.11 (m, 1H), 3.04 (m, 1H), 2.87 (m, 1H), 2.48–2.44 (m, 2H), 1.95 (m, 1H), 1.80 (m, 1H), 1.65 (m, 1H), 1.48 (m, 1H), 1.41 (m, 1H), 1.06 (m, 1H), 0.67 (m, 1H), 0.60 (m, 1H), 0.52 (m, 1H), 0.41 (m, 1H). ^13^C NMR (100 MHz, DMSO-*d*_6_) δ: 165.92, 142.22, 141.33, 140.42, 133.81, 129.73, 127.18, 125.24, 120.75, 120.61, 119.58, 119.25, 117.94, 113.39, 89.91, 69.78, 61.69, 56.70, 51.13, 46.51, 45.65, 29.47, 27.36, 23.79, 23.05, 5.73, 5.11, 2.63. HRMS *m*/*z*: calc. 487.2345 for C_28_H_31_N_4_O_4_ [M + H]^+^; obs.: 487.2331 [M + H]^+^. The purity of the compound was checked by HPLC (Rt= 2.455 min) and was found to be 99.81% pure.

#### 17-Cyclopropylmethyl-3,14β-dihydro-4,5α-epoxy-6α-[1H-benzo­[d]­imidazole-4-carboxamide]­morphinan Hydrochloride (29)

Compound **29** was synthesized as shown in the general procedure with 69% yield.^1^H NMR (400 MHz, DMSO-*d*_6_) δ: 9.20 (s, 2H), 8.92 (s, 1H), 8.15 (d, *J* = 7.5 Hz, 1H), 7.96 (d, *J* = 8.1 Hz, 1H), 7.57 (t, *J* = 7.8 Hz, 1H), 6.74 (d, *J* = 8.1 Hz, 1H), 6.59 (d, *J* = 8.1 Hz, 1H), 6.46 (s, 1H), 4.83 (d, *J* = 3.7 Hz, 1H), 4.79–4.71 (m, 1H), 3.98 (d, *J* = 6.7 Hz, 1H), 3.32–3.24 (m, 2H), 3.12–3.10 (m, 1H), 3.07–3.04 (m, 1H), 3.01–2.95 (m, 1H), 2.78–2.64 (m, 1H), 2.56–2.51 (m, 1H), 2.04–1.96 (m, 1H), 1.68–1.63 (m, 1H), 1.62–1.58 (m, 1H), 1.52–1.45 (m, 1H), 1.27–1.17 (m, 1H), 1.14–1.06 (m, 1H), 0.73–0.66 (m, 1H), 0.66–0.59 (m, 1H), 0.53–0.48 (m, 1H), 0.43–0.38 (m, 1H). ^13^C NMR (100 MHz, DMSO-*d*_6_) δ: 164.11, 146.05, 142.12, 138.93, 132.59, 128.72, 124.28, 123.96, 122.07, 121.25, 119.16, 118.29, 117.23, 117.12, 87.16, 69.49, 61.01, 57.04, 46.00, 45.28, 30.30, 29.16, 23.54, 19.47, 5.73, 5.21, 2.61. HRMS *m*/*z*: calc. 487.2345 for C_28_H_31_N_4_O_4_ [M + H]^+^; obs.: 487.2360 [M + H]^+^. The purity of the compound was checked by HPLC (Rt= 2.313 min) and was found to be 99.88% pure.

#### 17-Cyclopropylmethyl-3,14β-dihydro-4,5α-epoxy-6β-[1H-benzo­[d]­imidazole-4-carboxamide]­morphinan Hydrochloride (30)

Compound **30** was synthesized as shown in the general procedure with 35% yield. ^1^H NMR (400 MHz, DMSO-*d*_6_) δ: 9.51 (s, 1H, exchangeable), 9.31 (s, 1H), 8.92 (s, 1H), 8.15 (d, *J* = 7.3 Hz, 1H), 7.99 (d, *J* = 8.1 Hz, 1H), 7.62 (t, *J* = 7.8 Hz, 1H), 6.76 (d, *J* = 8.1 Hz, 1H), 6.67 (d, *J* = 8.1 Hz, 1H), 6.36 (s, 1H, exchangeable), 4.89 (d, *J* = 7.8 Hz, 1H), 3.93 (d, *J* = 4.7 Hz, 1H), 3.83–3.79 (m, 1H), 3.35–3.29 (m, 2H), 3.12–3.06 (m, 2H), 2.91–2.85 (m, 1H), 2.47–2.44 (m, 2H), 2.07–1.95 (m, 1H), 1.85–1.81 (m, 1H), 1.70–1.63 (m, 1H), 1.49–1.47 (m, 1H), 1.46–1.39 (m, 1H), 1.11–1.06 (m, 1H), 0.72–0.65 (m, 1H), 0.64–0.57 (m, 1H), 0.56–0.49 (m, 1H), 0.44–0.39 (m, 1H). ^13^C NMR (100 MHz, DMSO-*d*_6_) δ: 163.98, 142.08, 142.05, 141.37, 133.14, 132.72, 129.65, 124.68, 123.62, 120.90, 120.58, 119.36, 117.89, 117.58, 89.95, 69.75, 61.54, 56.70, 51.17, 46.48, 45.72, 29.51, 27.31, 23.78, 23.02, 5.73, 5.11, 2.64. HRMS *m*/*z*: calc. 487.2345 for C_28_H_31_N_4_O_4_ [M + H]^+^; obs.: 487.2334 [M + H]^+^.The purity of the compound was checked by HPLC (Rt= 2.305 min) and was found to be 99.54% pure.

#### 17-Cyclopropylmethyl-3,14β-dihydro-4,5α-epoxy-6α-[1H-pyrrolo­[3,2-*c*]­pyridine-4-carboxamide]­morphinan Hydrochloride (31)

Compound **31** was synthesized as shown in the general procedure with 77% yield. 1H NMR (400 MHz, DMSO-*d*_6_) δ: 12.57 (s, 1H), 9.32 (s, 1H), 8.92 (s, 1H), 8.82 (s, 1H), 8.35 (d, *J* = 6.0 Hz, 1H), 7.87 (s, 2H), 7.24 (s, 1H), 6.76 (d, *J* = 8.1 Hz, 1H), 6.60 (d, *J* = 8.1 Hz, 1H), 6.43 (s, 1H), 4.82 (d, *J* = 3.7 Hz, 1H), 4.75–4.68 (m, 1H), 3.98 (d, *J* = 6.6 Hz, 1H), 3.33–3.22 (m, 3H), 3.12–2.98 (m, 3H), 2.77–2.69 (m, 1H), 2.59–2.52 (m, 1H), 2.01–1.93 (m, 1H), 1.65 (dd, *J* = 20.3, 12.0 Hz, 2H), 1.48 (dd, *J* = 15.2, 9.8 Hz, 1H), 1.12–1.05 (m, 1H), 0.74–0.67 (m, 1H), 0.64–0.59 (m, 1H), 0.51–0.48 (m, 1H), 0.43–0.39 (m, 1H). ^13^C NMR (100 MHz, DMSO-*d*_6_) δ: 159.58, 145.83, 142.50, 138.97, 132.33, 128.73, 125.70, 122.79, 122.12, 119.38, 118.30, 110.04, 103.21, 87.31, 69.35, 64.89, 60.93, 57.05, 45.81, 45.36, 45.20, 30.19, 29.26, 23.51, 19.72, 5.70, 5.18, 2.58. HRMS *m*/*z*: calc. 487.2345 for C_28_H_31_N_4_O_4_ [M + H]^+^; obs.: 487.2345 [M + H]^+^. The purity of the compound was checked by HPLC (Rt= 2.302 min) and was found to be 98.84% pure.

#### 17-Cyclopropylmethyl-3,14β-dihydro-4,5α-epoxy-6β-[1H-pyrrolo­[3,2-*c*]­pyridine-4-carboxamide]­morphinan Hydrochloride (32)

Compound **32** was synthesized as shown in the general procedure with 75% yield. ^1^H NMR (400 MHz, DMSO-*d*_6_) δ: 12.71 (s, 1H), 9.35 (s, 1H), 8.89 (s, 1H), 8.36 (d, *J* = 6.1 Hz, 1H), 7.93 (s, 2H), 7.25 (s, 1H), 6.75 (d, *J* = 8.1 Hz, 1H), 6.68 (d, *J* = 8.1 Hz, 1H), 6.31 (s, 1H), 4.98 (d, *J* = 7.7 Hz, 1H), 3.90 (d, *J* = 5.1 Hz, 1H), 3.82–3.77 (m, 1H), 3.30–3.28 (m, 3H), 3.13–3.05 (m, 2H), 2.88–2.81 (m, 1H), 2.53–2.51 (m, 1H), 2.11–2.02 (m, 1H), 1.82–1.79 (m, 1H), 1.68–1.64 (m, 1H), 1.49–1.40 (m, 2H), 1.09–1.07 (m, 1H), 0.73–0.66 (m, 1H), 0.62–0.58 (m, 1H), 0.53–0.50 (m, 1H), 0.43–0.40 (m, 1H). ^13^C NMR (100 MHz, DMSO-*d*_6_) δ: 158.65, 142.11, 141.40, 129.65, 122.75, 122.48, 121.45, 120.65, 119.43, 119.39, 117.96, 110.23, 103.48, 89.74, 69.71, 61.59, 56.69, 51.57, 46.48, 45.70, 29.51, 27.31, 23.56, 23.00, 5.73, 5.11, 2.63. HRMS *m*/*z*: calc. 487.2345 for C_28_H_31_N_4_O_4_ [M + H]^+^; obs.: 487.2333 [M + H]^+^. The purity of the compound was checked by HPLC (Rt= 2.308 min) and was found to be 98.57% pure.

#### 17-Cyclopropylmethyl-3,14β-dihydro-4,5α-epoxy-6α-[1H-pyrrolo­[2,3-*c*]­pyridine-4-carboxamide]­morphinan Hydrochloride (33)

Compound **33** was synthesized as shown in the general procedure with 74% yield. ^1^H NMR (400 MHz, DMSO-*d*_6_) δ: 13.31 (s, 1H), 9.26 (s, 1H), 9.24 (s, 1H), 8.92 (s, 1H), 8.74 (s, 1H), 8.72 (d, *J* = 7.8 Hz, 1H), 8.39 (s, 1H), 7.24 (s, 1H), 6.74 (d, *J* = 8.1 Hz, 1H), 6.59 (d, *J* = 8.1 Hz, 1H), 6.43 (s, 1H), 4.83 (d, *J* = 3.6 Hz, 1H), 4.71–4.66 (m, 1H), 3.98 (d, *J* = 6.6 Hz, 1H), 3.28–3.22 (m, 2H), 3.12–3.06 (m, 2H), 3.01–2.96 (m, 1H), 2.78–2.69 (t, *J* = 12.4 Hz, 1H), 2.57–2.53 (m, 1H), 2.01–1.94 (m, 1H), 1.67 (d, *J* = 11.8 Hz, 1H), 1.61–1.56 (m, 1H), 1.52–1.46 (m, 1H), 1.25–1.16 (m, 1H), 1.11–1.07 (m, 1H), 0.71–0.69 (m, 1H), 0.64–0.60 (m, 1H), 0.52–0.46 (m, 1H), 0.42–0.39 (m, 1H). ^13^C NMR (100 MHz, DMSO-*d*_6_) δ: 163.71, 146.60, 140.46, 139.36, 135.27, 132.34, 129.19, 128.72, 123.49, 122.64, 119.66, 118.90, 104.33, 87.41, 69.86, 61.44, 57.49, 46.68, 45.76, 30.68, 29.68, 24.00, 19.75, 6.19, 5.67, 3.06. HRMS *m*/*z*: calc. 487.2345 for C_28_H_31_N_4_O_4_ [M + H]^+^; obs.: 487.2320 [M + H]^+^. The purity of the compound was checked by HPLC (Rt= 2.287 min) and was found to be 99.40% pure.

#### 17-Cyclopropylmethyl-3,14β-dihydro-4,5α-epoxy-6β-[1H-pyrrolo­[2,3-*c*]­pyridine-4-carboxamide]­morphinan Hydrochloride (34)

Compound **34** was synthesized as shown in the general procedure with 68% yield.^1^H NMR (400 MHz, DMSO-*d*_6_) δ: 12.72 (s, 1H), 9.63 (d, *J* = 8.0 Hz, 1H), 9.42 (s, 2H), 9.19 (s, 1H), 8.94 (s, 1H), 7.85 (m, 1H), 7.10 (m, 1H), 6.78 (d, *J* = 8.8 Hz, 1H), 6.67 (d, *J* = 8.2 Hz, 1H), 6.35 (s, 1H), 4.93 (d, *J* = 8.0 Hz, 1H), 3.94 (d, *J* = 5.3 Hz, 1H), 3.80–3.72 (m, 1H), 3.12–3.07 (m, 2H), 2.90–2.89 (m, 1H), 2.04–2.01 (m, 1H), 1.89–1.85 (m, 1H), 1.68–1.65 (m, 1H), 1.50–1.40 (m, 2H), 1.09–1.07 (m, 1H), 0.68–0.62 (m, 1H), 0.61–0.54 (m, 1H), 0.53–0.51 (m, 1H), 0.43–0.40 (m, 1H). ^13^C NMR (100 MHz, DMSO-*d*_6_) δ: 165.39, 146.15, 142.35, 138.83, 133.31, 131.70, 131.37, 128.74, 125.01, 122.14, 119.12, 118.39, 114.38, 114.19, 87.09, 69.41, 64.89, 61.02, 57.02, 46.31, 45.24, 30.26, 29.21, 23.53, 19.29, 5.72, 5.20, 2.58. HRMS *m*/*z*: calc. 487.2345 for C_28_H_31_N_4_O_4_ [M + H]^+^; obs.: 487.2333 [M + H]^+^. The purity of the compound was checked by HPLC (Rt= 2.310 min) and was found to be 99.88% pure.

#### 17-Cyclopropylmethyl-3,14β-dihydro-4,5α-epoxy-6α-[1H-pyrrolo­[2,3-*b*]­pyridine-4-carboxamide]­morphinan Hydrochloride (35)

Compound **35** was synthesized as shown in the general procedure with 85% yield. ^1^H NMR (400 MHz, DMSO-*d*_6_) δ 12.05 (s, 1H), 8.90 (s, 1H), 8.36 (d, *J* = 5.0 Hz, 1H), 8.18 (d, *J* = 7.8 Hz, 1H), 7.63 (t, *J* = 3.1 Hz, 1H), 7.45 (d, *J* = 5.0 Hz, 1H), 6.83 (dd, *J* = 3.1, 1.8 Hz, 1H), 6.73 (d, *J* = 8.1 Hz, 1H), 6.58 (d, *J* = 8.1 Hz, 1H), 4.84 (d, *J* = 3.9 Hz, 1H), 4.68–4.63 (m, 1H), 3.96 (d, *J* = 6.7 Hz, 1H), 3.38–3.25 (m, 2H), 3.10–3.04 (m, 2H), 2.99–2.95 (m, 1H), 2.78–2.68 (m, 1H), 2.58–2.53 (m, 1H), 2.02–1.91 (m, 1H), 1.67 (d, *J* = 11.0 Hz, 1H), 1.59–1.54 (m, 1H), 1.47 (dd, *J* = 15.2, 9.8 Hz, 1H), 1.18–1.11 (m, 2H), 0.73–0.66 (m, 1H), 0.65–0.58 (m, 1H), 0.53–0.50 (m, 1H), 0.43–0.41 (m, 1H). ^13^C NMR (100 MHz, DMSO-*d*_6_) δ: 166.18, 149.07, 146.51, 141.87, 139.38, 134.77, 129.24, 128.47, 122.61, 119.68, 118.79, 118.19, 114.08, 100.81, 87.60, 69.83, 61.47, 57.48, 49.05, 46.40, 45.75, 30.67, 29.75, 23.99, 19.93, 6.18, 5.66, 3.05. HRMS *m*/*z*: calc. 487.2345 for C_28_H_31_N_4_O_4_ [M + H]^+^; obs.: 487.2316 [M + H]^+^. The purity of the compound was checked by HPLC (Rt= 2.397 min) and was found to be 99.69% pure.

#### 17-Cyclopropylmethyl-3,14β-dihydro-4,5α-epoxy-6β-[1H-pyrrolo­[2,3-*b*]­pyridine-4-carboxamide]­morphinan Hydrochloride (36)

Compound **36** was synthesized as shown in the general procedure with 69% yield. ^1^H NMR (400 MHz, DMSO-*d*_6_) δ: 11.98 (s, 1H), 8.89 (s, 1H), 8.79 (d, *J* = 8.1 Hz, 1H), 8.35 (d, *J* = 5.0 Hz, 1H), 7.65–7.58 (m, 1H), 7.48 (d, *J* = 5.0 Hz, 1H), 6.84 (dd, *J* = 3.2, 1.9 Hz, 1H), 6.74 (d, *J* = 8.1 Hz, 1H), 6.67 (d, *J* = 8.1 Hz, 1H), 4.88 (d, *J* = 7.8 Hz, 1H), 3.89 (d, *J* = 5.0 Hz, 1H), 3.80–3.72 (m, 1H), 3.38–3.30 (m, 2H), 3.12–3.05 (m, 2H), 2.91–2.84 (m, 1H), 2.47–2.43 (m, 2H), 1.96 (dd, *J* = 24.8, 12.9 Hz, 1H), 1.80 (d, *J* = 13.6 Hz, 1H), 1.69–1.60 (m, 1H), 1.50–1.39 (m, 2H), 1.09–1.04 (m, 1H), 0.72–0.62 (m, 1H), 0.62–0.58 (m, 1H), 0.55–0.49 (m, 1H), 0.44–0.40 (m, 1H). ^13^C NMR (100 MHz, DMSO-*d*_6_) δ: 166.42, 149.65, 142.61, 142.31, 141.76, 134.04, 130.13, 128.28, 121.11, 119.86, 118.44, 118.03, 113.58, 101.09, 90.23, 70.21, 62.20, 57.18, 51.65, 46.95, 46.14, 29.95, 27.79, 24.12, 23.45, 6.16, 5.62, 3.04. *HRMS* *m*/*z*: calc. 487.2345 for C_28_H_31_N_4_O_4_ [M + H]^+^; obs.: 487.2354 [M + H]^+^. The purity of the compound was checked by HPLC (Rt= 2.393 min) and was found to be 99.79% pure.

### Biological Evaluation of Drugs

Morphine (morphine sulfate pentahydrate) was obtained from Mallinckrodt (St. Louis, MO) or provided by the National Institute on Drug Abuse (NIDA). Naltrexone and naloxone hydrochloride salts were purchased from Sigma-Aldrich (St. Louis, MO). Test compounds and reference drugs were dissolved in pyrogen-free isotonic saline (Baxter Healthcare, Deerfield, IL) or sterile-filtered distilled/deionized water. All other reagents and radioligands were obtained from Sigma-Aldrich or Thermo Fisher.

### Animals

Male Swiss Webster mice (25–35 g, 6–8 weeks old; Harlan Laboratories, Indianapolis, IN) were housed in a temperature-controlled (20–22 °C) AAALAC-accredited facility with ad libitum access to food and water. Mice were maintained on a 12 h/12 h light–dark cycle (lights on 06:00–18:00) and tested during the light phase. Upon arrival, mice were housed 5 per cage and acclimated for 1 week before being individually housed for at least 24 h prior to experiments. Animals were randomly assigned to treatment groups, and experimenters were blinded to group assignments. No adverse events occurred, and all animals were included in data analysis. All procedures were approved by the Institutional Animal Care and Use Committee (IACUC, Animal Welfare Assurance Number D16–00180) at Virginia Commonwealth University Medical Center and were conducted in accordance with the recommendations of the International Association for the Study of Pain (IASP).

### In Vitro Competitive Radioligand Binding Assay

Competition binding assays were performed using CHO cells expressing monoclonal mouse opioid receptors (MOR, KOR) or human δ-opioid receptor (DOR), kindly provided by Dr. Selley (Virginia Commonwealth University). Membrane preparations (20–30 μg protein) were incubated with the corresponding radioligand and varying concentrations of test compounds in TME buffer (50 mM Tris, 3 mM MgCl_2_, 0.2 mM EGTA, pH 7.7) for 1.5 h at 30 °C. Bound radioligand was separated by filtration using a Brandel harvester. Specific receptor binding was defined as the difference in binding in the absence and presence of selective antagonists (5 μM naltrexone, U50,488, and SNC80 for MOR, KOR, and DOR, respectively). Competition binding data were expressed as % bound = (specific binding in the presence of competitor/specific binding in the absence of competitor) × 100%.

### In Vitro [^35^S]-GTPγS Functional Assay

Functional activity at MOR was determined using [^35^S]-GTPγS binding. Membrane protein (10 μg) was incubated in 500 μL TME buffer containing 100 mM NaCl, 20 μM GDP, 0.1 nM [^35^S]-GTPγS, and varying concentrations of test compounds for 1.5 h at 30 °C. Protein concentrations were determined and adjusted using the Bradford assay. Nonspecific binding was measured in the presence of 20 μM unlabeled GTPγS, and 3 μM DAMGO was included as a maximal agonist control. Following incubation, bound radioligand was separated by filtration through GF/B glass fiber filters and washed three times with ice-cold buffer (50 mM Tris–HCl, pH 7.2) using a Brandel harvester. Radioactivity was quantified by liquid scintillation counting. Net-stimulated binding was defined as agonist-stimulated minus basal binding. Percent of DAMGO-stimulated binding was calculated as (net binding by ligand/net binding by 3 μM DAMGO) × 100%.

### Data Analysis of Receptor Binding and [^35^S]­GTPyS Functional Assay

All assays were performed in duplicate and repeated at least three times (≥3 independent experiments). Results are reported as mean ± SEM. Concentration–response curves were fitted by nonlinear regression using a four-parameter model in GraphPad Prism (minimum constrained to 0) to determine EC_5__0_, *E*_max_, and Hill coefficients. IC_5__0_ values were obtained from nonlinear regression with the maximum constrained to 100% and minimum to 0. By using the Cheng–Prusoff equation K_i_ = IC_50_/[1 + ([L]/K_D_)], where [L] is the concentration of the competitor and K_D_ is the K_D_ of the radioligand; binding K_i_ values were determined from IC_50_ values.

### Warm-Water Tail Immersion Assay

Antinociceptive activity of the synthesized compounds was evaluated using the warm-water tail immersion assay in male Swiss Webster mice (n = 6 per group, 25–35 g, 6–8 weeks old). The water bath temperature was maintained at 56 ± 0.1 °C. Baseline tail-flick latency was measured prior to compound administration, and only mice with a baseline of 2–4 s were included. For agonist studies, compounds were administered subcutaneously (s.c.), and tail immersion was performed 20 min postdose, corresponding to the peak effect of morphine. A cutoff time of 10 s was imposed to prevent tissue damage. Antinociceptive response was calculated as the percentage of the maximum possible effect (%MPE) using [(test – control latency)/(10 – control latency)] × 100.

For antagonist studies, test compounds (s.c.) were administered 5 min prior to morphine (10 mg/kg, s.c.), and tail immersion was conducted 20 min after morphine administration. AD_5__0_ values were determined by least-squares linear regression, and 95% confidence intervals were calculated using the Bliss method.

### Opioid-Withdrawal Studies

Opioid withdrawal was assessed in male Swiss Webster mice (n = 6 per group, 25–35 g, 6–8 weeks old) using a previously reported protocol. A 75 mg morphine pellet was implanted subcutaneously in the back of each mouse, and animals were allowed to recover in their home cages. Prior to testing, mice were habituated for 30 min in an open-topped Plexiglas chamber (26 × 26 × 26 cm^3^) divided into quadrants. Test compounds and reference drugs were administered s.c., and withdrawal was precipitated 72 h after pellet implantation using naloxone (1 mg/kg, s.c.) or varying doses of test compounds. Withdrawal behaviors–including escape jumps, paw tremors, and wet dog shakes–were recorded for 20 min for each mouse. Data are presented as mean ± SEM.

### Calcium Flux

Dr. Arnatt from Saint Louis University kindly provided cDNA used to enable expression of G_qi4_. The hMOR-CHO cells were cultured. Following 24 h of Gα_qi4_ transfection, the cells were seeded at 20,000 cells per well in a clear-bottom black-walled 96-well plate (Greiner Bio-One) and allowed to incubate for 24 h. The growth medium was then removed, and the wells were rinsed with a 50:1 mixture of HBSS:HEPES assay buffer. Cells were subsequently incubated with Fluo4 loading buffer (comprising 40 μL of 2 μM Fluo4-AM (Invitrogen), 84 μL of 2.5 mM probenecid, and 8 mL of assay buffer) for 60 min. For antagonism assays, different concentrations of test compounds were added in triplicate, and the plate was incubated for an additional 15 min. The plates were then analyzed using a FlexStation3 microplate reader (Molecular Devices) at 494/516 nm (ex/em) for a total duration of 120 s. For agonism assays, after 15 s of reading, varying concentrations of test compounds in triplicate or 500 nM DAMGO (used in antagonism studies), or assay buffer alone (control), were added. Calcium flux changes were monitored, and peak height values were recorded. The data were subjected to nonlinear regression analysis to determine EC_50_ or IC_50_ values using GraphPad Prism 6.0 (GraphPad Software, San Diego, CA).

### Molecular Modeling

Docking studies were performed to investigate the binding mode of compound **7** (Figure S2A) with the inactive mu-opioid receptor (MOR) crystal structure and to provide mechanistic insights into ligand–receptor interactions. Compound **7** was constructed in Sybyl X2.1, assigned Gasteiger–Hückel charges, and energy-minimized for 10,000 iterations to a gradient of 0.05 using the Tripos force field. The X-ray crystal structure of antagonist-bound MOR (PDB ID: 4DKL) was obtained from the Protein Data Bank and prepared for docking by adding hydrogens, removing water molecules and cocrystallized ligands, and modeling missing residues in ICL-3 using Sybyl 8.0 (Tripos, MO, USA).

Docking was performed using the GOLD 2020 genetic algorithm. The binding site was defined as atoms within 10 Å of the γ-carbon of D147, with a distance constraint applied between the protonated 17-amino nitrogen of the ligand and the carboxylate of D147, reflecting the canonical ionic interaction for epoxymorphinan structures. A hydrogen-bond constraint was also applied between the ligand’s dihydrofuran oxygen and the phenolic oxygen of Tyr148. The top-ranked CHEM-PLP poses were visualized in PyMOL and selected for subsequent molecular dynamics (MD) simulations.

### Molecular Dynamics Simulations

MD simulations were carried out using the Amber 2020 package. The membrane system was constructed using CHARMM-GUI, incorporating POPC lipids, a TIP3P water box, and 0.15 M NaCl in complex with the protein–ligand system (Figure S1B). Simulations were performed for 200 ns under NPT conditions (P = 1 atm, T = 310 K) with periodic boundary conditions. Temperature was maintained using the Langevin thermostat, and long-range electrostatics were calculated via the Particle Mesh Ewald (PME) method. Nonbonded van der Waals interactions were truncated at 10 Å. MD trajectories were analyzed using Visual Molecular Dynamics (VMD) software to evaluate the stability and conformational dynamics of the ligand–receptor complex.

### UPLC-MS/MS Analysis

The identification and quantification of compound **7** in mouse plasma and brain were performed using a modification of a previously described method with naloxone-*d*_5_ as the internal standard.^2^ Prior to extraction, brain tissues were homogenized with deionized water at a 1:3 (w/w) ratio using an Omni Bead Ruptor (Omni International Inc., Kennesaw, GA). Each analytical run included seven-point calibration curves (10–1000 ng/mL or ng/g) for VZMN424, quality control samples at 30, 300, and 750 ng/mL or ng/g, as well as negative and blank controls, all prepared in plasma or brain homogenate. After mixing, 100 μL of 5 M ammonium hydroxide and 2 mL of a 25:75 methylene chloride:diethyl ether mixture were added. Samples were vortexed for 2 min and centrifuged at 3000 rpm for 5 min. The organic layer was evaporated under nitrogen and reconstituted with 100 μL of mobile phase before LC-MS/MS analysis. Chromatography was performed on a Sciex ExionLC 2.0+ system coupled to a Sciex 6500 QTRAP with an IonDrive Turbo V source (Sciex, Ontario, Canada), using a Zorbax Eclipse column (4.6 × 75 mm, 3.5 μm; Agilent, USA) and an isocratic mobile phase of 10 mM ammonium formate:methanol (50:50, v/v) at 0.6 mL/min. Source conditions included a temperature of 600 °C, curtain gas at 30 mL/min, ion spray voltage of 5000 V, and ion source gases 1 and 2 at 50 and 30 mL/min, respectively. Data were acquired in positive-ion mode using multiple reaction monitoring (MRM) with the following transitions (*m*/*z*), and collision energy (eV) in parentheses: **7**, 487 > 469 (29) and 487 > 267 (46). Total run time was 4 min. Quantification was performed using linear regression of analyte-to-ISTD peak area ratios from the calibration curves.

### Statistical Analysis

One-way ANOVA followed by the posthoc Dunnett test were performed to assess the significance using GraphPad Prism software (GraphPad Software, San Diego, CA).

## Supplementary Material







## Notes

Spectra data for target compounds (^1^H NMR, ^13^C NMR, mass spectra, and HPLC graphs) (CSV)
ACD/Percepta (v2020.2.0) predicted physiochemical properties (PDF)
Molecular modeling figures (PDB)

## Abbreviations Used

cAMP: Cyclic adenosine monophosphate
CHO: Chinese hamster ovary
CL: Confidence level
DAMGO: [d-Ala2-MePhe4-Gly­(ol)­5]­enkephalin
DOR: δ opioid receptor
EDCI: 1-Ethyl-3- (3-(dimethylamino)­propyl)­carbodiimide
GPCR: G protein-coupled receptor
HOBt: Hydroxybenzotriazole
KOR: κ opioid receptor
MD: Molecular dynamics
MOR: μ opioid receptor
NAN: 17-Cyclopropylmethyl-3,14β dihydroxy-4,5α-epoxy-6α-(indole-7-carboxamido)­morphinan
NIDA: National Institute on Drug Abuse
% MPE: Percentage maximum possible effect
