# Supplementary material for: Investigating the Landscape of C6-Azaindole Side Chain on the Epoxymorphinan Skeleton via the Nitrogen Walk Concept: A Strategy to Enhance Drug-Like Properties
Source: J Med Chem. 2026 Jan 22;69(3):2330–48. doi: 10.1021/acs.jmedchem.5c02175 (PMC12910667; doi:10.1021/acs.jmedchem.5c02175)
Supplement: Supplementary file 2 [file jm5c02175_si_002.pdf]

Supporting Information

Investigating the Landscape of C6-Azaindole Sidechain on the Epoxymorphinan Skeleton via the Nitrogen Walk Concept: A Strategy to Enhance Drug-Like Properties

Logan Neel[a,b], Hongguang Ma[a], Ahmed Reda[a], Mengchu Li[a], Rachael Flammia[a], Samuel Woodard[b], James C. Gillespie[b], Dana E. Selley[b], William L. Dewey[b], Piyusha P. Pagare[a], Yan Zhang[a,b,c,d,*]

[a] Department of Medicinal Chemistry, School of Pharmacy, Virginia Commonwealth University, 800 E Leigh Street, Richmond, Virginia 23298, United States

[b] Department of Pharmacology and Toxicology, Virginia Commonwealth University, 410 North 12th Street, Richmond, Virginia 23298, United States

[c] Center for Drug Discovery, Virginia Commonwealth University, 800 E Leigh Street, Richmond, VA 23298, United States

[d] Institute for Drug and Alcohol Studies, Virginia Commonwealth University, 203 East Cary Street, Richmond, VA 23298, United States

* Corresponding author. Tel.: +1 804 8280021. E-mail address: yzhang2@vcu.edu (Y. Zhang).

Table of contents

1. ^{1}H NMR, ^{13}C NMR, and mass spectra of final compounds (S3 to S73)
2. **Table S1**. Purity data of final compounds (S74 to S75)
3. HPLC chromatograms of final compounds (S76 to S93)
4. **Table S2**. Predicted physiochemical properties (S94)
5. **Figure S1**, **Figure S2**, and **Table S3**. Molecular Modeling (S95 to S96)

1.) ^{1}H NMR, ^{13}C NMR, and mass spectra of final compounds.

17-Cyclopropylmethyl-3,14β-dihydro-4,5α-epoxy-6α-[1H-indazole-7-carboxamide]morphinan Hydrochloride (**1**)

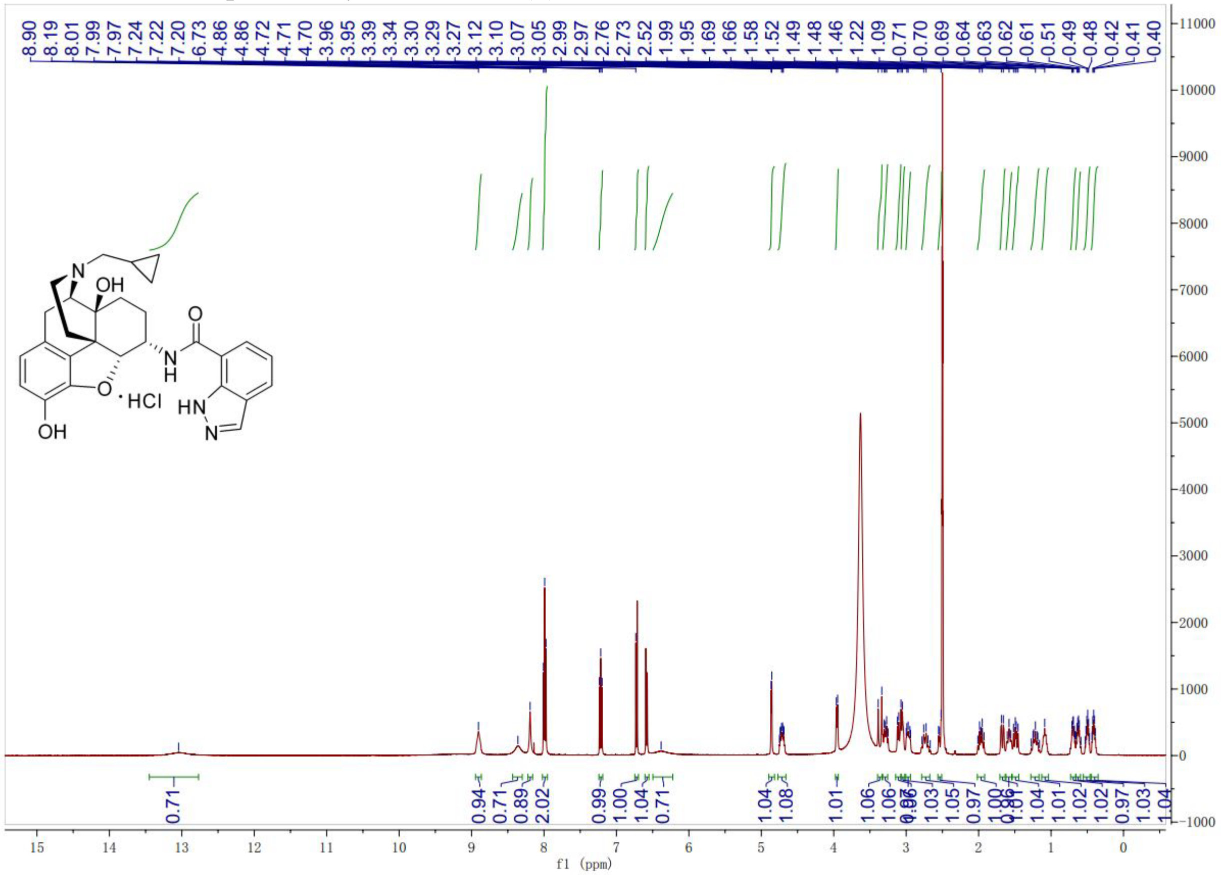

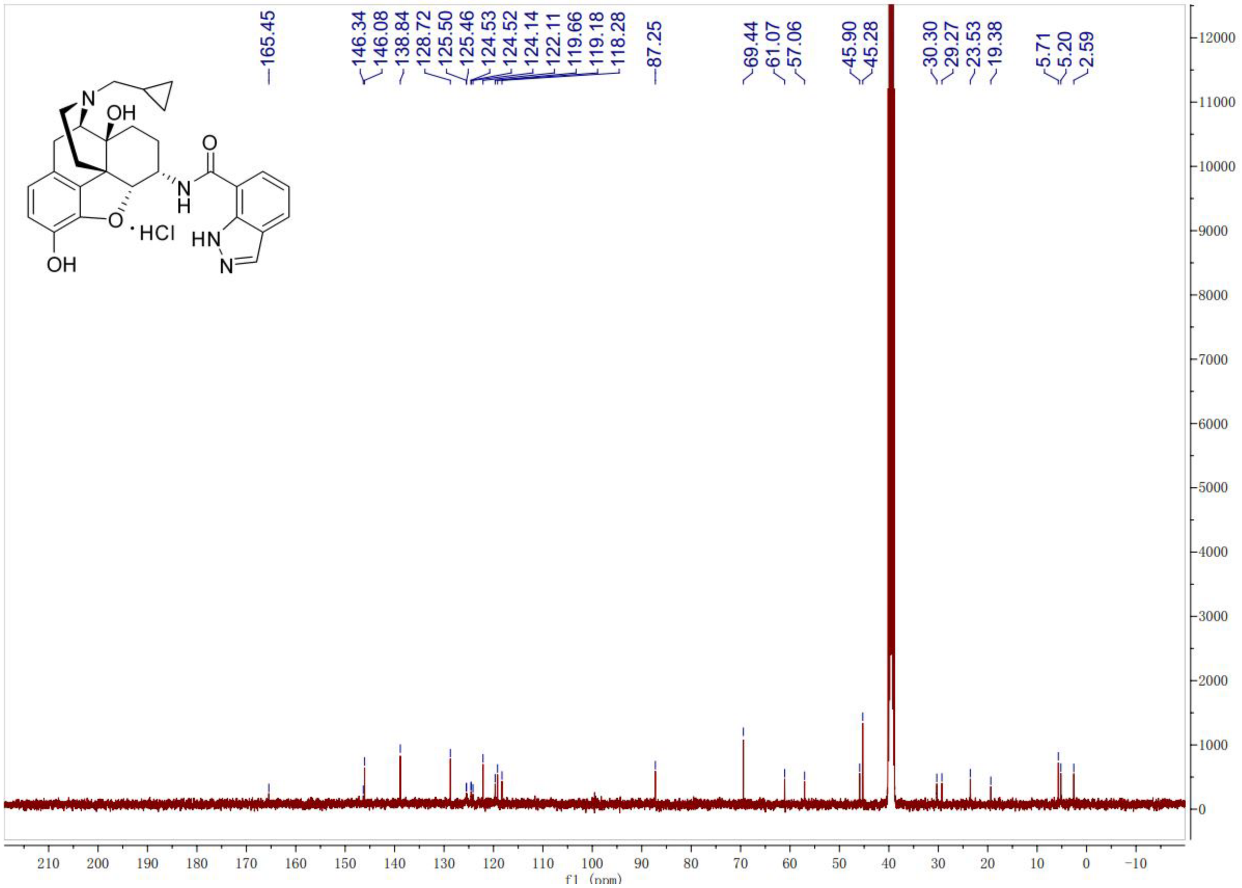
165.45
146.34
146.08
138.84
128.72
125.50
125.46
124.53
124.52
124.14
122.11
119.66
119.18
118.28
87.25
69.44
61.07
57.06
45.90
45.28
30.30
29.27
23.53
19.38
5.71
5.20
2.59
OH
O
N
H
O
HCl
HN
N
OH
f1 (ppm)

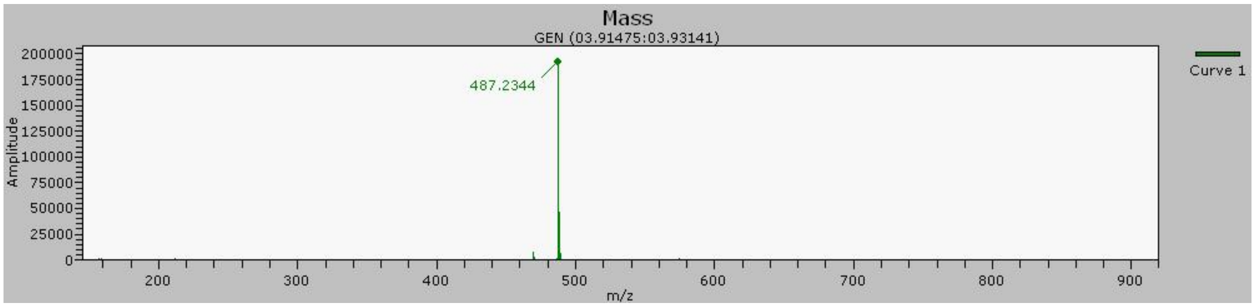
Mass
GEN (03.91475:03.93141)
487.2344
Amplitude
m/z
Curve 1

17-Cyclopropylmethyl-3,14β-dihydro-4,5α-epoxy-6β-[1H-indazole-7-carboxamide]morphinan Hydrochloride (**2**)

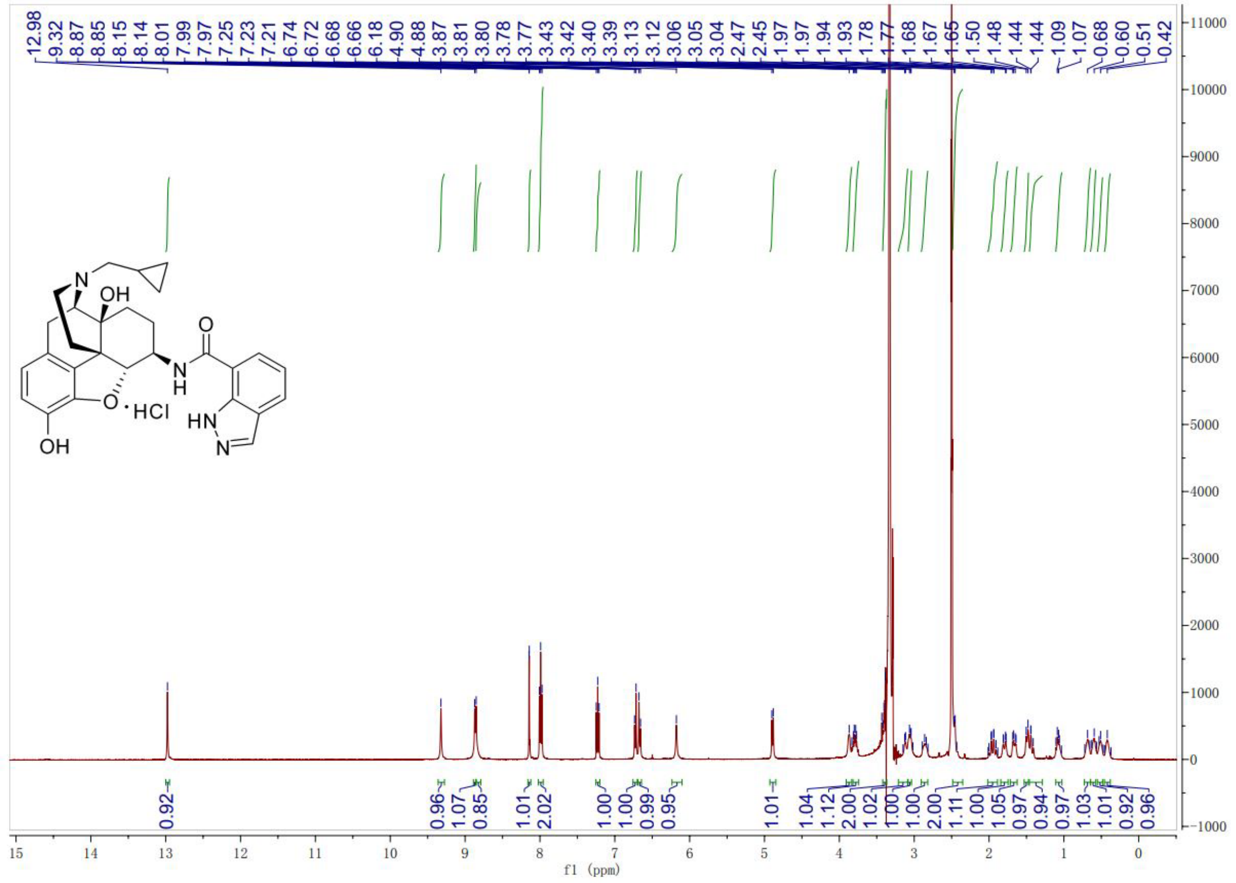

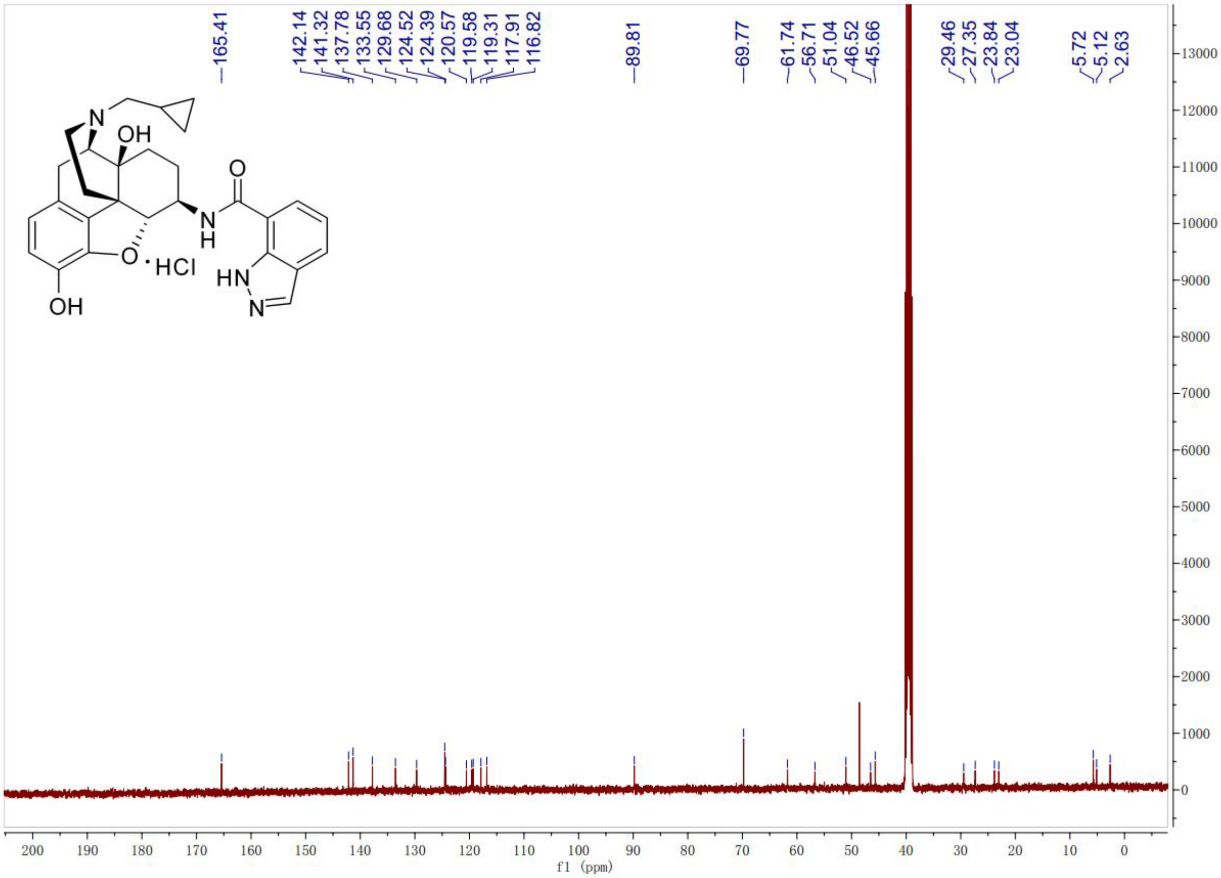
165.41
142.14
141.32
137.78
133.55
129.68
124.52
124.39
120.57
119.58
119.31
117.91
116.82
89.81
69.77
61.74
56.71
51.04
46.52
45.66
29.46
27.35
23.84
23.04
5.72
5.12
2.63
N
OH
O
N
H
O
· HCl
HN
N
OH
f1 (ppm)

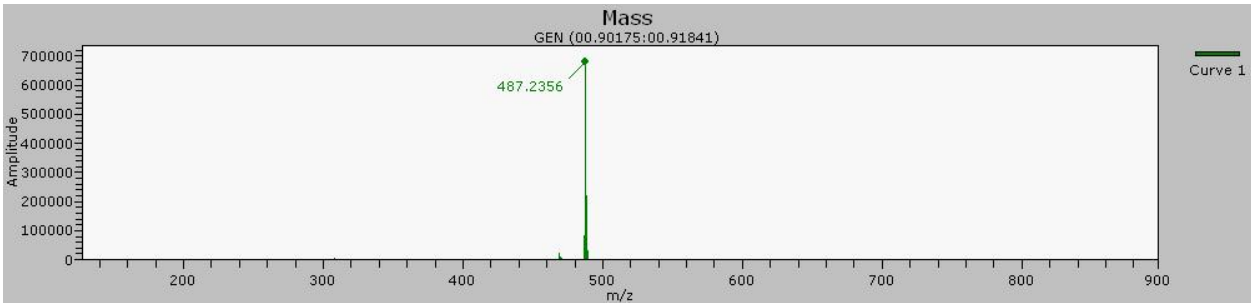
Mass
GEN (00.90175:00.91841)
487.2356
Curve 1
Amplitude
m/z

17-Cyclopropylmethyl-3,14β-dihydro-4,5α-epoxy-6α-[1H-pyrrolo[3,2-b]pyridine-7-carboxamide]morphinan Hydrochloride (**3**)

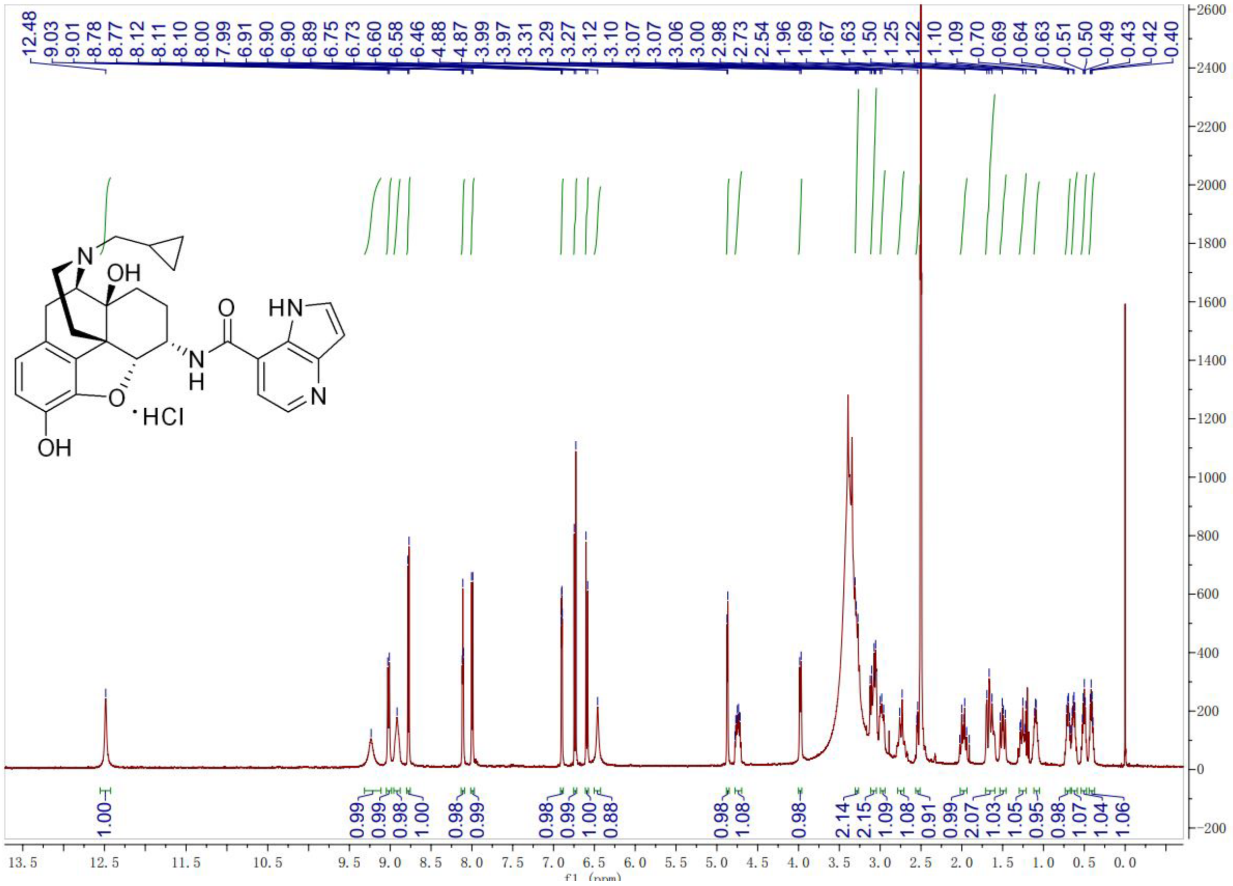

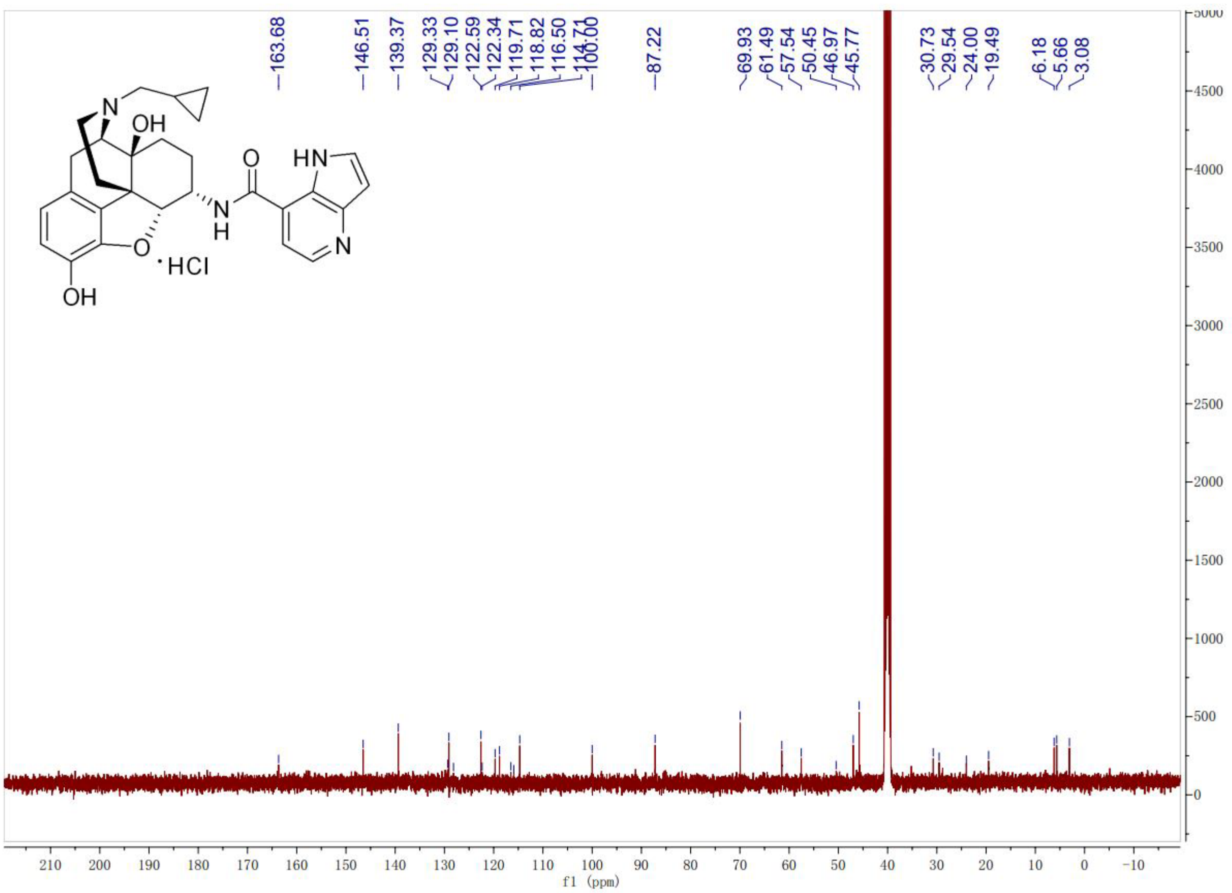
163.68
146.51
139.37
129.33
129.10
122.59
122.34
119.71
118.82
116.50
114.71
100.00
87.22
69.93
61.49
57.54
50.45
46.97
45.77
30.73
29.54
24.00
19.49
6.18
5.66
3.08
N
OH
O
HN
N
H
N
O
· HCl
OH
f1 (ppm)

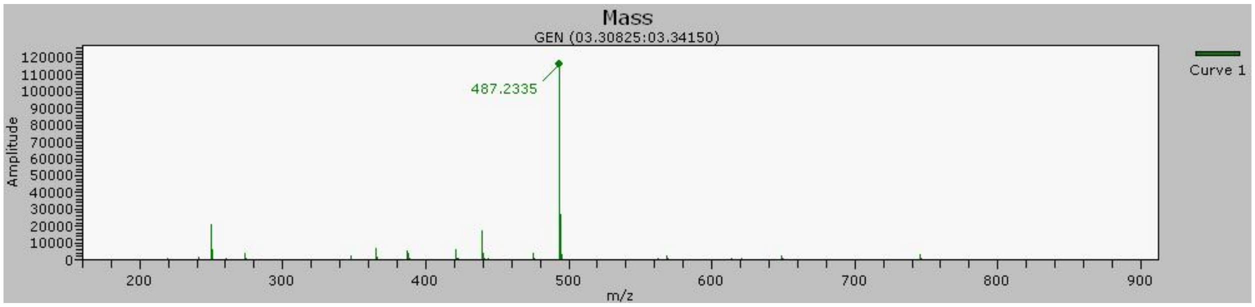
Mass
GEN (03.30825:03.34150)
487.2335
Amplitude
m/z
Curve 1

17-Cyclopropylmethyl-3,14β-dihydro-4,5α-epoxy-6β-[1H-pyrrolo[3,2-b]pyridine-7-carboxamide]morphinan Hydrochloride (**4**)

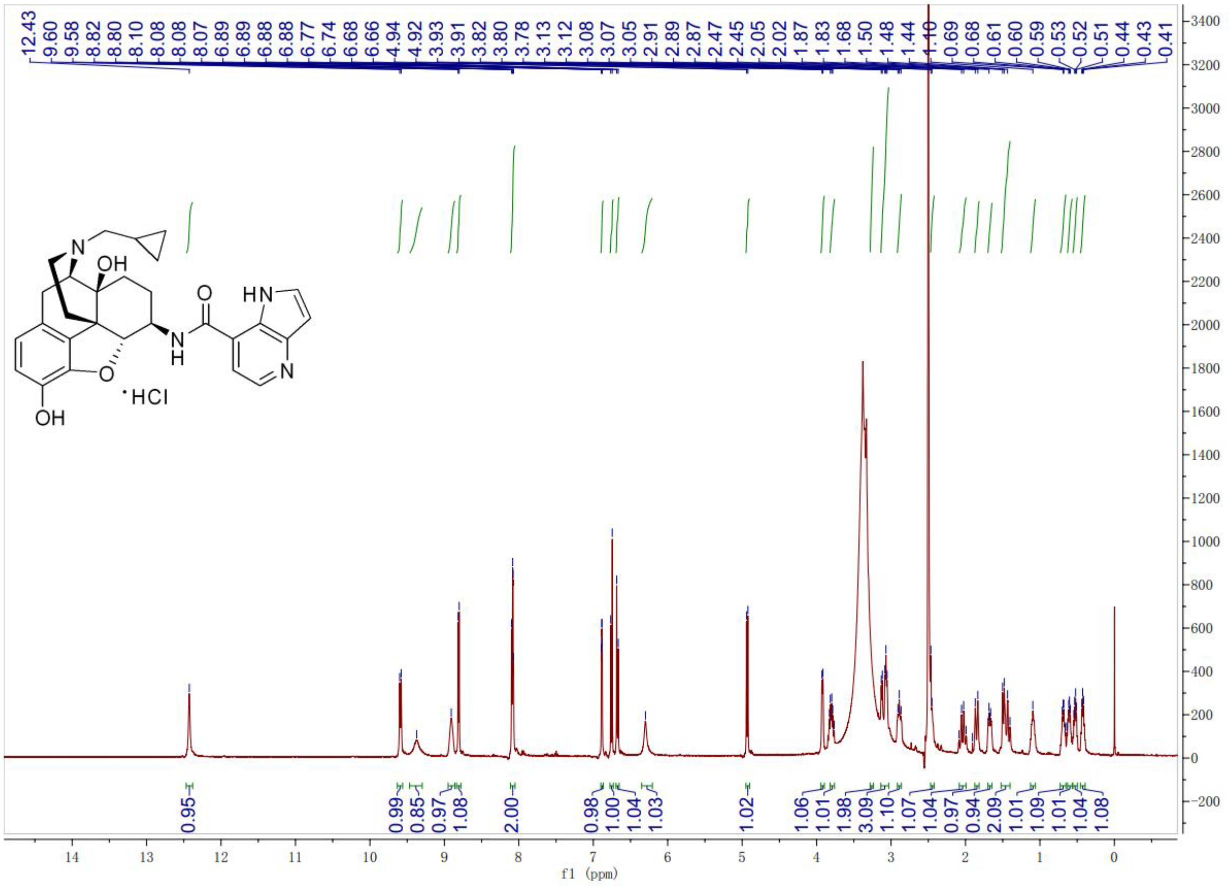

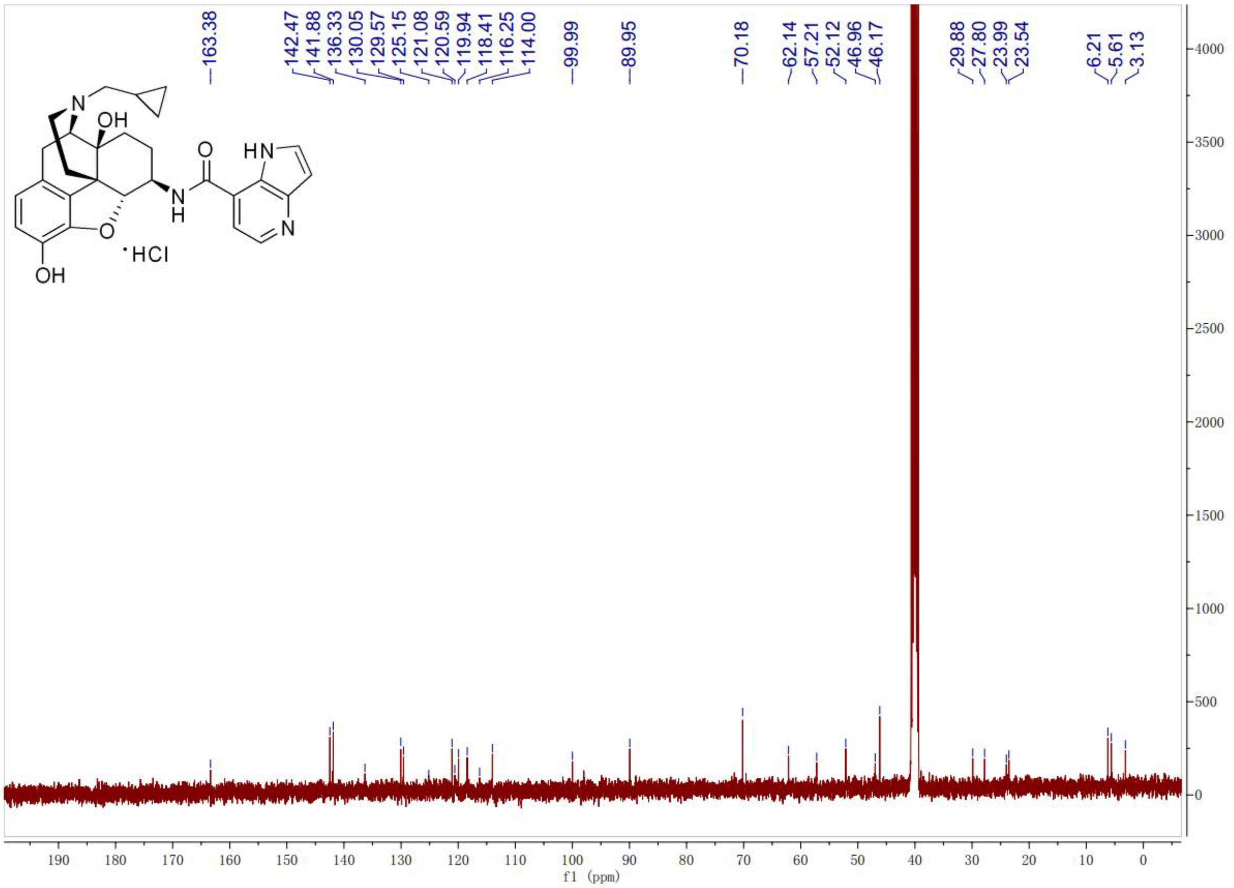

163.38
142.47
141.88
136.33
130.05
129.57
125.15
121.08
120.59
119.94
118.41
116.25
114.00
99.99
89.95
70.18
62.14
57.21
52.12
46.96
46.17
29.88
27.80
23.99
23.54
6.21
5.61
3.13
OH
O
HN
N
H
N
O
OH
·HCl
f1 (ppm)

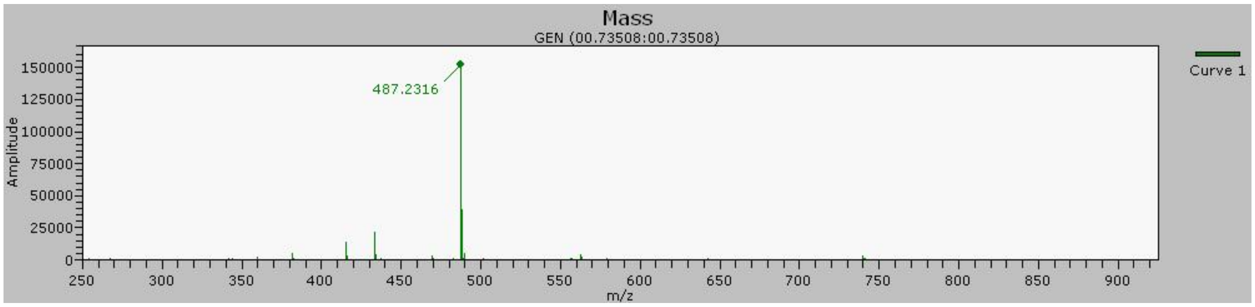

Mass
GEN (00.73508:00.73508)
487.2316
Amplitude
m/z
Curve 1

17-Cyclopropylmethyl-3,14β-dihydro-4,5α-epoxy-6α-[1H-pyrrolo[3,2-c]pyridine-7-carboxamide]morphinan Hydrochloride (**5**)

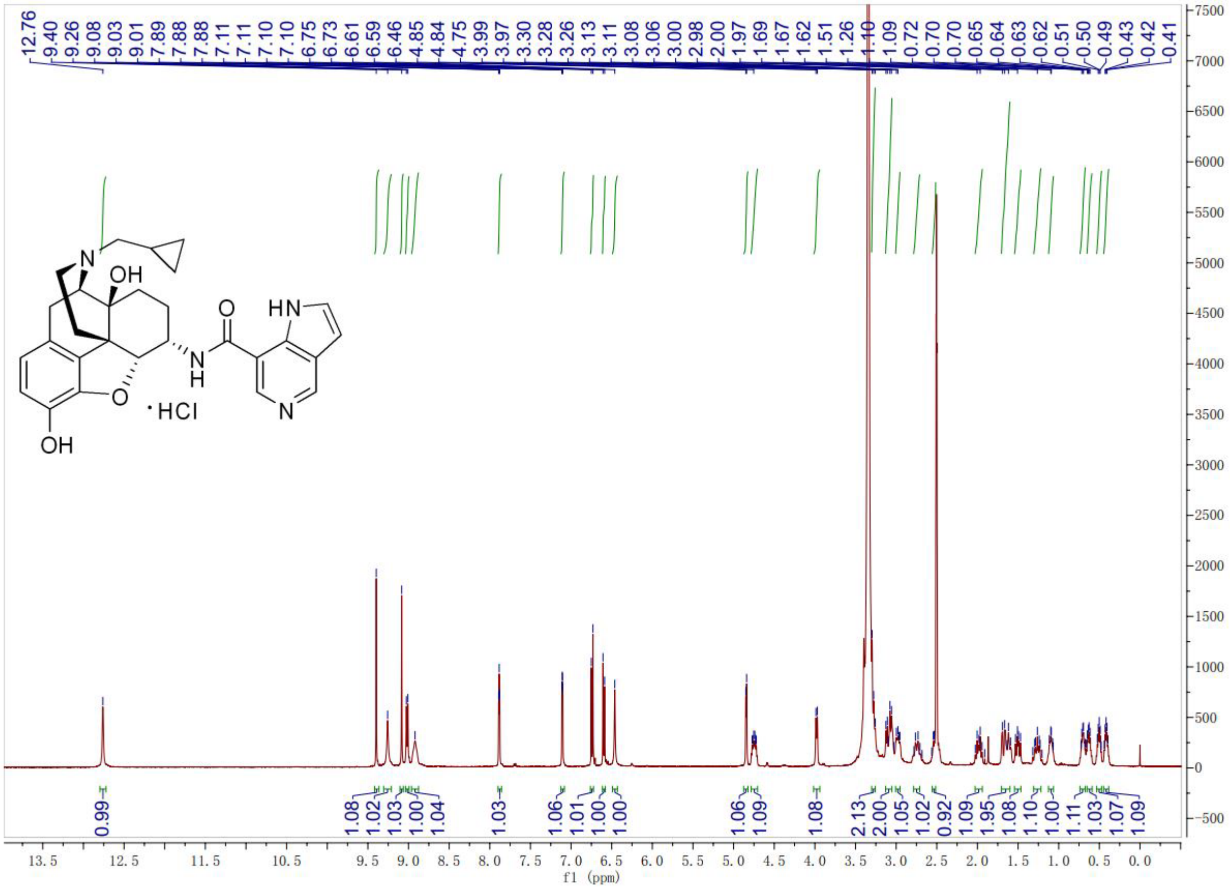

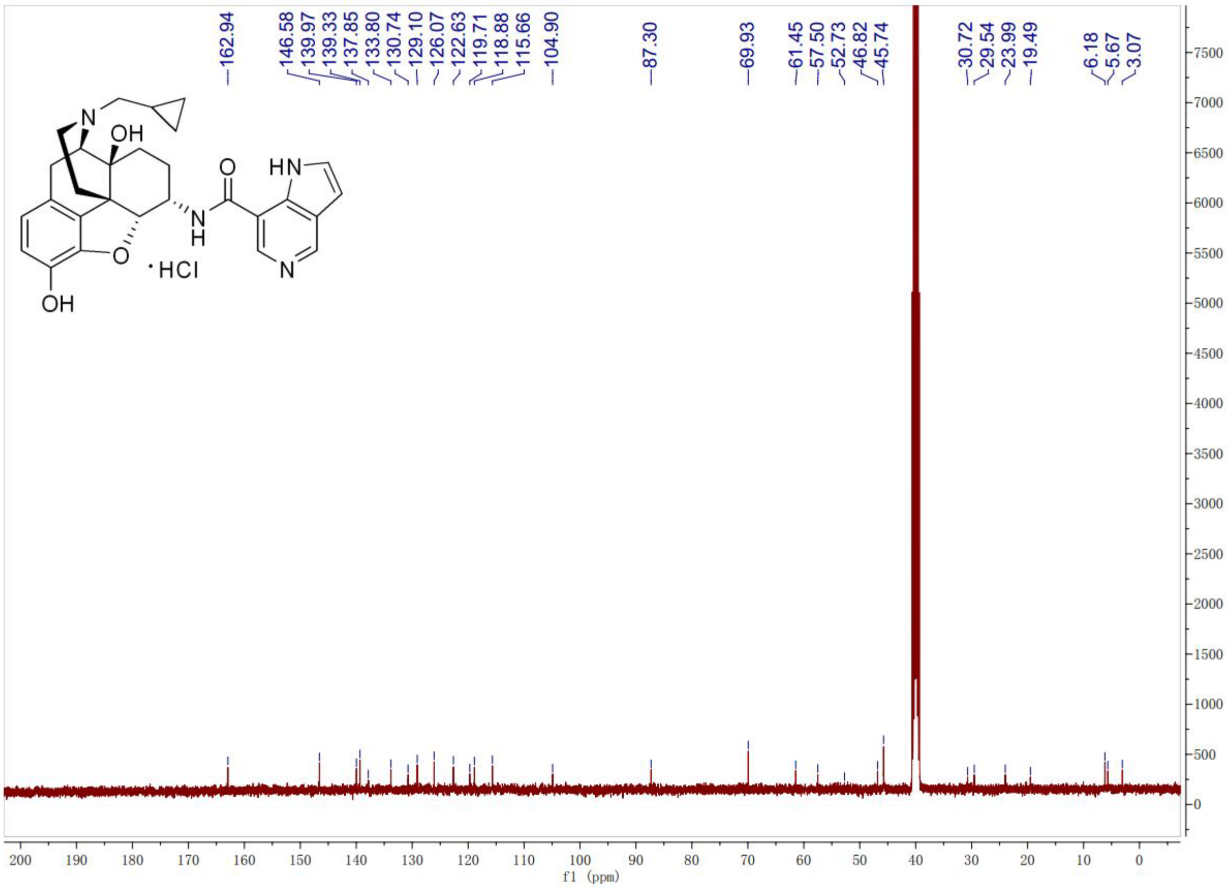
162.94
146.58
139.97
139.33
137.85
133.80
130.74
129.10
126.07
122.63
119.71
118.88
115.66
104.90
87.30
69.93
61.45
57.50
52.73
46.82
45.74
30.72
29.54
23.99
19.49
6.18
5.67
3.07
OH
HN
O
N
H
·HCl
N
OH
f1 (ppm)

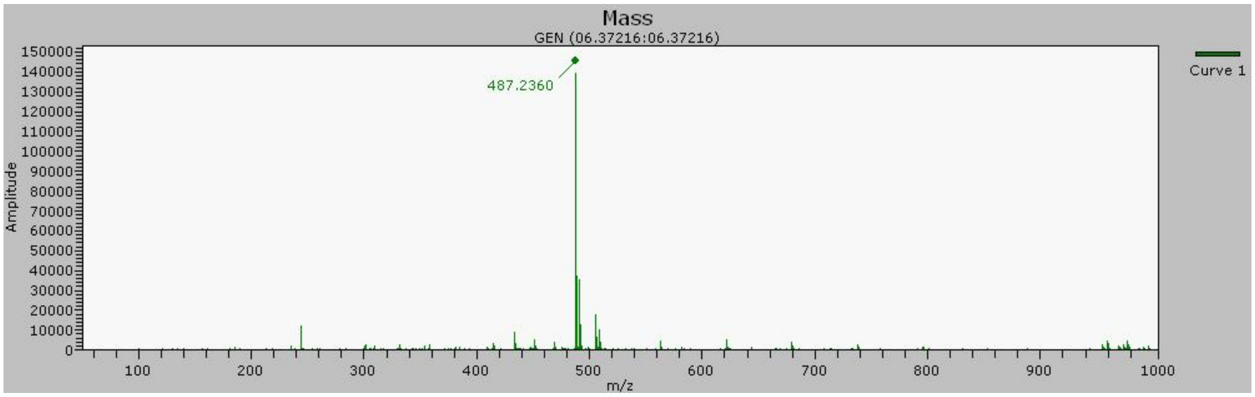
Mass
GEN (06.37216:06.37216)
487.2360
Curve 1
Amplitude
m/z

17-Cyclopropylmethyl-3,14β-dihydro-4,5α-epoxy-6β-[1H-pyrrolo[3,2-c]pyridine-7-carboxamide]morphinan Hydrochloride (**6**)

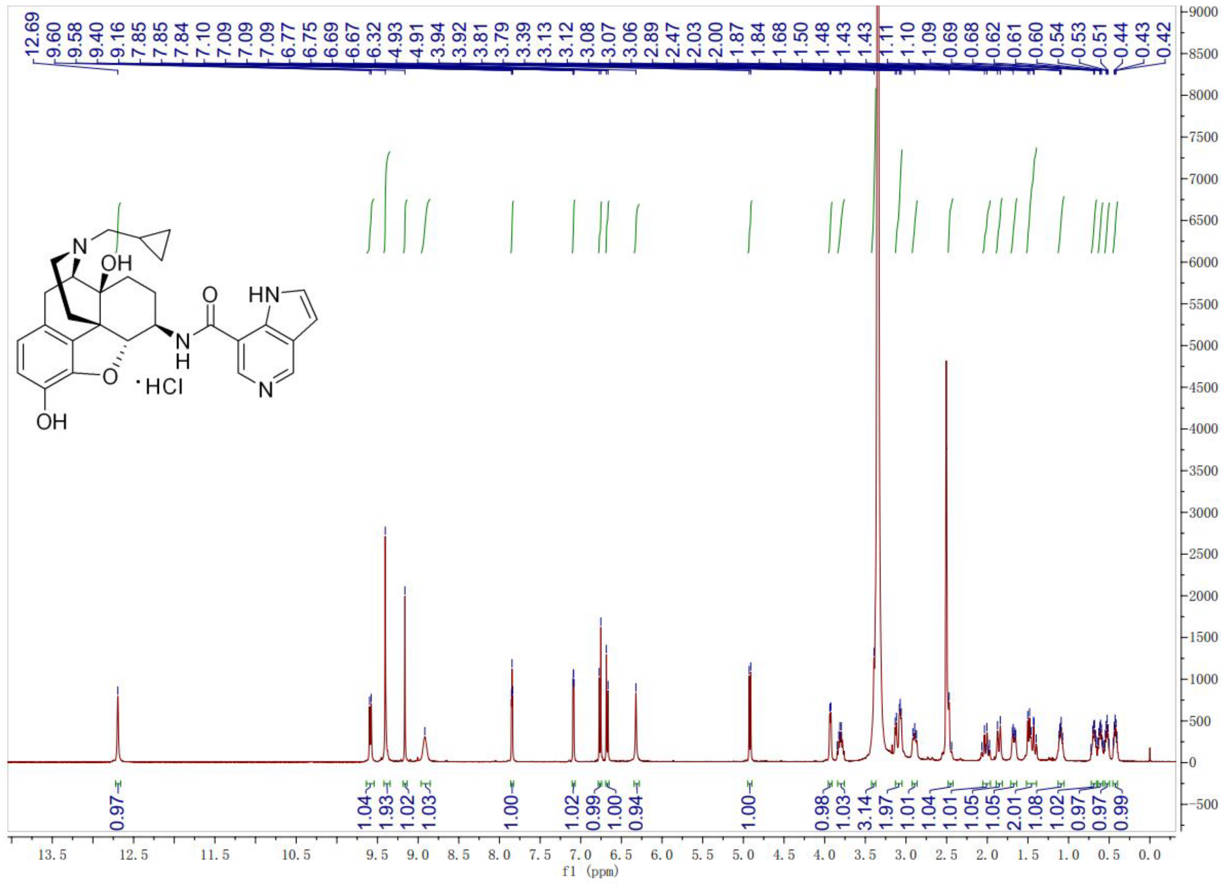

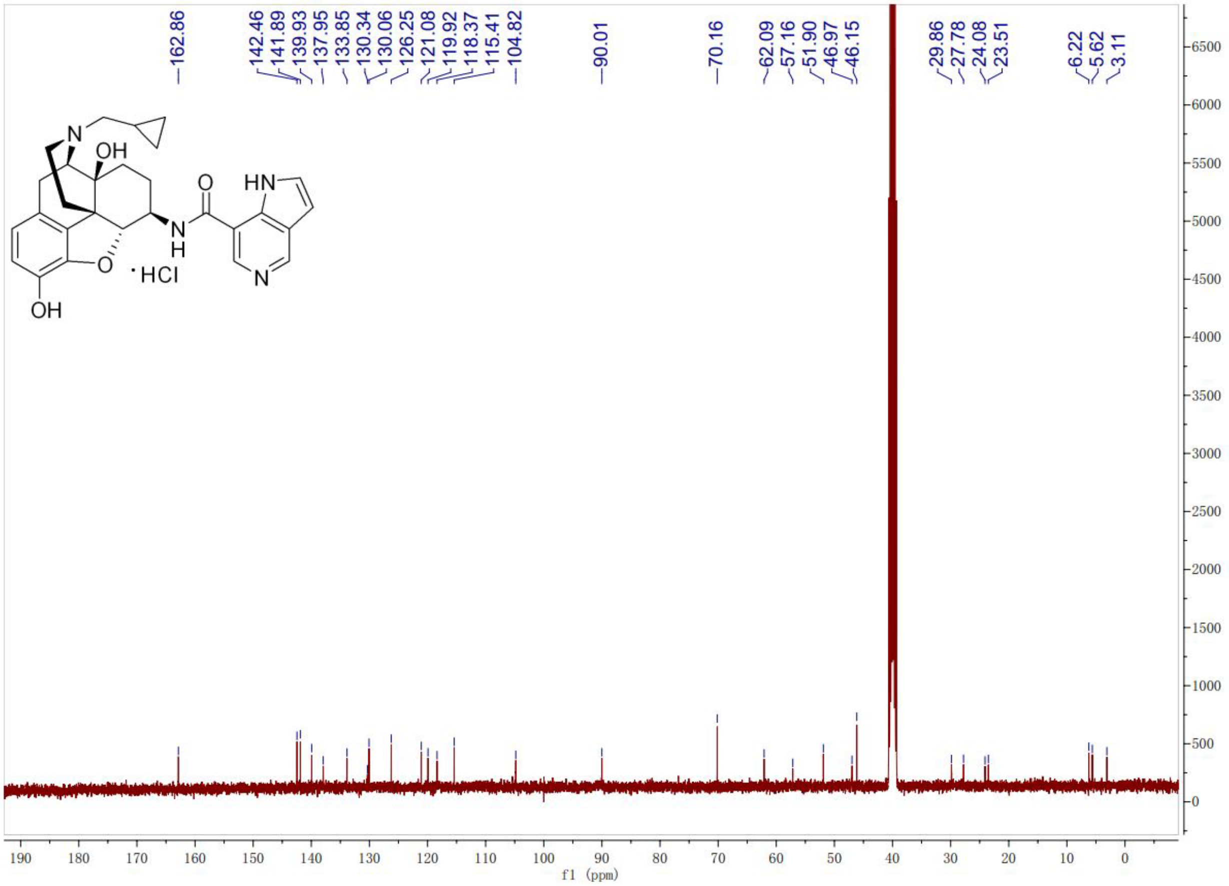

162.86
142.46
141.89
139.93
137.95
133.85
130.34
130.06
126.25
121.08
119.92
118.37
115.41
104.82
90.01
70.16
62.09
57.16
51.90
46.97
46.15
29.86
27.78
24.08
23.51
6.22
5.62
3.11
OH
N
O
HN
N
H
O
·HCl
N
OH
f1 (ppm)

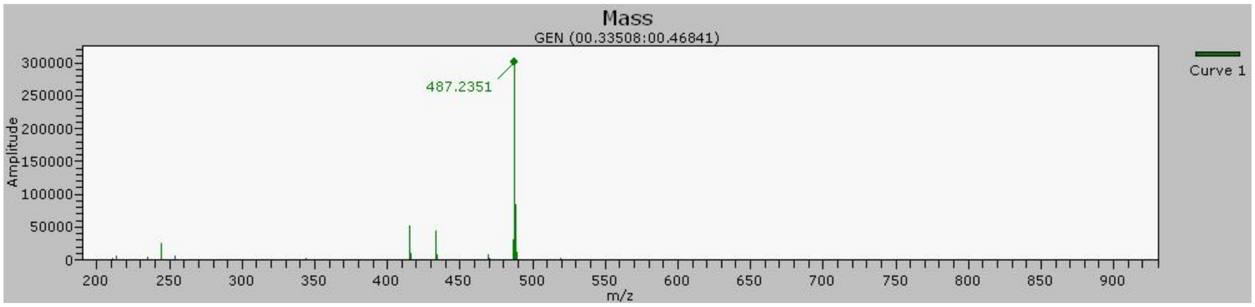

Mass
GEN (00.33508:00.46841)
487.2351
Amplitude
m/z
Curve 1

17-Cyclopropylmethyl-3,14β-dihydro-4,5α-epoxy-6α-[1H-pyrrolo[2,3-c]pyridine-7-carboxamide]morphinan Hydrochloride (**7**)

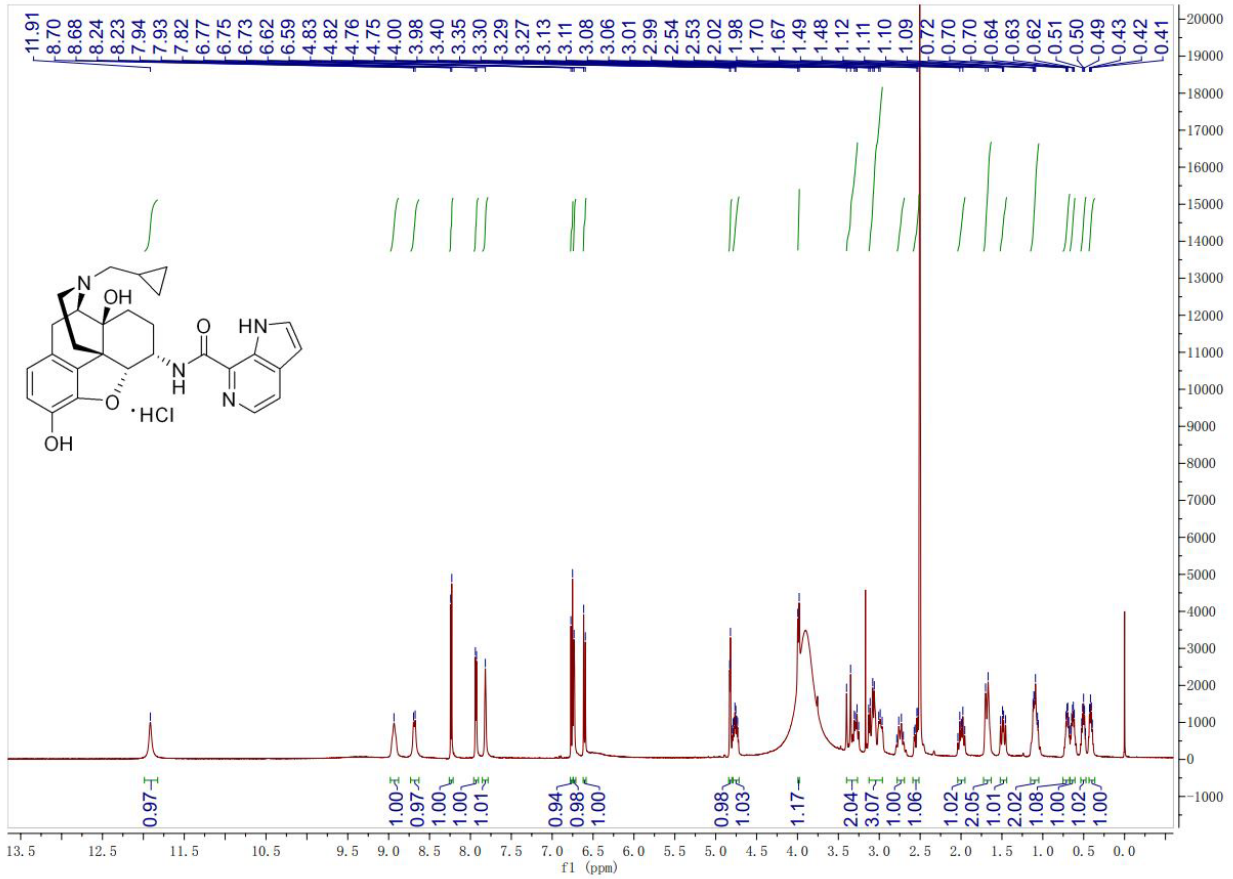

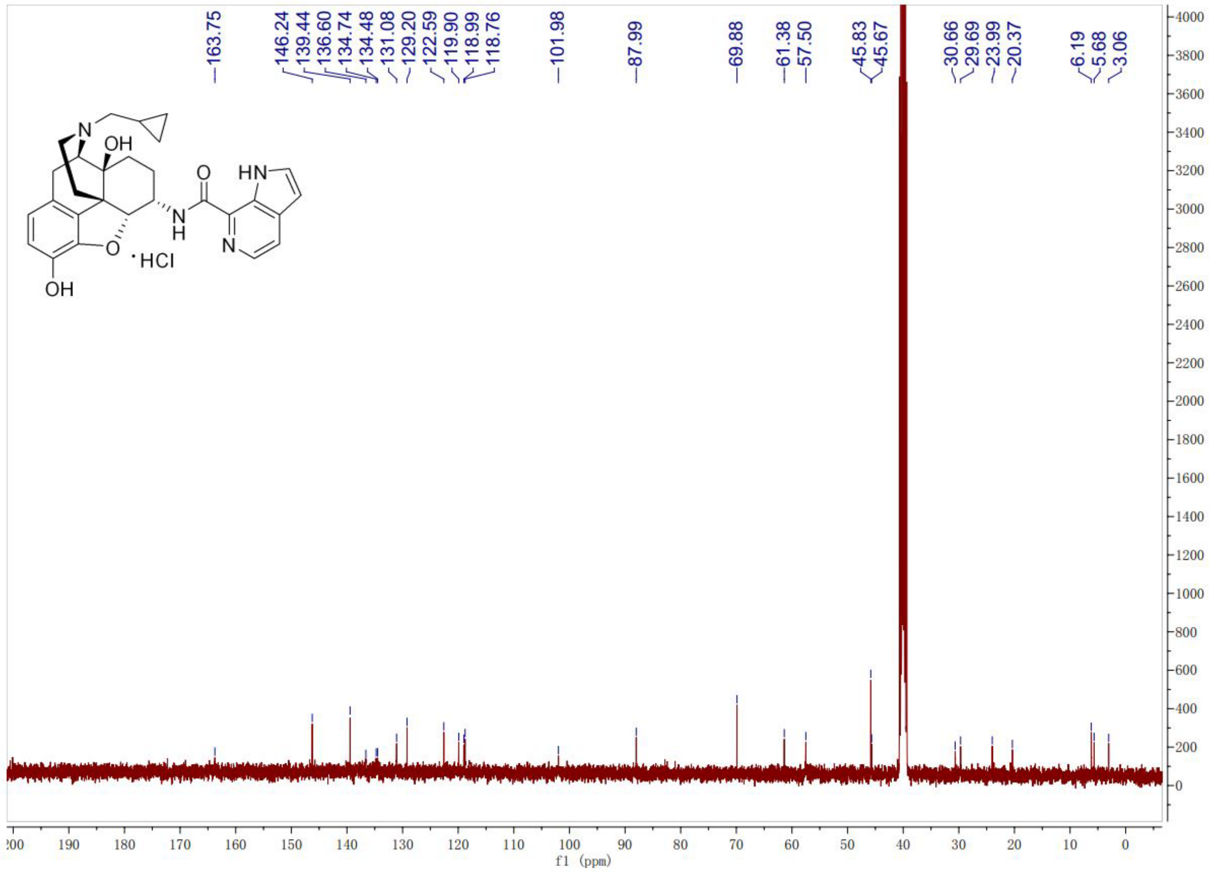
163.75
146.24
139.44
136.60
134.74
134.48
131.08
129.20
122.59
119.90
118.99
118.76
101.98
87.99
69.88
61.38
57.50
45.83
45.67
30.66
29.69
23.99
20.37
6.19
5.68
3.06
N
OH
O
HN
N
H
N
O
OH
· HCl
f1 (ppm)

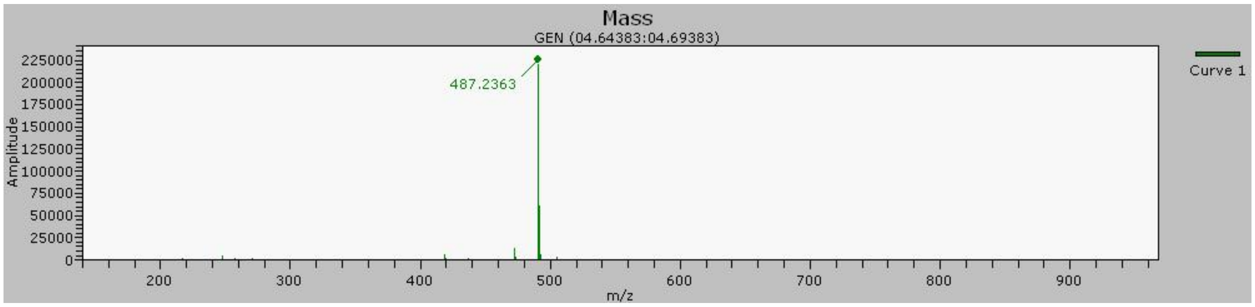
Mass
GEN (04.64383:04.69383)
487.2363
Amplitude
m/z
Curve 1

17-Cyclopropylmethyl-3,14β-dihydro-4,5α-epoxy-6β-[1H-pyrrolo[2,3-c]pyridine-7-carboxamide]morphinan Hydrochloride (**8**)

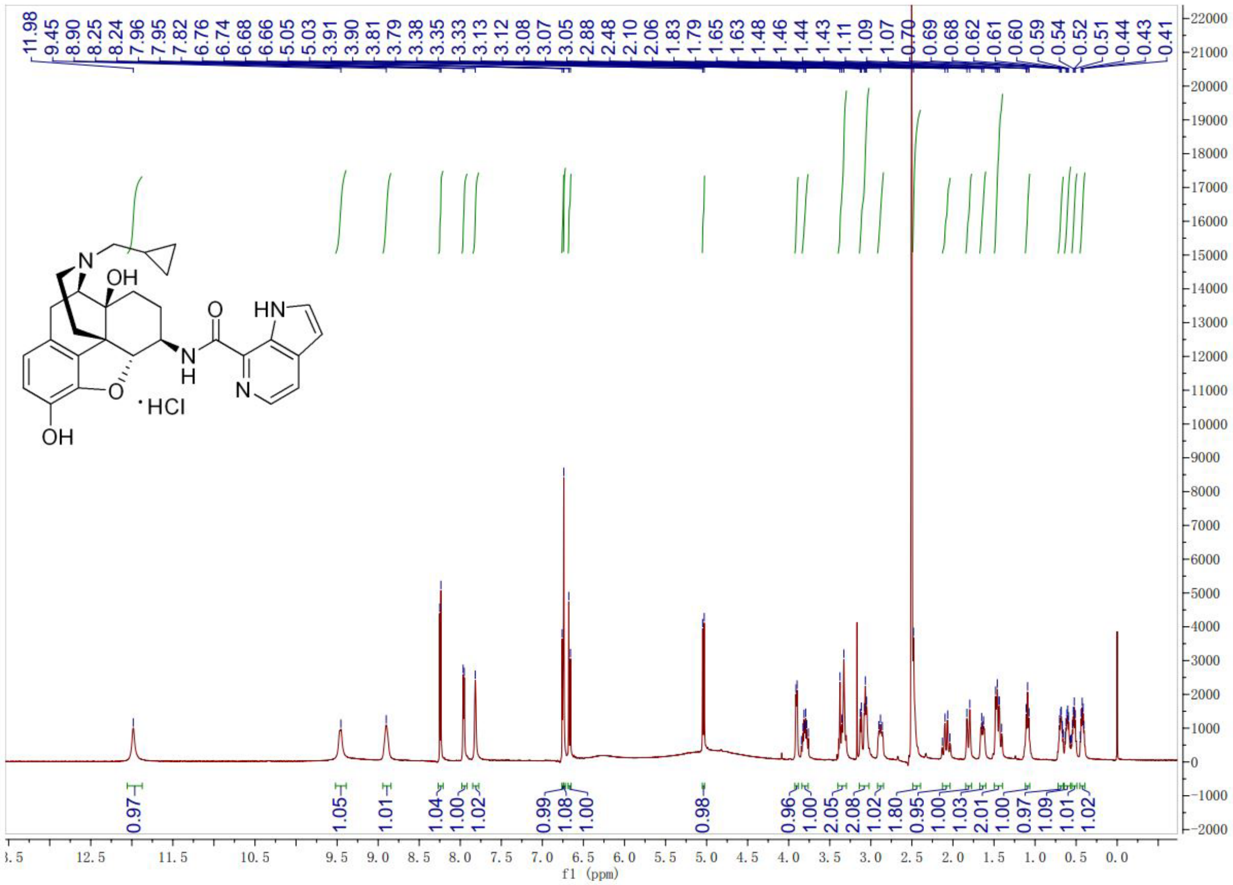

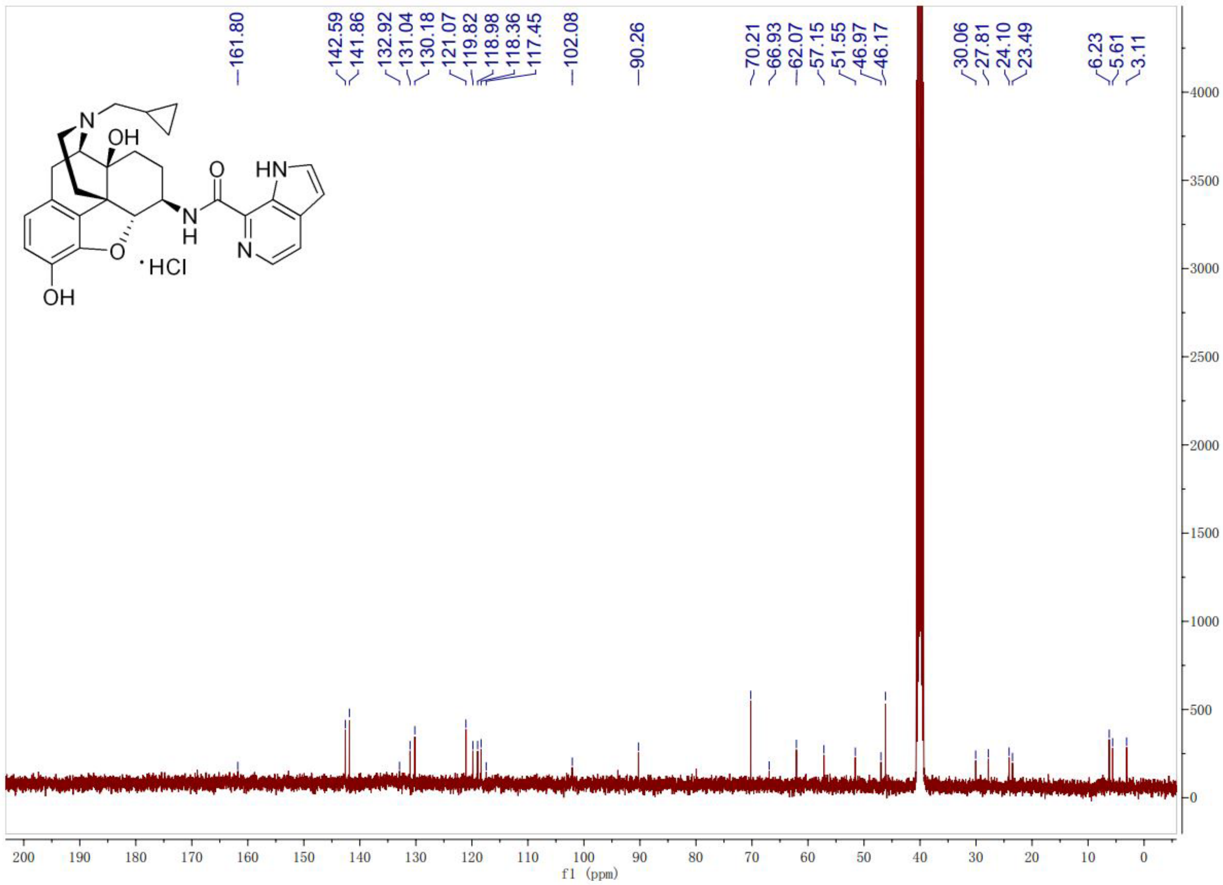

161.80
142.59
141.86
132.92
131.04
130.18
121.07
119.82
118.98
118.36
117.45
102.08
90.26
70.21
66.93
62.07
57.15
51.55
46.97
46.17
30.06
27.81
24.10
23.49
6.23
5.61
3.11
OH
HN
O
N
H
N
O
·HCl
OH
f1 (ppm)

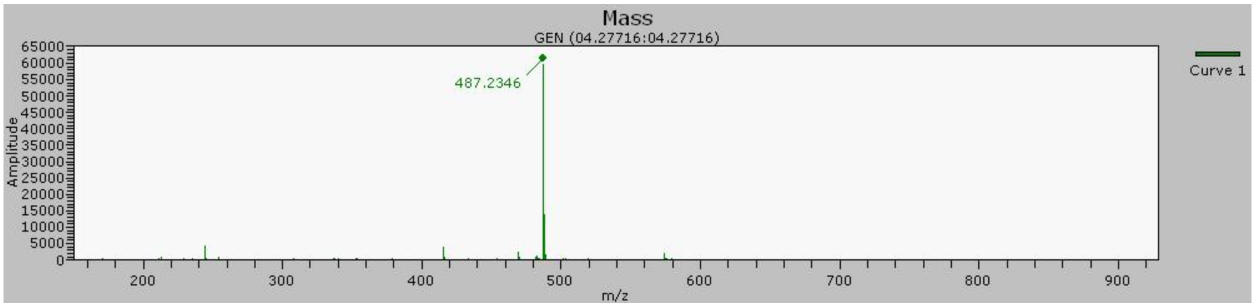

Mass
GEN (04.27716:04.27716)
487.2346
Amplitude
m/z
Curve 1

17-Cyclopropylmethyl-3,14β-dihydro-4,5α-epoxy-6α-[1H-indazole-6-carboxamide]morphinan Hydrochloride (**9**)

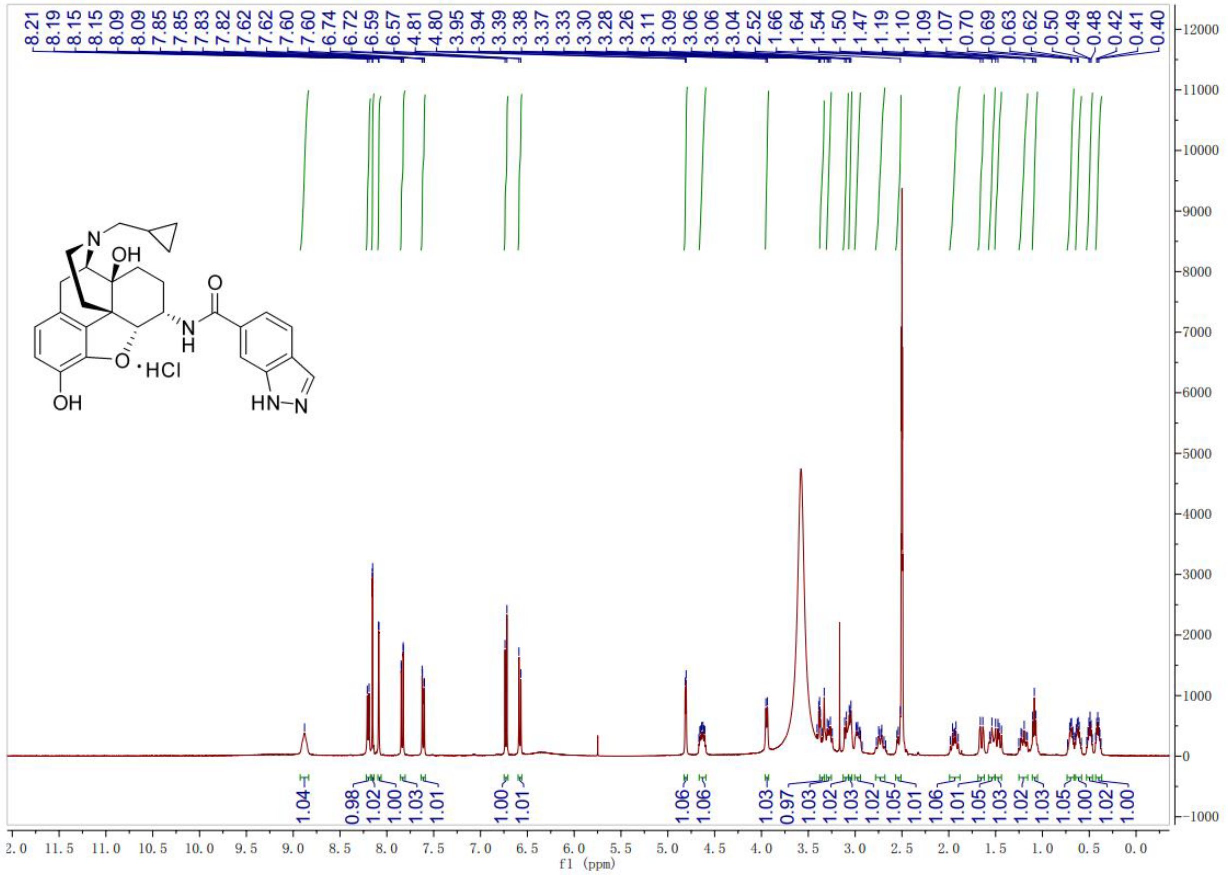

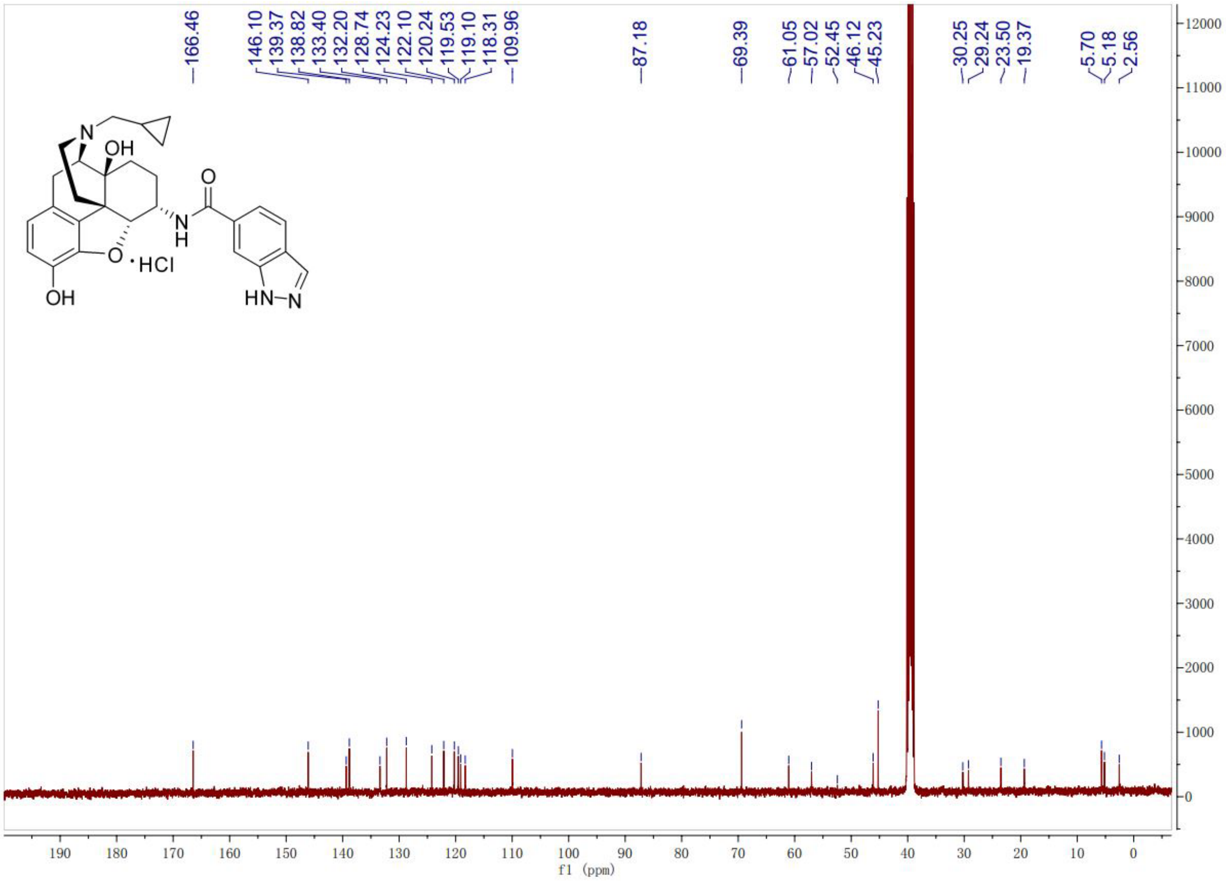
166.46
146.10
139.37
138.82
133.40
132.20
128.74
124.23
122.10
120.24
119.53
119.10
118.31
109.96
87.18
69.39
61.05
57.02
52.45
46.12
45.23
30.25
29.24
23.50
19.37
5.70
5.18
2.56
OH
O
N
H
O
· HCl
OH
HN–N
f1 (ppm)

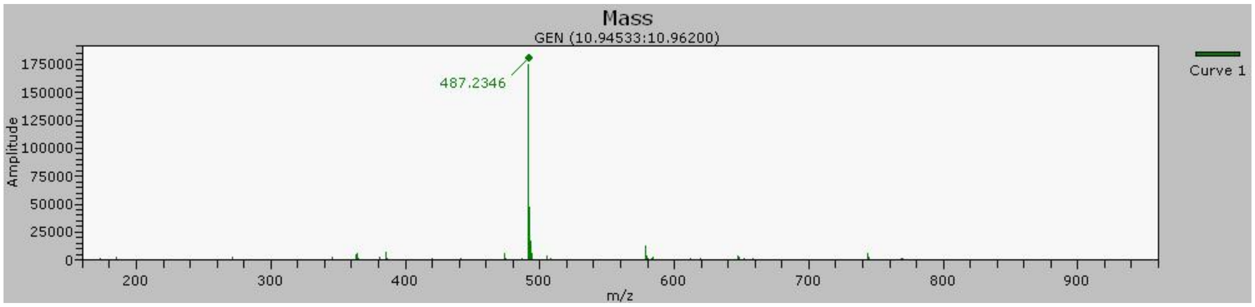
Mass
GEN (10.94533:10.96200)
487.2346
Amplitude
m/z
Curve 1

17-Cyclopropylmethyl-3,14β-dihydro-4,5α-epoxy-6β-[1H-indazole-6-carboxamide]morphinan Hydrochloride (**10**)

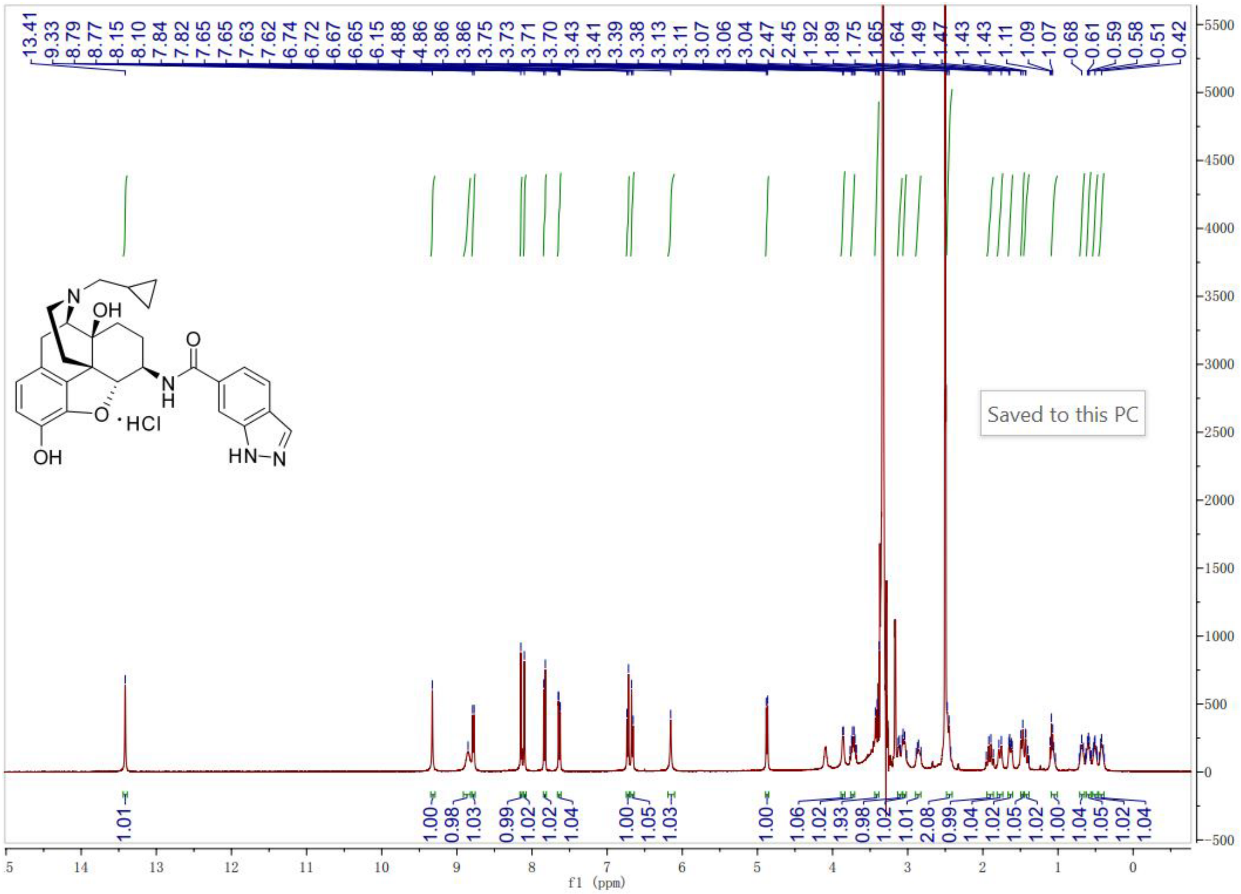

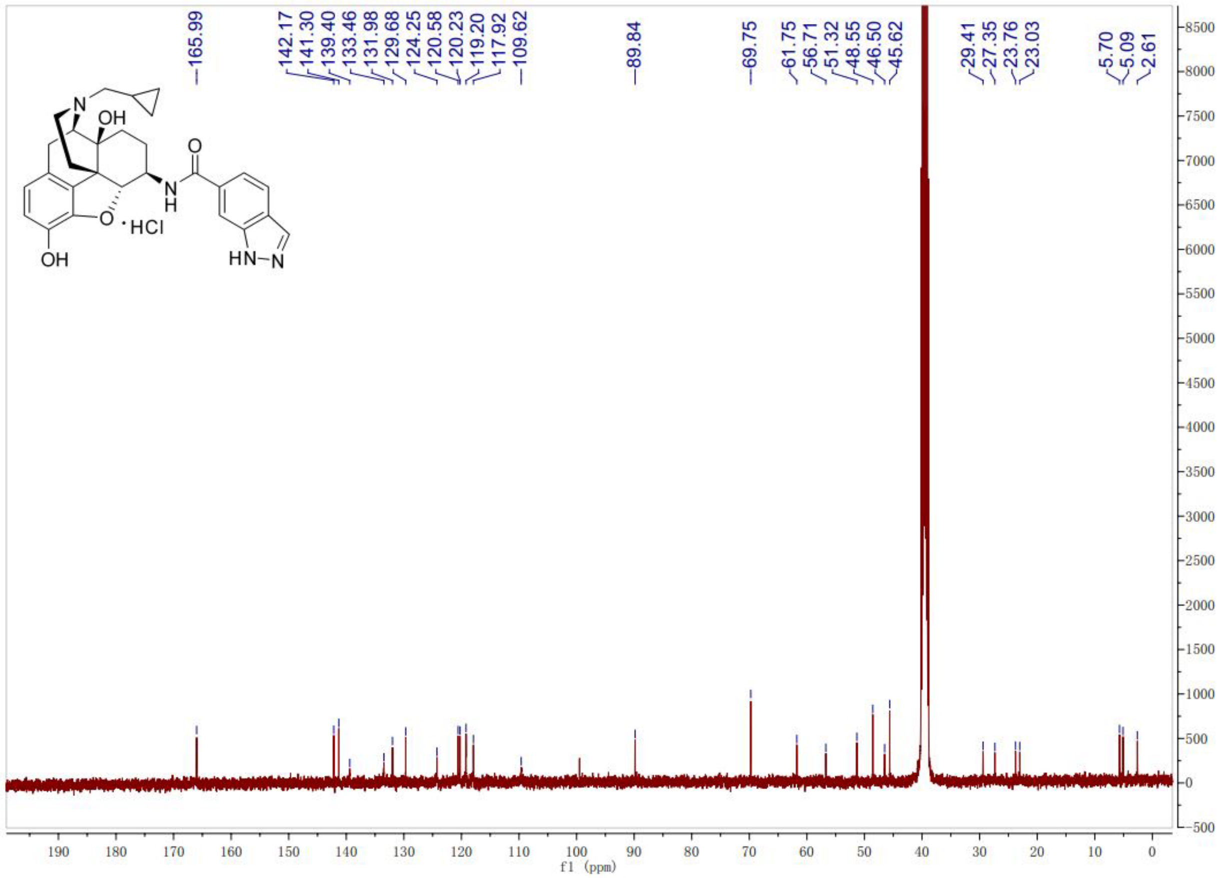

165.99
142.17
141.30
139.40
133.46
131.98
129.68
124.25
120.58
120.23
119.20
117.92
109.62
89.84
69.75
61.75
56.71
51.32
48.55
46.50
45.62
29.41
27.35
23.76
23.03
5.70
5.09
2.61
OH
O
N
H
HCl
OH
HN–N
f1 (ppm)

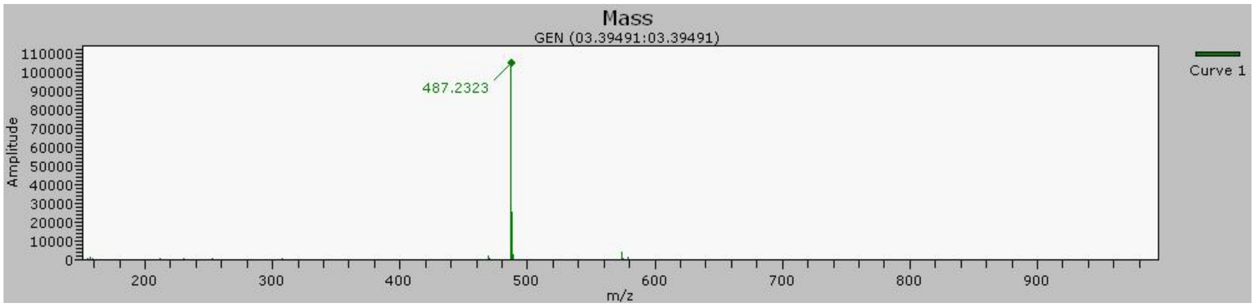

Mass
GEN (03.39491:03.39491)
487.2323
Amplitude
m/z
Curve 1

17-Cyclopropylmethyl-3,14β-dihydro-4,5α-epoxy-6α-[1H-pyrrolo[3,2-b]pyridine-6-carboxamide]morphinan Hydrochloride (**11**)

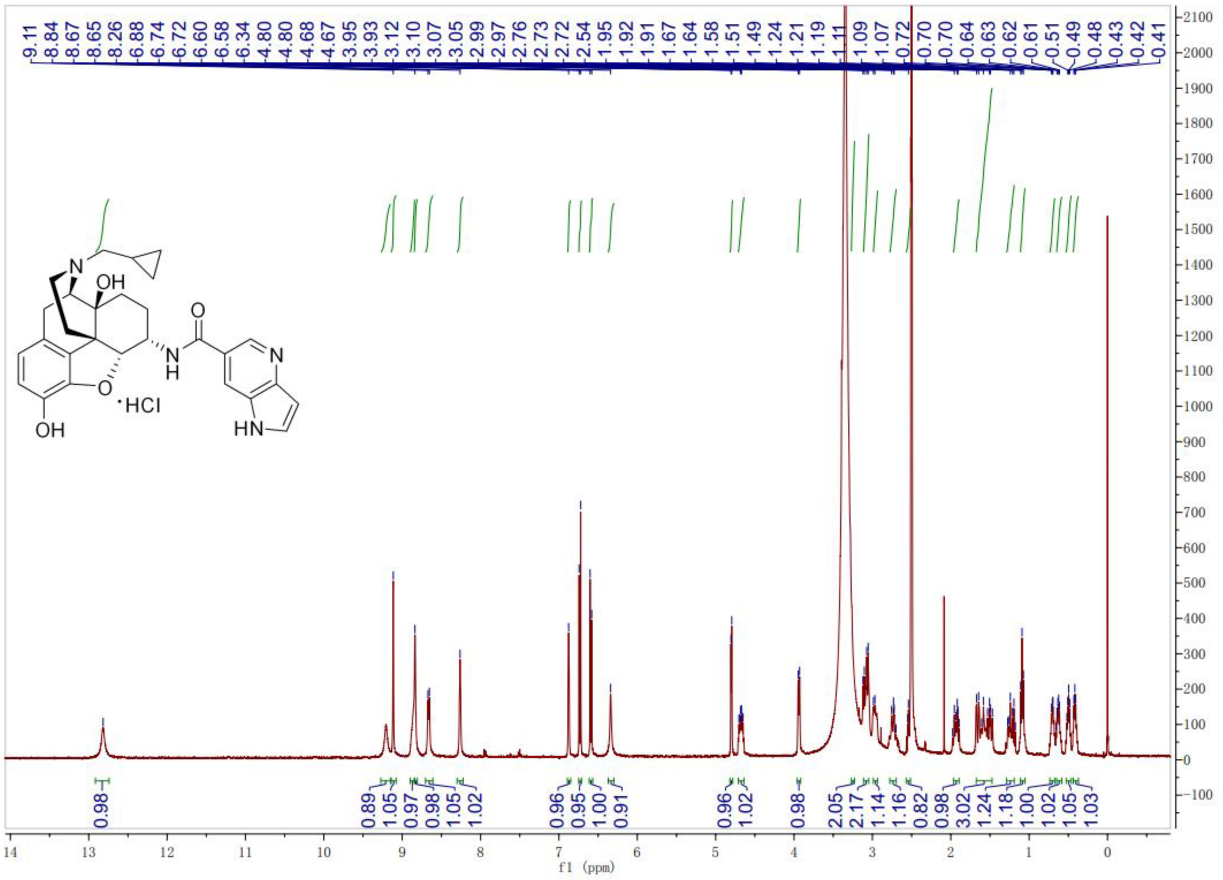

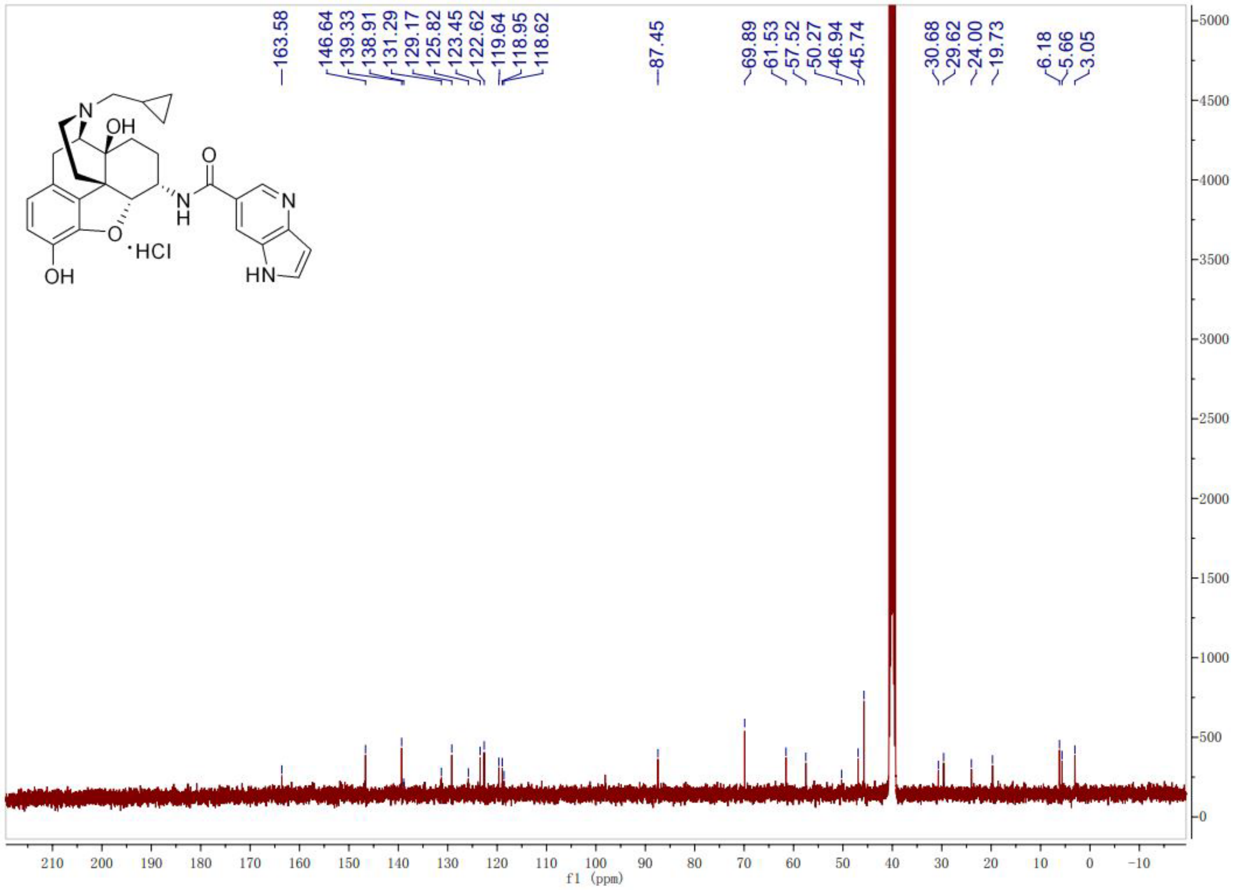
163.58
146.64
139.33
138.91
131.29
129.17
125.82
123.45
122.62
119.64
118.95
118.62
87.45
69.89
61.53
57.52
50.27
46.94
45.74
30.68
29.62
24.00
19.73
6.18
5.66
3.05
OH
N
O
H
·HCl
HN
f1 (ppm)

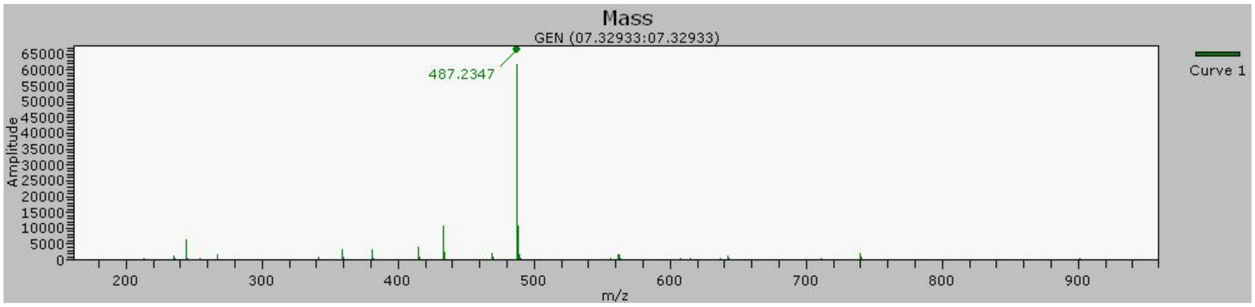
Mass
GEN (07.32933:07.32933)
487.2347
Curve 1
Amplitude
m/z

17-Cyclopropylmethyl-3,14β-dihydro-4,5α-epoxy-6β-[1H-pyrrolo[3,2-b]pyridine-6-carboxamide] morphinan Hydrochloride (**12**)

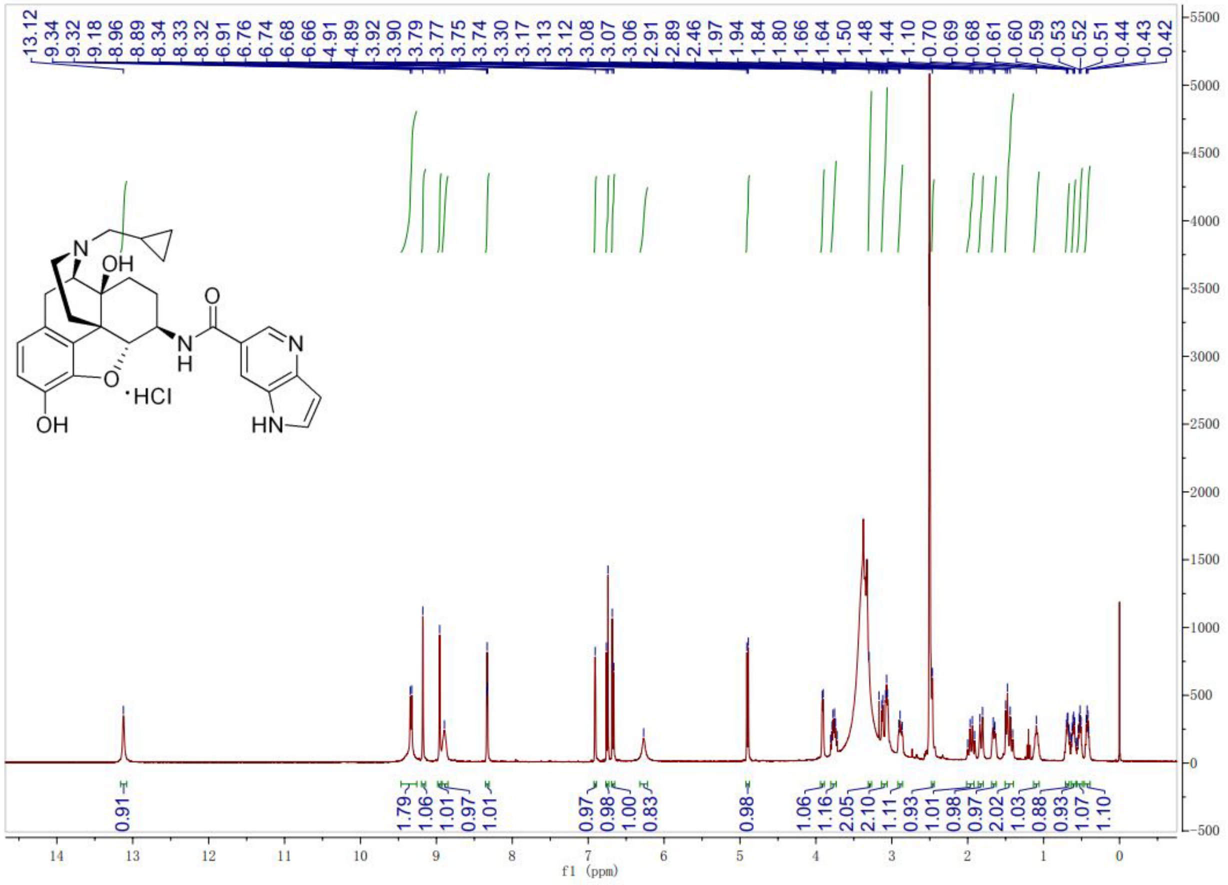

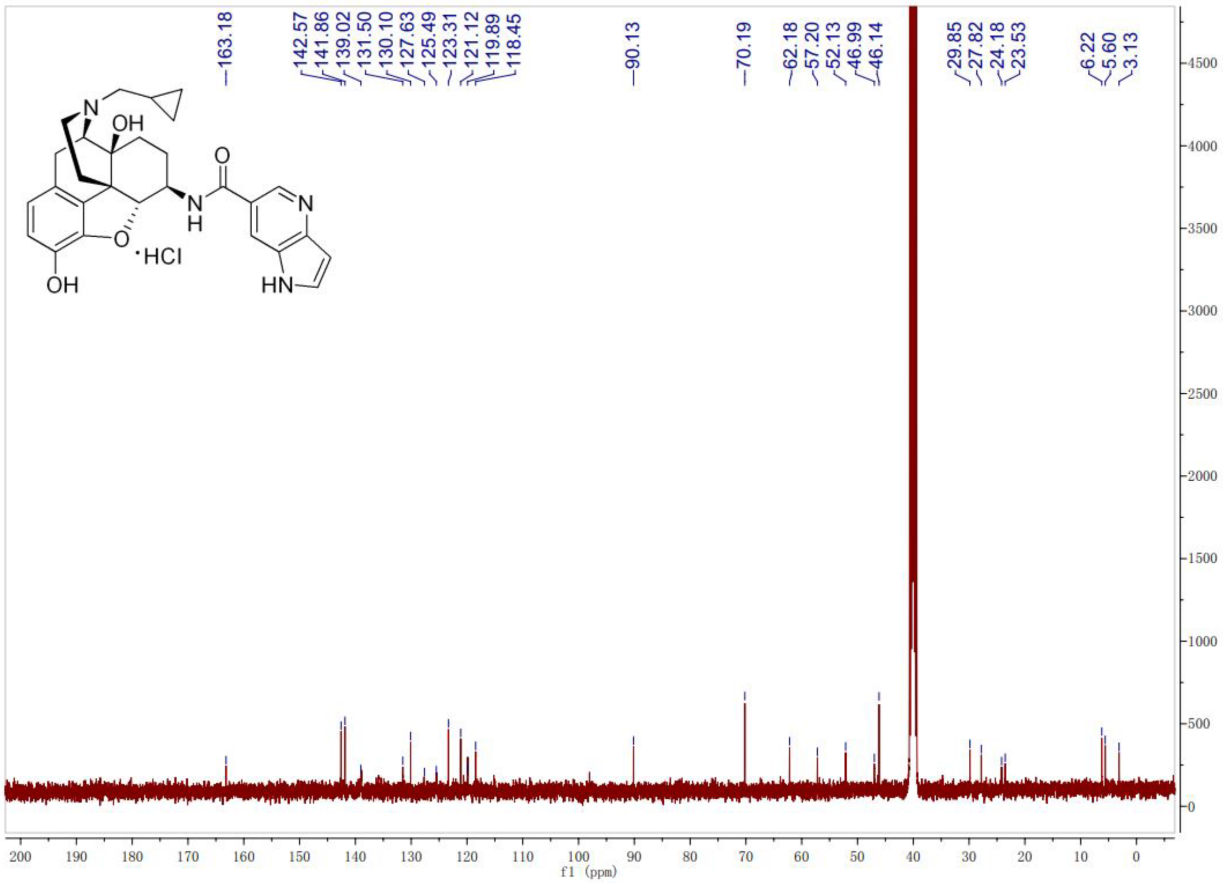
163.18
142.57
141.86
139.02
131.50
130.10
127.63
125.49
123.31
121.12
119.89
118.45
90.13
70.19
62.18
57.20
52.13
46.99
46.14
29.85
27.82
24.18
23.53
6.22
5.60
3.13
OH
O
N
H
N
O
·HCl
OH
HN
f1 (ppm)

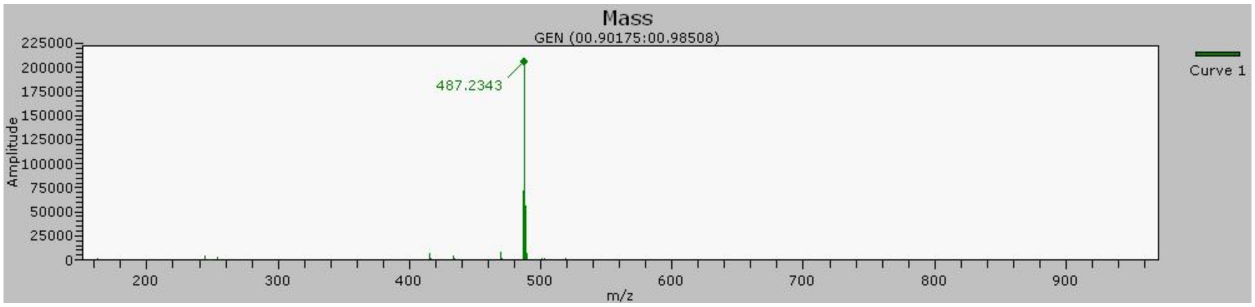
Mass
GEN (00.90175:00.98508)
487.2343
Amplitude
m/z
Curve 1

17-Cyclopropylmethyl-3,14β-dihydro-4,5α-epoxy-6α-[1H-pyrrolo[3,2-c]pyridine-6-carboxamide]morphinan Hydrochloride (**13**)

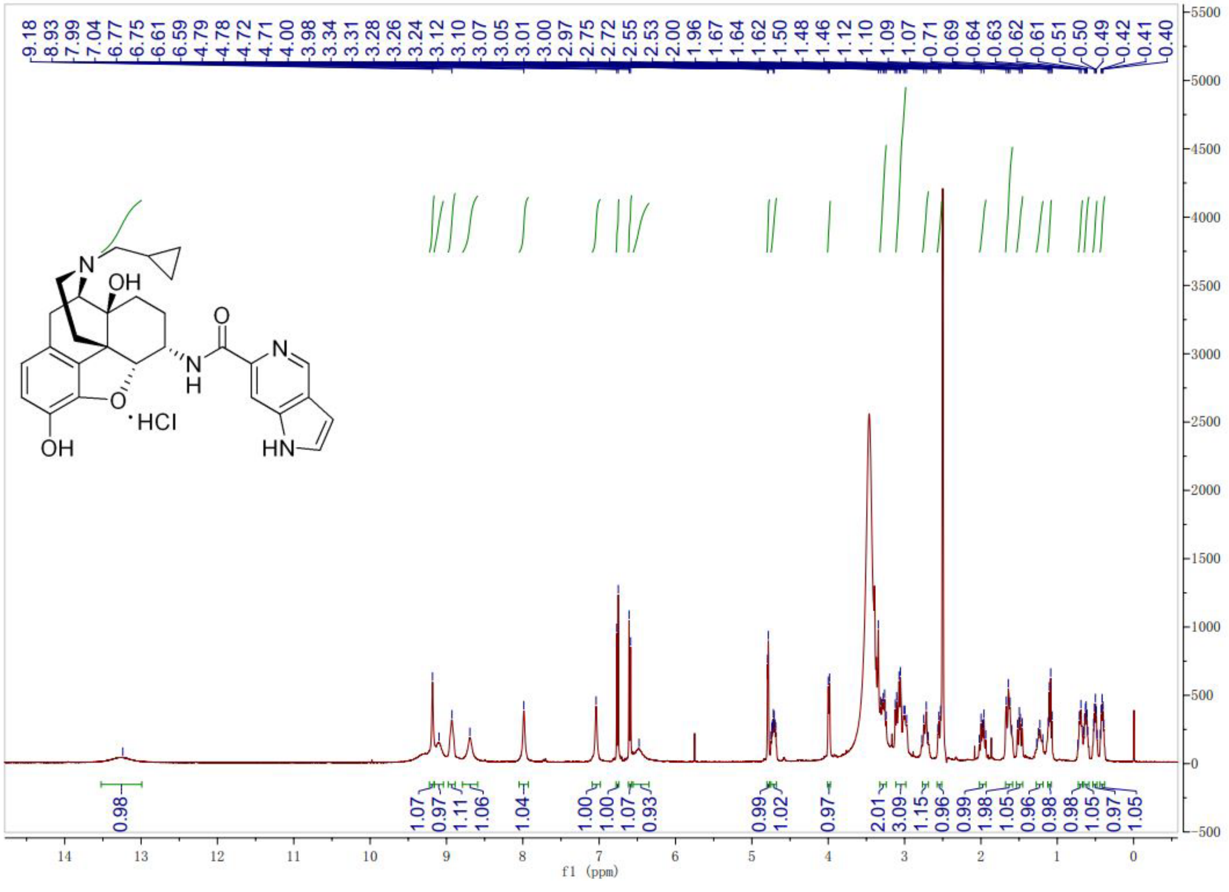

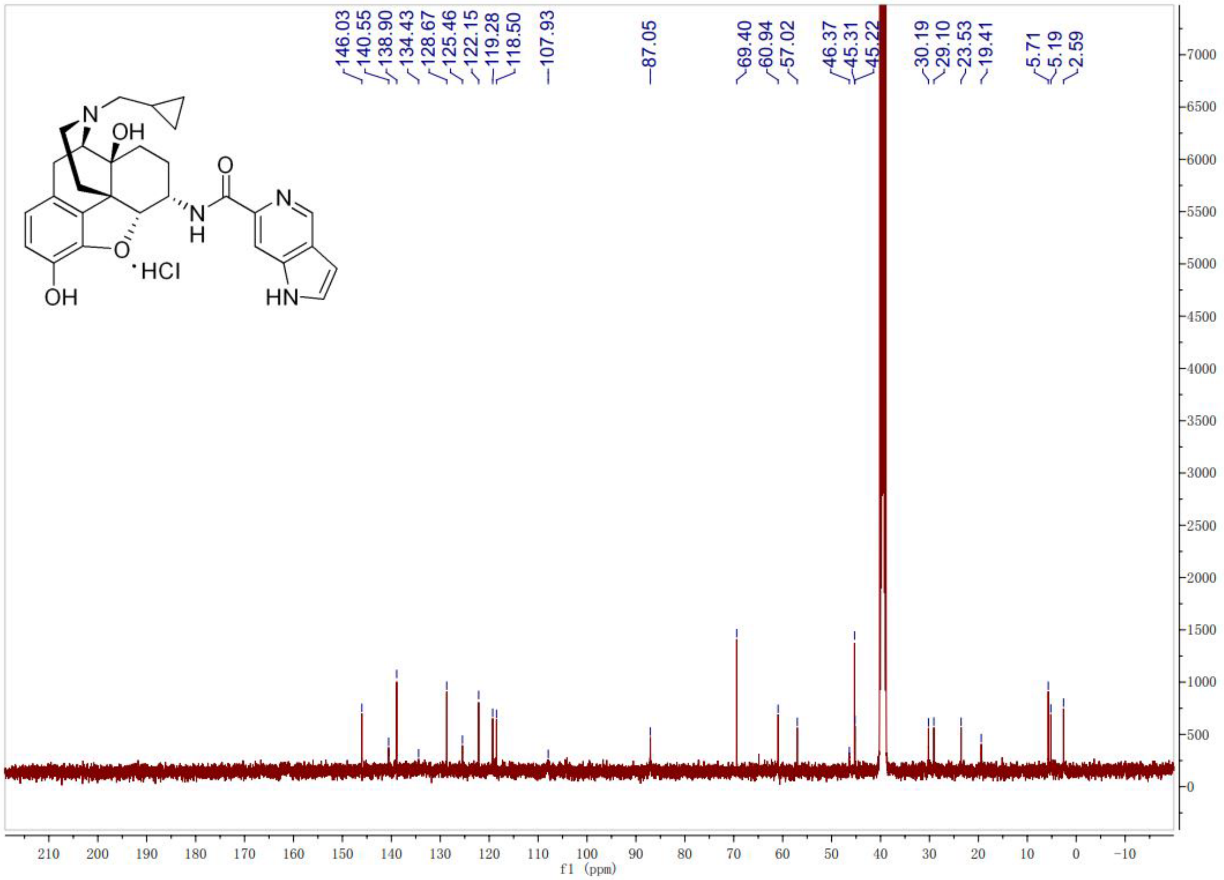
146.03
140.55
138.90
134.43
128.67
125.46
122.15
119.28
118.50
107.93
87.05
69.40
60.94
57.02
46.37
45.31
45.22
30.19
29.10
23.53
19.41
5.71
5.19
2.59
OH
N
O
N
H
N
HN
O
·HCl
OH
f1 (ppm)

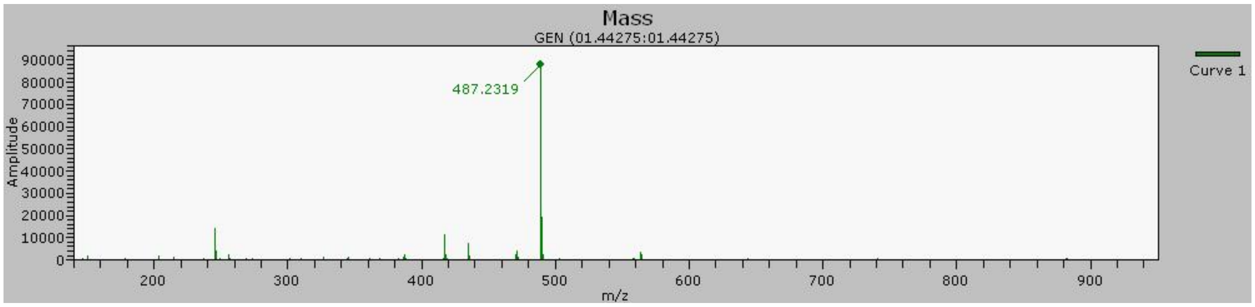
Mass
GEN (01.44275:01.44275)
487.2319
Amplitude
m/z
Curve 1

17-Cyclopropylmethyl-3,14β-dihydro-4,5α-epoxy-6β-[1H-pyrrolo[3,2-c]pyridine-6-carboxamide]morphinan Hydrochloride (**14**)

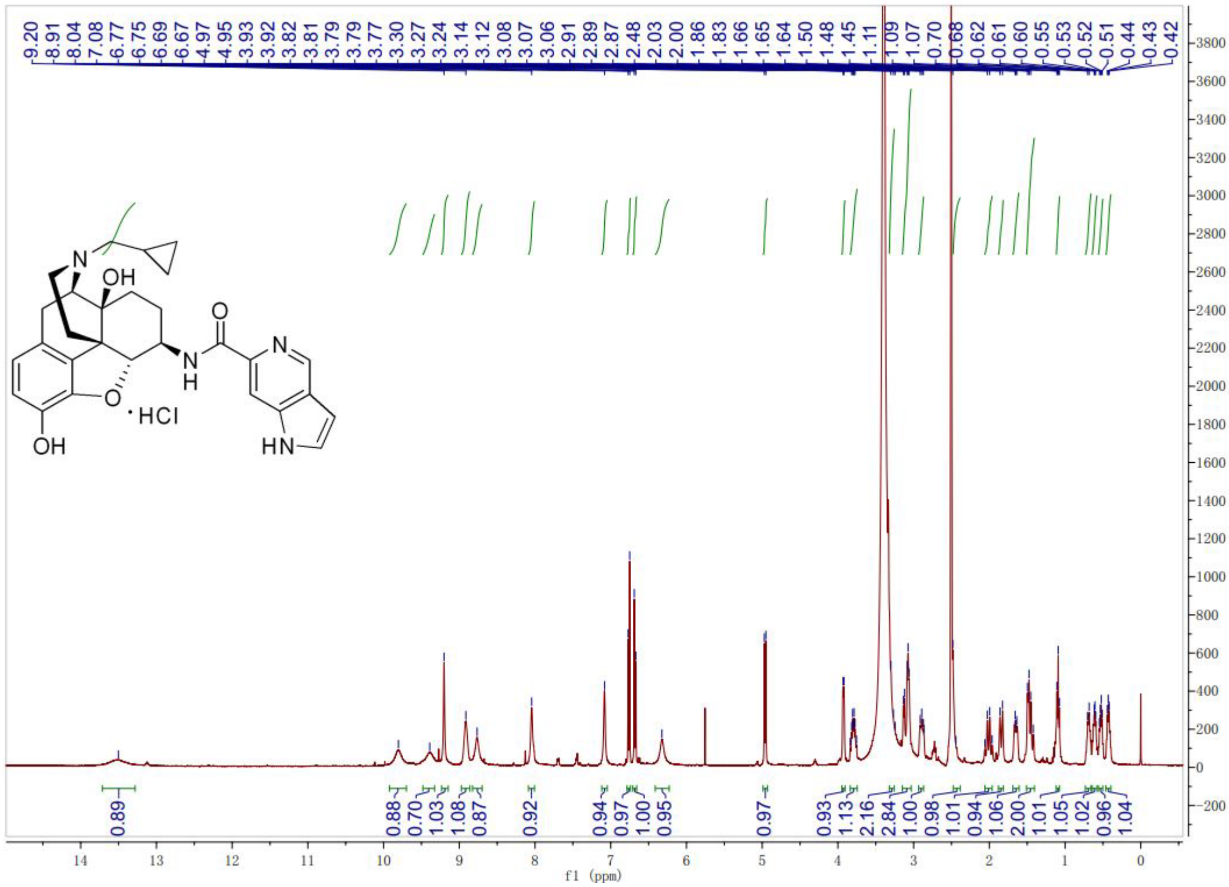

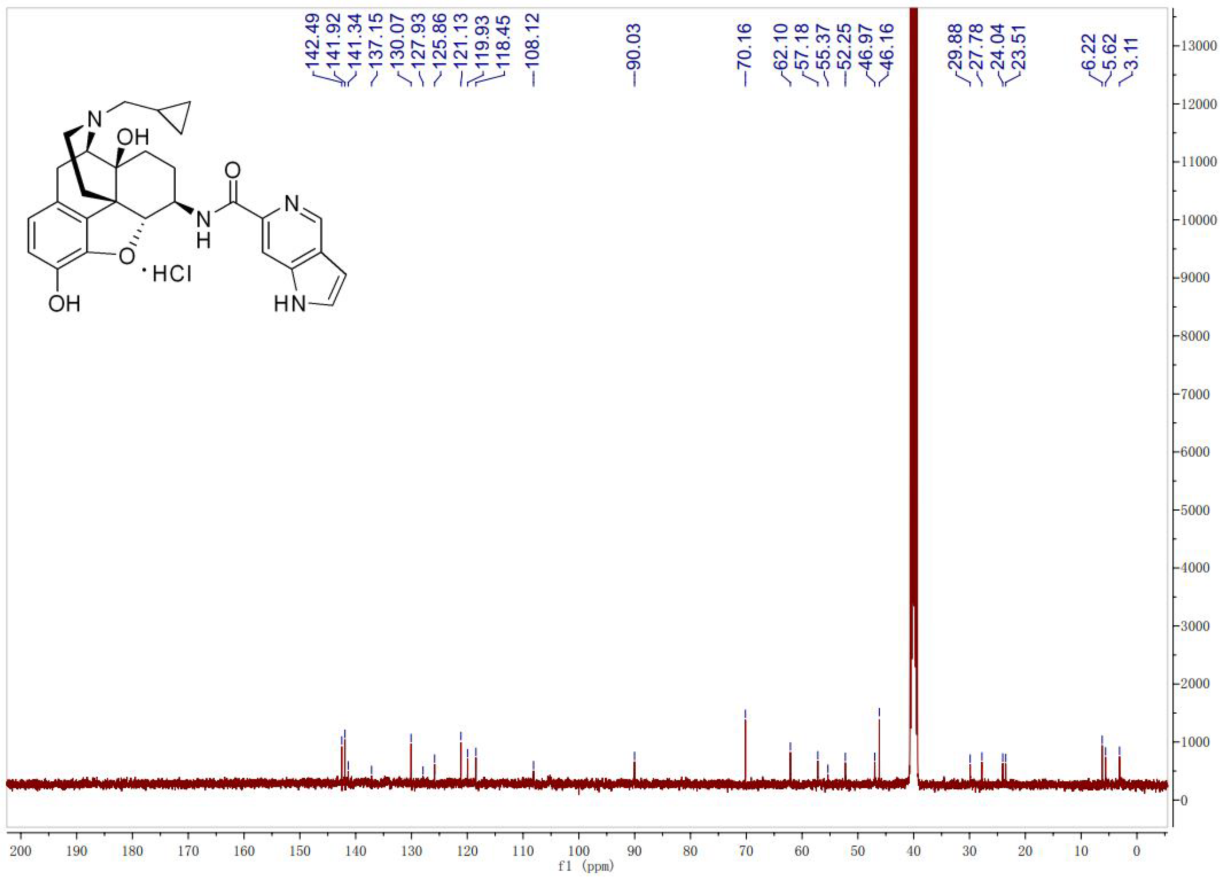
142.49
141.92
141.34
137.15
130.07
127.93
125.86
121.13
119.93
118.45
108.12
90.03
70.16
62.10
57.18
55.37
52.25
46.97
46.16
29.88
27.78
24.04
23.51
6.22
5.62
3.11
OH
O
N
N
H
O
· HCl
OH
N
HN
f1 (ppm)

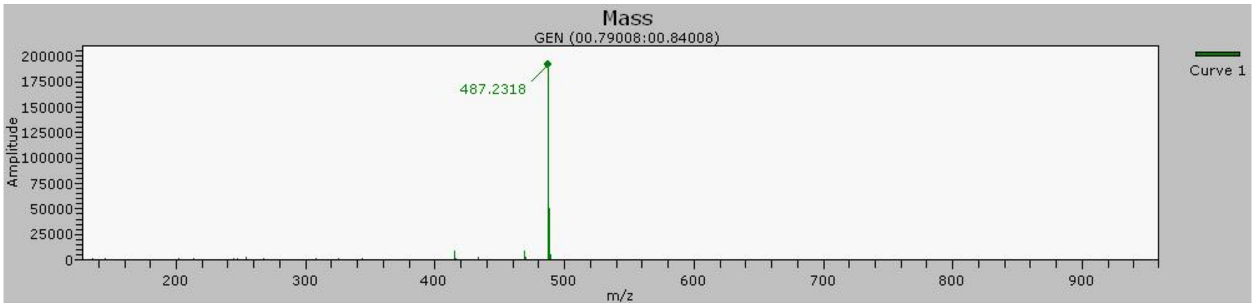
Mass
GEN (00.79008:00.84008)
487.2318
Amplitude
m/z
Curve 1

17-Cyclopropylmethyl-3,14β-dihydro-4,5α-epoxy-6α-[1H-pyrrolo[2,3-b]pyridine-6-carboxamide]morphinan Hydrochloride (**15**)

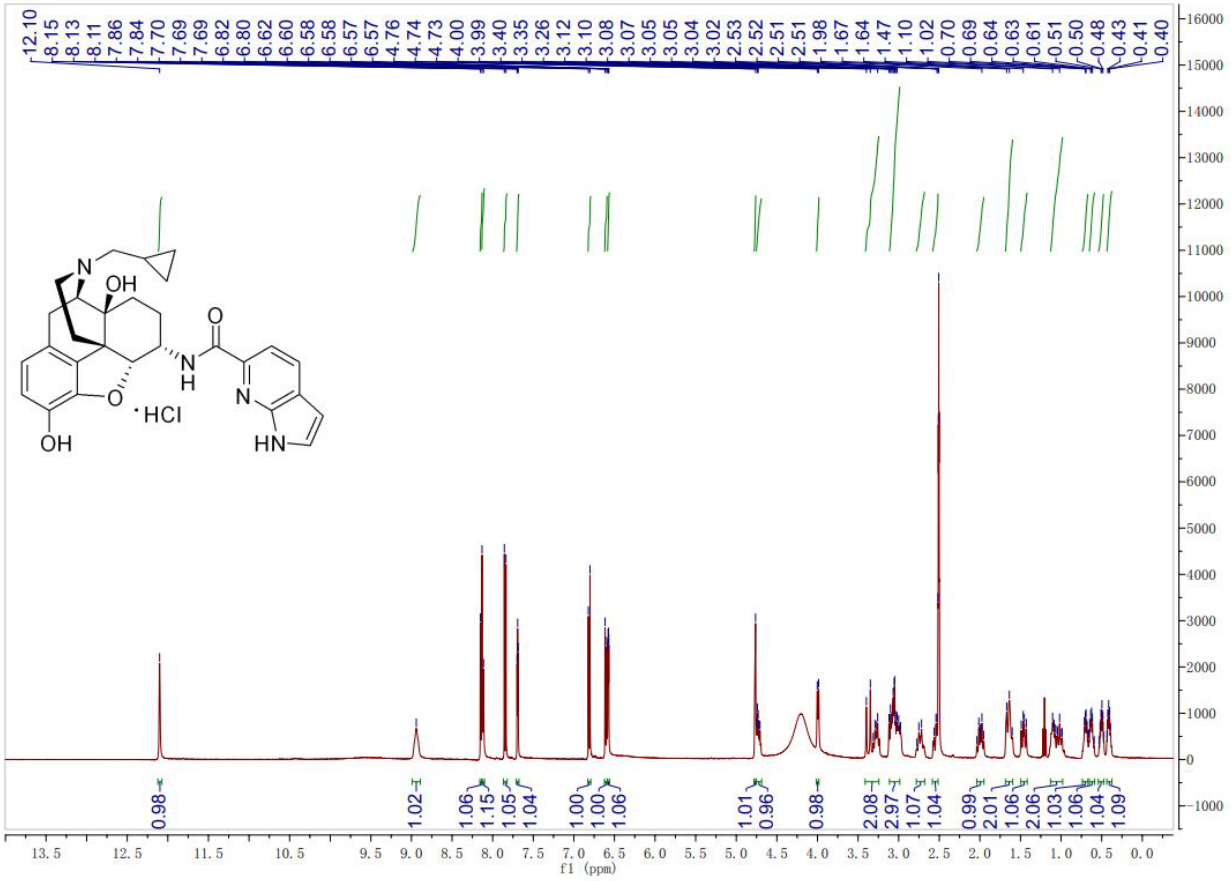

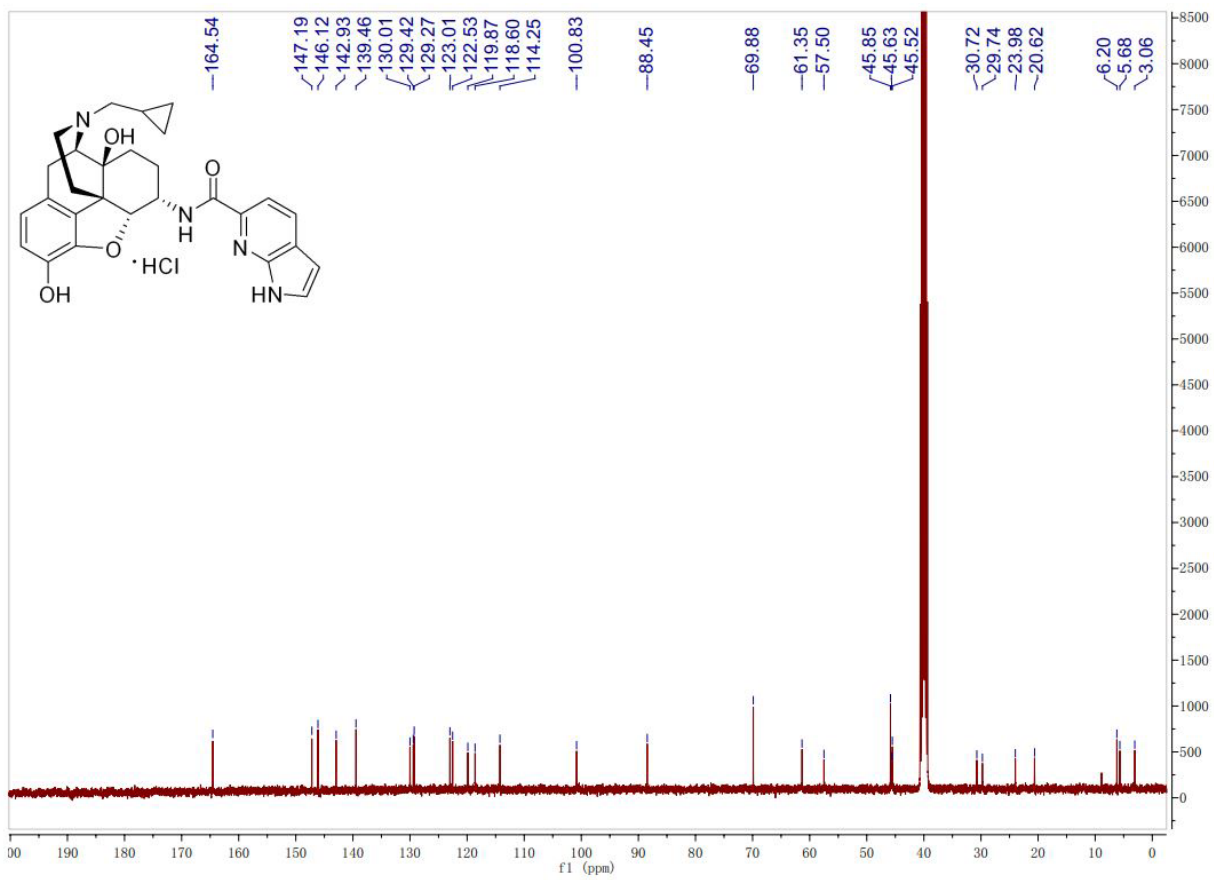

164.54
147.19
146.12
142.93
139.46
130.01
129.42
129.27
123.01
122.53
119.87
118.60
114.25
100.83
88.45
69.88
61.35
57.50
45.85
45.63
45.52
30.72
29.74
23.98
20.62
6.20
5.68
3.06
N
OH
O
N
H
O
·HCl
N
HN
OH
f1 (ppm)

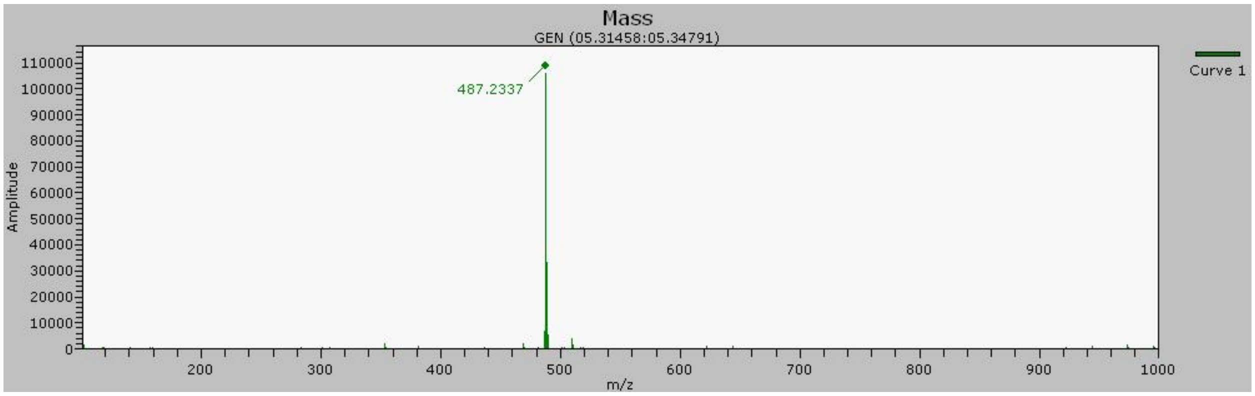

Mass
GEN (05.31458:05.34791)
Curve 1
487.2337
Amplitude
m/z

17-Cyclopropylmethyl-3,14β-dihydro-4,5α-epoxy-6β-[1H-pyrrolo[2,3-b]pyridine-6-carboxamide]morphinan Hydrochloride (**16**)

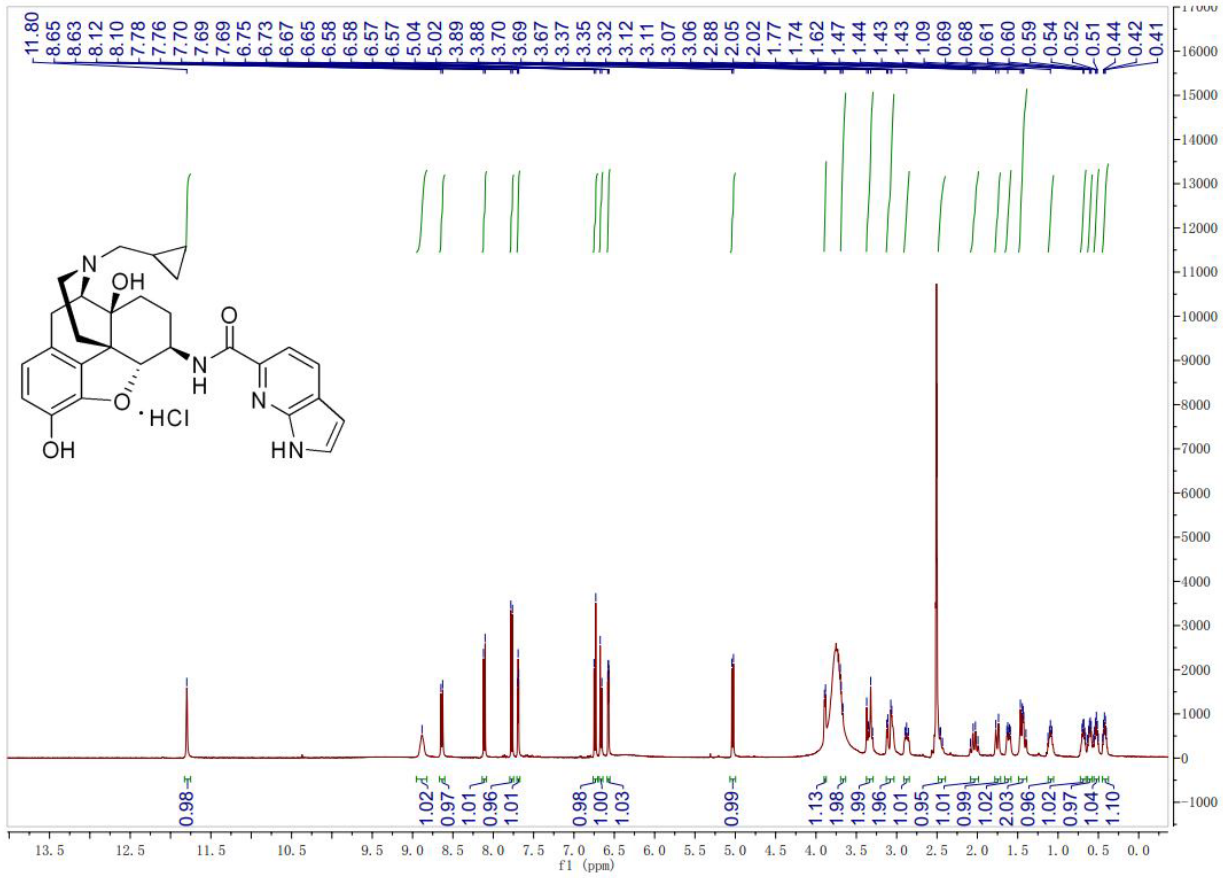

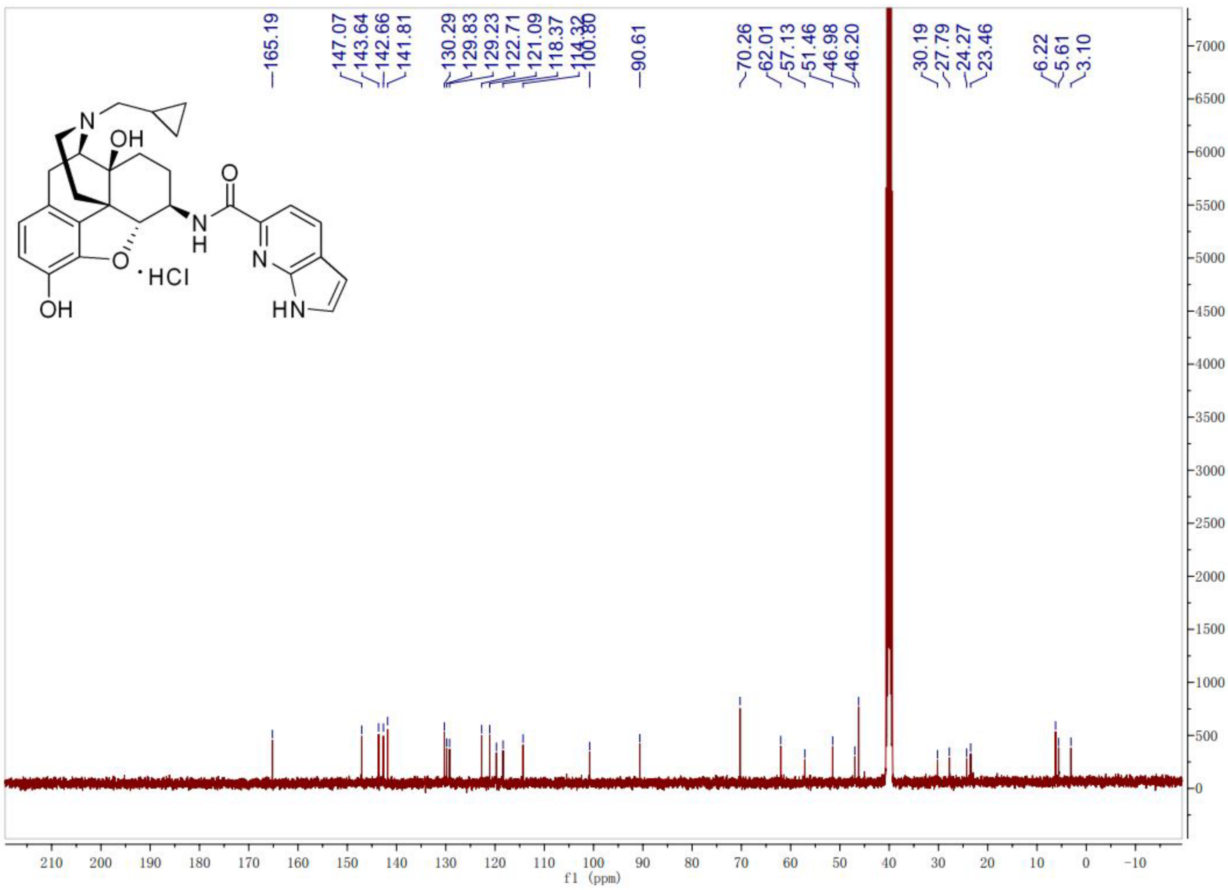
165.19
147.07
143.64
142.66
141.81
130.29
129.83
129.23
122.71
121.09
118.37
114.32
100.80
90.61
70.26
62.01
57.13
51.46
46.98
46.20
30.19
27.79
24.27
23.46
6.22
5.61
3.10
OH
O
N
H
N
HN
O
· HCl
OH
f1 (ppm)

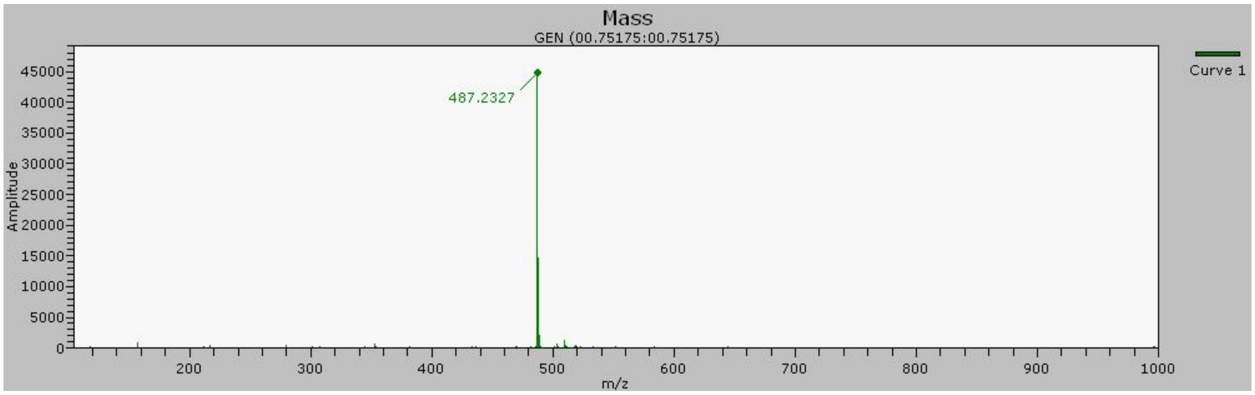
Mass
GEN (00.75175:00.75175)
487.2327
Curve 1
Amplitude
m/z

17-Cyclopropylmethyl-3,14β-dihydro-4,5α-epoxy-6α-[1H-indazole-5-carboxamide]morphinan Hydrochloride (**17**)

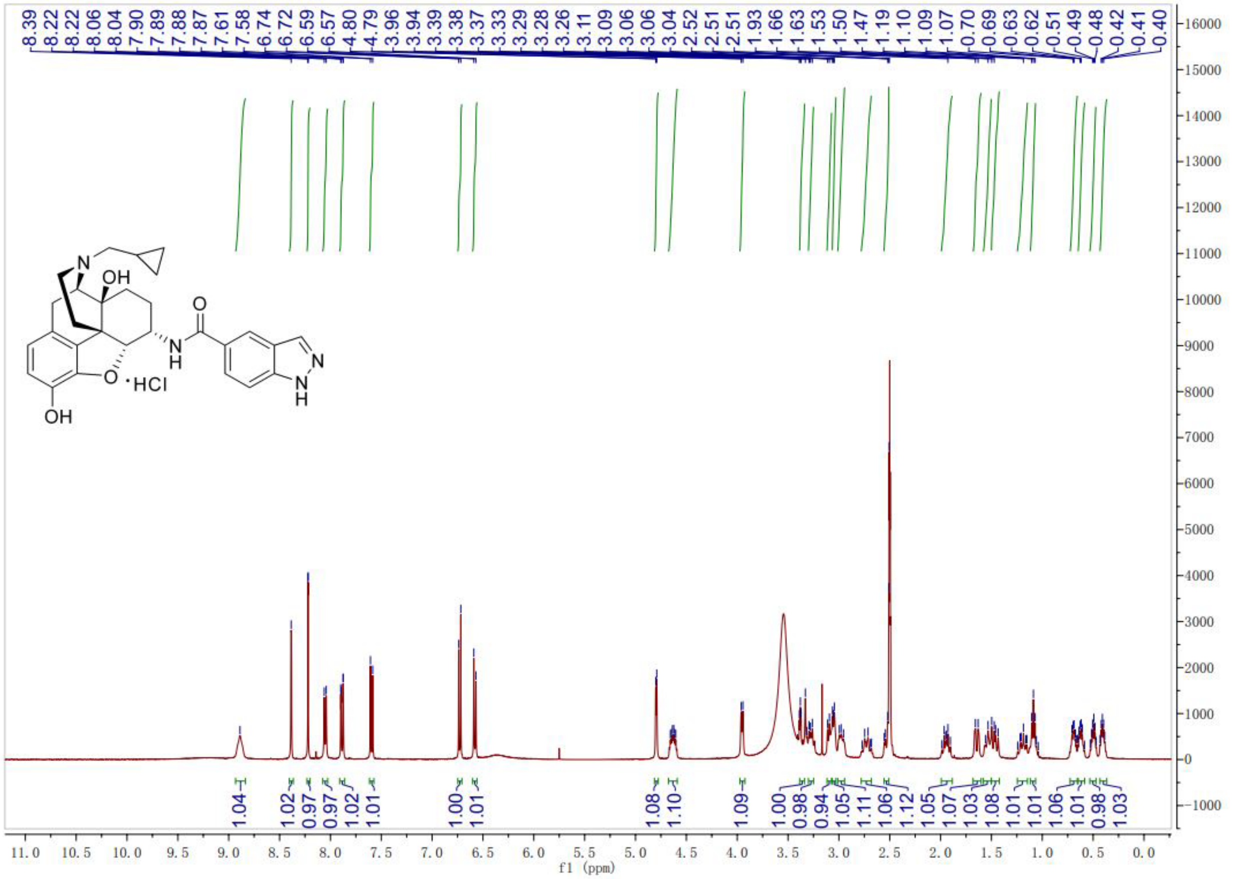

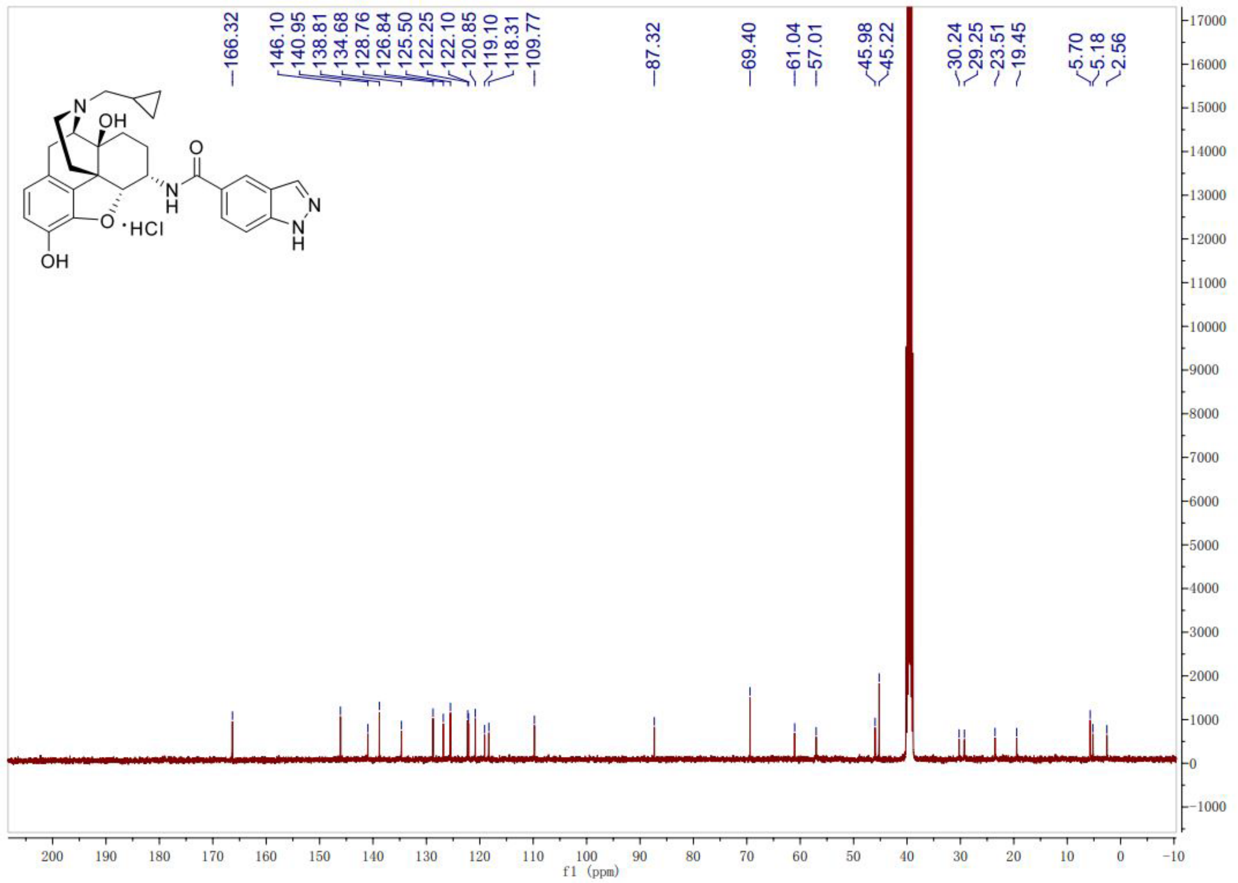

166.32
146.10
140.95
138.81
134.68
128.76
126.84
125.50
122.25
122.10
120.85
119.10
118.31
109.77
87.32
69.40
61.04
57.01
45.98
45.22
30.24
29.25
23.51
19.45
5.70
5.18
2.56
OH
O
N
H
N
N
H
O
·HCl
OH
f1 (ppm)

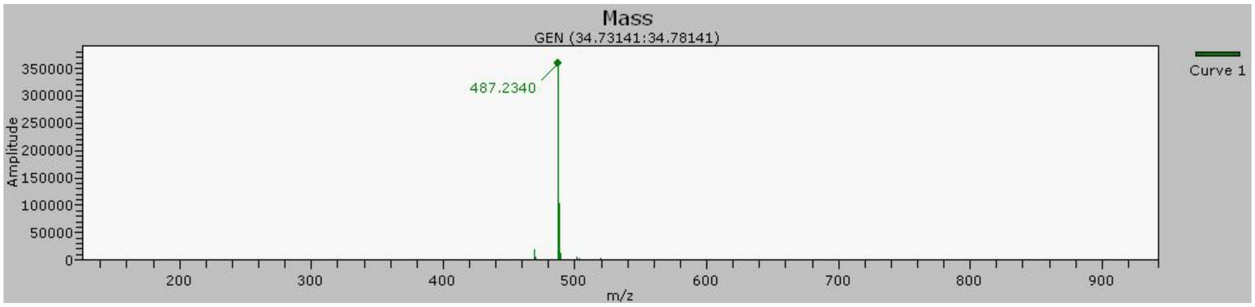

Mass
GEN (34.73141:34.78141)
487.2340
Curve 1
Amplitude
m/z

17-Cyclopropylmethyl-3,14β-dihydro-4,5α-epoxy-6β-[1H-indazole-5-carboxamide]morphinan Hydrochloride (**18**)

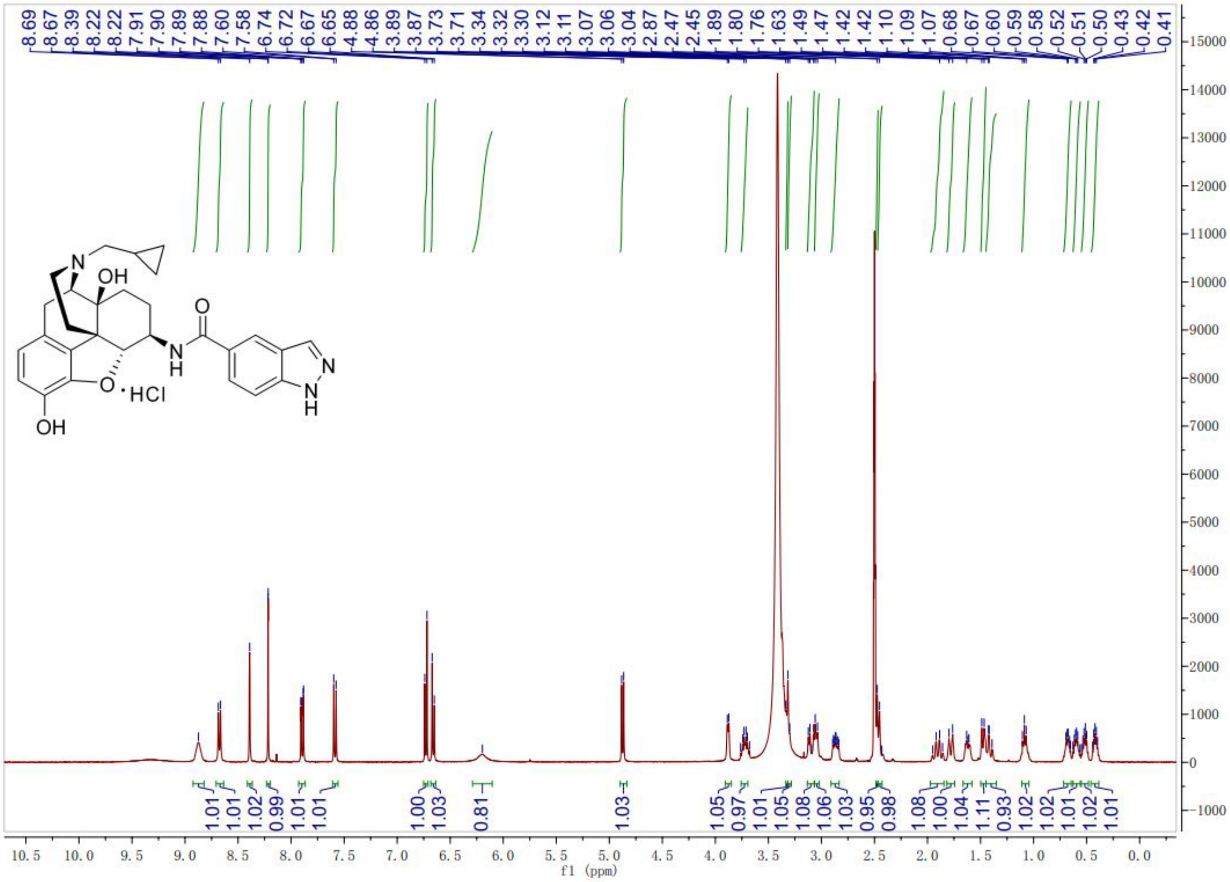

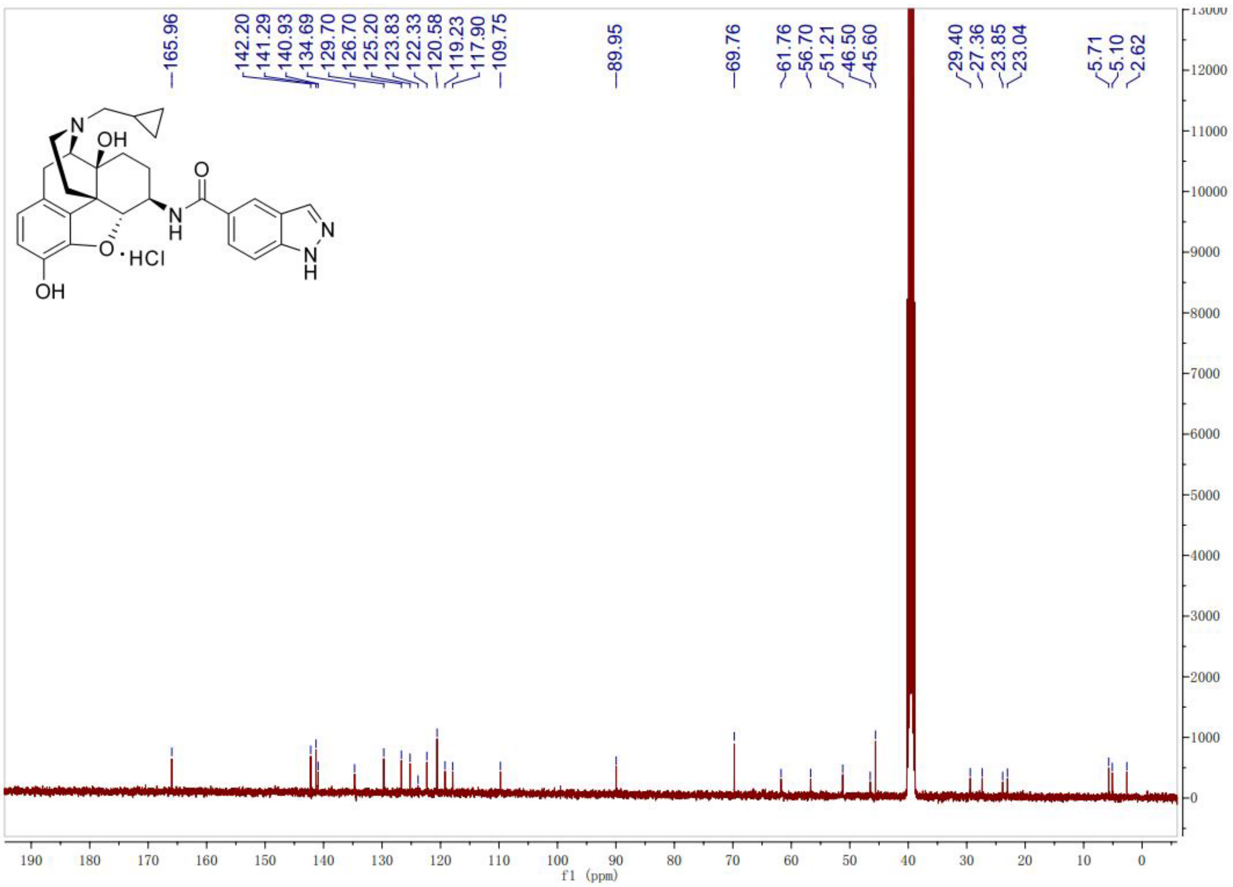
165.96
142.20
141.29
140.93
134.69
129.70
126.70
125.20
123.83
122.33
120.58
119.23
117.90
109.75
89.95
69.76
61.76
56.70
51.21
46.50
45.60
29.40
27.36
23.85
23.04
5.71
5.10
2.62
OH
N
O
N
H
N
N
H
O
· HCl
OH
f1 (ppm)

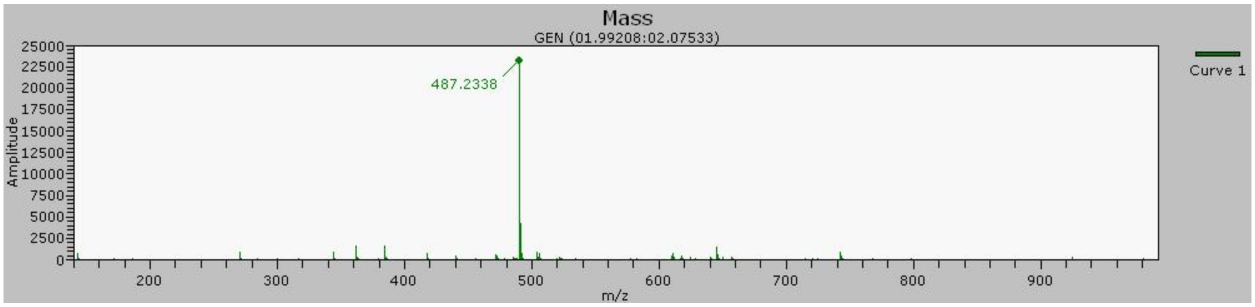
Mass
GEN (01.99208:02.07533)
487.2338
Amplitude
m/z
Curve 1

17-Cyclopropylmethyl-3,14β-dihydro-4,5α-epoxy-6α-[1H-benzo[d]imidazole-5-carboxamide]morphinan Hydrochloride (**19**)

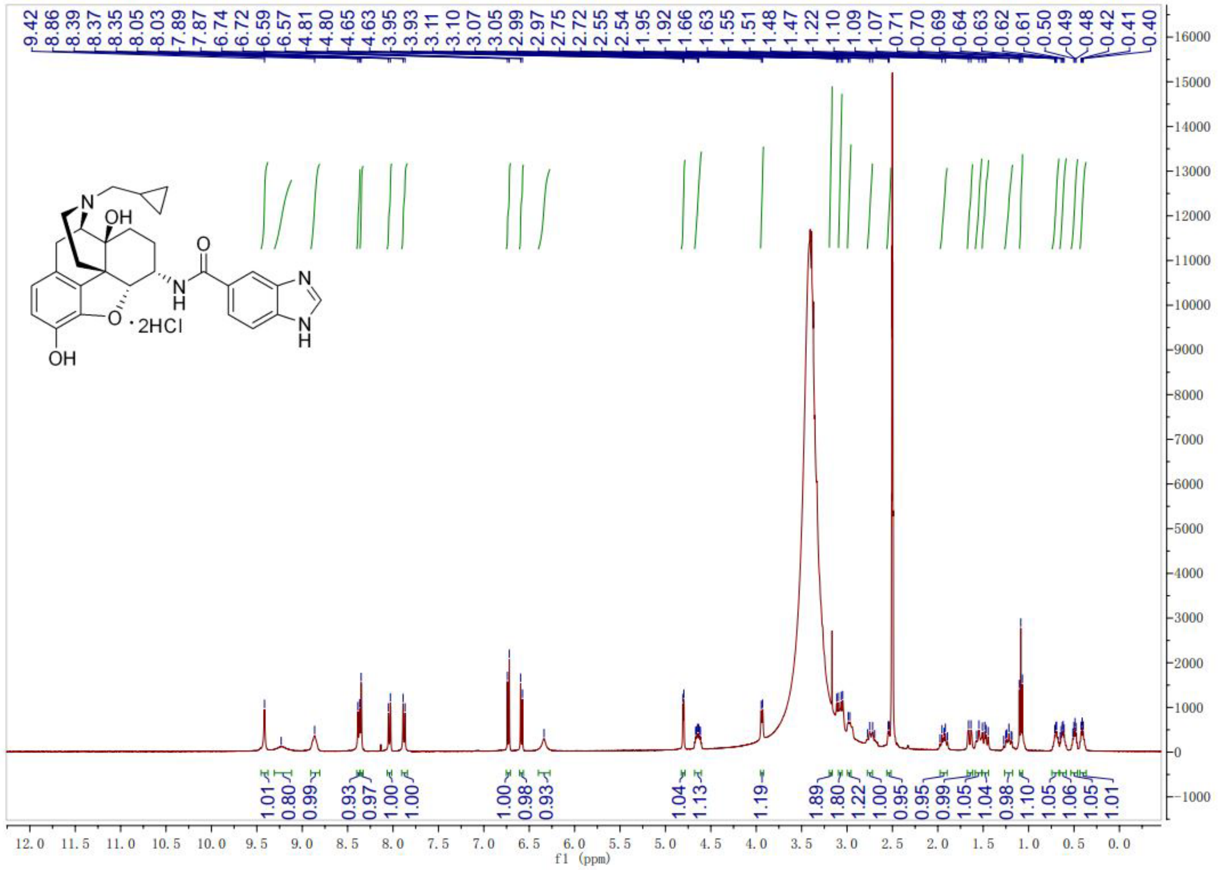

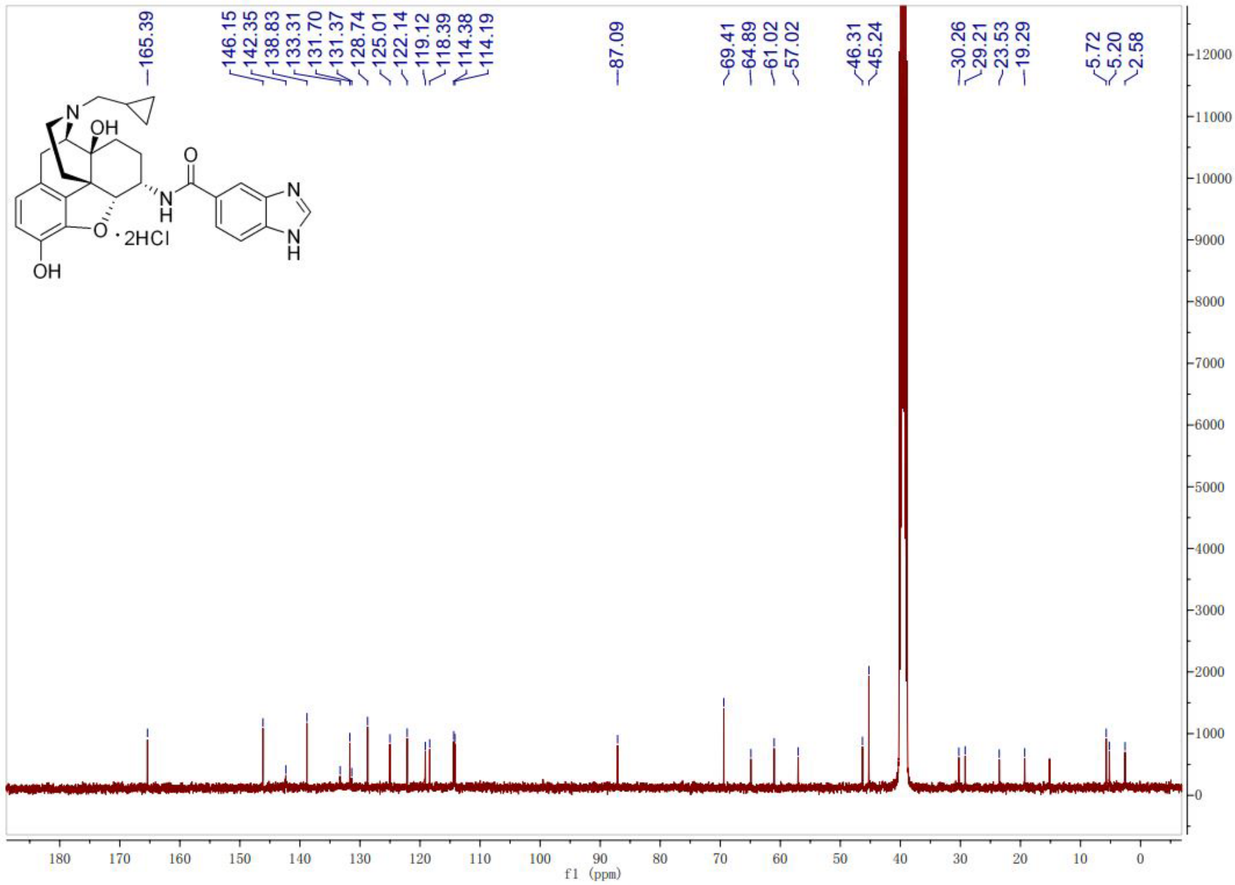

165.39
146.15
142.35
138.83
133.31
131.70
131.37
128.74
125.01
122.14
119.12
118.39
114.38
114.19
87.09
69.41
64.89
61.02
57.02
46.31
45.24
30.26
29.21
23.53
19.29
5.72
5.20
2.58
·2HCl
f1 (ppm)

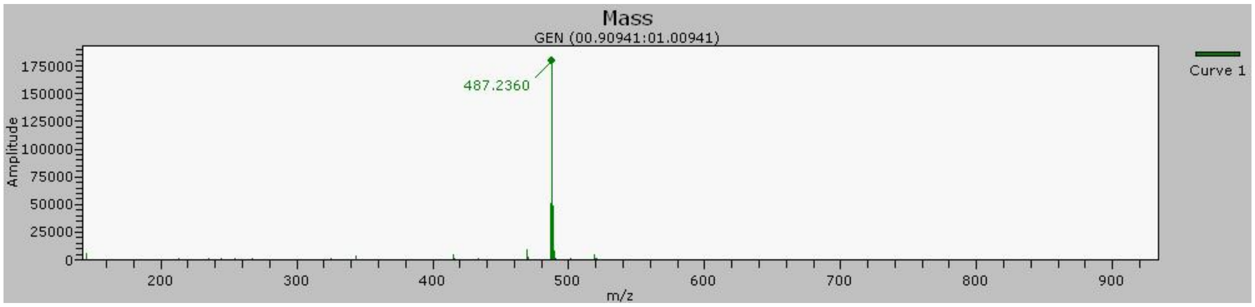

Mass
GEN (00.90941:01.00941)
487.2360
Curve 1
Amplitude
m/z

17-Cyclopropylmethyl-3,14β-dihydro-4,5α-epoxy-6β-[1H-benzo[d]imidazole-5-carboxamide]morphinan Hydrochloride (**20**)

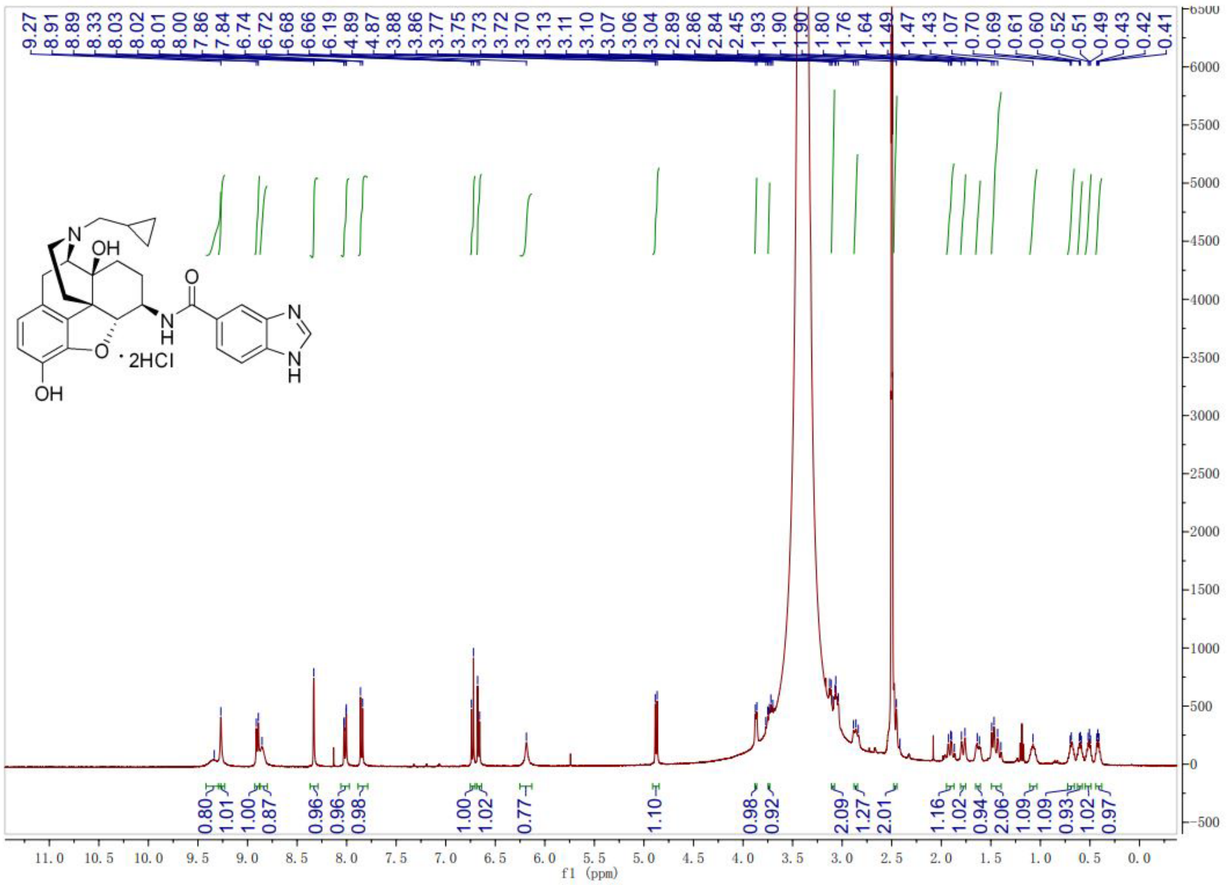

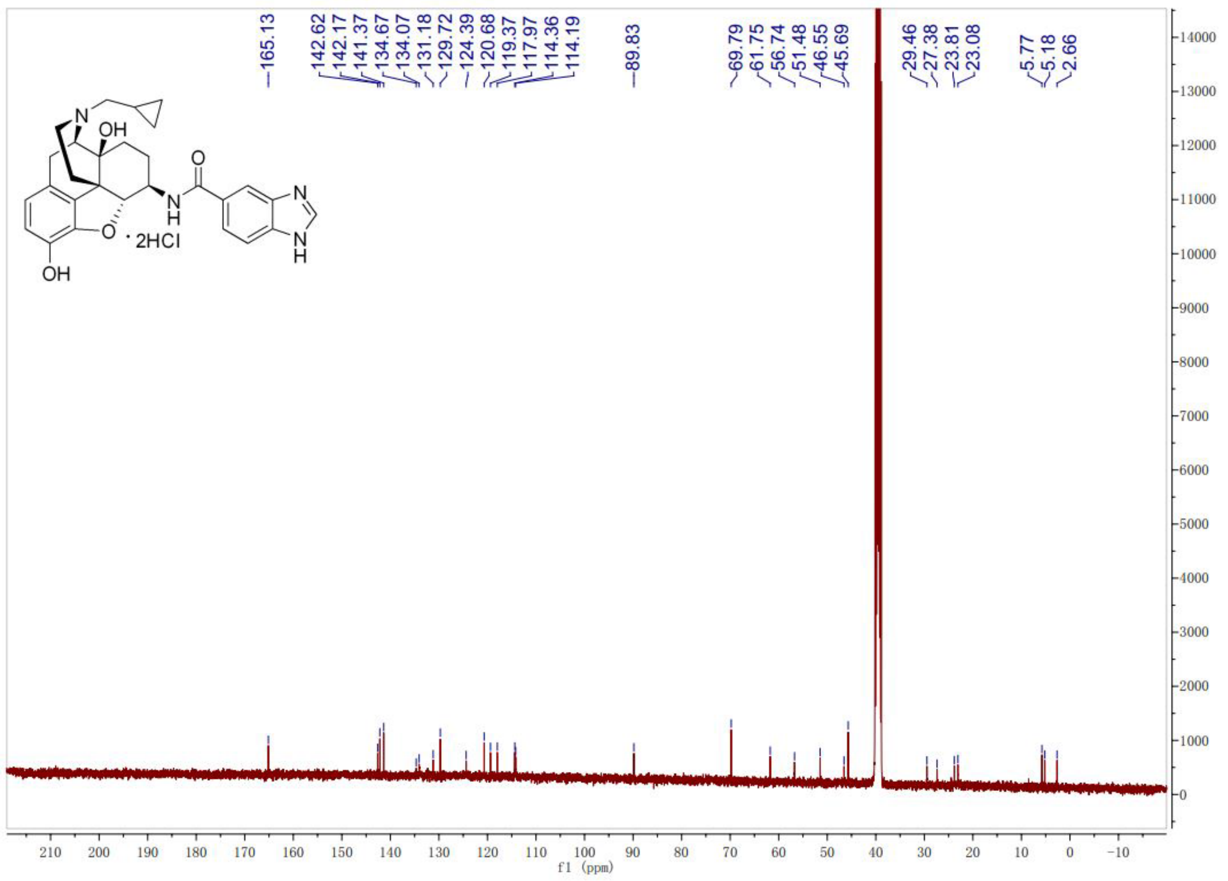

165.13
142.62
142.17
141.37
134.67
134.07
131.18
129.72
124.39
120.68
119.37
117.97
114.36
114.19
89.83
69.79
61.75
56.74
51.48
46.55
45.69
29.46
27.38
23.81
23.08
5.77
5.18
2.66
OH
O
N
H
N
N
H
O
· 2HCl
OH
f1 (ppm)

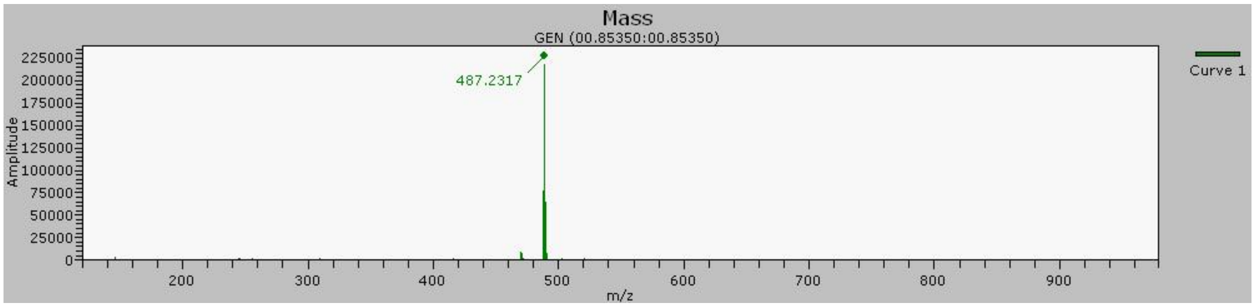

Mass
GEN (00.85350:00.85350)
487.2317
Curve 1
Amplitude
m/z

17-Cyclopropylmethyl-3,14β-dihydro-4,5α-epoxy-6α-[1H-pyrrolo[3,2-b]pyridine-5-carboxamide]morphinan Hydrochloride (**21**)

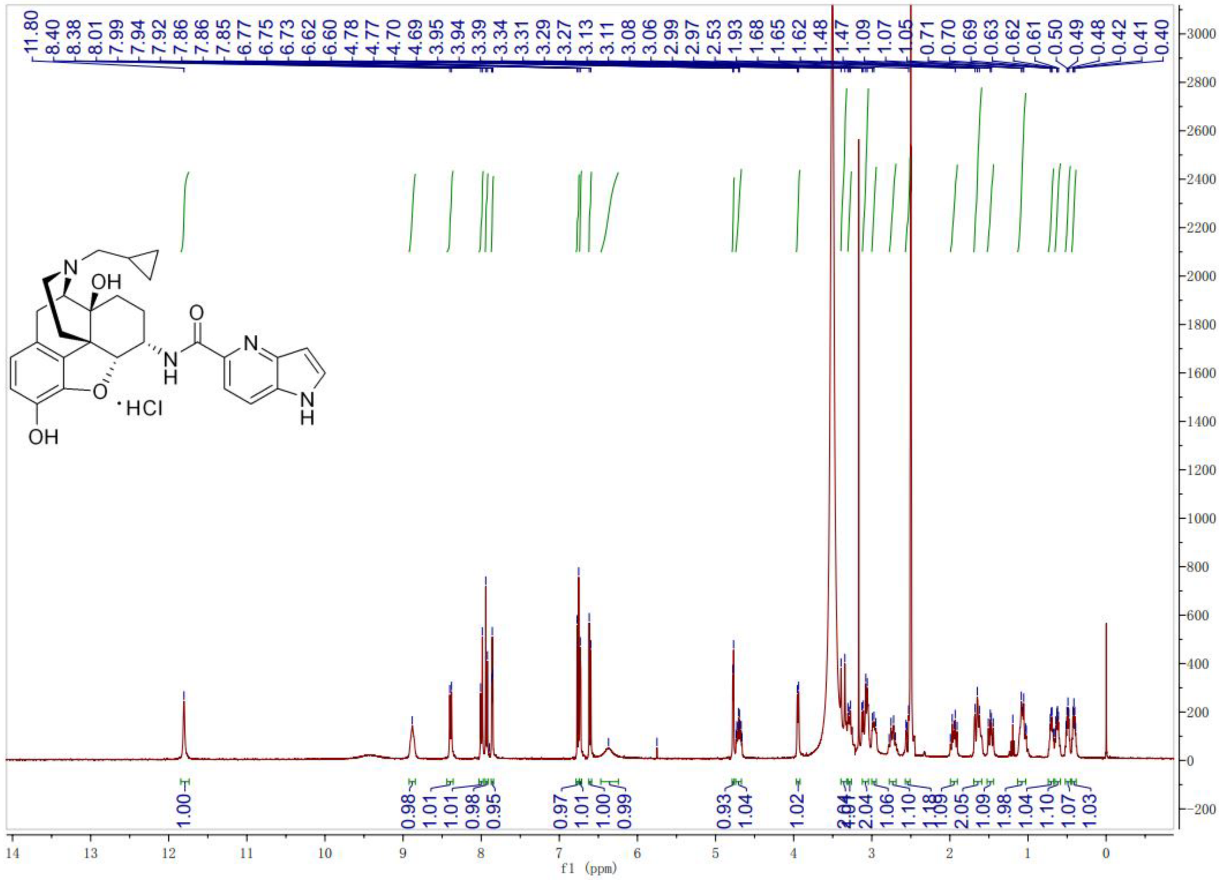

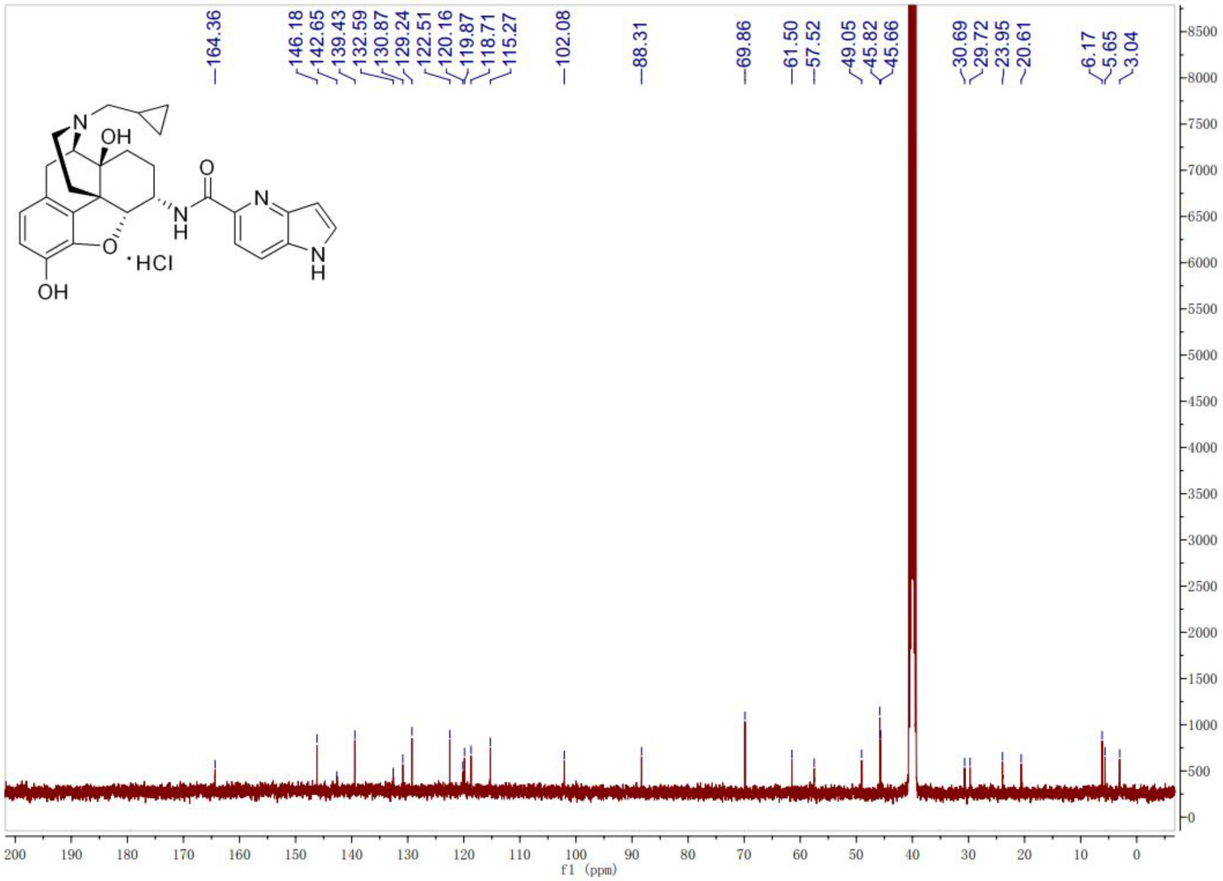
164.36
146.18
142.65
139.43
132.59
130.87
129.24
122.51
120.16
119.87
118.71
115.27
102.08
88.31
69.86
61.50
57.52
49.05
45.82
45.66
30.69
29.72
23.95
20.61
6.17
5.65
3.04
OH
O
N
H
N
NH
O
OH
·HCl
f1 (ppm)

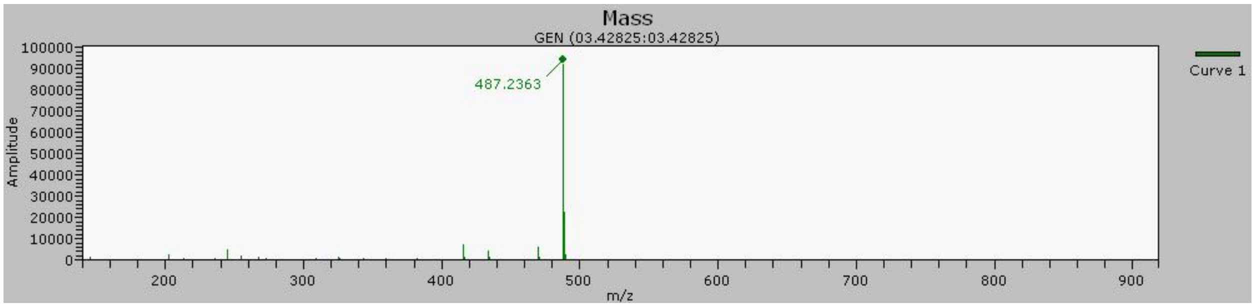
Mass
GEN (03.42825:03.42825)
487.2363
Amplitude
m/z
Curve 1

17-Cyclopropylmethyl-3,14β-dihydro-4,5α-epoxy-6β-[1H-pyrrolo[3,2-b]pyridine-5-carboxamide]morphinan Hydrochloride (**22**)

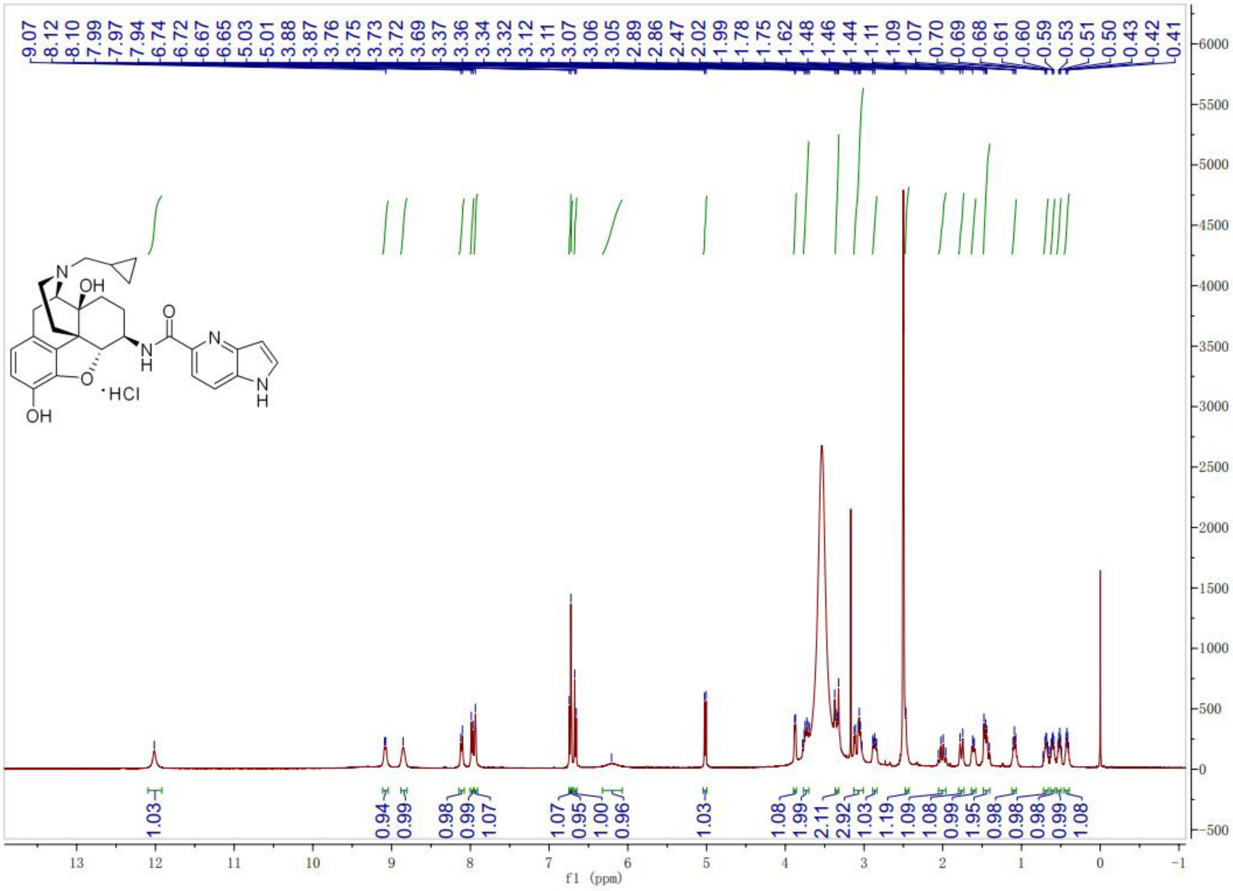

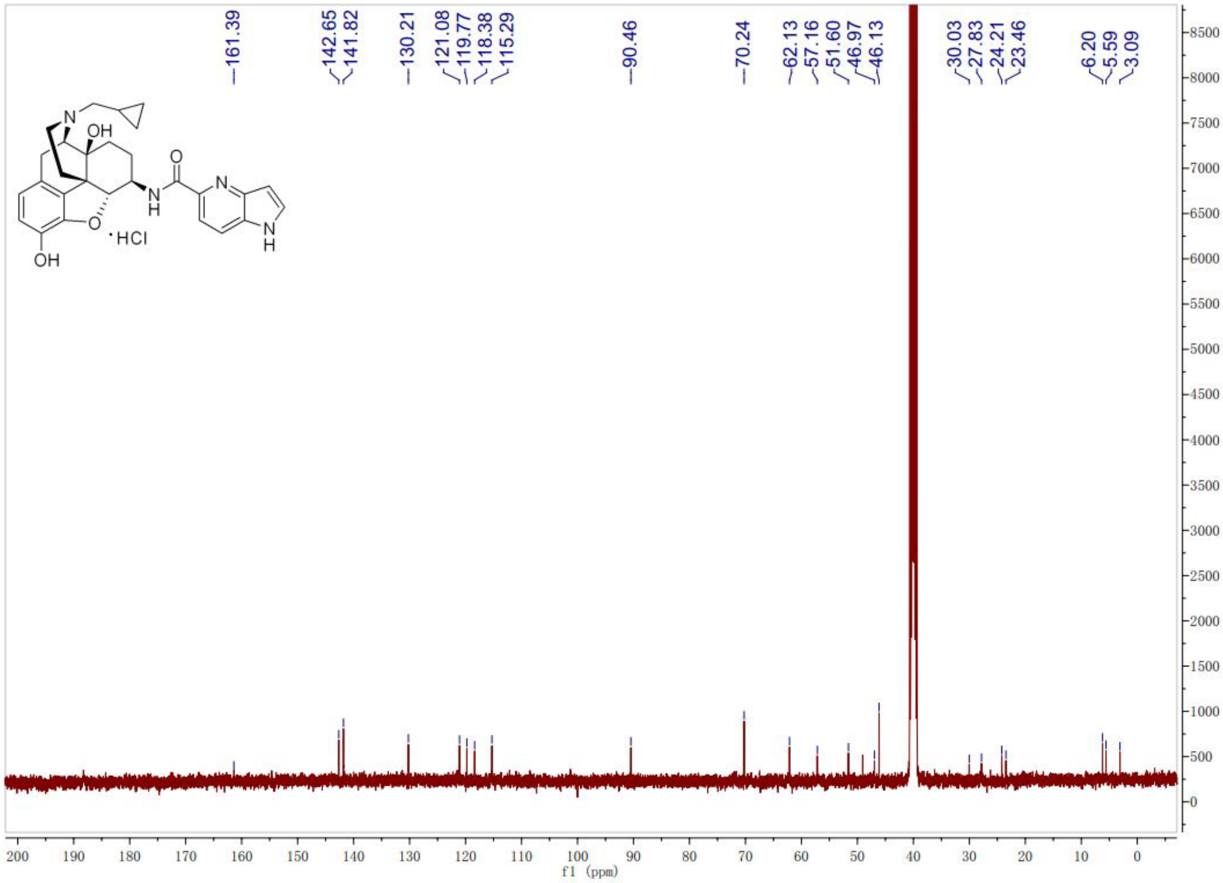

161.39
142.65
141.82
130.21
121.08
119.77
118.38
115.29
90.46
70.24
62.13
57.16
51.60
46.97
46.13
30.03
27.83
24.21
23.46
6.20
5.59
3.09
N
OH
O
N
H
N
N
H
O
·HCl
OH
f1 (ppm)

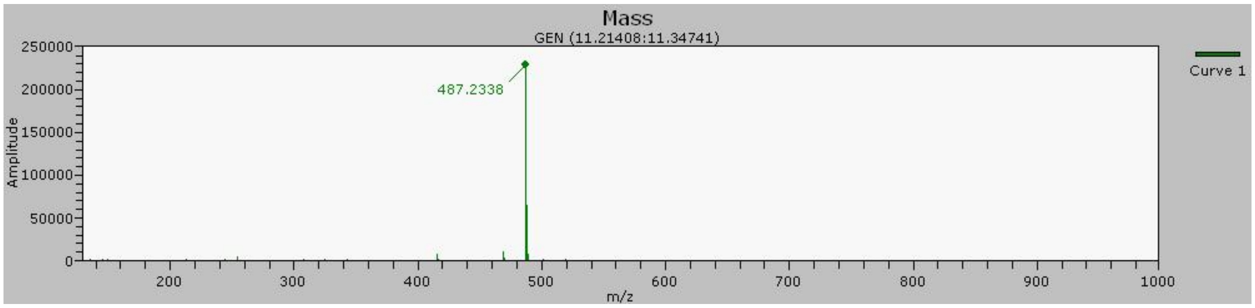

Mass
GEN (11.21408:11.34741)
487.2338
Amplitude
m/z
Curve 1

17-Cyclopropylmethyl-3,14β-dihydro-4,5α-epoxy-6α-[1H-pyrrolo[2,3-c]pyridine-5-carboxamide]morphinan Hydrochloride (**23**)

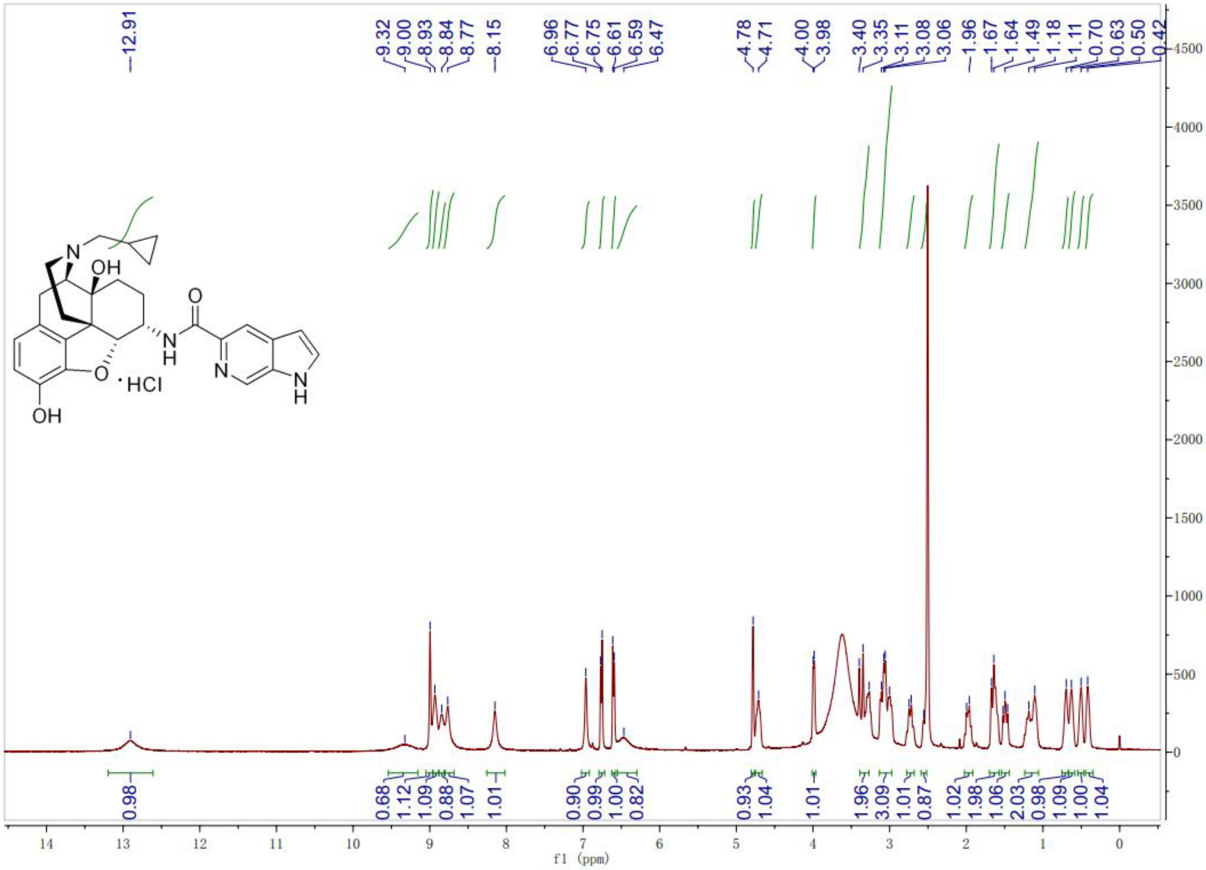

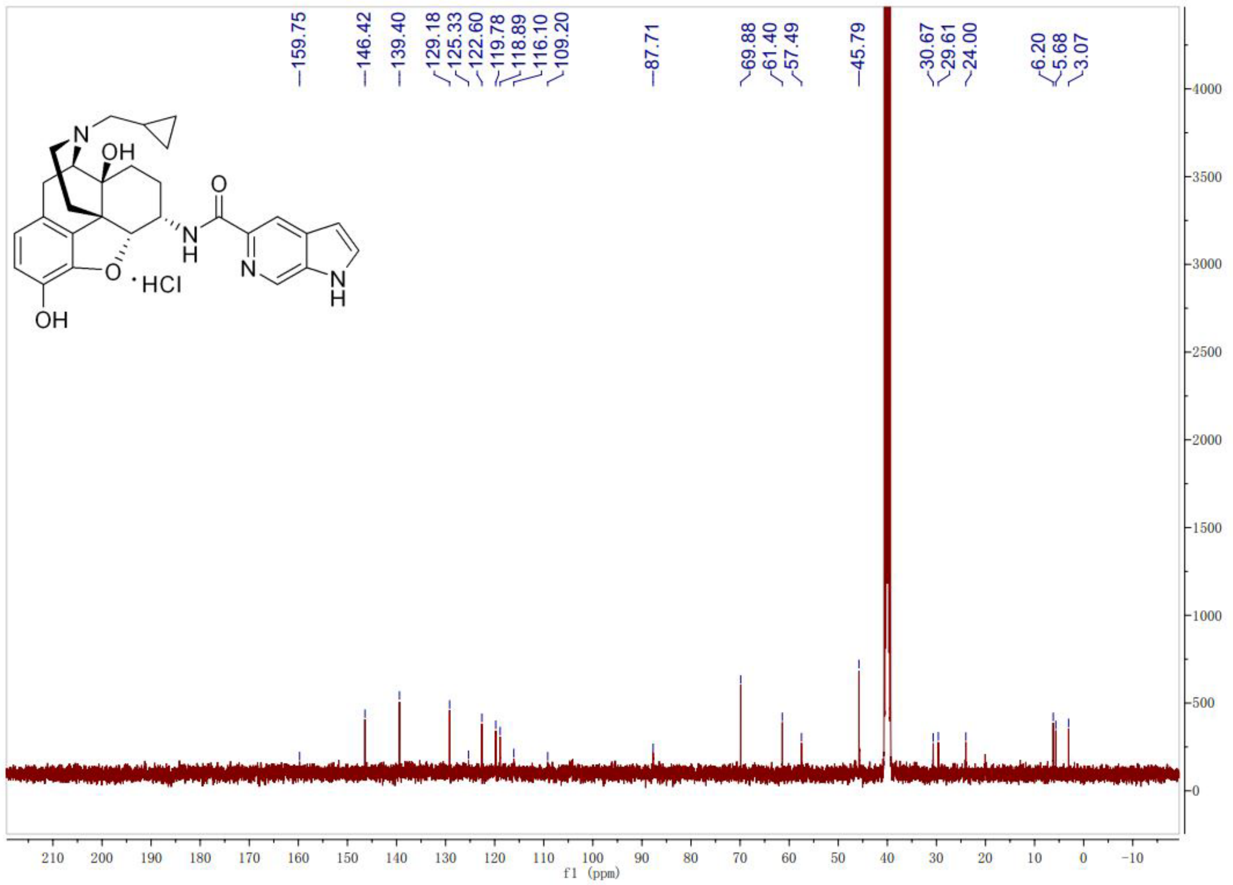

159.75
146.42
139.40
129.18
125.33
122.60
119.78
118.89
116.10
109.20
87.71
69.88
61.40
57.49
45.79
30.67
29.61
24.00
6.20
5.68
3.07
OH
N
O
N
H
O
·HCl
N
N
H
OH
f1 (ppm)

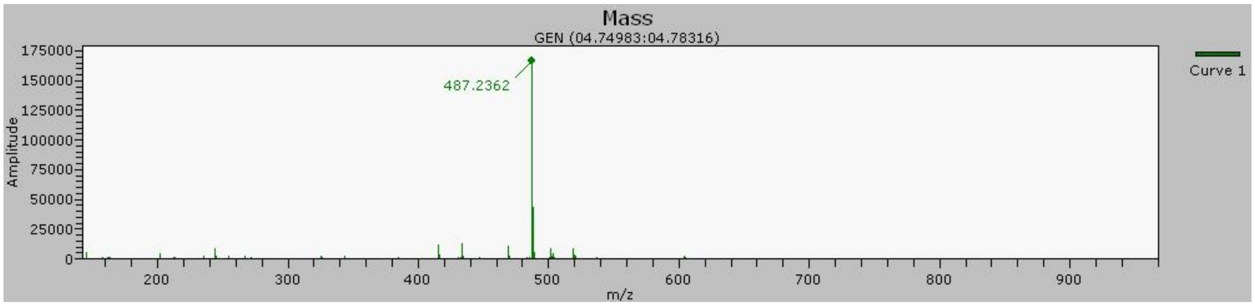

Mass
GEN (04.74983:04.78316)
487.2362
Amplitude
m/z
Curve 1

17-Cyclopropylmethyl-3,14β-dihydro-4,5α-epoxy-6β-[1H-pyrrolo[2,3-c]pyridine-5-carboxamide]morphinan Hydrochloride (**24**)

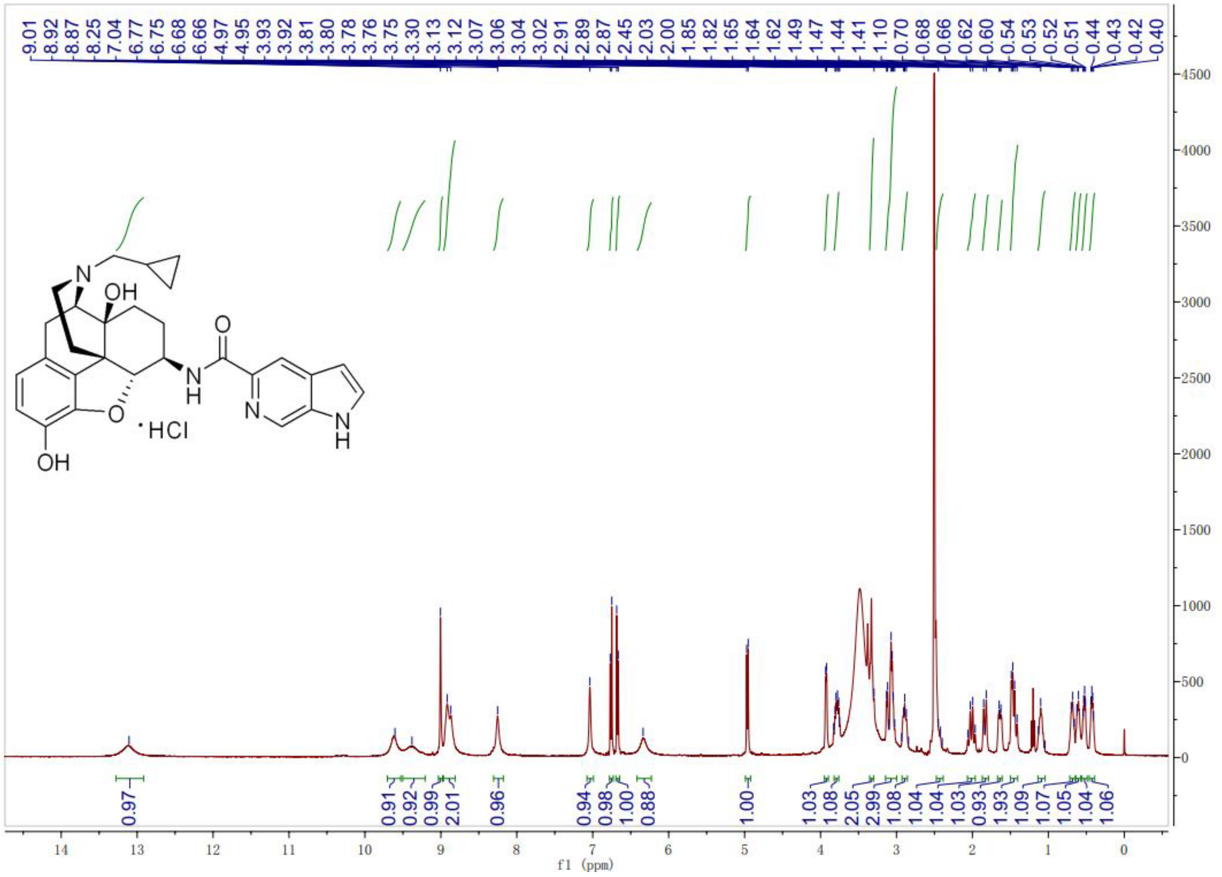

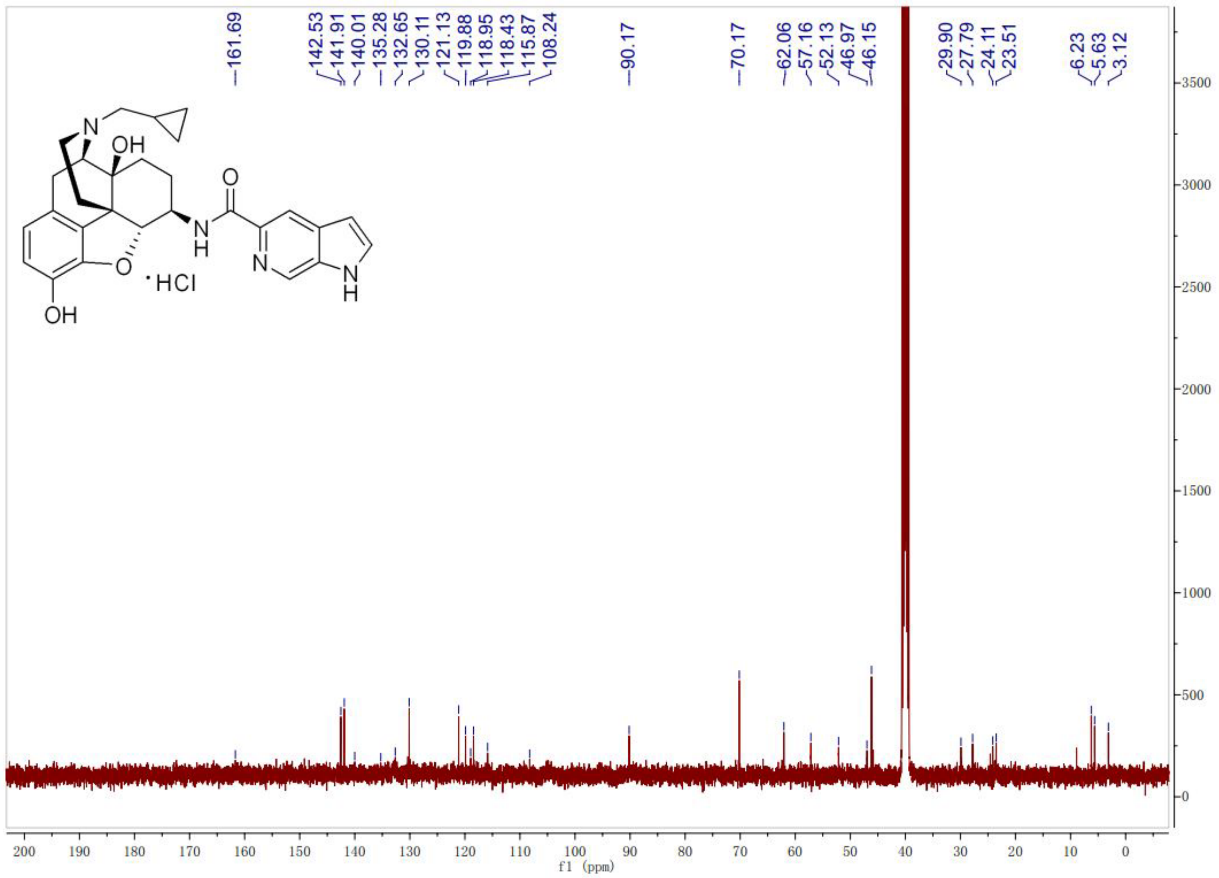
161.69
142.53
141.91
140.01
135.28
132.65
130.11
121.13
119.88
118.95
118.43
115.87
108.24
90.17
70.17
62.06
57.16
52.13
46.97
46.15
29.90
27.79
24.11
23.51
6.23
5.63
3.12
N
OH
O
N
H
N
N
H
O
·HCl
OH
f1 (ppm)

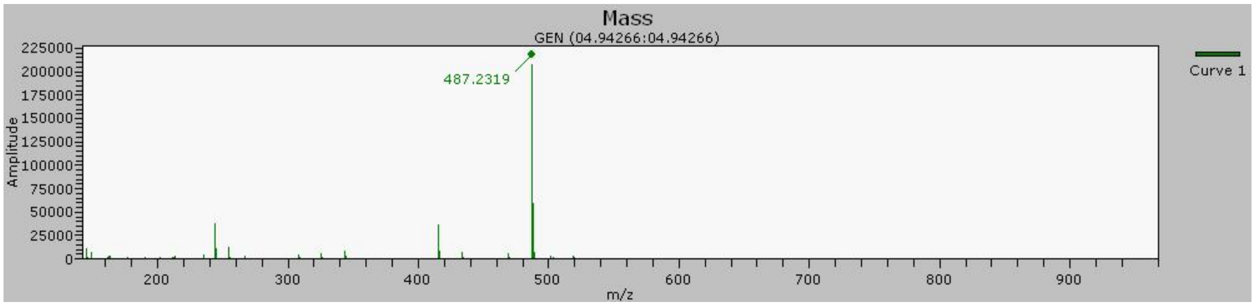
Mass
GEN (04.94266:04.94266)
487.2319
Amplitude
m/z
Curve 1

17-Cyclopropylmethyl-3,14β-dihydro-4,5α-epoxy-6α-[1H-pyrrolo[2,3-b]pyridine-5-carboxamide]morphinan Hydrochloride (**25**)

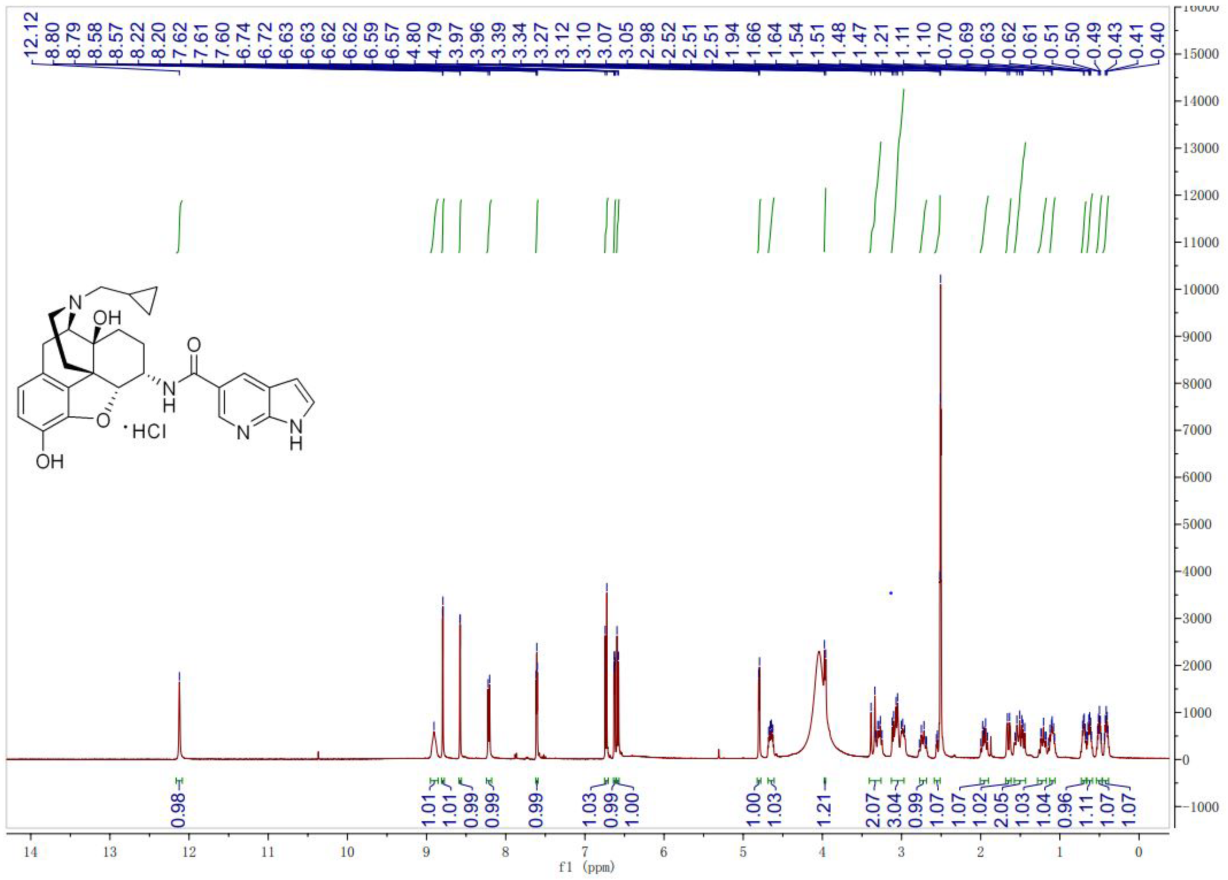

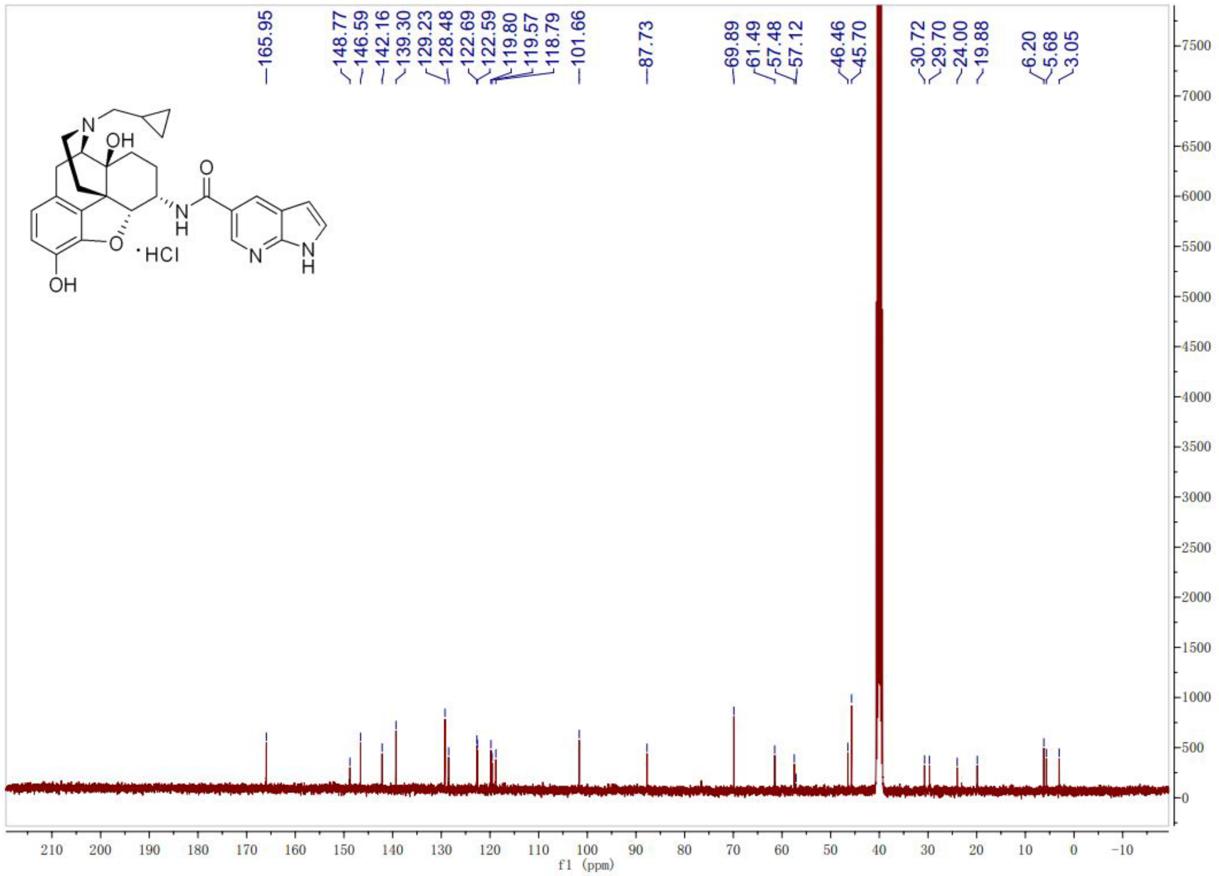
165.95
148.77
146.59
142.16
139.30
129.23
128.48
122.69
122.59
119.80
119.57
118.79
101.66
87.73
69.89
61.49
57.48
57.12
46.46
45.70
30.72
29.70
24.00
19.88
6.20
5.68
3.05
N
OH
O
N
H
O
·HCl
N
N
H
OH
f1 (ppm)

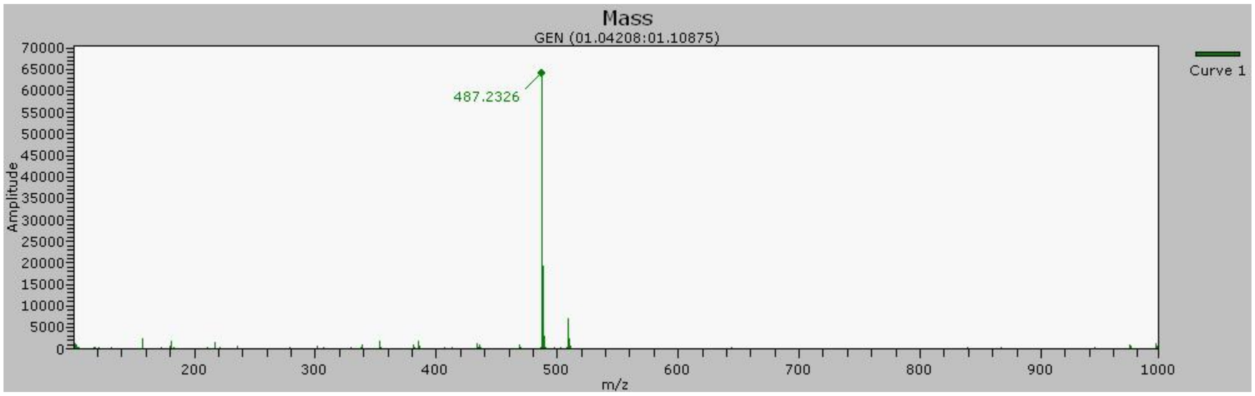
Mass
GEN (01.04208:01.10875)
487.2326
Amplitude
m/z
Curve 1

17-Cyclopropylmethyl-3,14β-dihydro-4,5α-epoxy-6β-[1H-pyrrolo[2,3-b]pyridine-5-carboxamide]morphinan Hydrochloride (**26**)

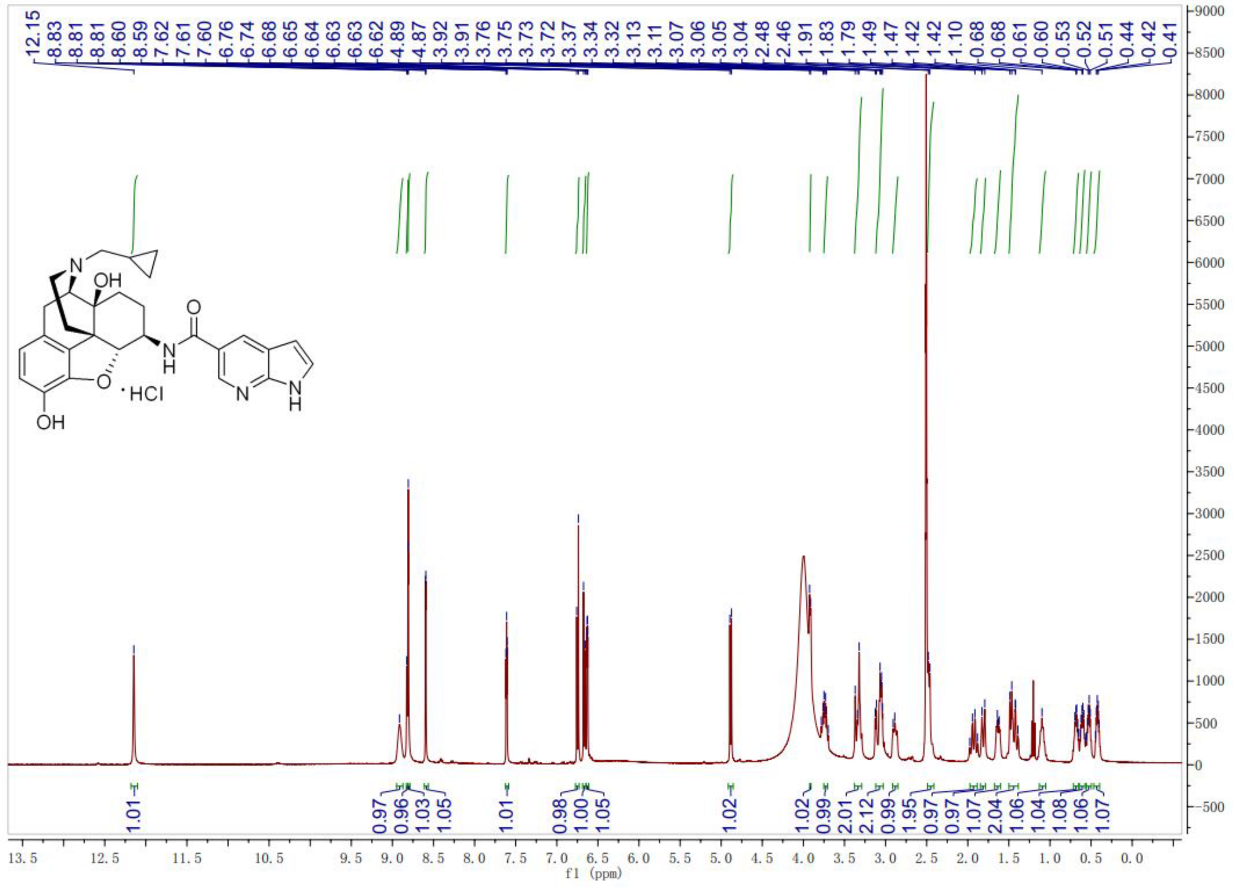

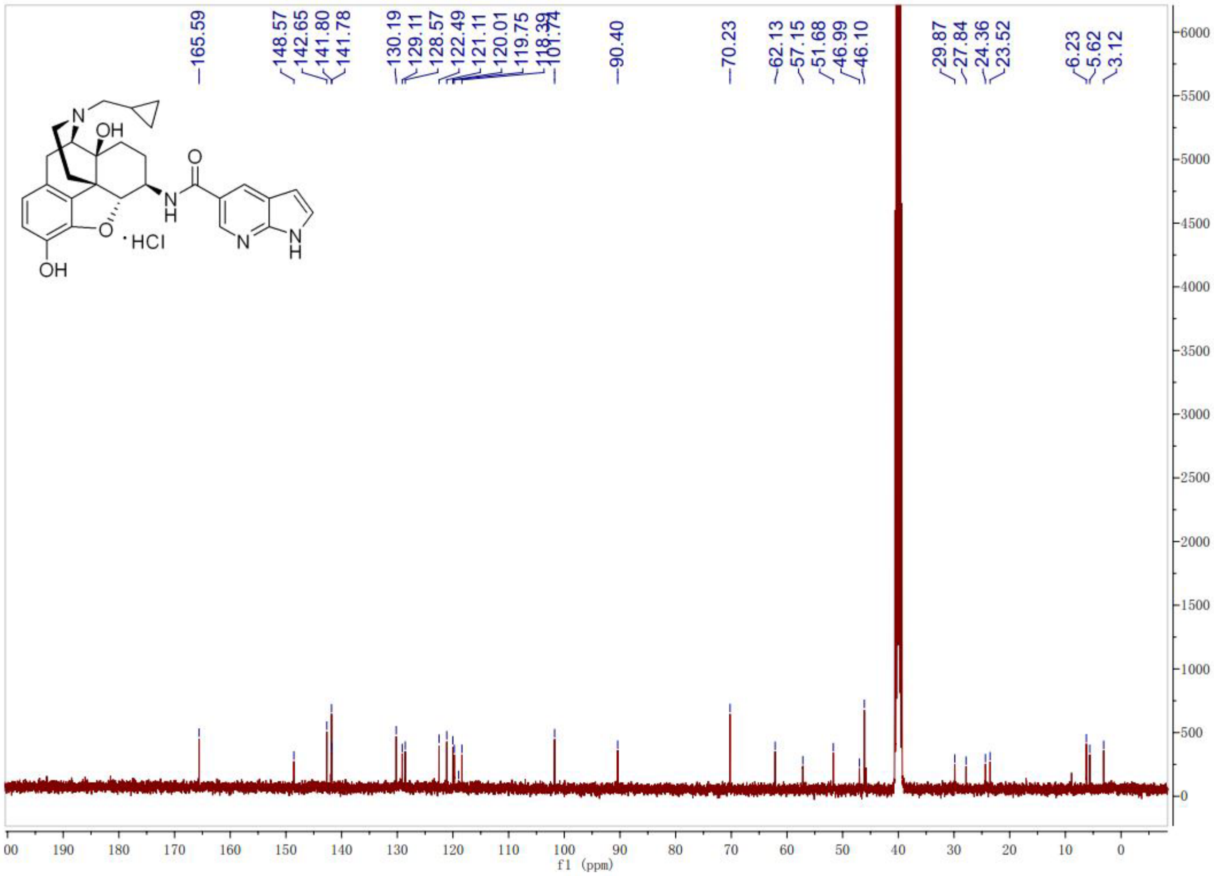
165.59
148.57
142.65
141.80
141.78
130.19
129.11
128.57
122.49
121.11
120.01
119.75
101.74
90.40
70.23
62.13
57.15
51.68
46.99
46.10
29.87
27.84
24.36
23.52
6.23
5.62
3.12
OH
N
O
H
·HCl
f1 (ppm)

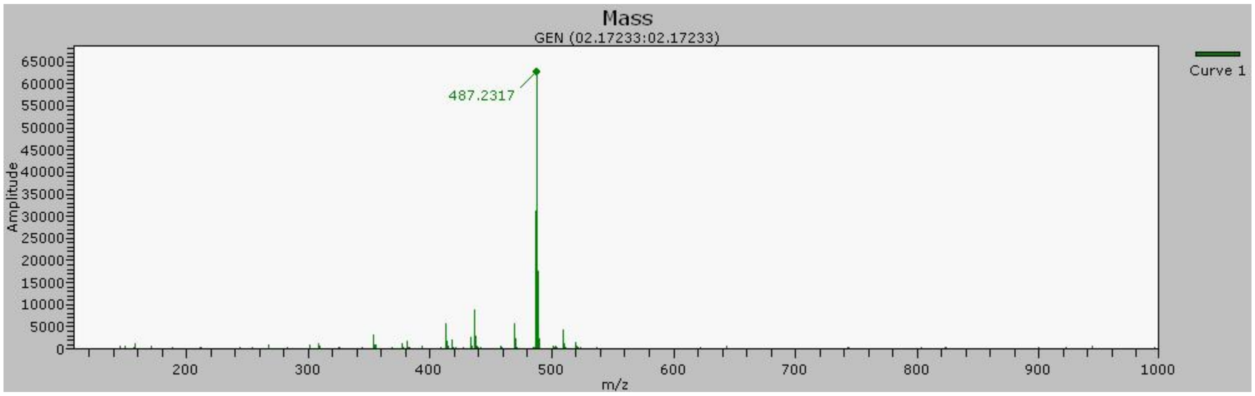
Mass
GEN (02.17233:02.17233)
487.2317
Curve 1
Amplitude
m/z

17-Cyclopropylmethyl-3,14β-dihydro-4,5α-epoxy-6α-[1H-indazole-4-carboxamide]morphinan Hydrochloride (**27**)

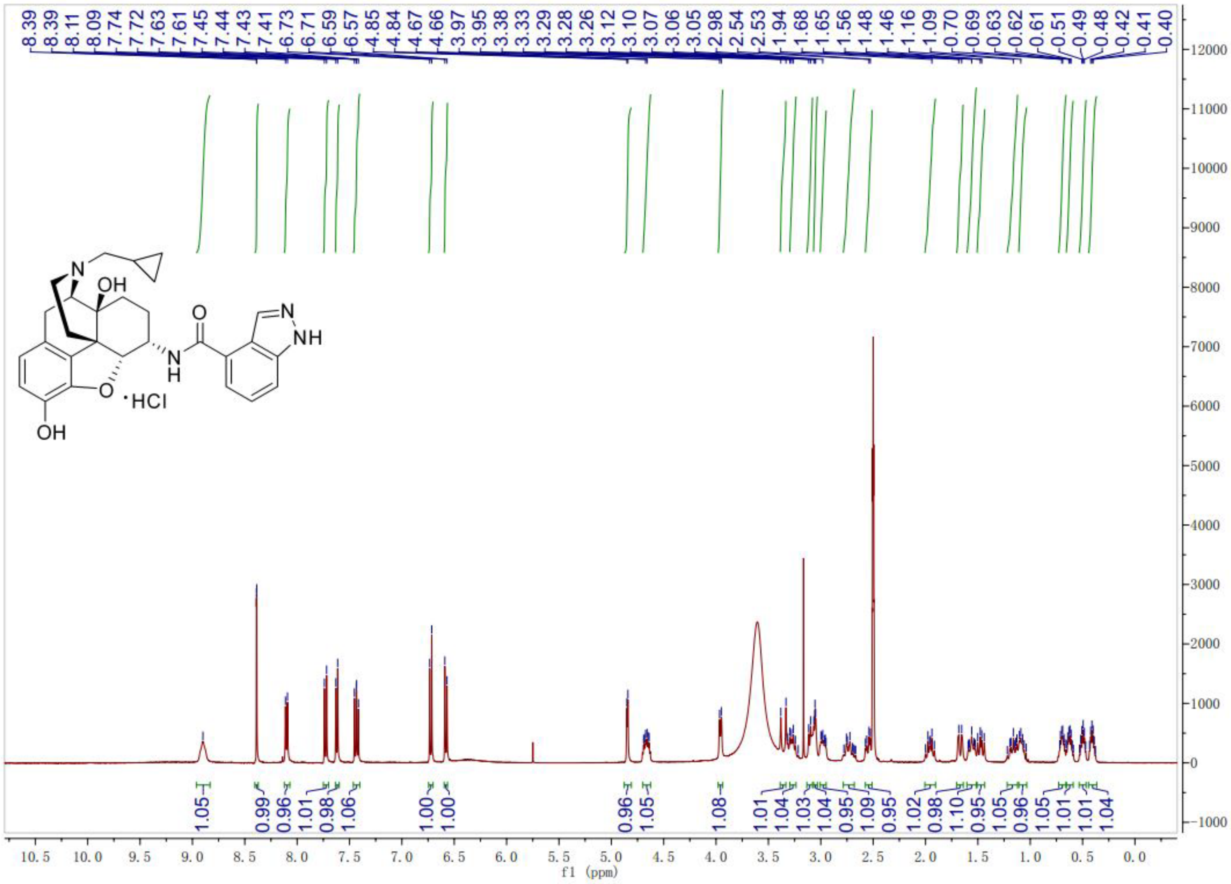

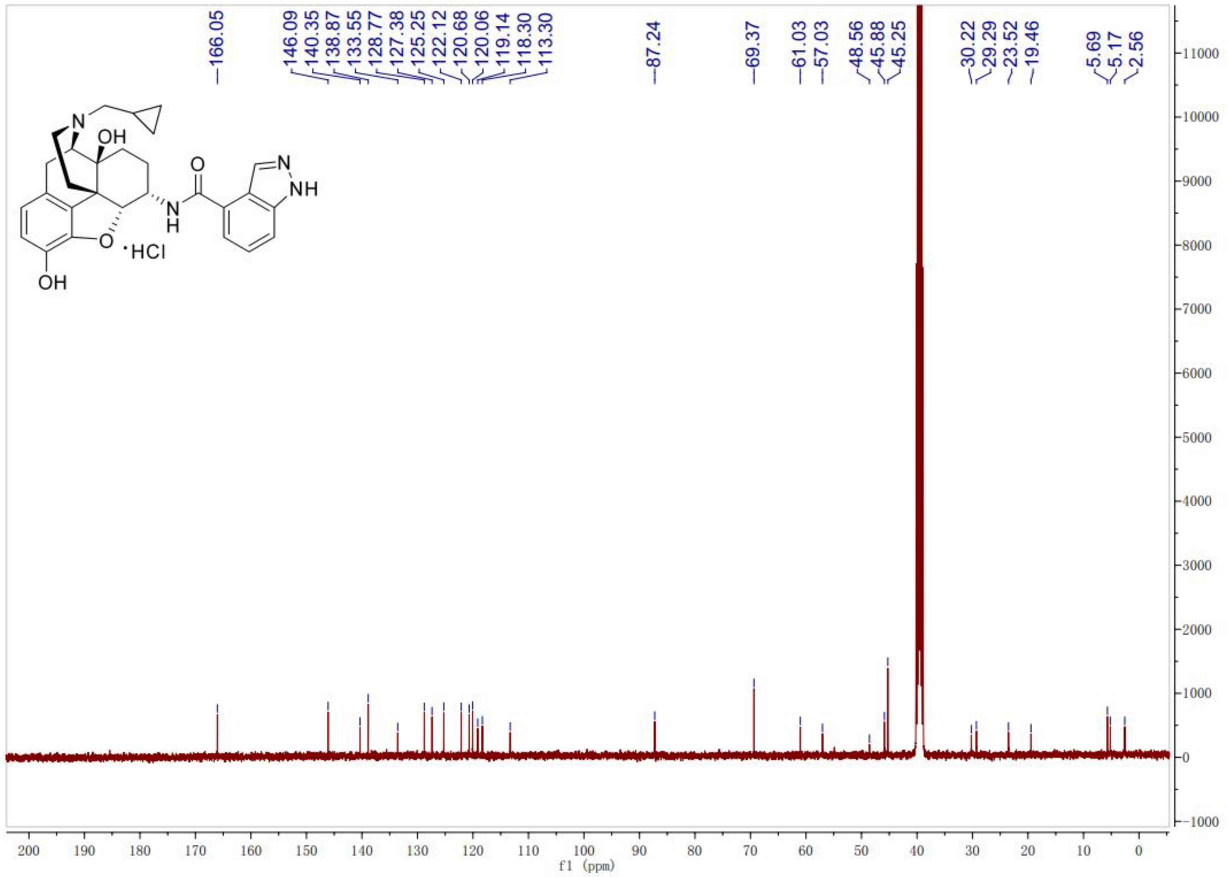
166.05
146.09
140.35
138.87
133.55
128.77
127.38
125.25
122.12
120.68
120.06
119.14
118.30
113.30
87.24
69.37
61.03
57.03
48.56
45.88
45.25
30.22
29.29
23.52
19.46
5.69
5.17
2.56
OH
N
O
NH
H
·HCl
f1 (ppm)

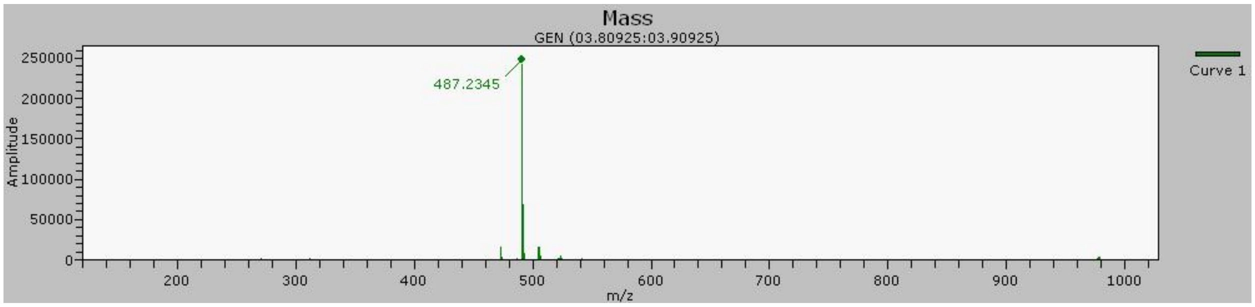
Mass
GEN (03.80925:03.90925)
487.2345
Amplitude
m/z
Curve 1

17-Cyclopropylmethyl-3,14β-dihydro-4,5α-epoxy-6β-[1H-indazole-4-carboxamide]morphinan Hydrochloride (**28**)

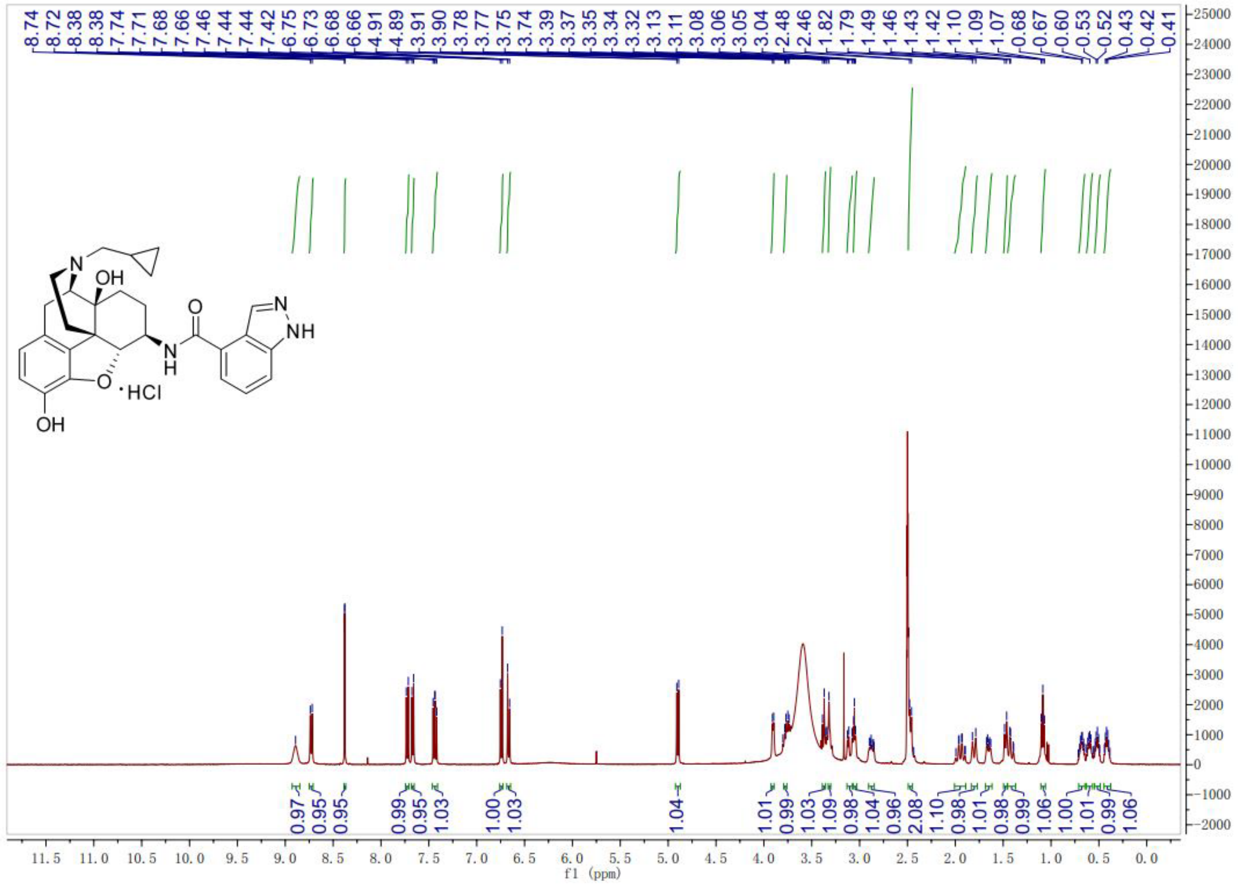

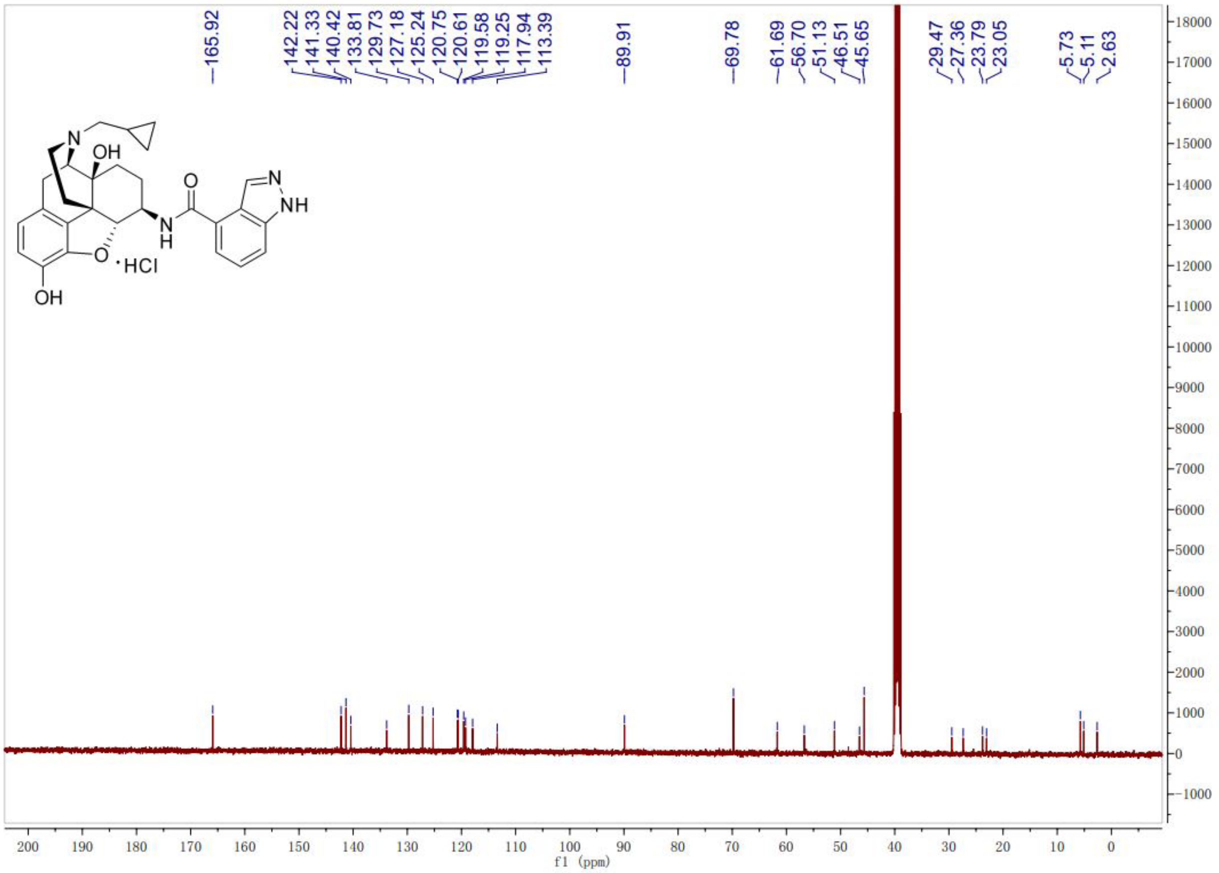

165.92
142.22
141.33
140.42
133.81
129.73
127.18
125.24
120.75
120.61
119.58
119.25
117.94
113.39
89.91
69.78
61.69
56.70
51.13
46.51
45.65
29.47
27.36
23.79
23.05
5.73
5.11
2.63
OH
N
O
NH
H
·HCl
f1 (ppm)

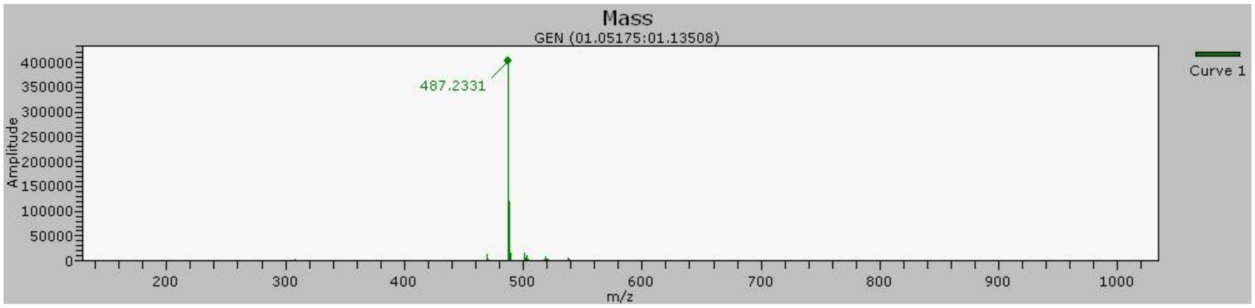

Mass
GEN (01.05175:01.13508)
487.2331
Curve 1
Amplitude
m/z

17-Cyclopropylmethyl-3,14β-dihydro-4,5α-epoxy-6α-[1H-benzo[d]imidazole-4-carboxamide]morphinan Hydrochloride (**29**)

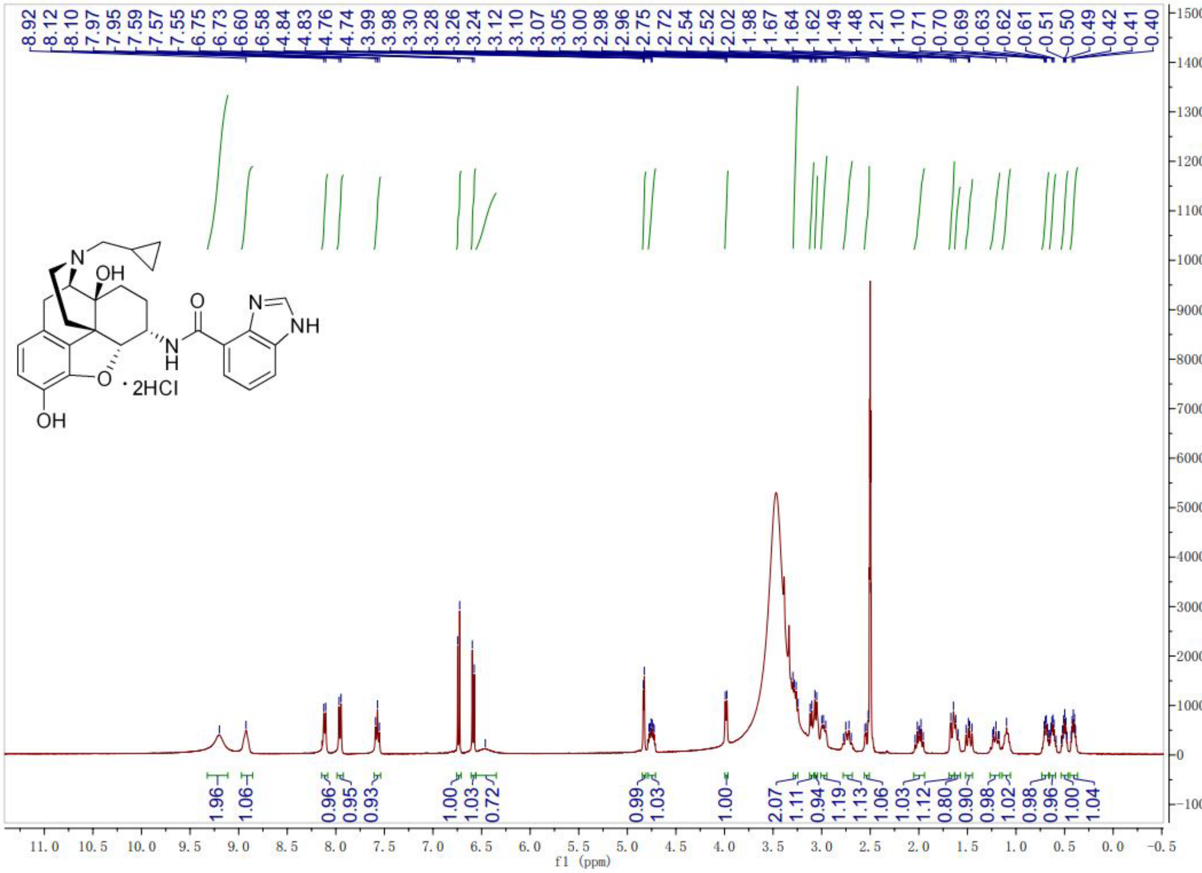

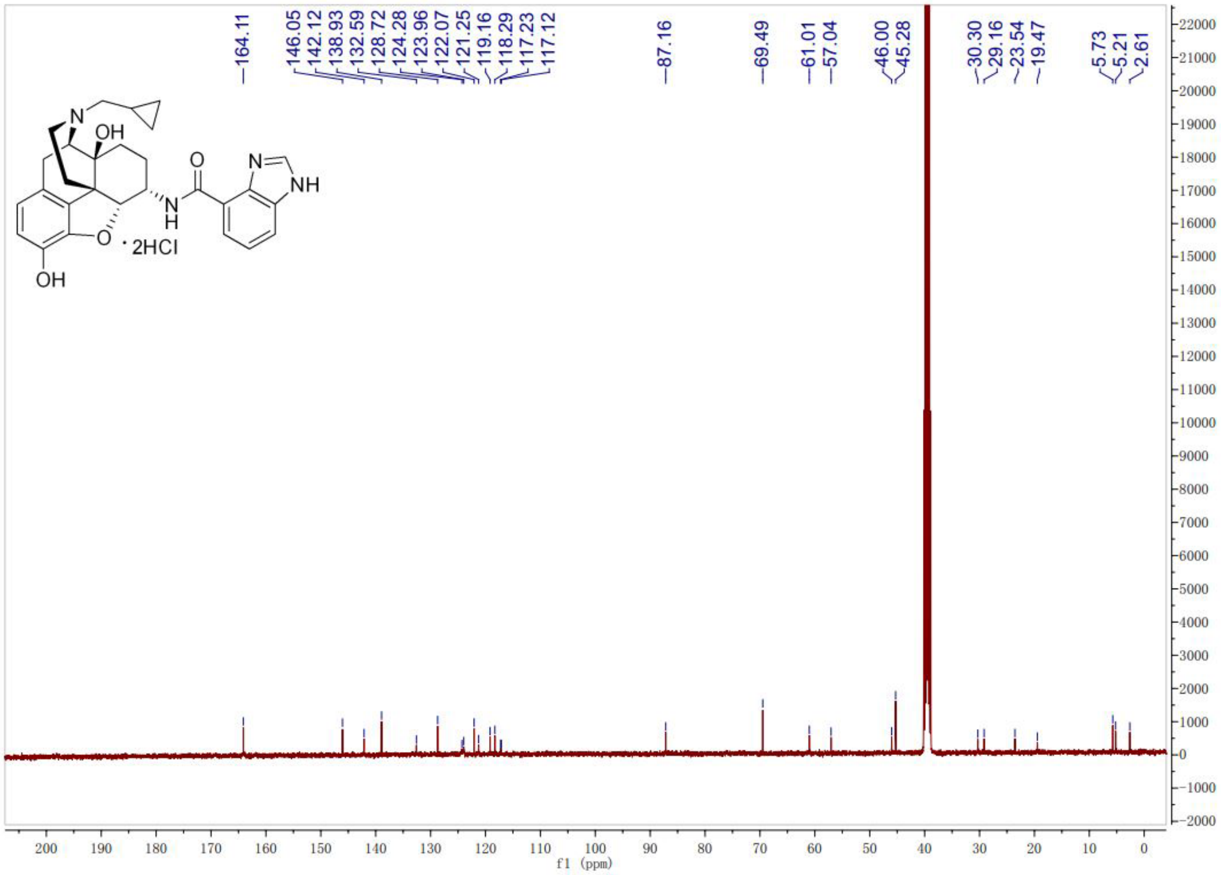

164.11
146.05
142.12
138.93
132.59
128.72
124.28
123.96
122.07
121.25
119.16
118.29
117.23
117.12
87.16
69.49
61.01
57.04
46.00
45.28
30.30
29.16
23.54
19.47
5.73
5.21
2.61
N
OH
O
N
NH
N
H
O
· 2HCl
OH
f1 (ppm)

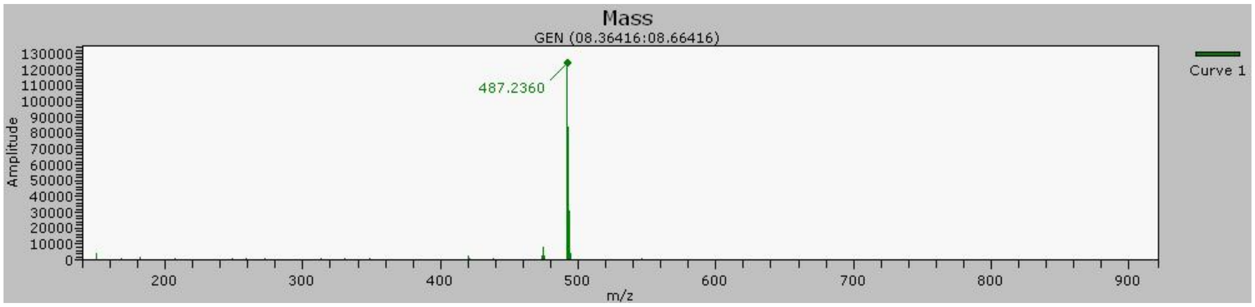

Mass
GEN (08.36416:08.66416)
487.2360
Curve 1
Amplitude
m/z

17-Cyclopropylmethyl-3,14β-dihydro-4,5α-epoxy-6β-[1H-benzo[d]imidazole-4-carboxamide]morphinan Hydrochloride (**30**)

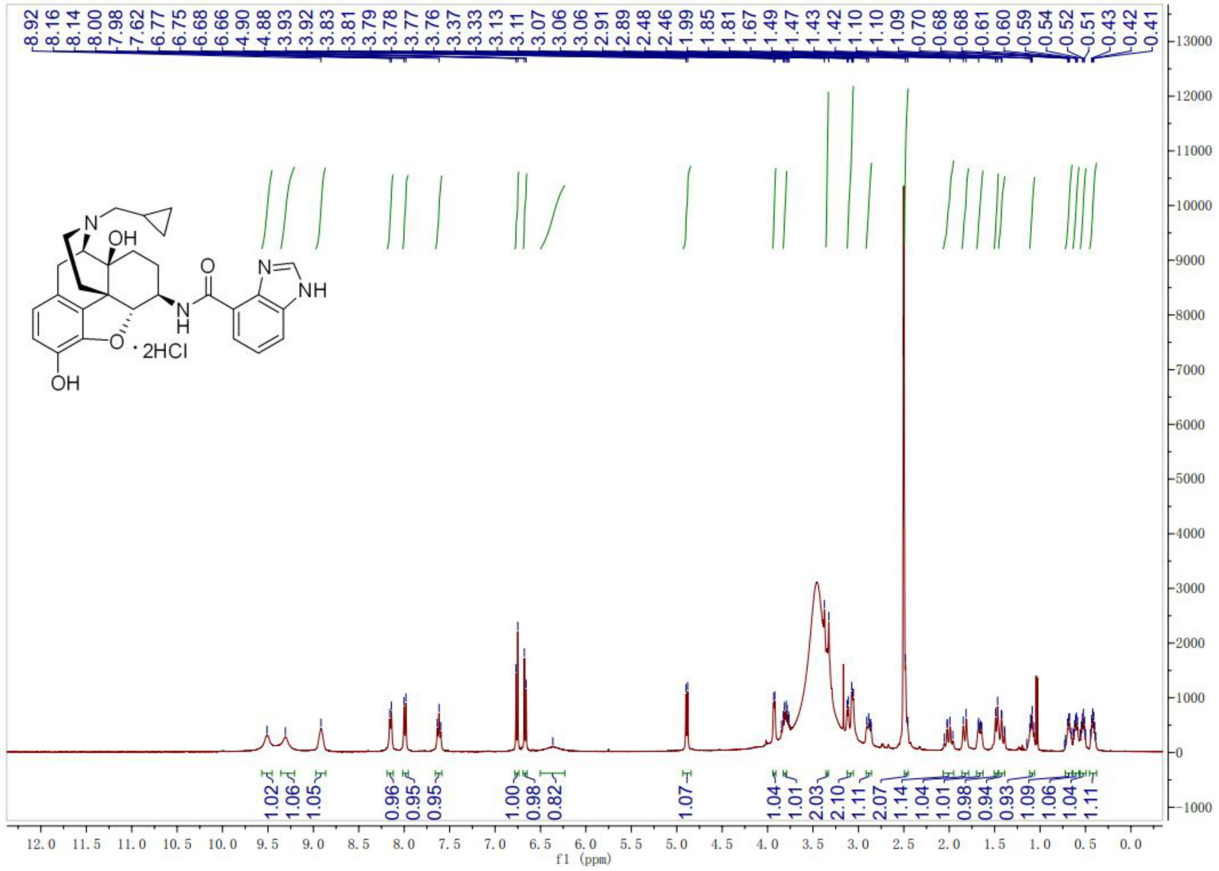

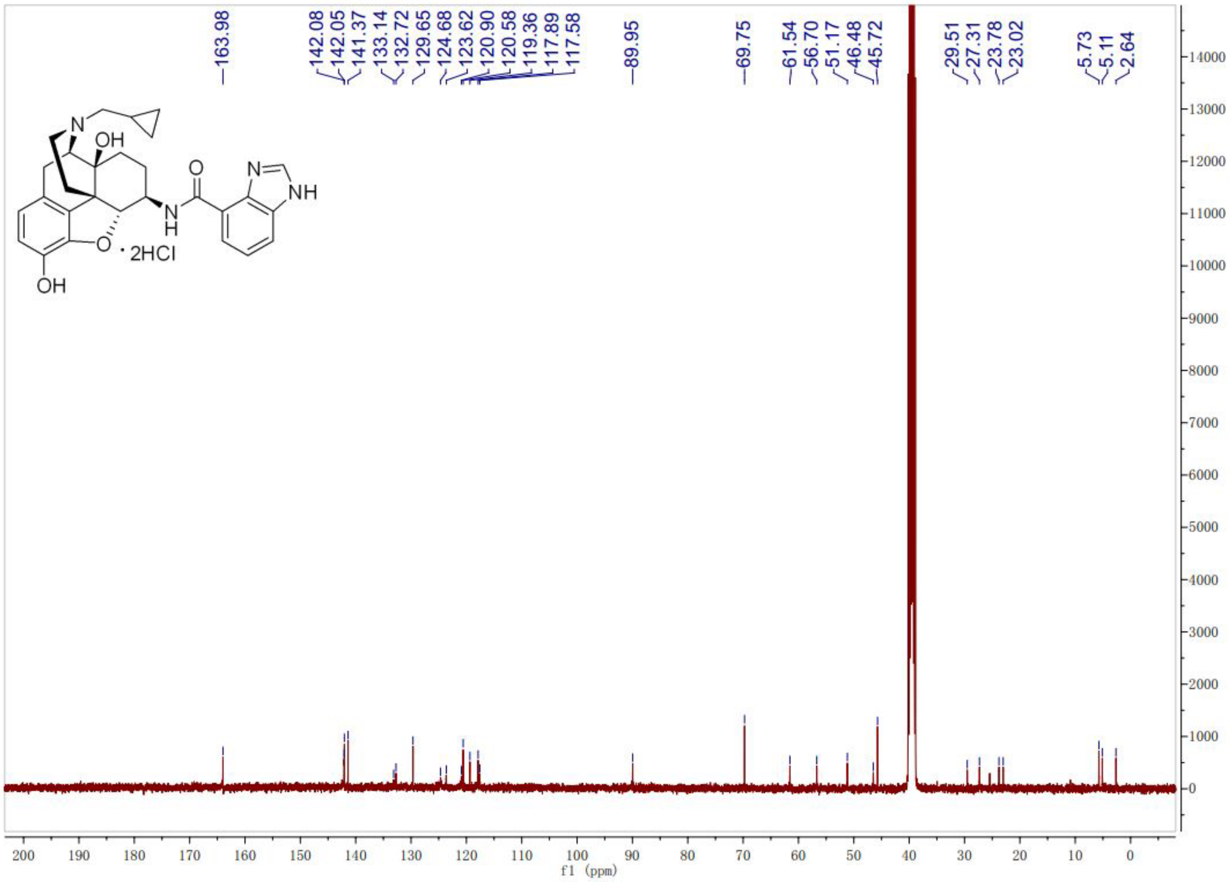

163.98
142.08
142.05
141.37
133.14
132.72
129.65
124.68
123.62
120.90
120.58
119.36
117.89
117.58
89.95
69.75
61.54
56.70
51.17
46.48
45.72
29.51
27.31
23.78
23.02
5.73
5.11
2.64
N
OH
O
N
NH
H
O
· 2HCl
OH
f1 (ppm)

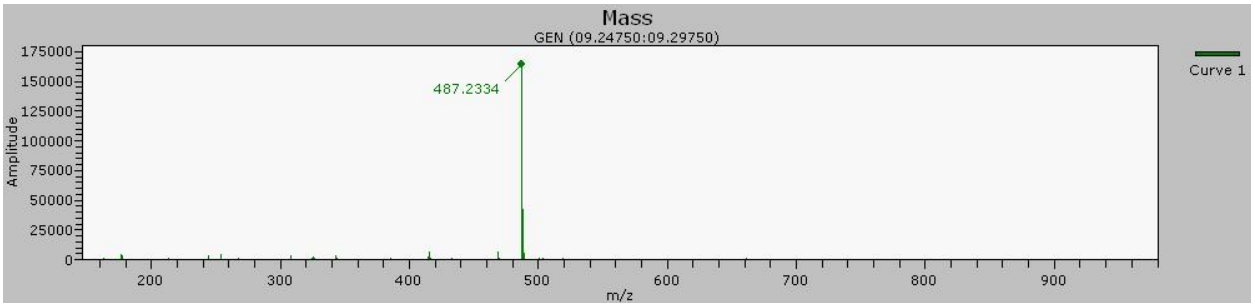

Mass
GEN (09.24750:09.29750)
487.2334
Amplitude
m/z
Curve 1

17-Cyclopropylmethyl-3,14β-dihydro-4,5α-epoxy-6α-[1H-pyrrolo[3,2-c]pyridine-4-carboxamide]morphinan Hydrochloride (**31**)

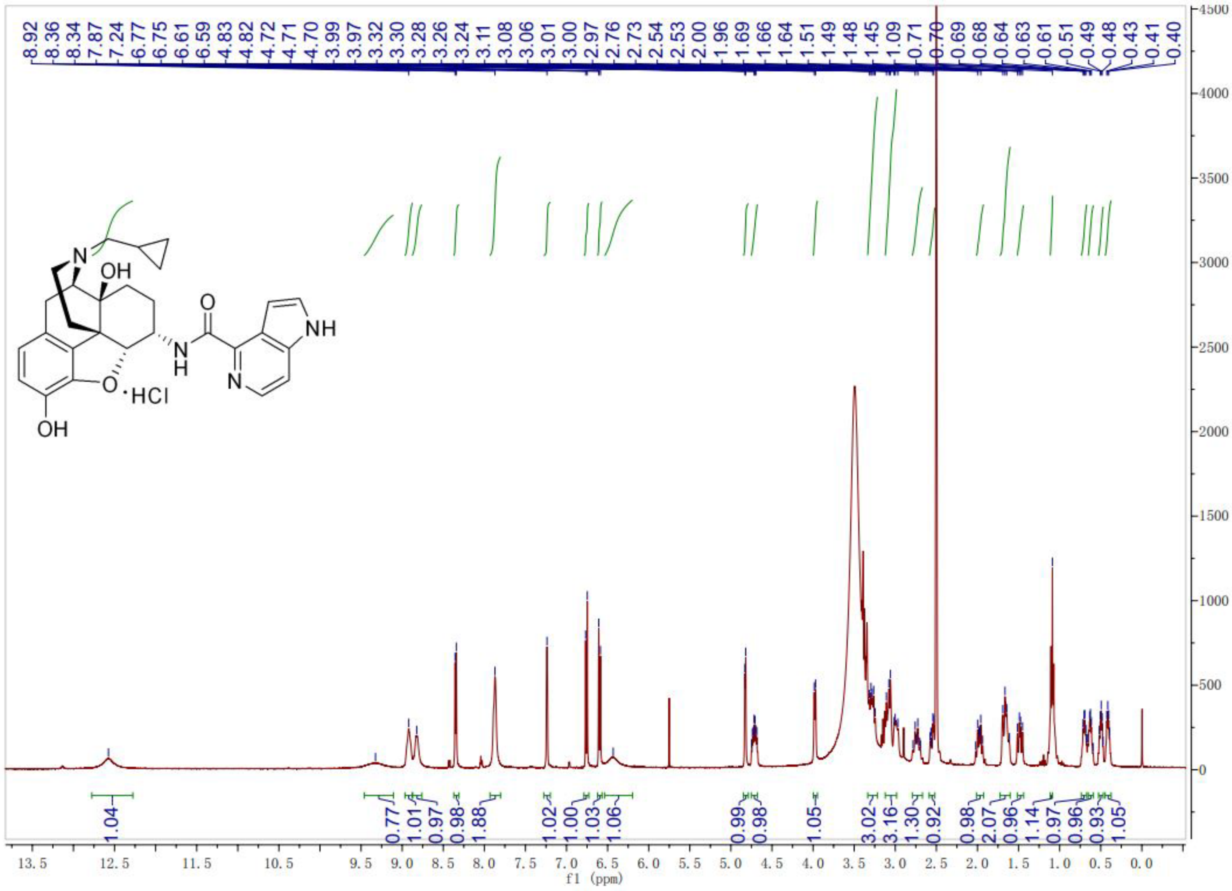

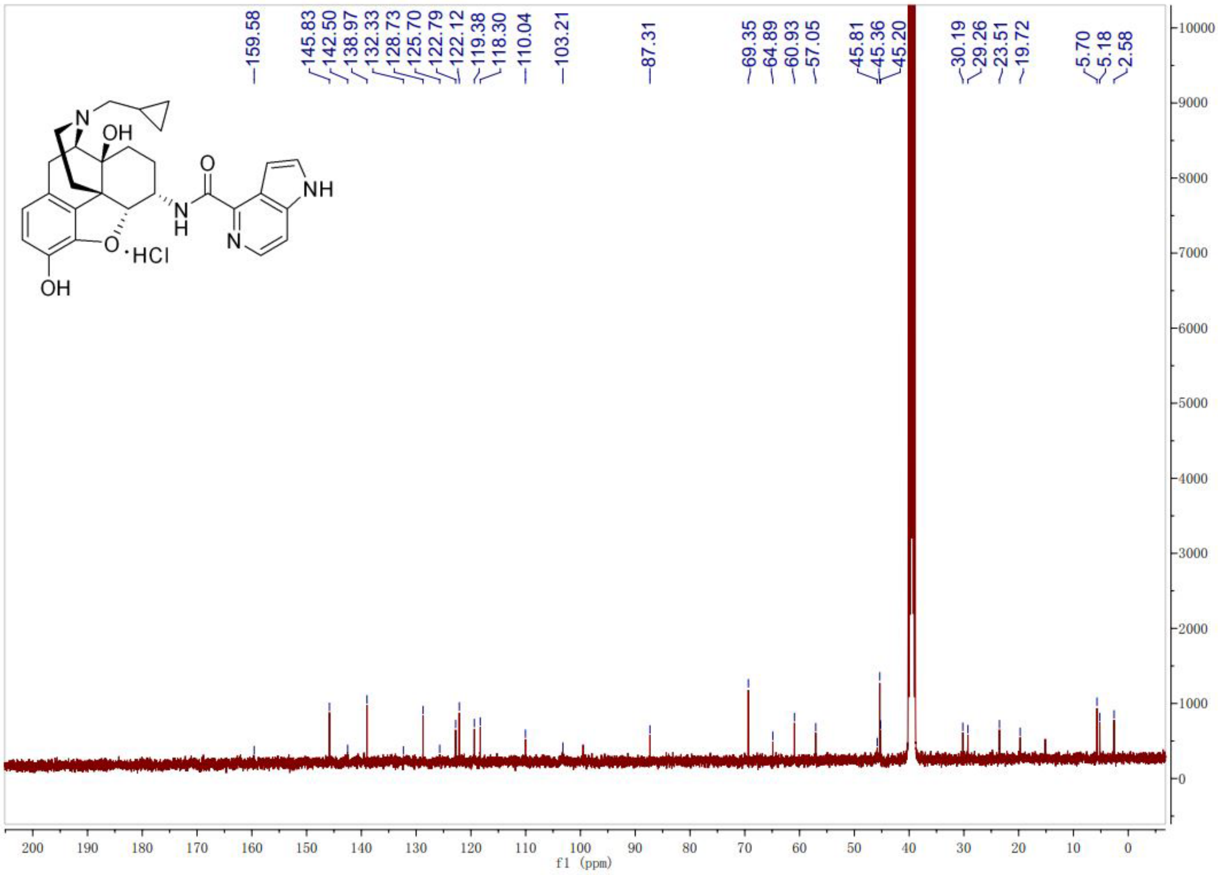
159.58
145.83
142.50
138.97
132.33
128.73
125.70
122.79
122.12
119.38
118.30
110.04
103.21
87.31
69.35
64.89
60.93
57.05
45.81
45.36
45.20
30.19
29.26
23.51
19.72
5.70
5.18
2.58
OH
O
NH
N
H
N
O
·HCl
OH
f1 (ppm)

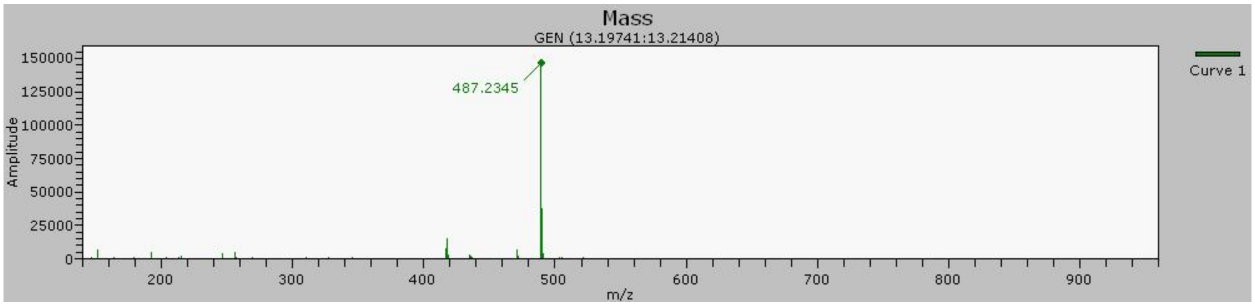
Mass
GEN (13.19741:13.21408)
487.2345
Amplitude
m/z
Curve 1

17-Cyclopropylmethyl-3,14β-dihydro-4,5α-epoxy-6β-[1H-pyrrolo[3,2-c]pyridine-4-carboxamide]morphinan Hydrochloride (**32**)

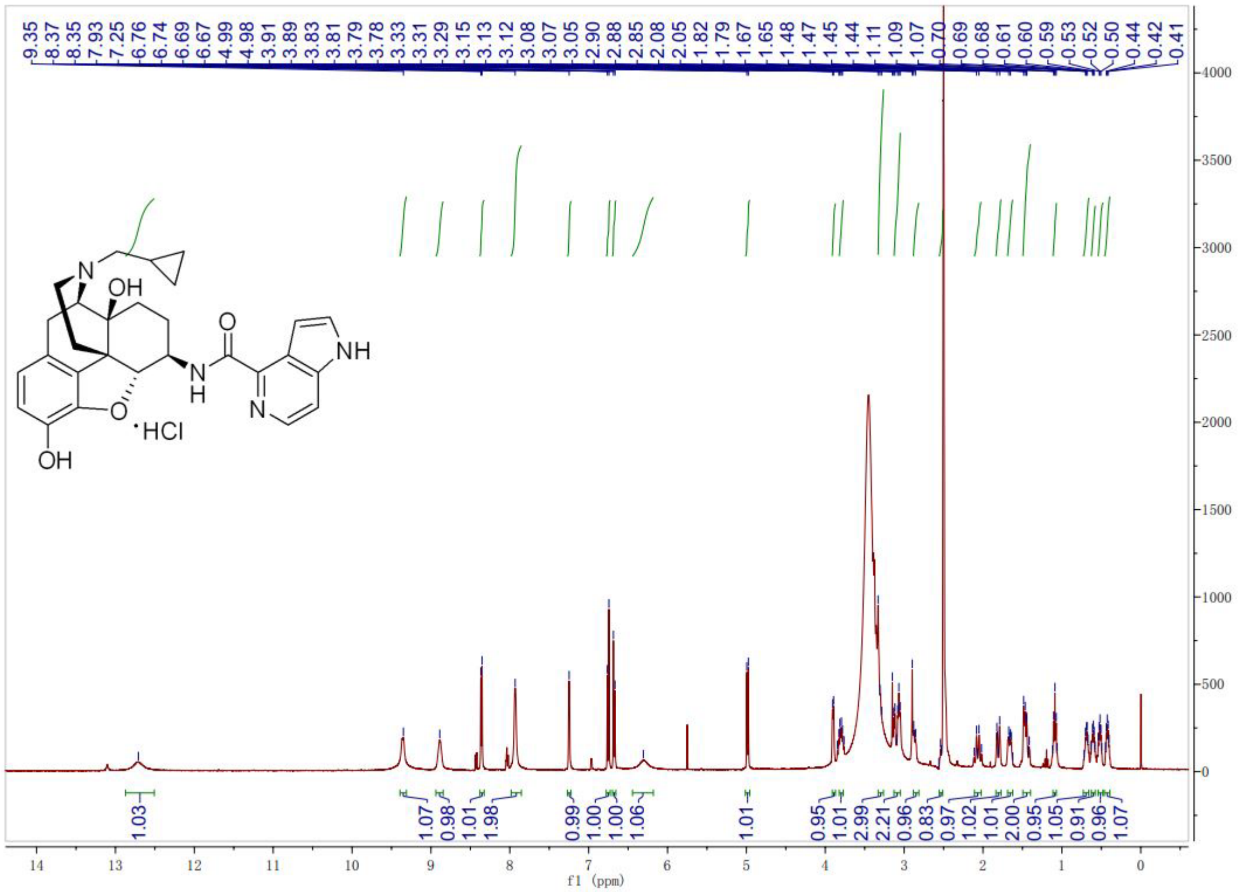

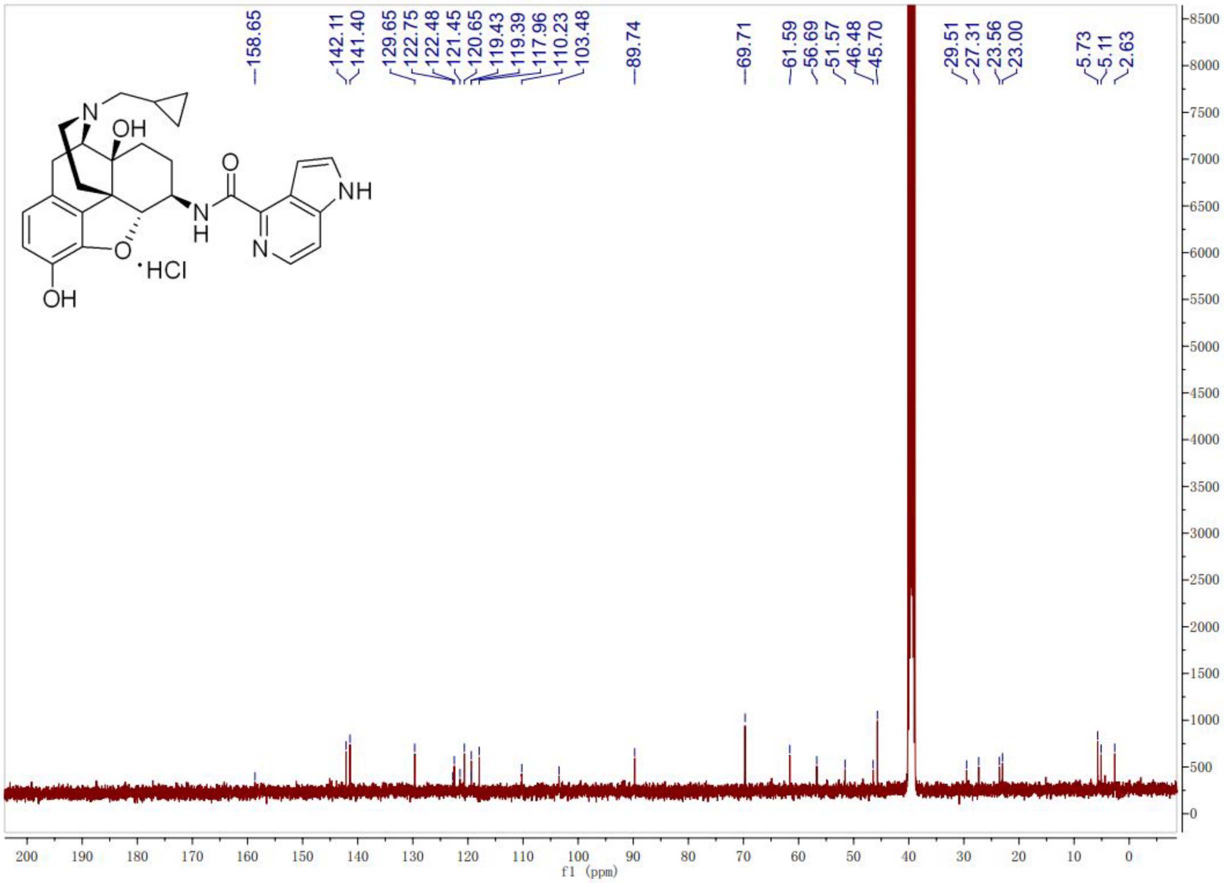

158.65
142.11
141.40
129.65
122.75
122.48
121.45
120.65
119.43
119.39
117.96
110.23
103.48
89.74
69.71
61.59
56.69
51.57
46.48
45.70
29.51
27.31
23.56
23.00
5.73
5.11
2.63
OH
N
NH
O
H
·HCl
f1 (ppm)

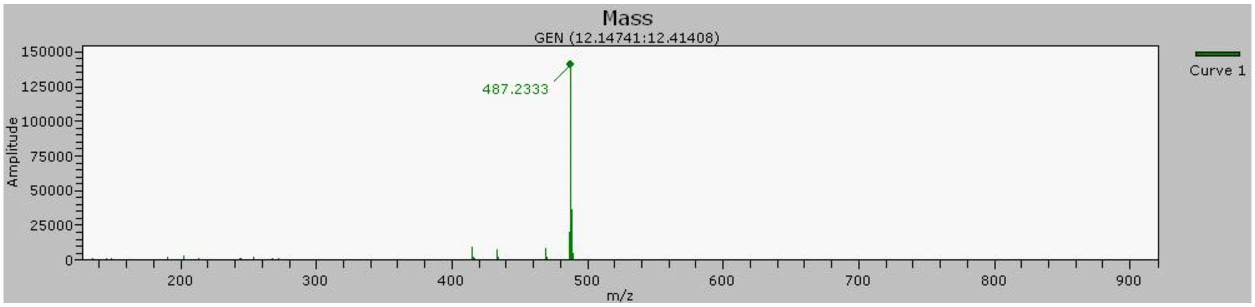

Mass
GEN (12.14741:12.41408)
Amplitude
487.2333
Curve 1
m/z

17-Cyclopropylmethyl-3,14β-dihydro-4,5α-epoxy-6α-[1H-pyrrolo[2,3-c]pyridine-4-carboxamide]morphinan Hydrochloride (**33**)

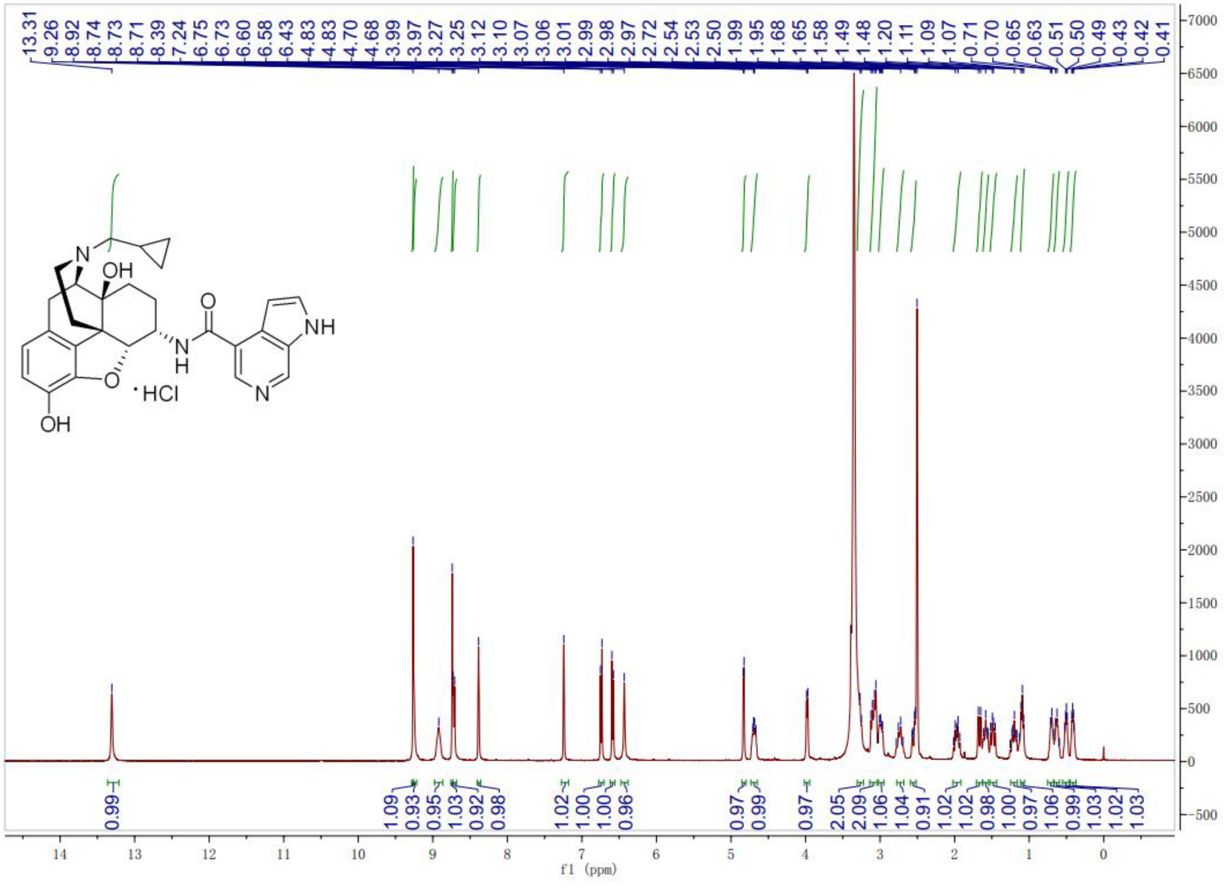

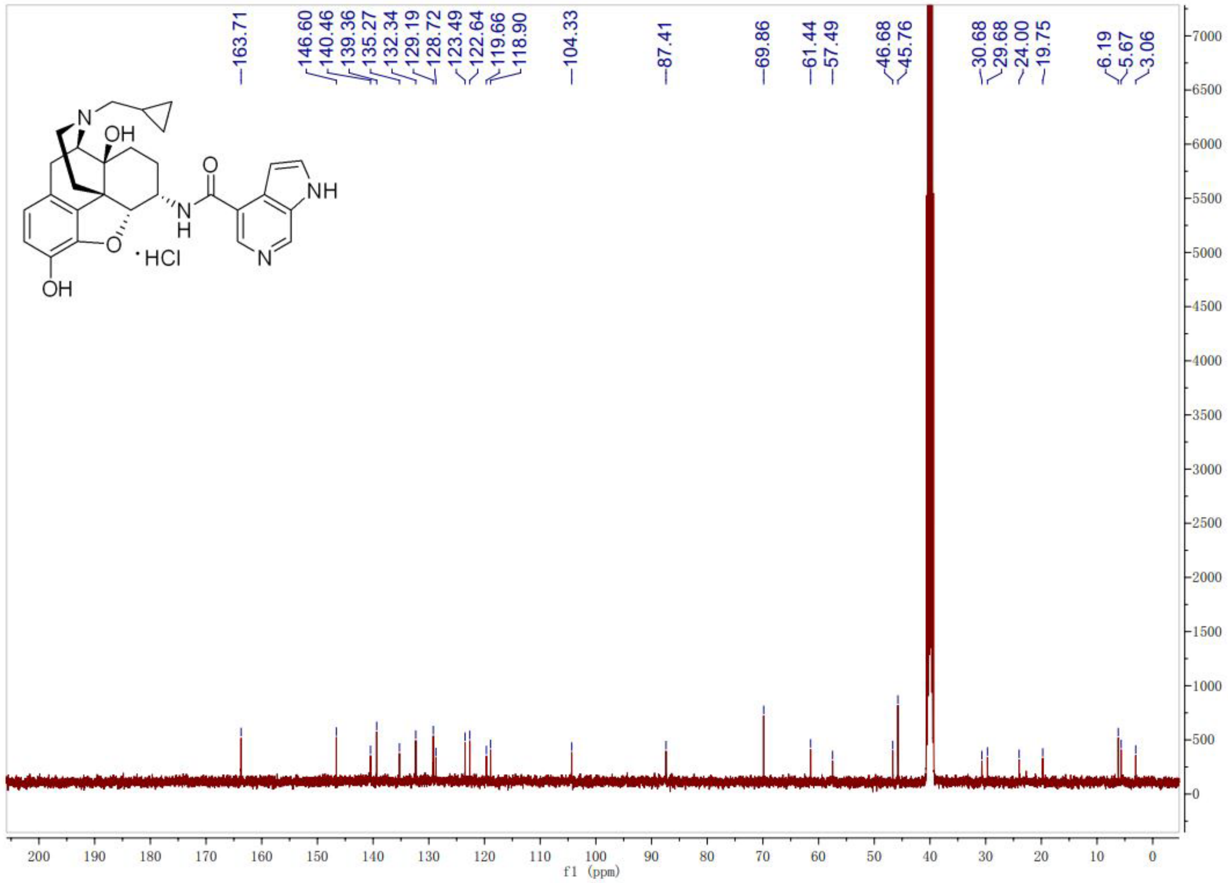
163.71
146.60
140.46
139.36
135.27
132.34
129.19
128.72
123.49
122.64
119.66
118.90
104.33
87.41
69.86
61.44
57.49
46.68
45.76
30.68
29.68
24.00
19.75
6.19
5.67
3.06
OH
O
NH
N
H
O
·HCl
N
OH
f1 (ppm)

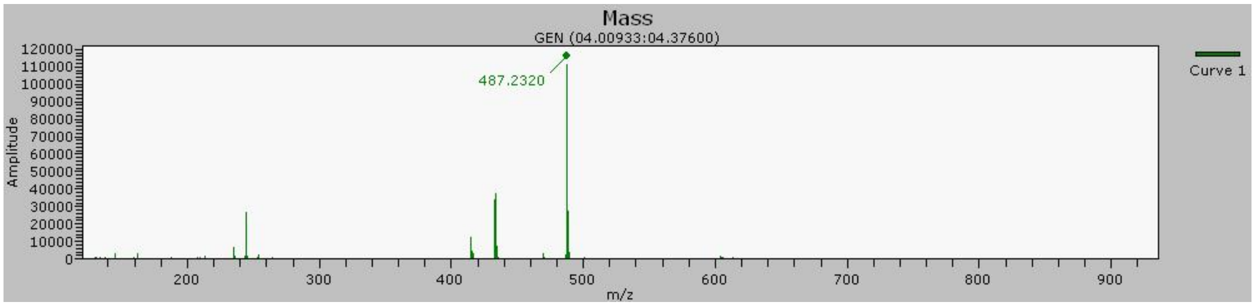
Mass
GEN (04.00933:04.37600)
487.2320
Amplitude
m/z
Curve 1

17-Cyclopropylmethyl-3,14β-dihydro-4,5α-epoxy-6β-[1H-pyrrolo[2,3-c]pyridine-4-carboxamide]morphinan Hydrochloride (**34**)

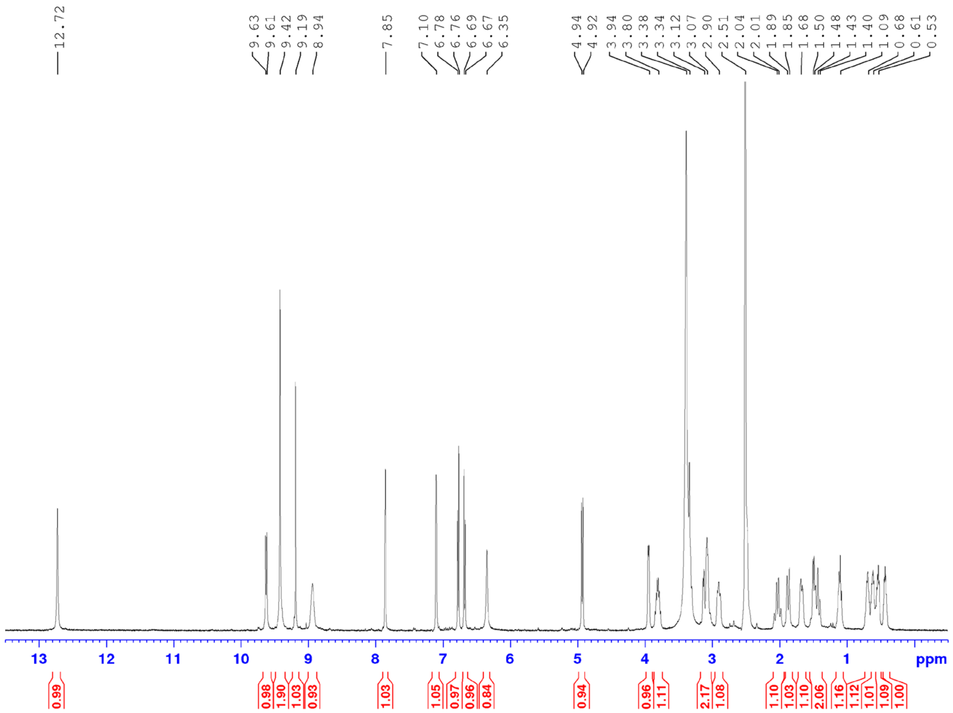

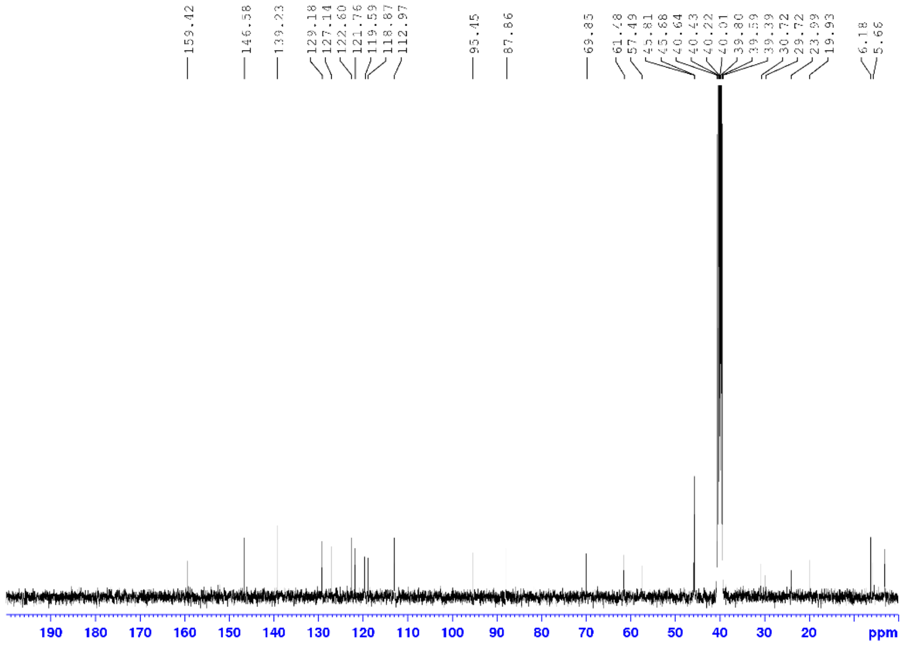

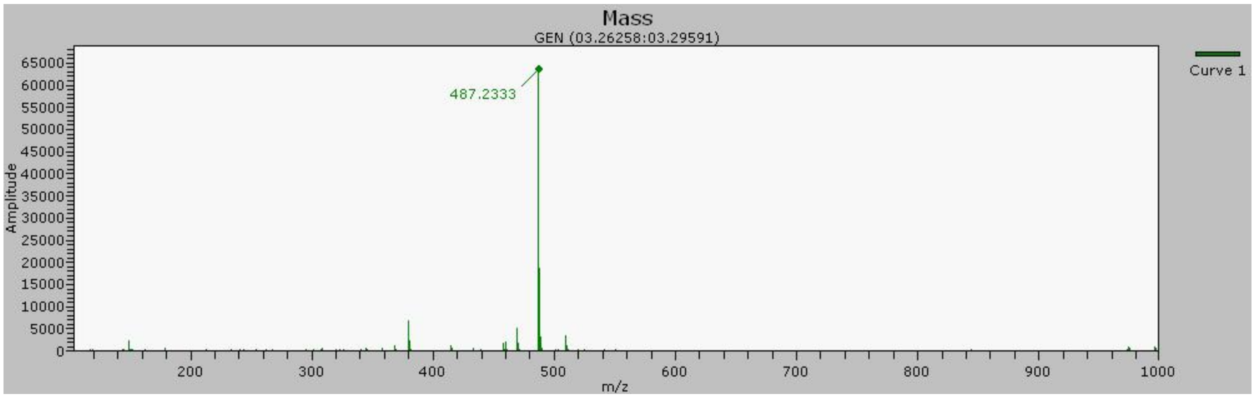

17-Cyclopropylmethyl-3,14β-dihydro-4,5α-epoxy-6α-[1H-pyrrolo[2,3-b]pyridine-4-carboxamide]morphinan Hydrochloride (**35**)

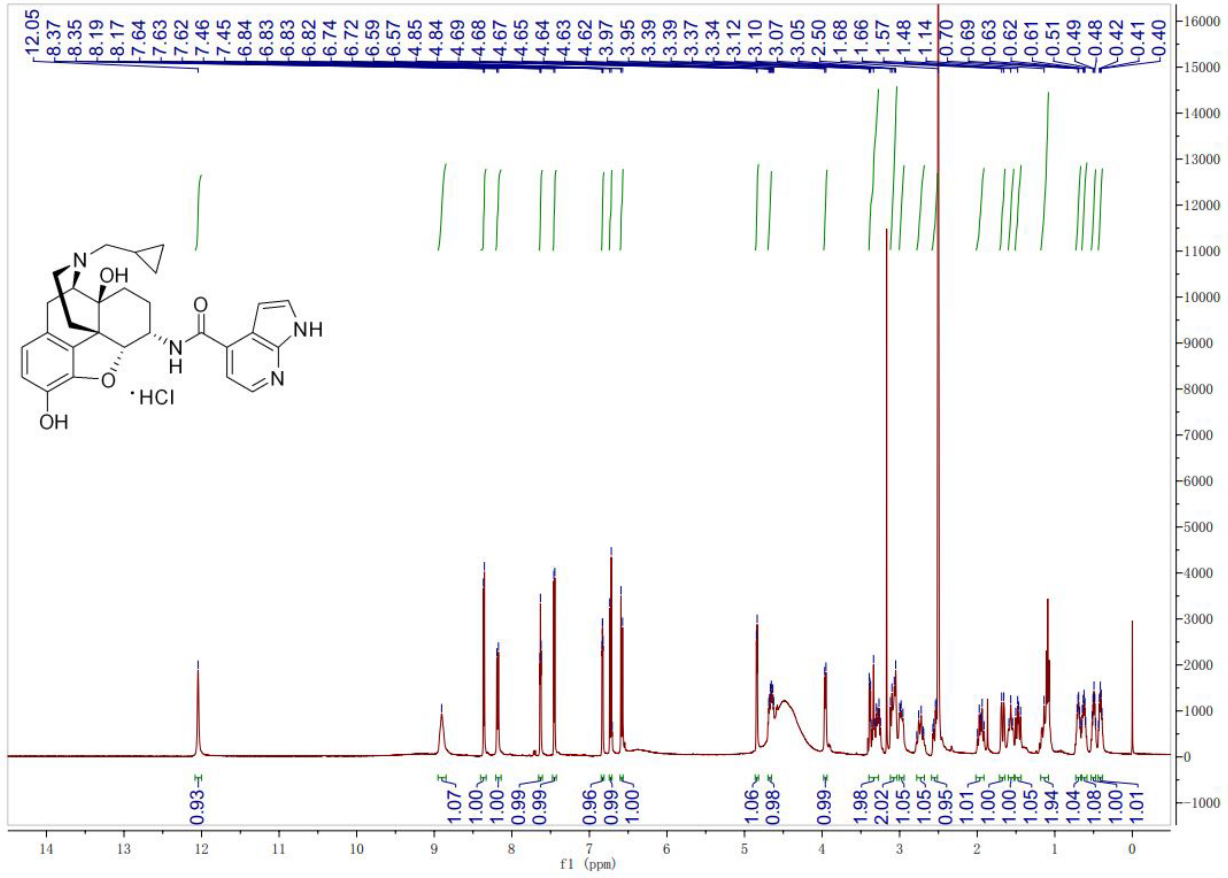

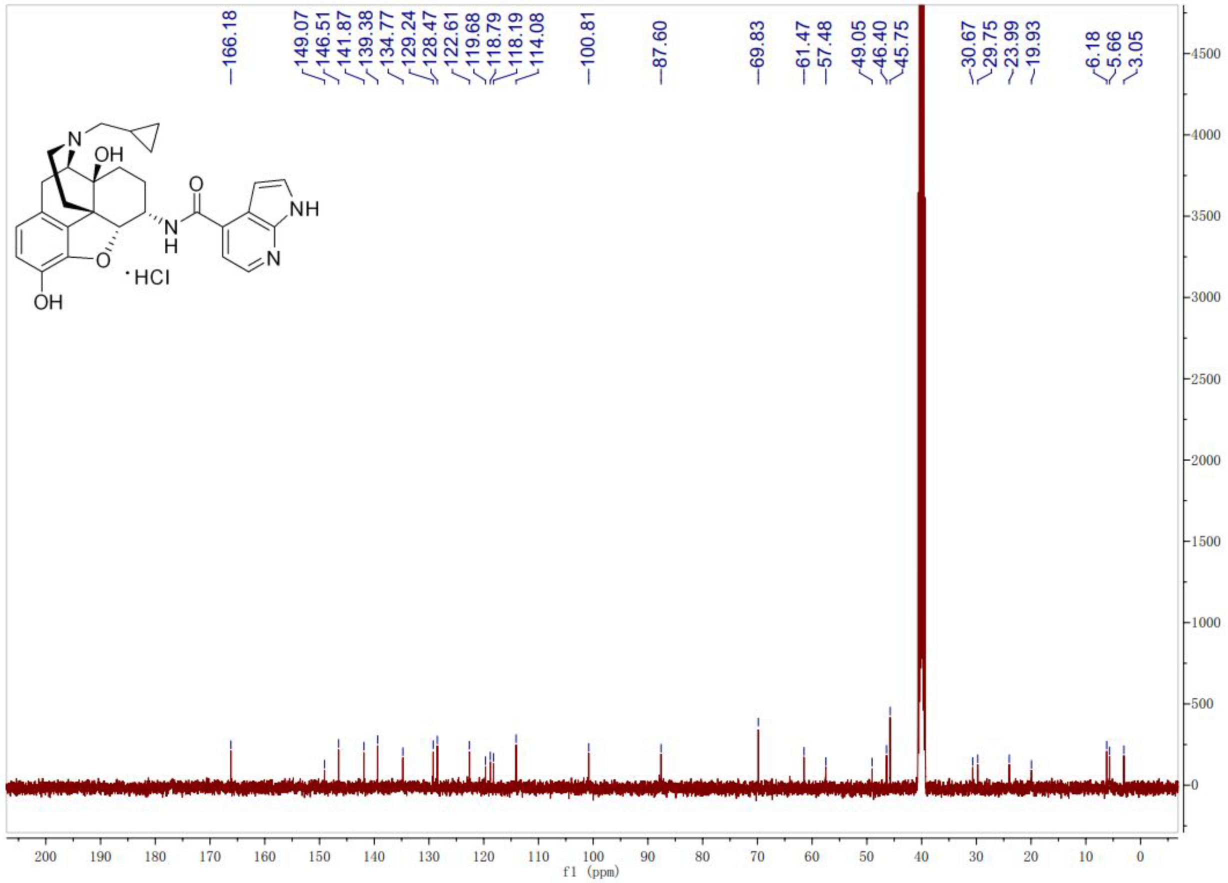

166.18
149.07
146.51
141.87
139.38
134.77
129.24
128.47
122.61
119.68
118.79
118.19
114.08
100.81
87.60
69.83
61.47
57.48
49.05
46.40
45.75
30.67
29.75
23.99
19.93
6.18
5.66
3.05
N
OH
O
N
H
NH
N
O
·HCl
OH
f1 (ppm)

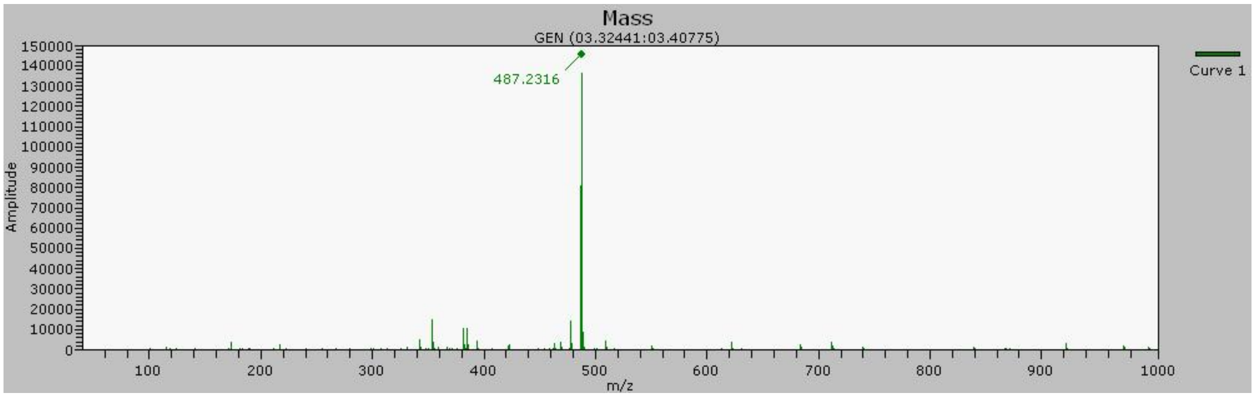

Mass
GEN (03.32441:03.40775)
487.2316
Curve 1
Amplitude
m/z

17-Cyclopropylmethyl-3,14β-dihydro-4,5α-epoxy-6β-[1H-pyrrolo[2,3-b]pyridine-4-carboxamide]morphinan Hydrochloride (**36**)

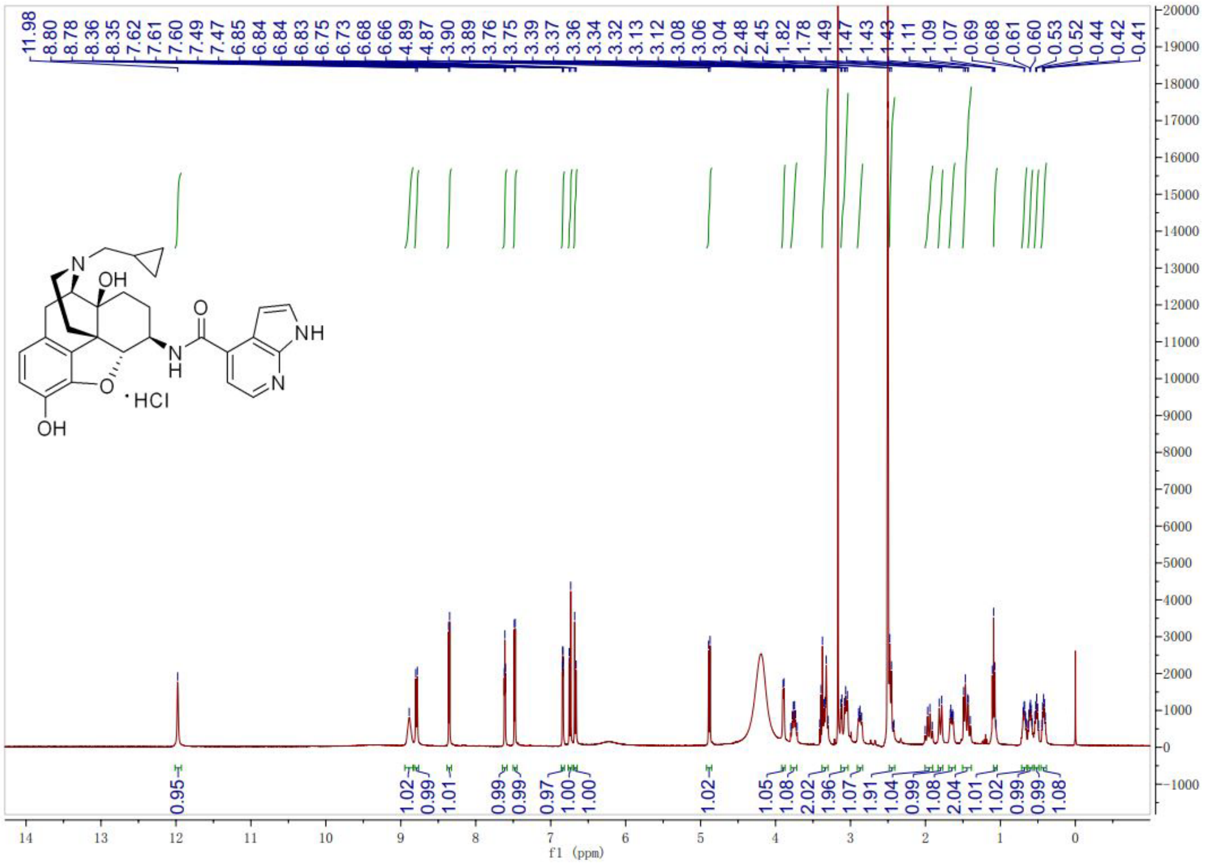

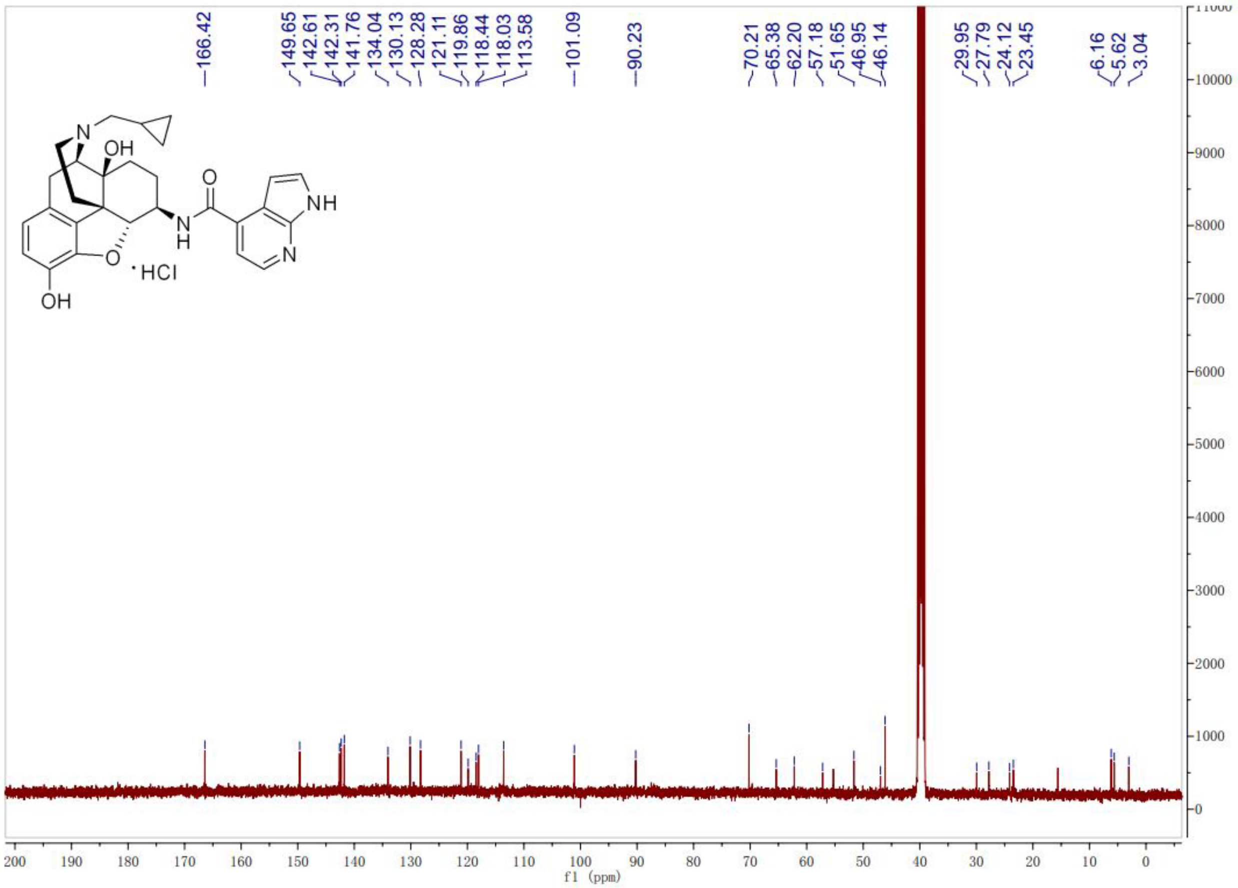

166.42
149.65
142.61
142.31
141.76
134.04
130.13
128.28
121.11
119.86
118.44
118.03
113.58
101.09
90.23
70.21
65.38
62.20
57.18
51.65
46.95
46.14
29.95
27.79
24.12
23.45
6.16
5.62
3.04
OH
N
O
NH
H
N
·HCl
OH
f1 (ppm)

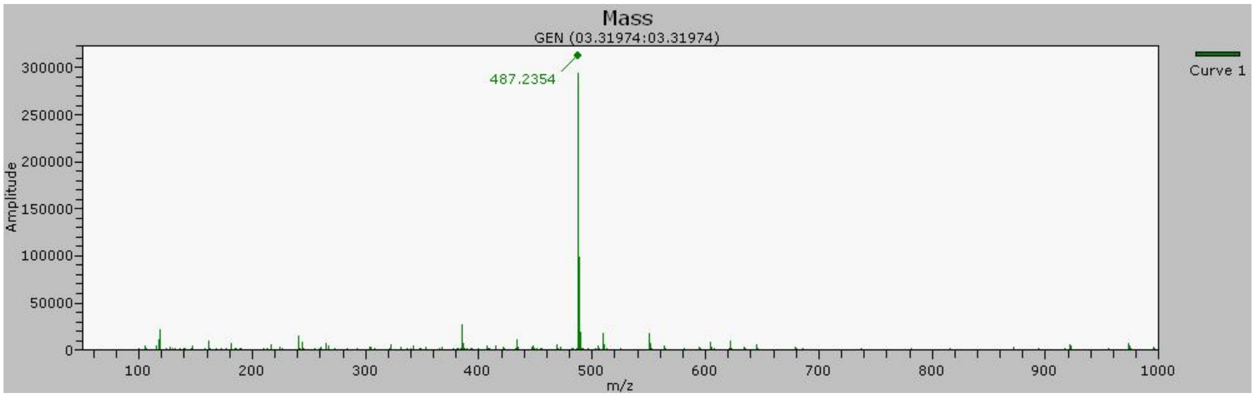

Mass
GEN (03.31974:03.31974)
487.2354
Amplitude
m/z
Curve 1

2.) Purity data of final compounds

HPLC System: Waters Arc HPLC
Column: XBridge[TM] C_{18} 3.5 µm (4.6 x 50 mm)
Sample Concentration: 1 mg/mL
Injection Solvent: 25%MeOH in H_2O
Injection Volume: 5 µL
Gradient Mobile Phase:
Start:
90% Mobile Phase A – 0.1% Trifluoroacetic acid in water
10% Mobile Phase B – Acetonitrile
End:
10% Mobile Phase A – 0.1% Trifluoroacetic acid in water
90% Mobile Phase B – Acetonitrile
Flow Rate: 0.8 mL/min
Single Wavelength: 254 nm
Run time: 10 min or 15 min

Table S1. HPLC Analysis of Target Compounds.

Compound	Retention Time (min)	Purity (%)
**1**	5.166	97.06
**2**	4.925	95.83
**3**	4.087	99.00
**4**	5.677	98.62
**5**	4.053	98.67
**6**	4.132	97.32
**7**	4.722	95.98
**8**	5.185	98.67
**9**	5.563	96.92
**10**	4.720	98.88
**11**	4.050	98.79
**12**	5.113	97.49
**13**	4.457	95.56
**14**	4.102	95.76
**15**	5.405	96.32
**16**	4.670	96.74
**17**	5.450	99.50
**18**	5.867	99.16
**19**	4.057	95.65
**20**	6.003	98.22
**21**	4.875	96.63
**22**	4.178	95.93
**23**	6.017	97.13
**24**	4.422	99.59
**25**	5.273	98.99
**26**	4.230	99.20
**27**	5.730	99.30

**28**	4.602	95.42
**29**	4.198	98.41
**30**	5.580	97.95
**31**	5.630	97.12
**32**	5.865	96.98
**33**	5.527	96.59
**34**	5.530	95.20
**35**	6.103	99.28
**36**	5.775	95.76

3.) HPLC chromatograms of final compounds

Compound 1

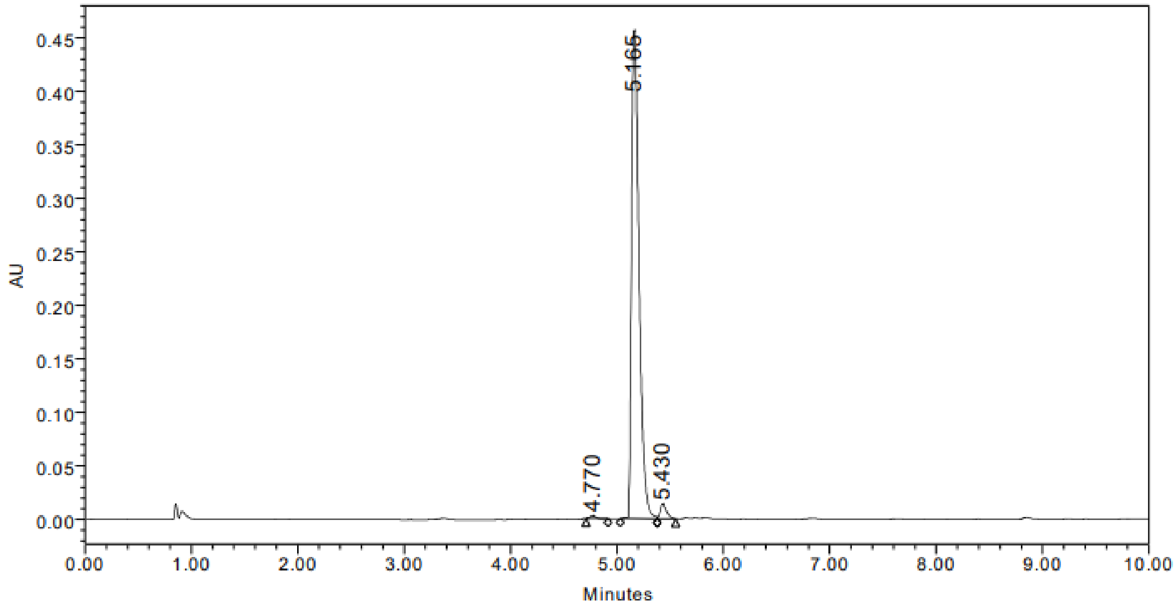

Compound 2

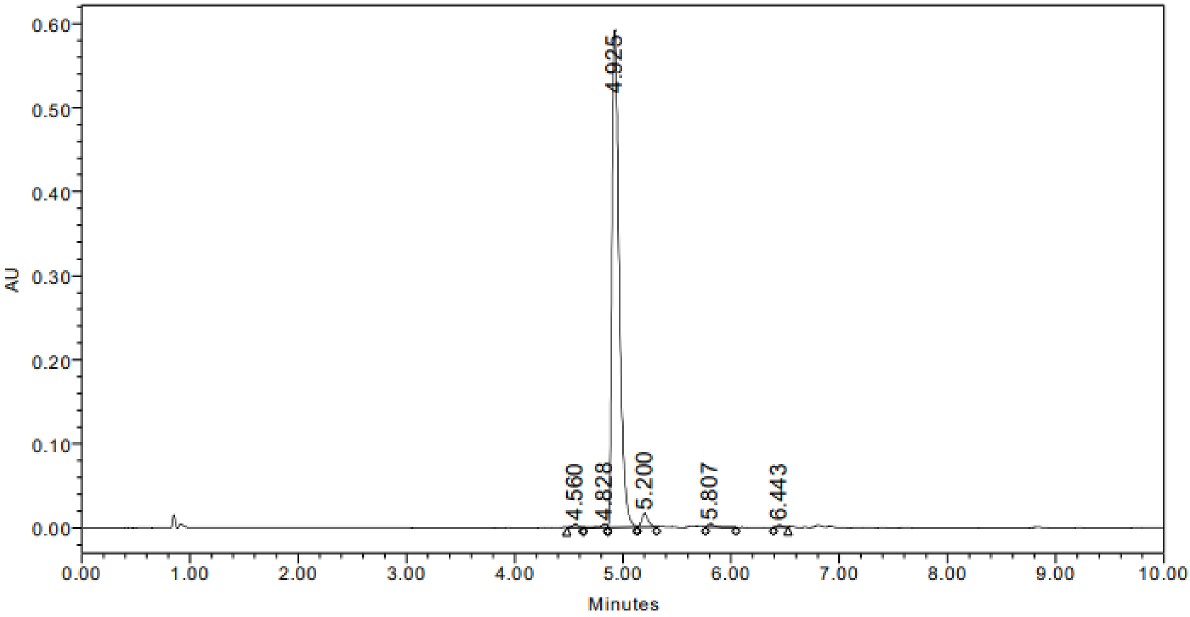

Compound 3

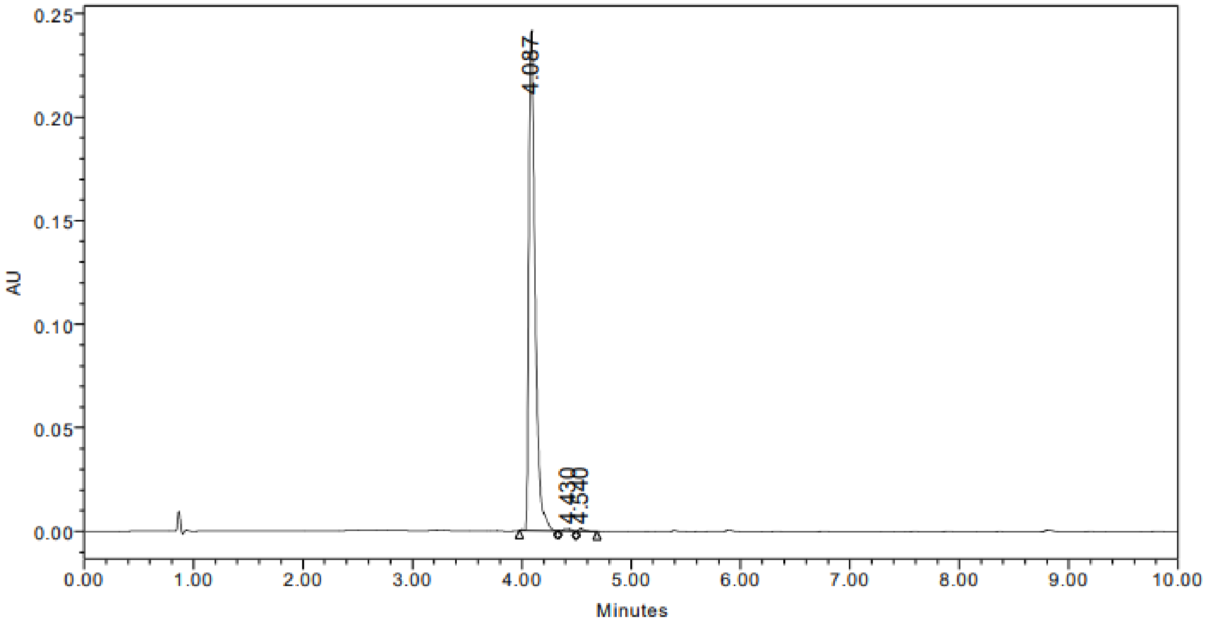

Compound 4

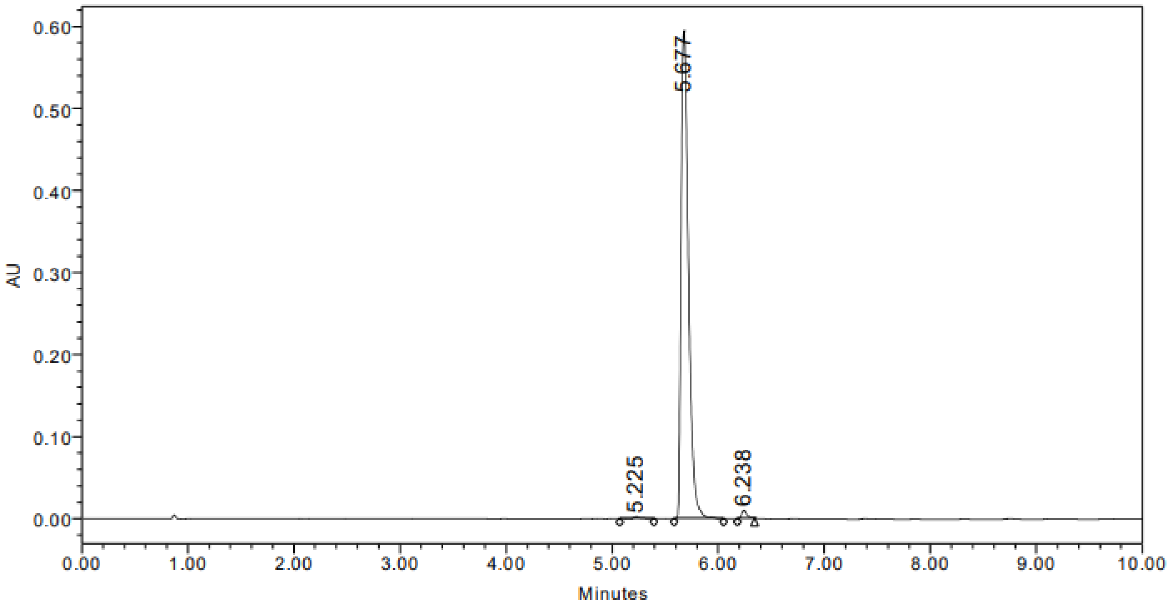

Compound 5

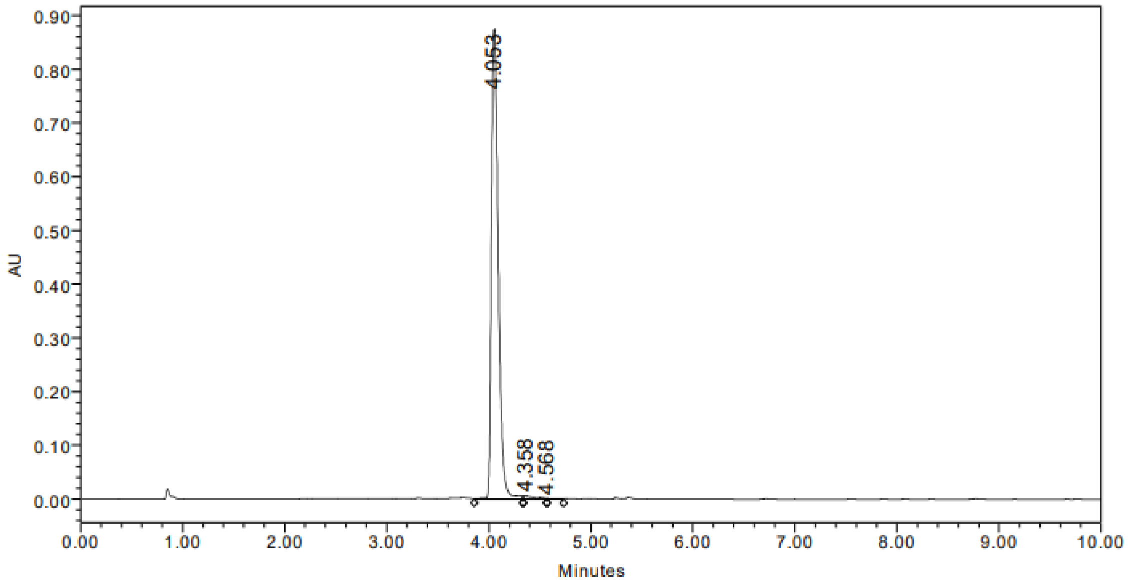

Compound 6

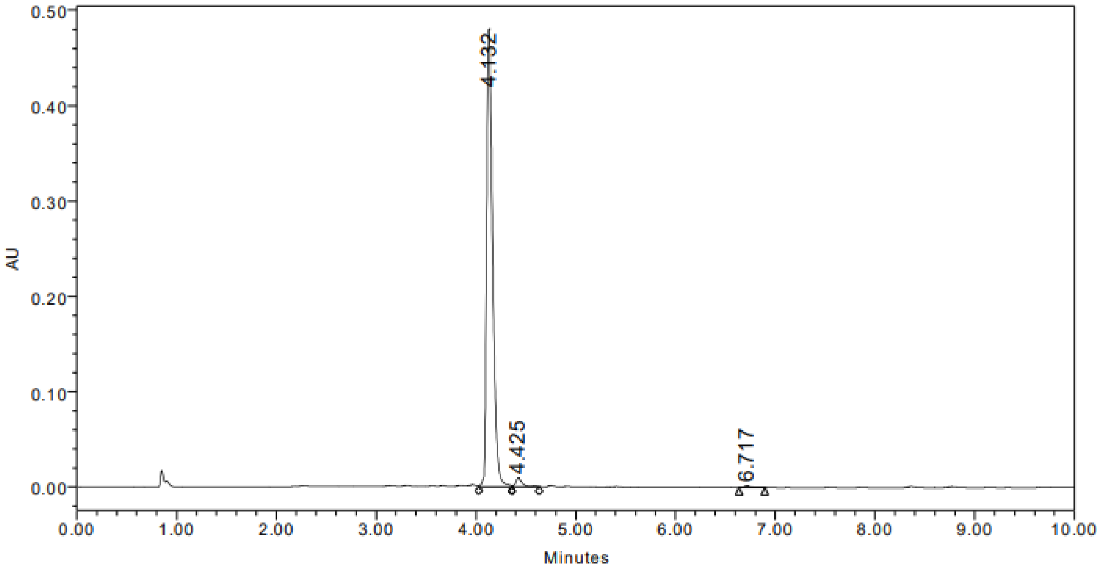

Compound 7

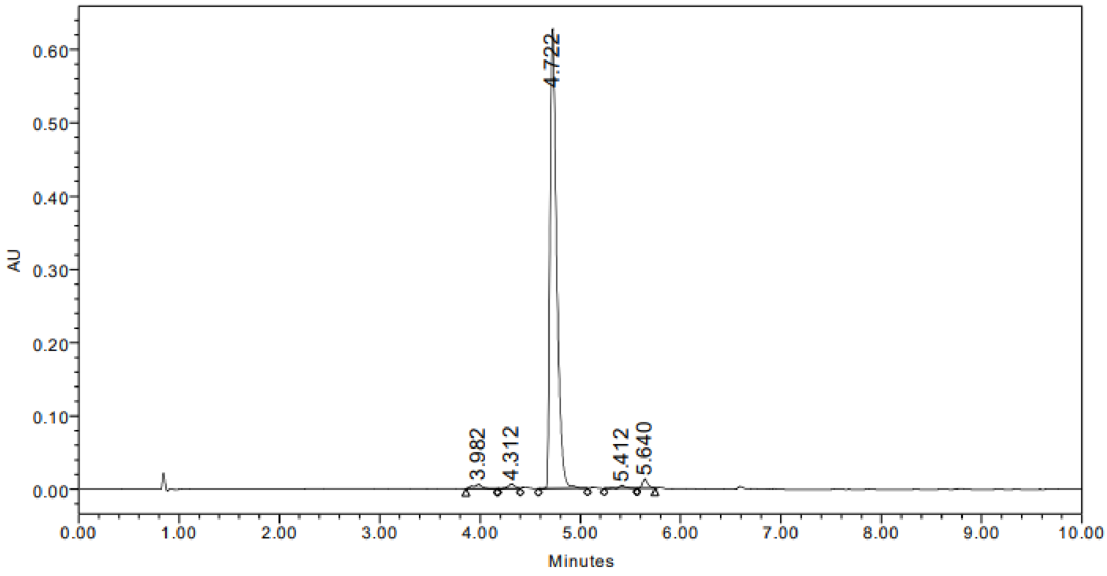

Compound 8

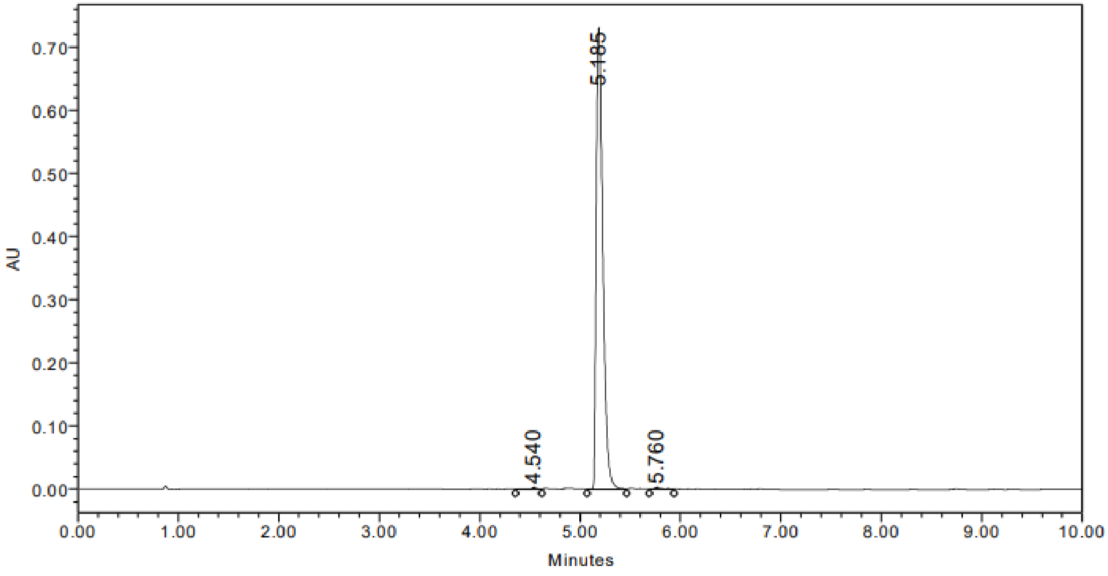

Compound 9

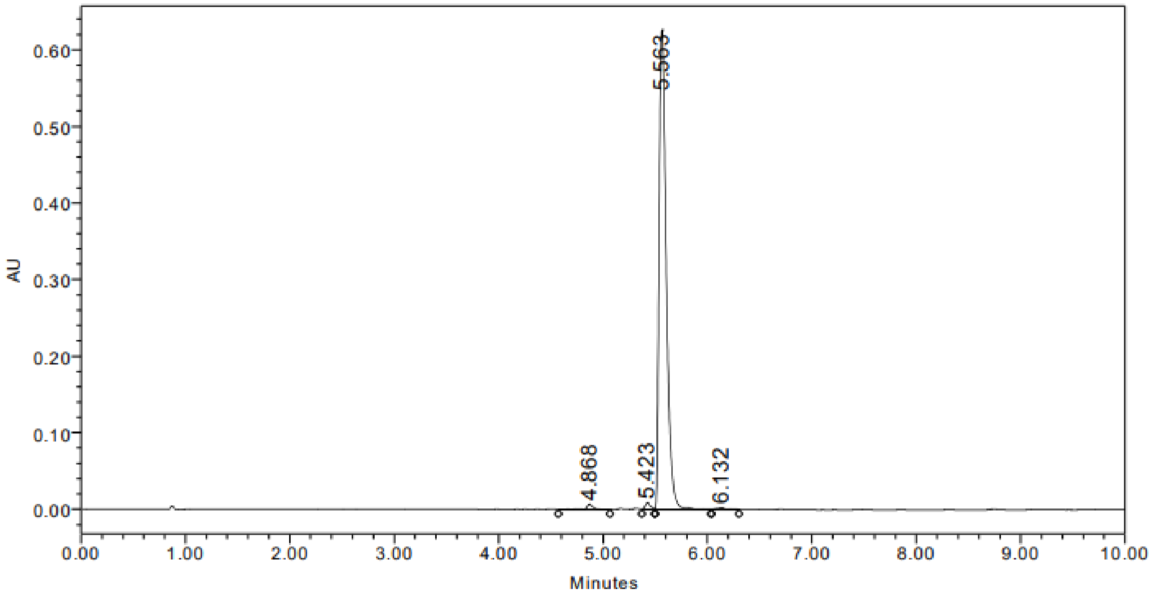

Compound 10

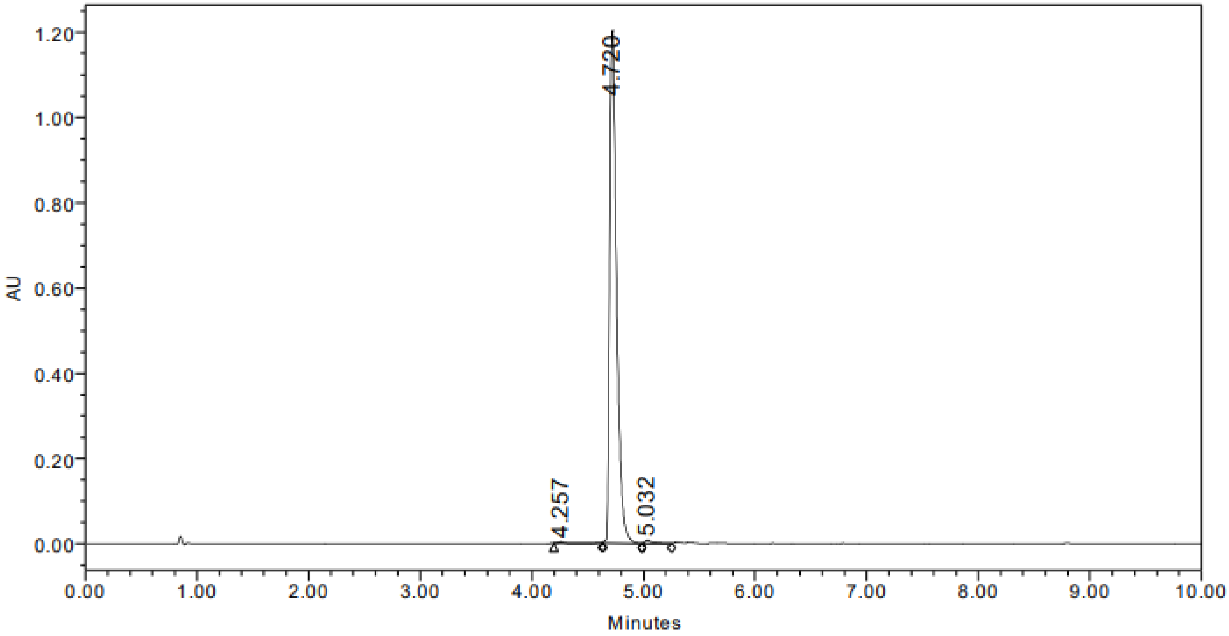

Compound 11

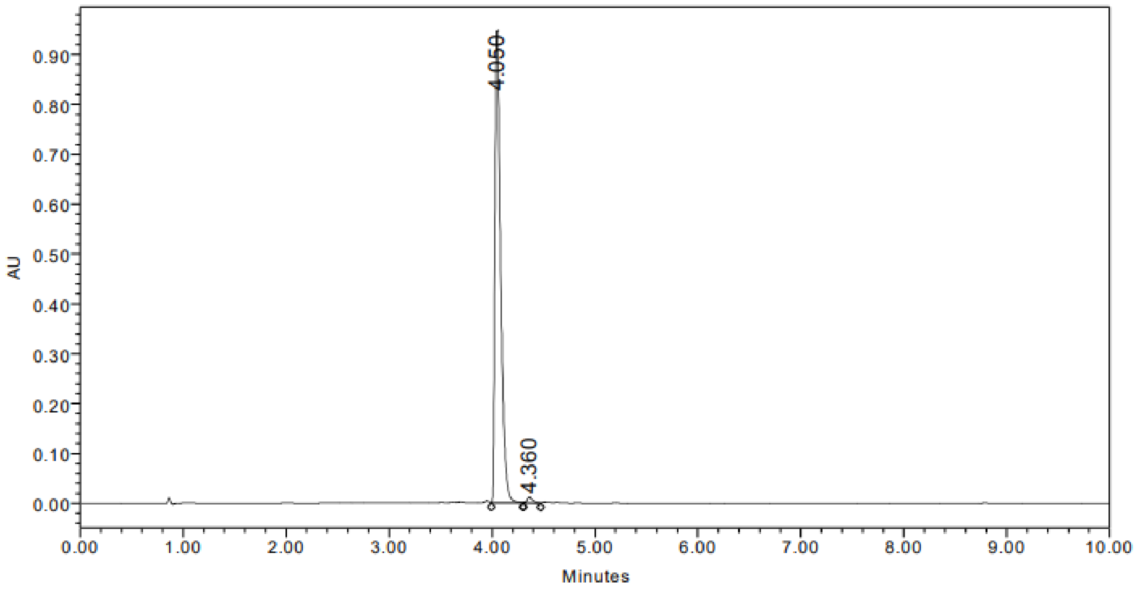

Compound 12

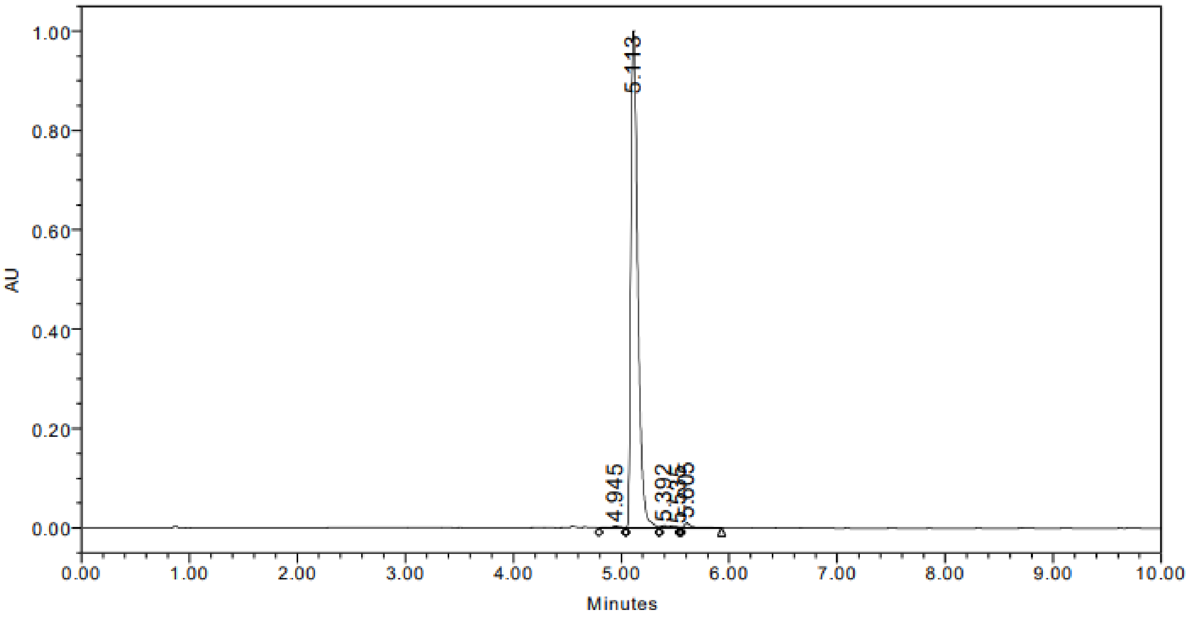

Compound 13

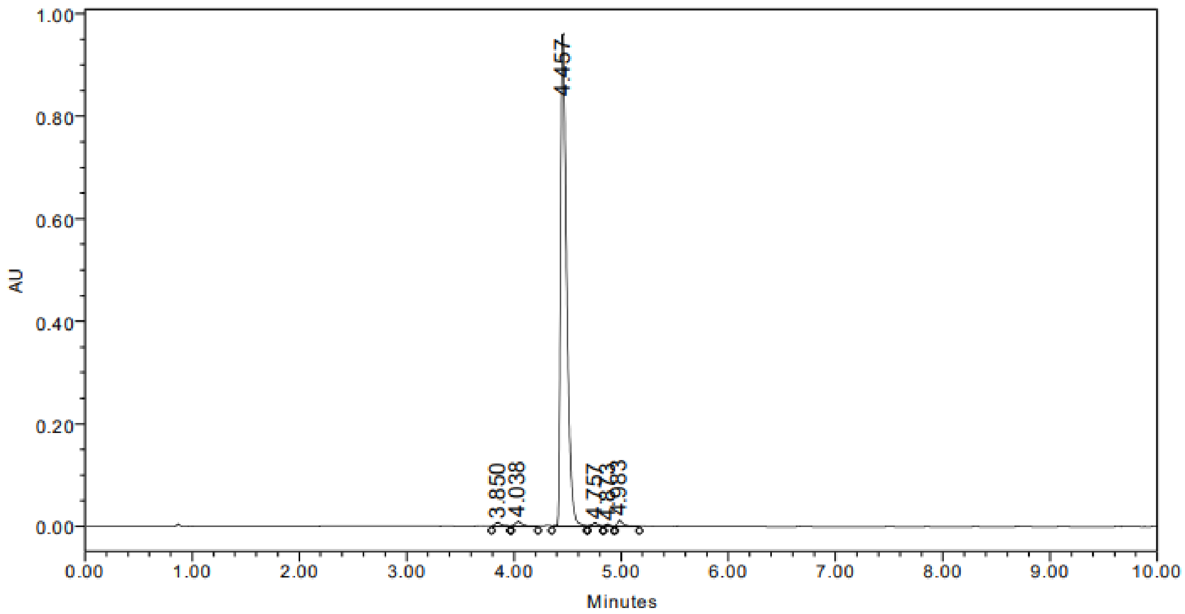

Compound 14

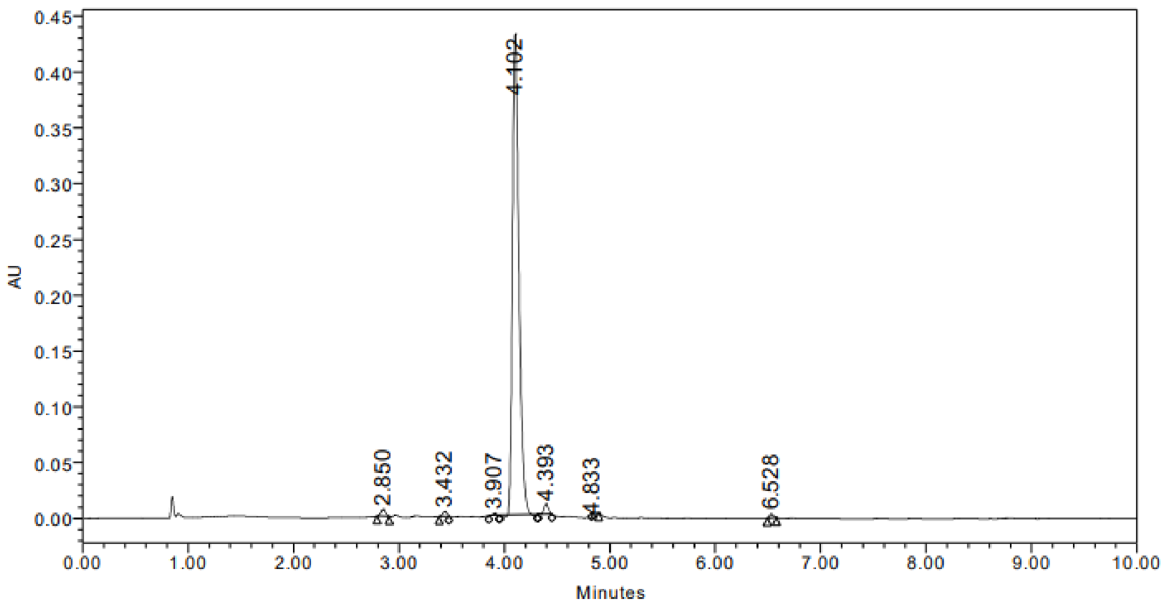

Compound 15

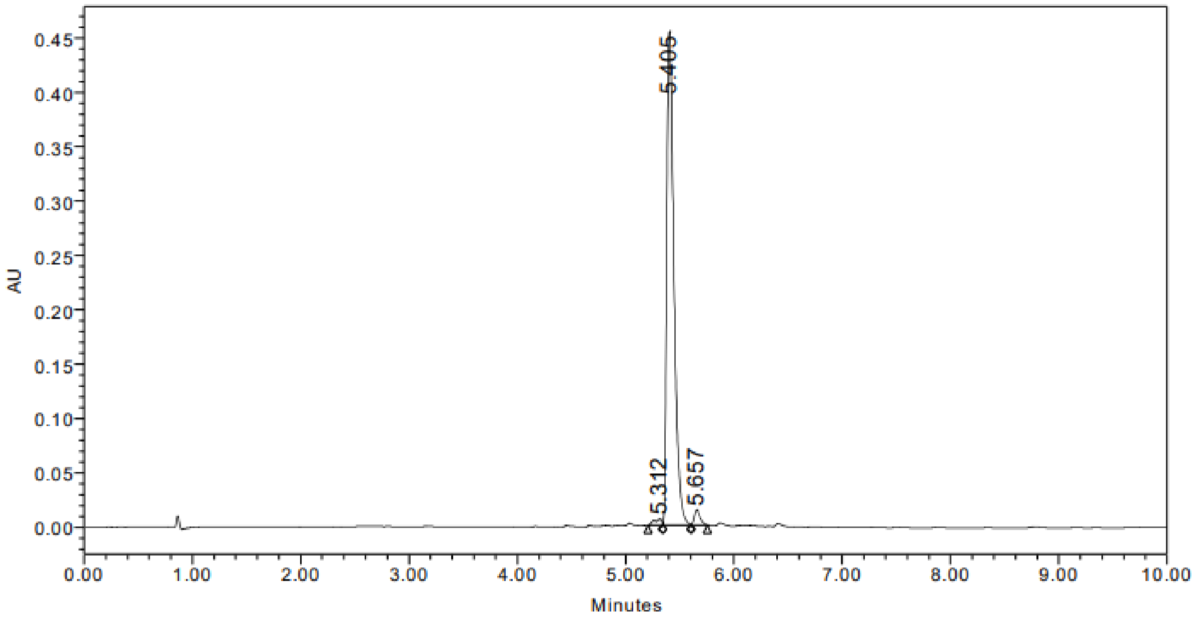

Compound 16

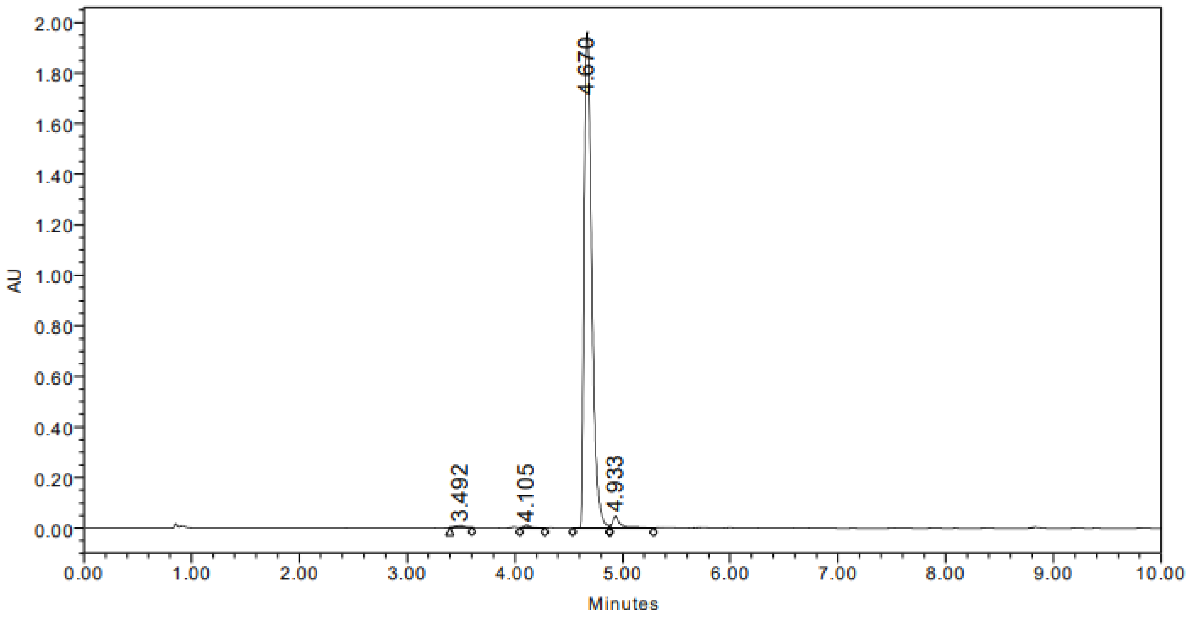

Compound 17

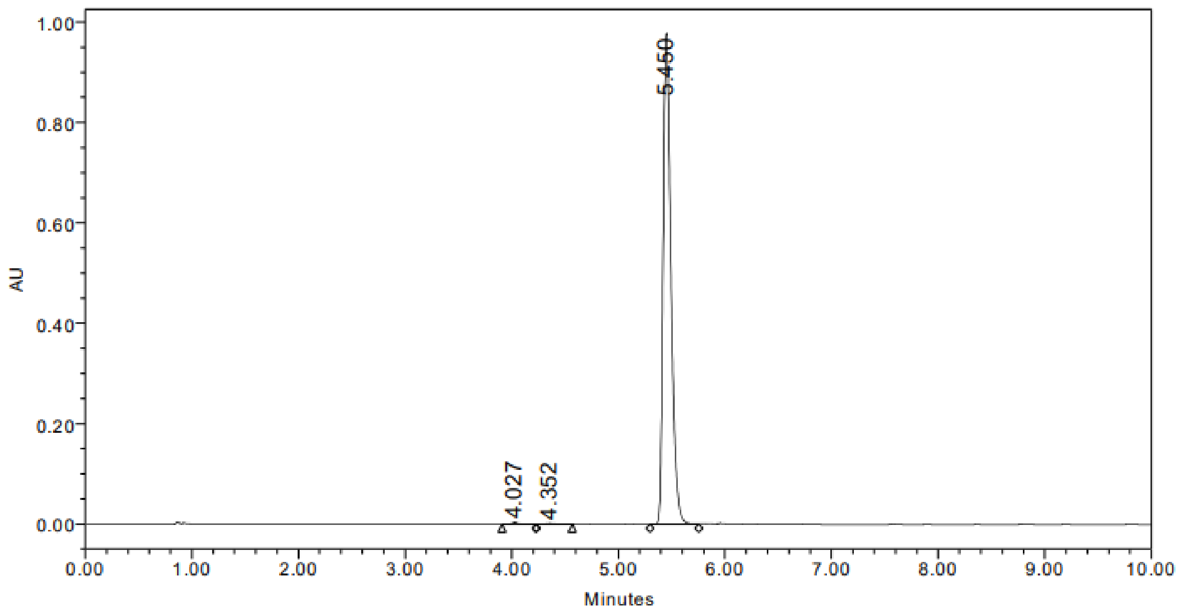

Compound 18

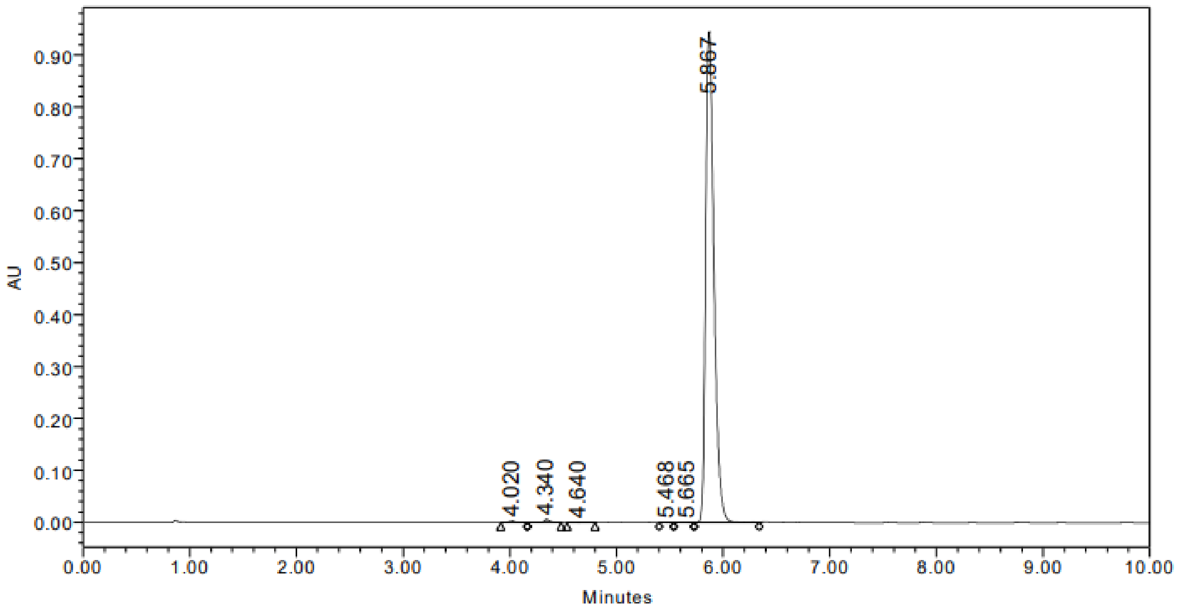

Compound 19

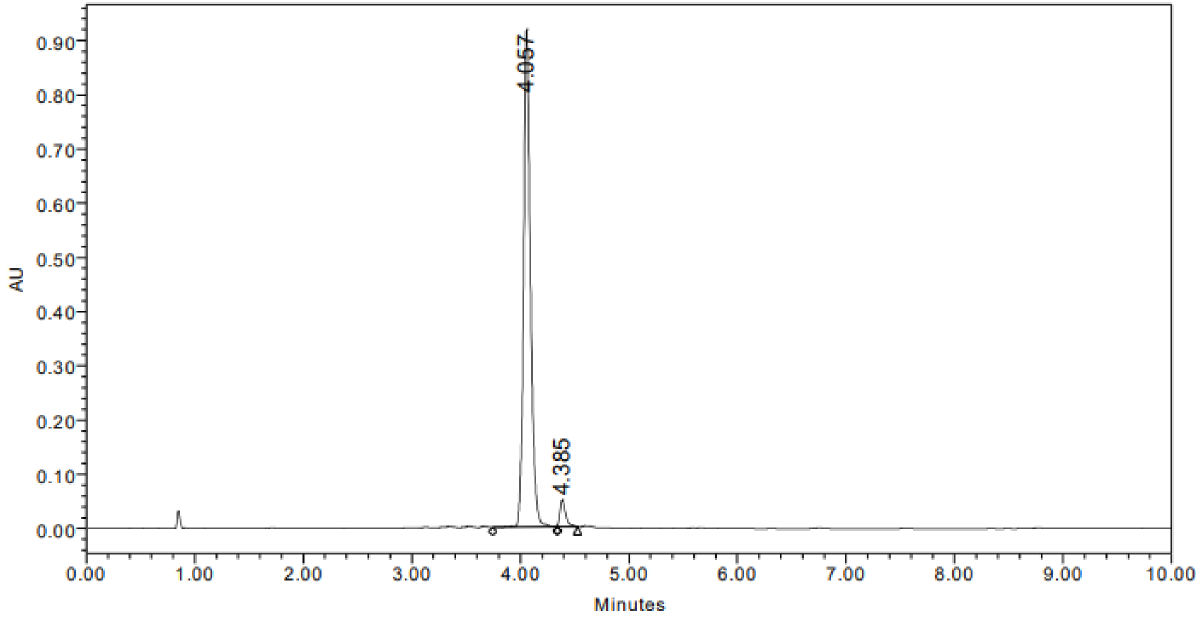

Compound 20

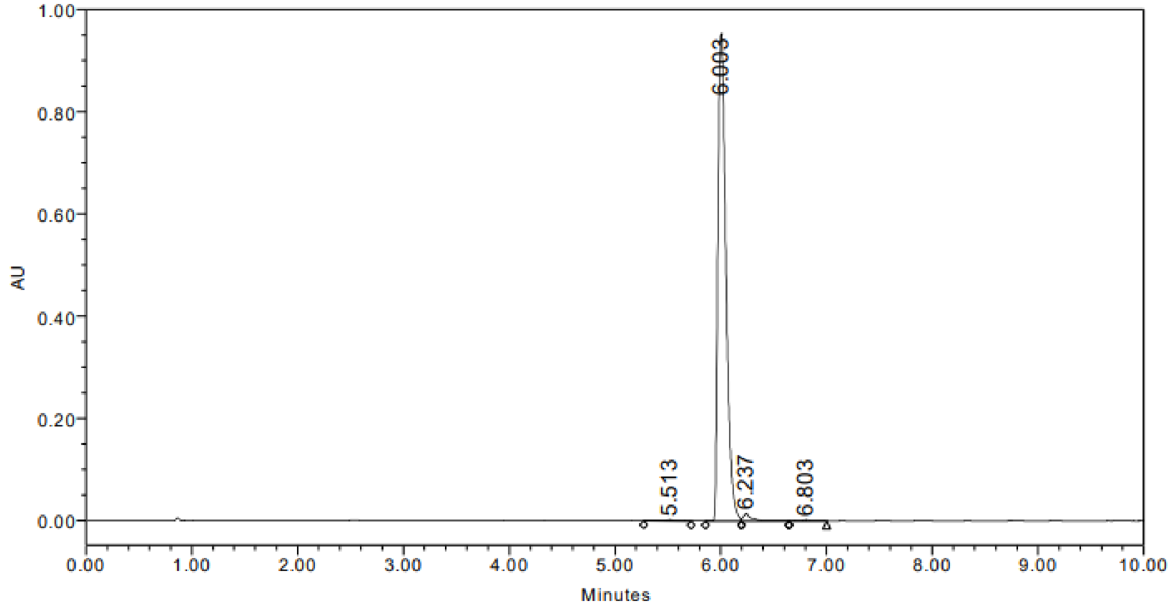

Compound 21

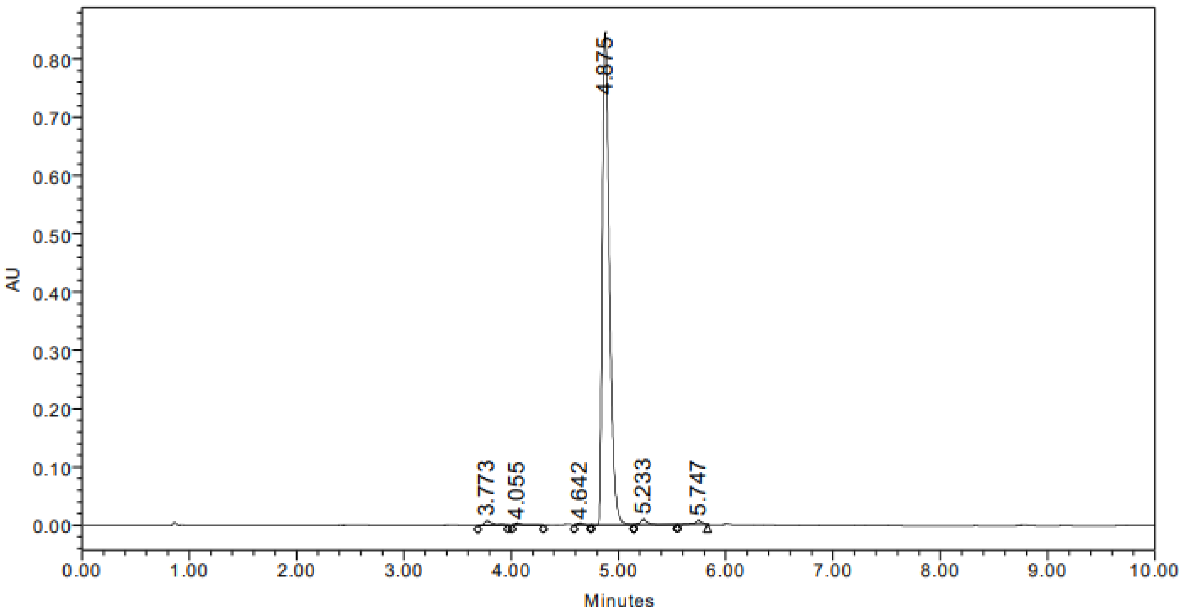

Compound 22

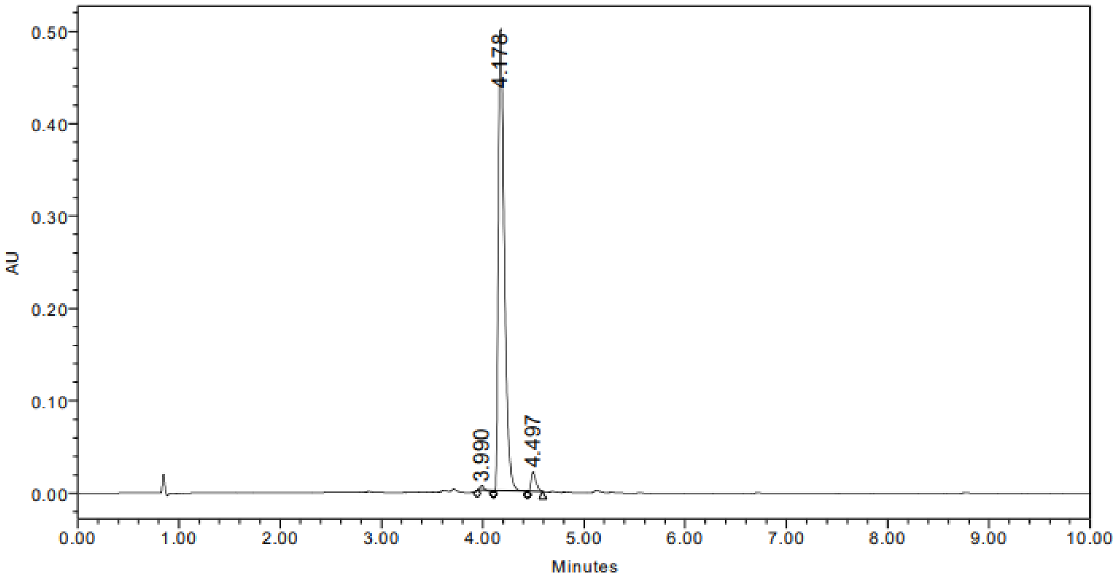

Compound 23

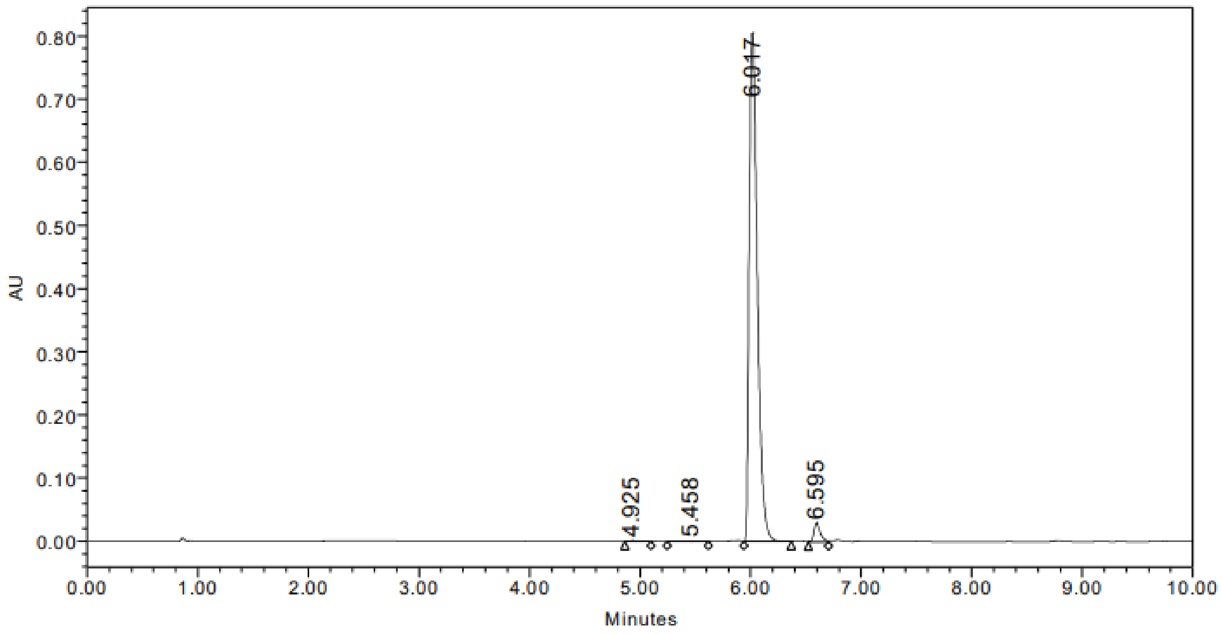

Compound 24

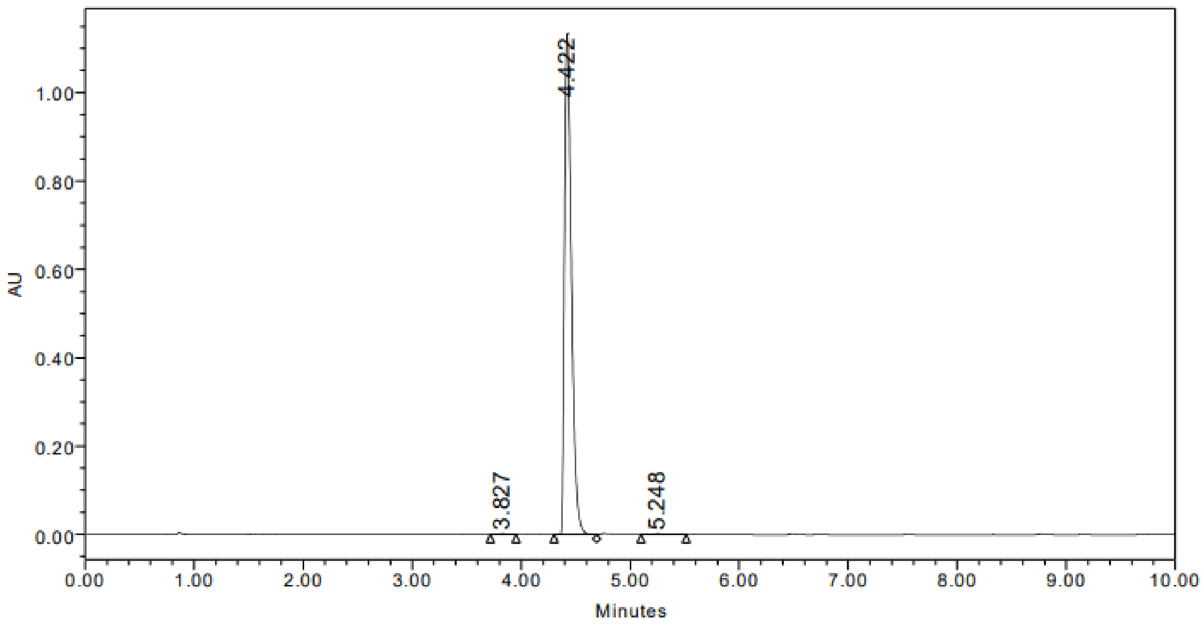

Compound 25

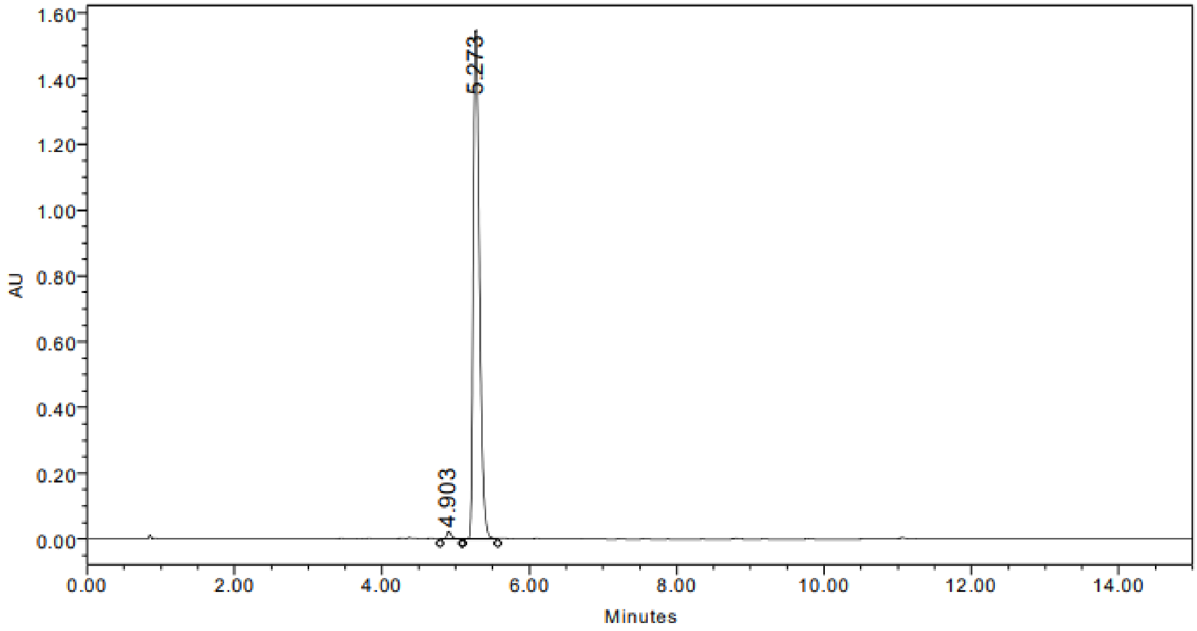

Compound 26

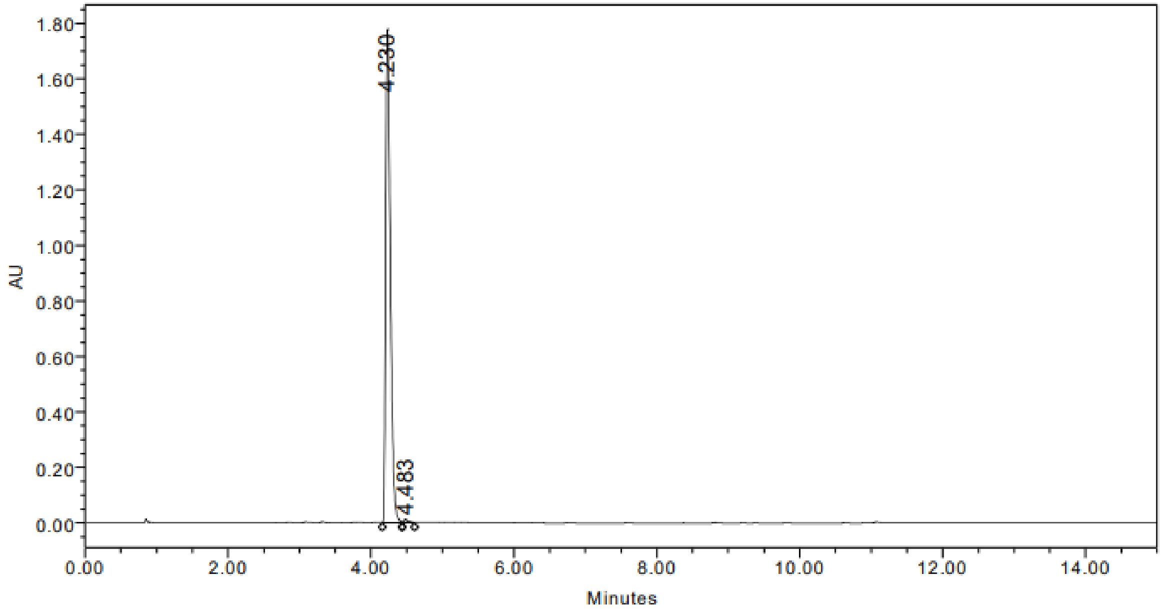

Compound 27

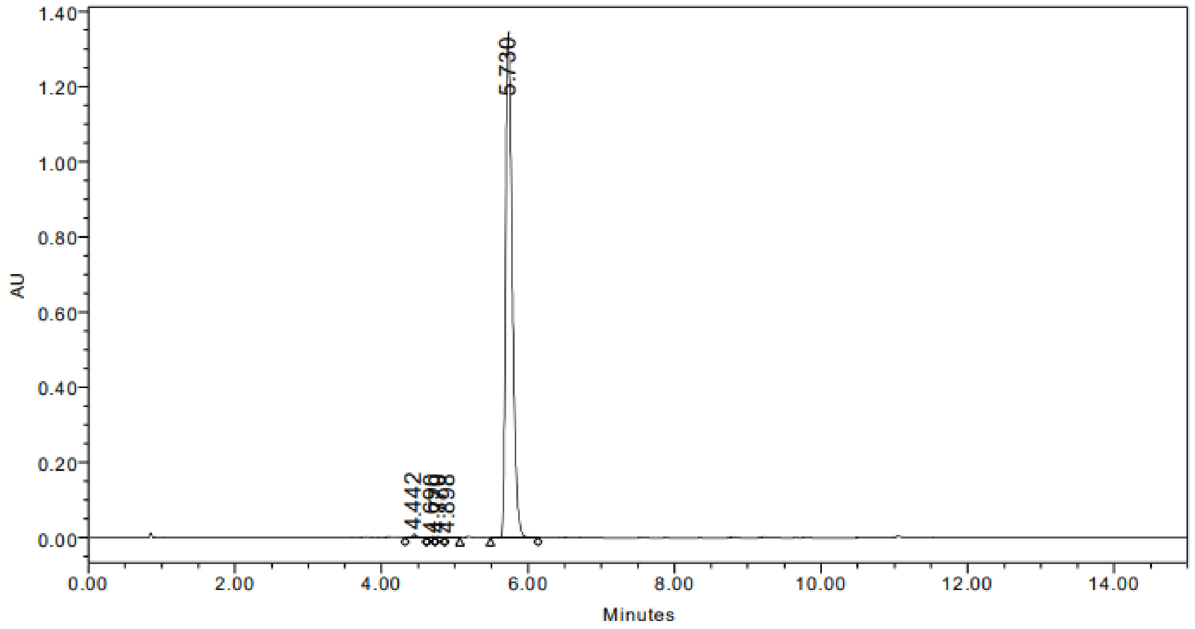

Compound 28

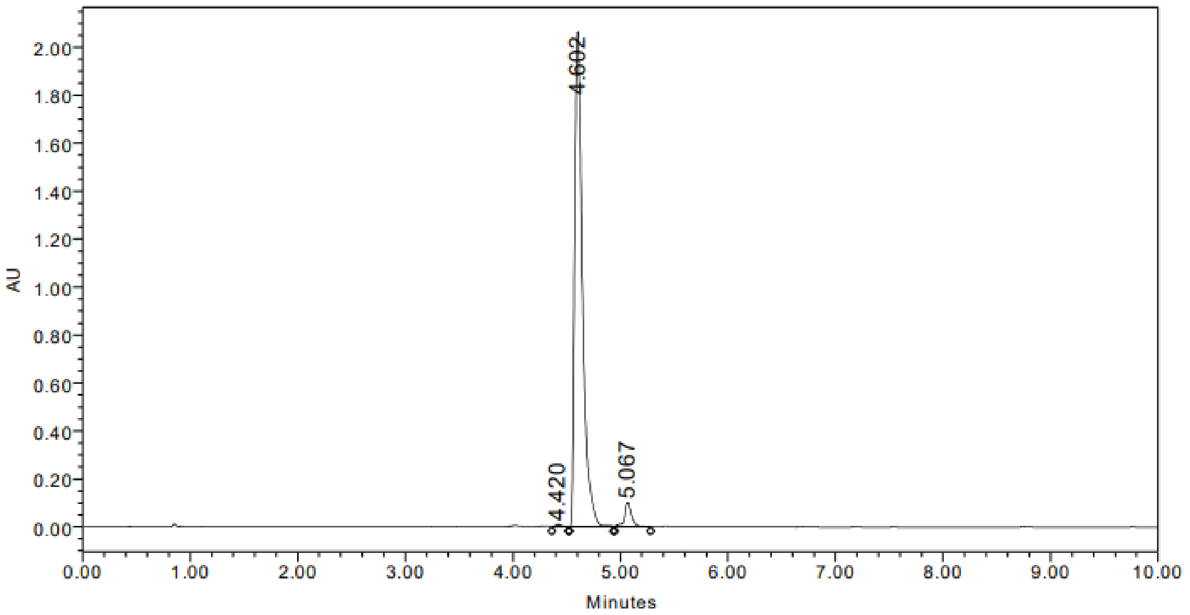

Compound 29

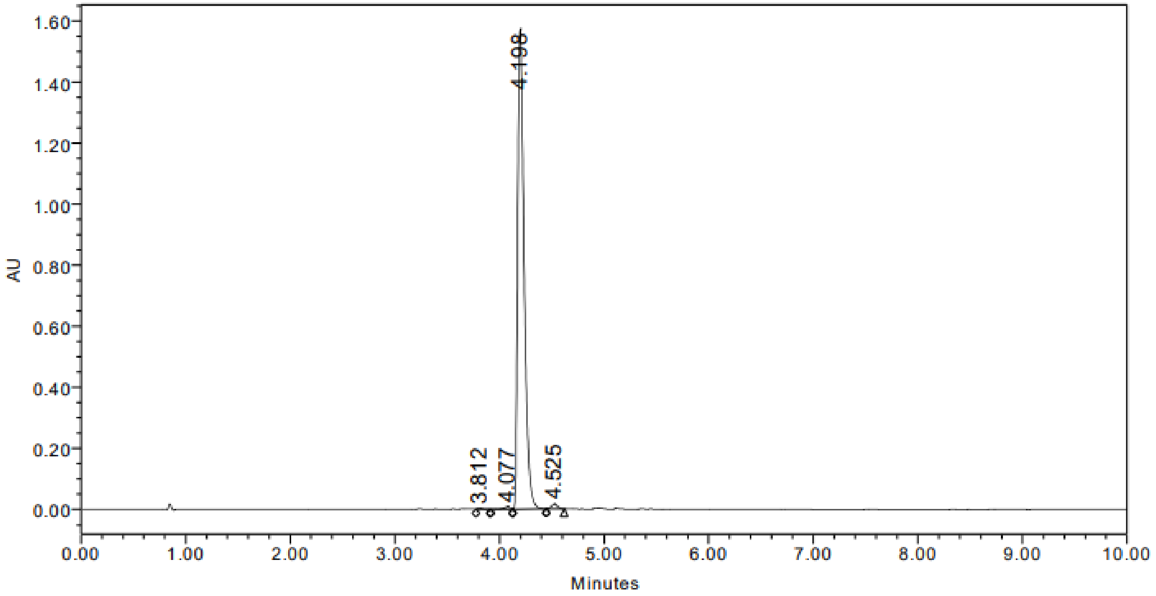

Compound 30

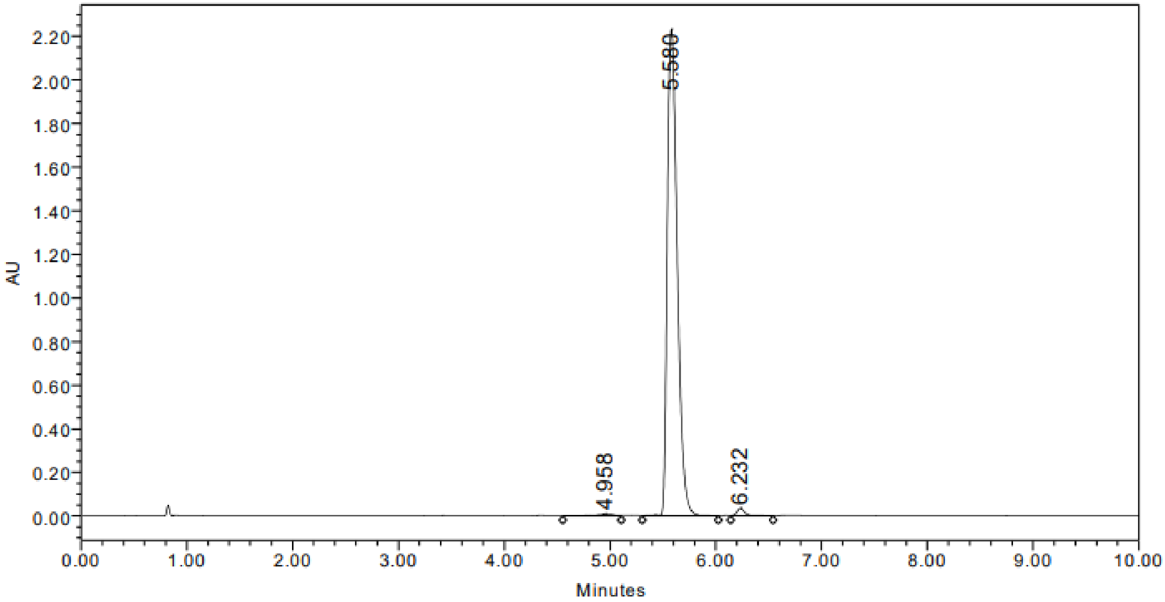

Compound 31

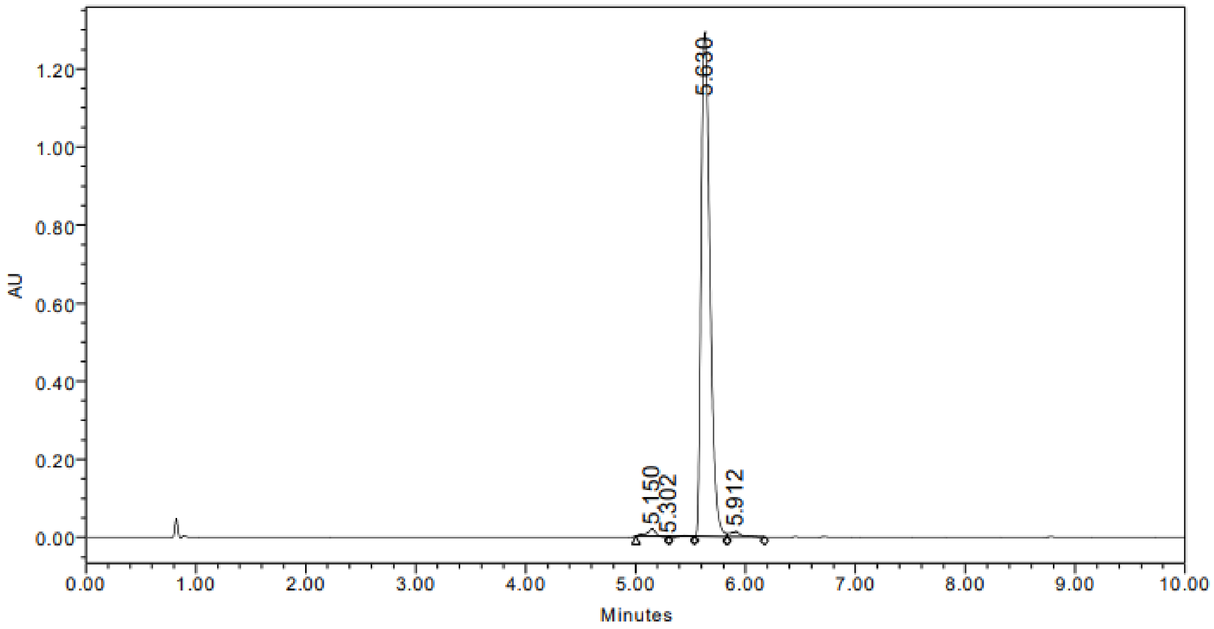

Compound 32

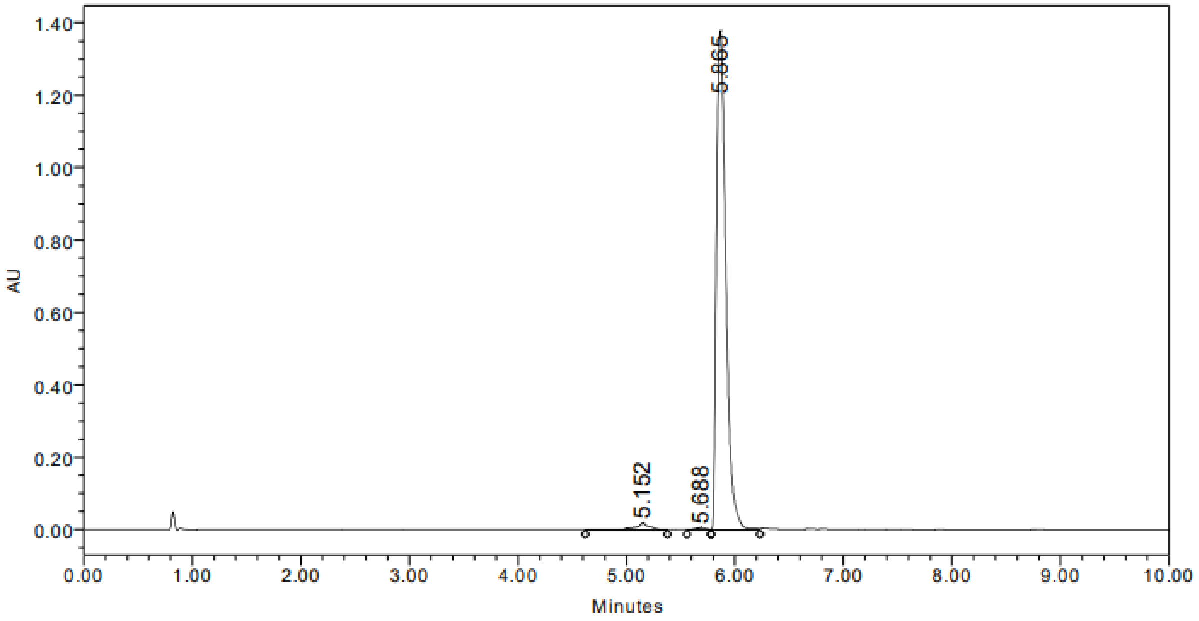

Compound 33

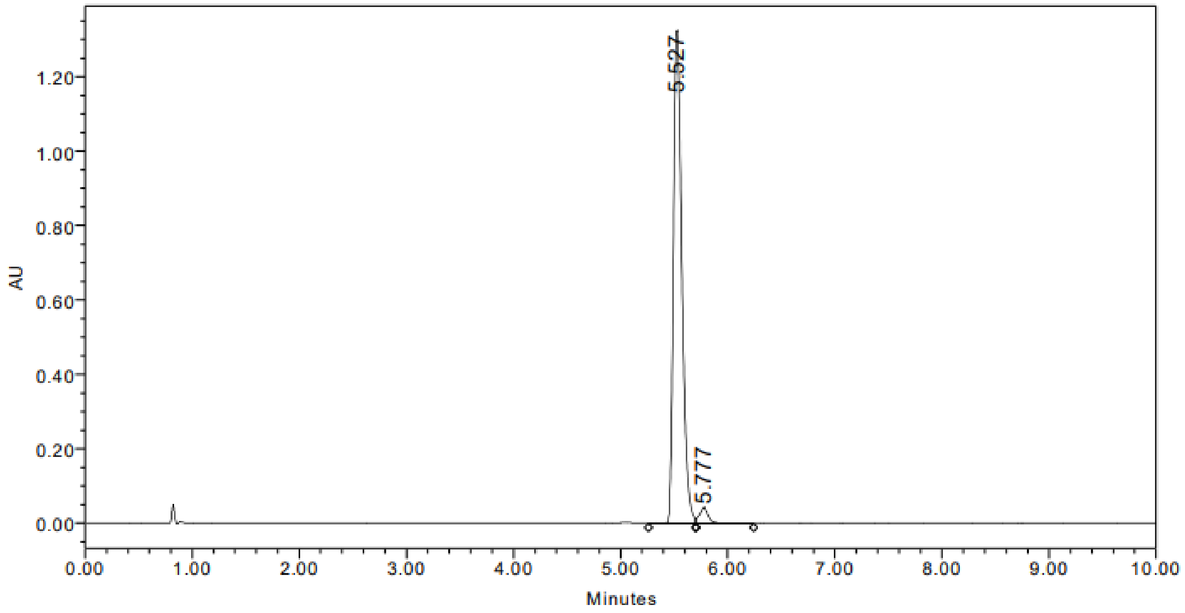

Compound 34

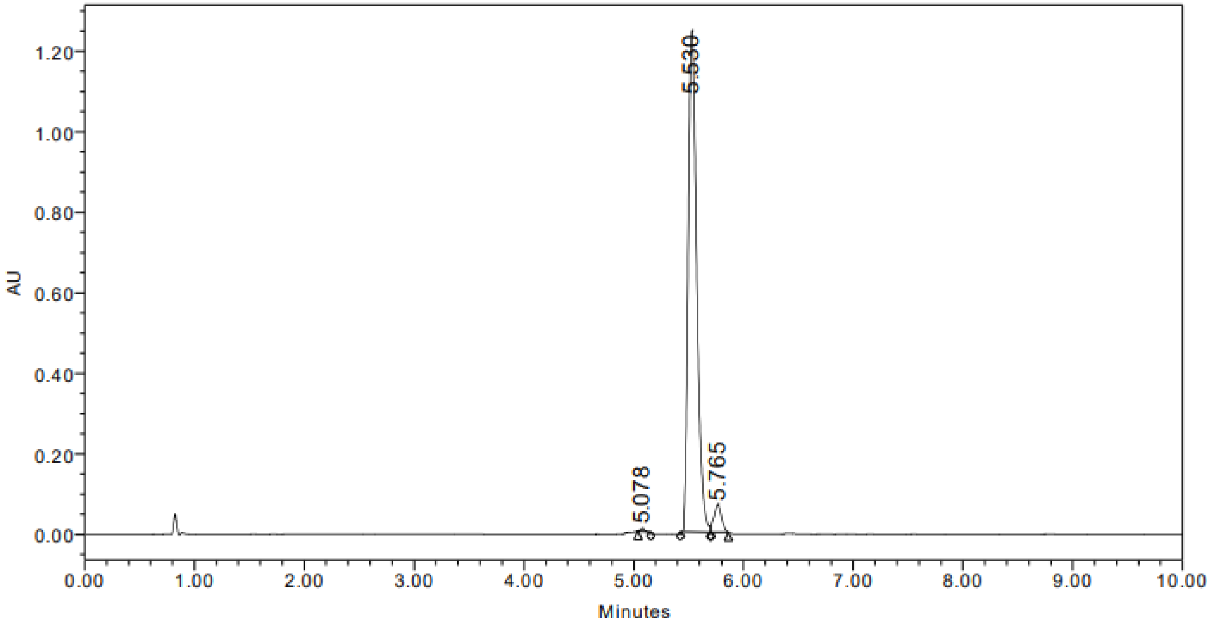

Compound 35

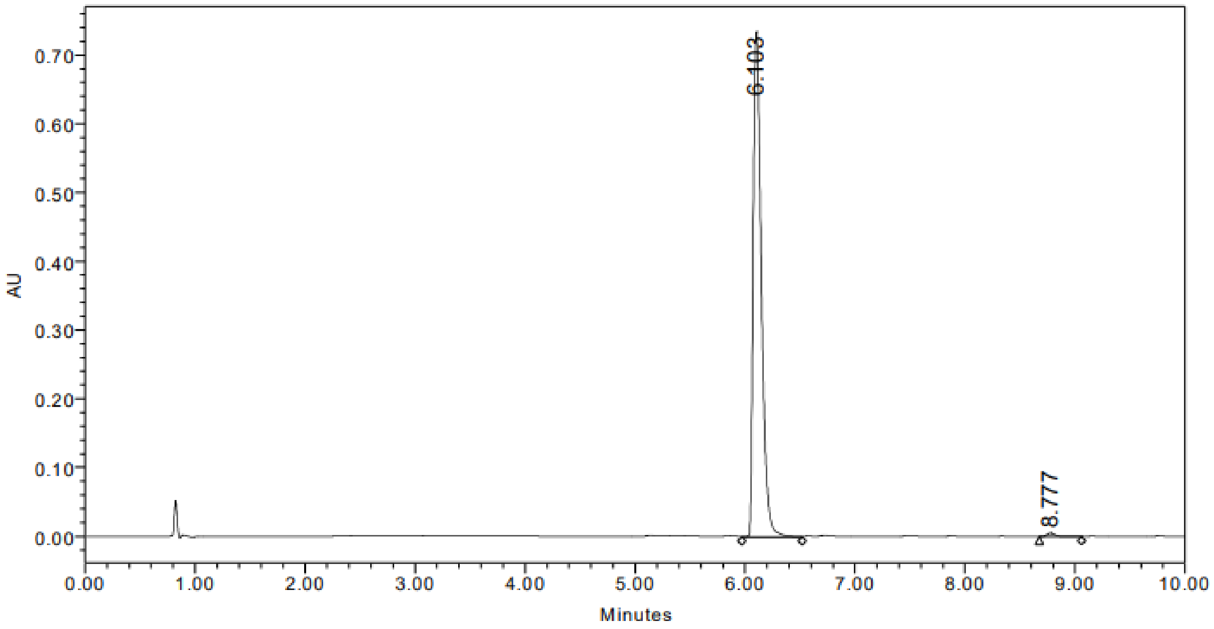

Compound 36

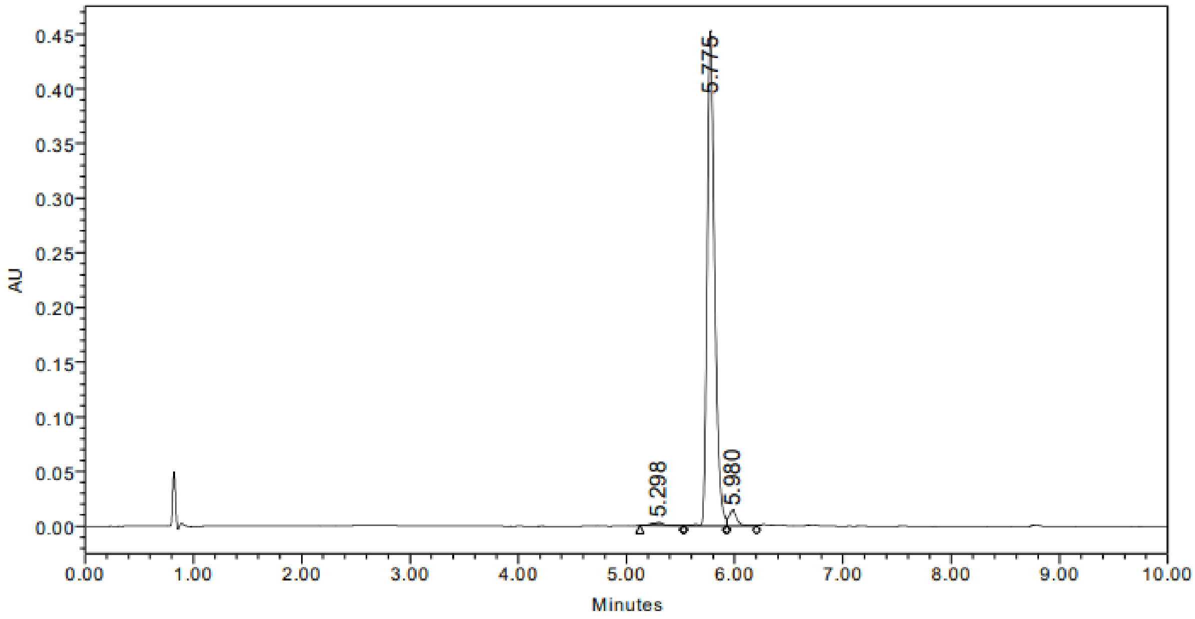

4.) Predicted physiochemical properties

Table S2. ACD/Percepta (v2020.2.0) predicted physiochemical properties

Compound	cLogP	cLogD (pH=7.40)	pKa	MW	TPSA	HBD
**1**	2.22	1.28	7.92	486.56	110.71	4
**2**	2.22	1.28	7.92	486.56	110.71	4
**3**	2.01	0.86	7.81	486.56	110.71	4
**4**	2.01	0.86	7.81	486.56	110.71	4
**5**	2.01	0.78	7.88	486.56	110.71	4
**6**	2.01	0.78	7.88	486.56	110.71	4
**7**	2.01	0.82	7.92	486.56	110.71	4
**8**	2.01	0.82	7.92	486.56	110.71	4
**9**	2.17	1.29	7.91	486.56	110.71	4
**10**	2.17	1.29	7.91	486.56	110.71	4
**11**	1.83	0.80	7.87	486.56	110.71	4
**12**	1.83	0.80	7.87	486.56	110.71	4
**13**	1.83	0.70	7.91	486.56	110.71	4
**14**	1.83	0.70	7.91	486.56	110.71	4
**15**	1.73	1.29	7.91	486.56	110.71	4
**16**	1.73	1.29	7.91	486.56	110.71	4
**17**	2.17	1.26	7.91	486.56	110.71	4
**18**	2.17	1.26	7.91	486.56	110.71	4
**19**	1.73	0.83	7.90	486.56	110.71	4
**20**	1.73	0.83	7.90	486.56	110.71	4
**21**	1.83	0.81	7.91	486.56	110.71	4
**22**	1.83	0.81	7.91	486.56	110.71	4
**23**	1.83	0.73	7.91	486.56	110.71	4
**24**	1.83	0.73	7.91	486.56	110.71	4
**25**	1.73	1.30	7.86	486.56	110.71	4
**26**	1.73	1.30	7.86	486.56	110.71	4
**27**	2.22	1.30	7.90	486.56	110.71	4
**28**	2.22	1.30	7.90	486.56	110.71	4
**29**	1.78	0.87	7.88	486.56	110.71	4
**30**	1.78	0.87	7.88	486.56	110.71	4
**31**	2.01	0.83	7.91	486.56	110.71	4
**32**	2.01	0.83	7.91	486.56	110.71	4
**33**	2.01	0.91	7.87	486.56	110.71	4
**34**	2.01	0.91	7.87	486.56	110.71	4
**35**	1.78	1.24	7.79	486.56	110.71	4
**36**	1.78	1.24	7.79	486.56	110.71	4

MW= molecular weight

TPSA=total polar surface area

HBD=hydrogen bond donor

The polar surface area (approximated by FISA using Schrödinger's QikProp) for Compound 7 was calculated to be 113.16 Å^2. This value serves as a surrogate for the exposed polar surface area (EPSA), which is commonly used to assess passive permeability and predict CNS penetration.

5.) Molecular modeling

A.

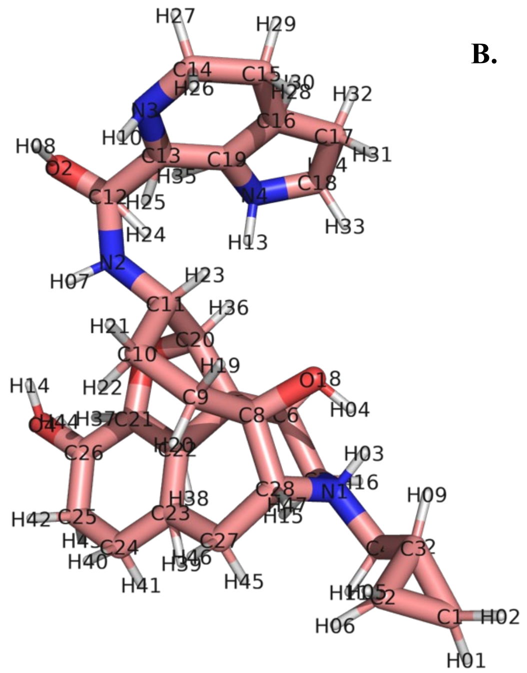

B.

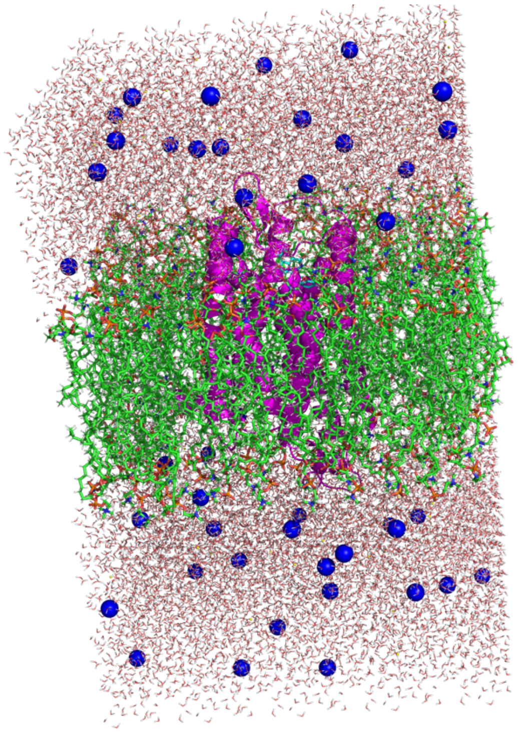

Figure S1. A. The three-dimension chemical structure of compound **7** with atom notation. **B.** A ligand-receptor complex within the POPC lipid membrane solvated by TIP3 water layers. The protein shown as cartoon model in Magenta; ligand shown as stick model in cyan; POPC lipid membrane shown as stick model in green; water molecule shown as line model in red. Sodium and chloride ion shown as sphere model in blue and yellow, respectively.

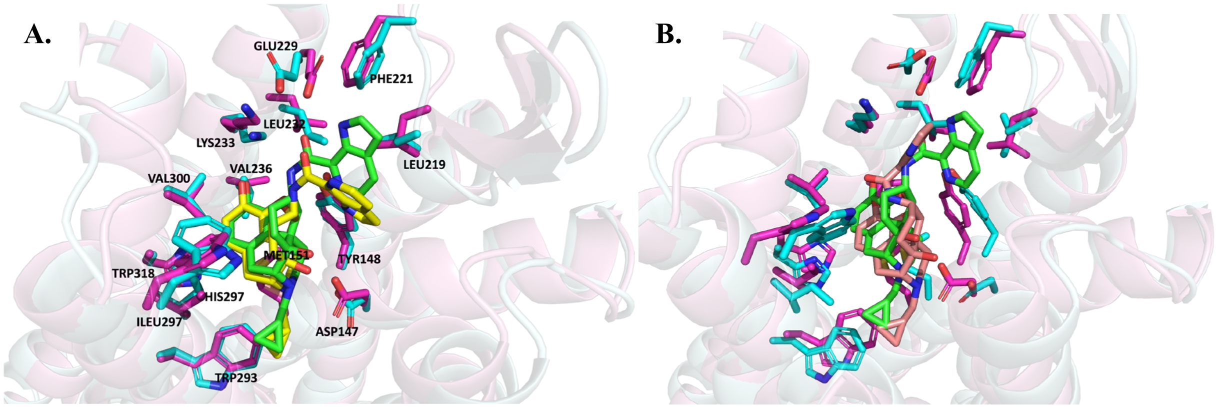

Figure S2A. Alignment of **docked pose (grey cartoon),** and the binding pocket after 200 ns **MD simulation (light pink cartoon) of compound 7** with the inactive MOR; (PDB 4DKL)[1]. **Compound 7** and key amino acid residues are shown on the sticks. Carbon atoms: **Compound 7 after MD** (green); **Compound 7** after docking (yellow); key amino acid residues (Magenta); oxygen atoms (red); nitrogen atoms (blue). **Figure S2B.** Alignment of the binding pose of

NAN and **Compound 7** in the inactive MOR with key residues in the binding pocket (PDB 4DKL)[1] after MD simulation. The MOR is shown as light pink cartoons for **compound 7** and light grey for NAN. NAN, Compound 7, and key amino acid residues are shown on the sticks. Carbon atoms: NAN (salmon); Compound 7 (green); key amino acid residues for compound 7 (cyan) (key amino residues for NAN (Magenta)); oxygen atoms (red); nitrogen atoms (blue).

Table S3. Measured Shortest Distances between Atoms on Critical Amino Acid Residues and Atoms on the Ligand with MOR after docking and 2000 ns MD Simulations

Ligand domain	Atom of ligand	Atom of residue	Distance after docking (Å)	Distance after 200 ns MD (Å)
message	N1	OD2@D147	3.2	2.8
	O3	OH@Y148	2.6	3.0
	C06	CE@M151	3.3	3.8
	C04	CH2@W293	3.4	3.7
	C27	CG1@I296	3.7	3.8
	C25	CE1@H297	4.3	3.9
	C09	CH2@W318	5.6	5.0
address	C17	CB@L219	4.0	5.3
	C18	CZ@F221	8.6	6.6
	N04	OE2@E229	8.1	2.7
	C12	CE@K233	3.8	3.9
